# Switchable, Reagent‐Controlled Diastereodivergent Photocatalytic Carbocyclisation of Imine‐Derived α‐Amino Radicals

**DOI:** 10.1002/anie.202107253

**Published:** 2021-10-13

**Authors:** J. Andrew P. Maitland, Jamie A. Leitch, Ken Yamazaki, Kirsten E. Christensen, Doyle J. Cassar, Trevor A. Hamlin, Darren J. Dixon

**Affiliations:** ^1^ Department of Chemistry Chemistry Research Laboratory University of Oxford 12 Mansfield Road Oxford OX1 3TA UK; ^2^ Current address: Department of Pharmaceutical and Biological Chemistry UCL (University College London) School of Pharmacy 29–39 Brunswick Square London WC1N 1AX UK; ^3^ Department of Theoretical Chemistry Amsterdam Institute of Molecular and Life Sciences (AIMMS) Amsterdam Center for Multiscale Modeling (ACMM) Vrije Universiteit Amsterdam De Boelelaan 1083 1081 HV Amsterdam The Netherlands; ^4^ Oncology R&D AstraZeneca Cambridge CB4 0WG UK

**Keywords:** diastereoselectivity, imines, photocatalysis, redox reactions, reduction

## Abstract

A reagent‐controlled stereodivergent carbocyclisation of aryl aldimine‐derived, photocatalytically generated, α‐amino radicals possessing adjacent conjugated alkenes, affording either bicyclic or tetracyclic products, is described. Under net reductive conditions using commercial Hantzsch ester, the α‐amino radical species underwent a single stereoselective cyclisation to give trans‐configured amino‐indane structures in good yield, whereas using a substituted Hantzsch ester as a milder reductant afforded cis‐fused tetracyclic tetrahydroquinoline frameworks, resulting from two consecutive radical cyclisations. Judicious choice of the reaction conditions allowed libraries of both single and dual cyclisation products to be synthesised with high selectivity, notable predictability, and good‐to‐excellent yields. Computational analysis employing DFT revealed the reaction pathway and mechanistic rationale behind this finely balanced yet readily controlled photocatalytic system.

## Graphical abstract

Through judicious choice of reductant, a switchable diastereodivergent cyclisation of a single alkene‐tethered imine, to either a *trans*‐fused amino‐indane or a *cis*‐fused tetracyclic tetrahydroquinoline structure, is herein described.

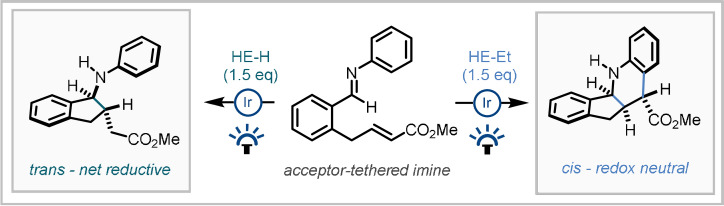

## Introduction

Recently, the photocatalytic generation and downstream reactivity of α‐amino radical species have attracted a lot of attention across the synthetic community.^[1]^ These high‐value reactive intermediates exhibit nucleophilic behaviour at the position alpha to the nitrogen atom, and have been shown to undergo various radical‐radical coupling reactions,^[2]^ to add to electrophilic acceptors,^[3]^ and to intercept dual catalytic manifolds.^[4]^ To this end, contemporary photocatalytic developments have enabled the generation of these key nucleophilic α‐amino radical species from inherently electrophilic imine derivatives via single‐electron reduction (often with concomitant proton transfer in a PCET mechanism).^[5]^ This umpolung approach to amine synthesis is complementary to traditional two‐electron conversions, and thus has expanded the range of α‐branched amine architectures that can be accessed from simple and readily obtained imine precursors.^[6]^

**Scheme 1:**
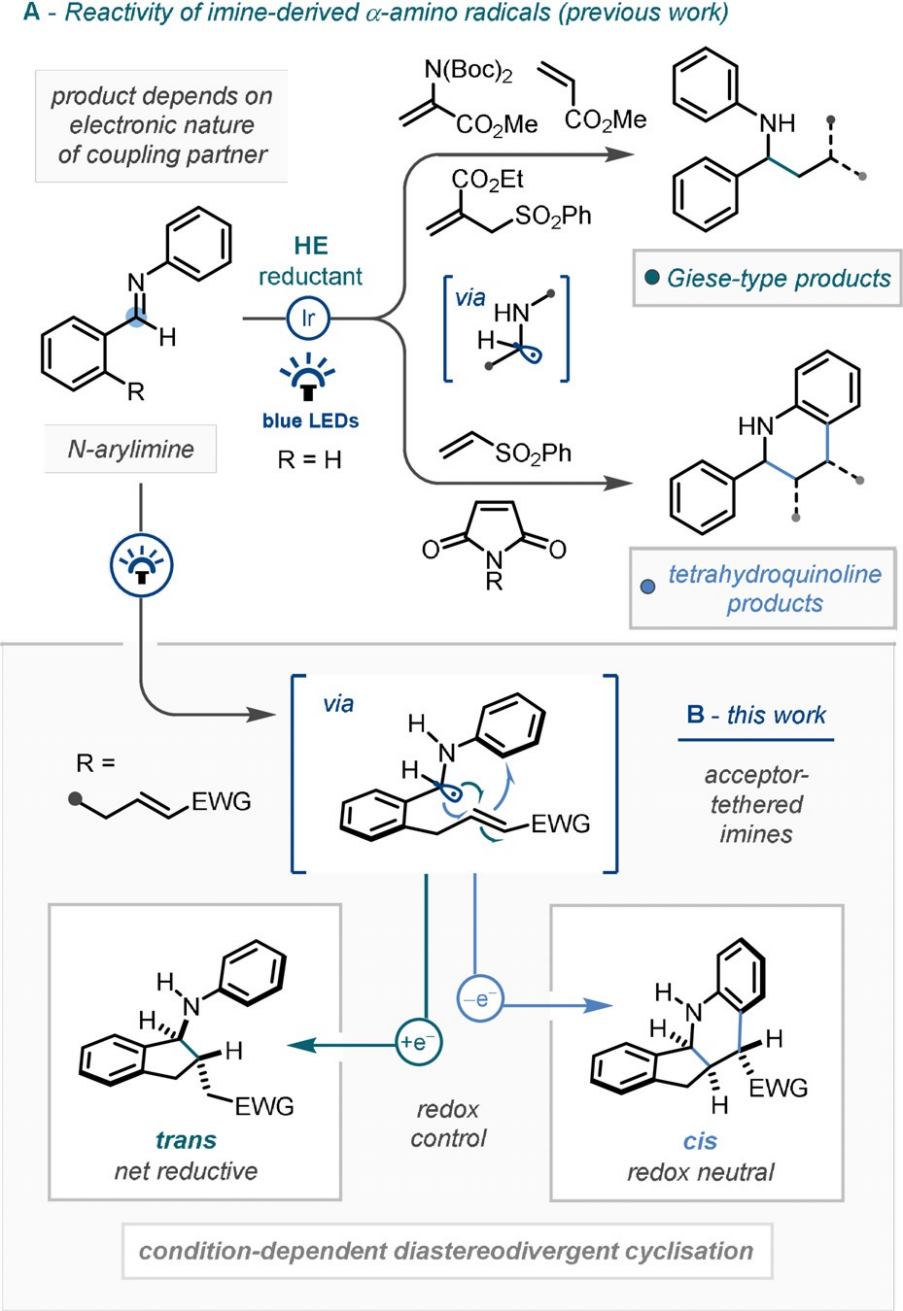
Reductive photocatalytic functionalisation of imines in the context of this work.

Previous studies from our group have revealed two distinct product‐forming pathways under photocatalytic conditions, where the outcome depended almost exclusively on the nature of the electrophilic coupling partner (Scheme 1 A). Firstly, the net reductive Giese‐type products were demonstrated to be favoured when coupling with α,β‐unsaturated esters (such as allyl sulfones, and dehydroalanine derivatives).^[7]^ Secondly, tetrahydroquinoline products were formed through a redox‐neutral reverse‐polarity Povarov‐type radical cascade mechanism, whose pathway was favoured by vinyl sulfone and maleimide derivatives.^[8]^ Notably, in the latter cases, the alternative Giese‐type product was not obtained, even with suitable adjustment of the reaction conditions.

In a continuation of our research programme into establishing new reactivity of imine‐derived α‐amino radical species, we sought to explore the performance of electrophile‐tethered imines as a means of achieving intramolecular C−C bond formation, thereby constructing complex (poly)cyclic molecular frameworks (Scheme 1 B). Whilst singly cyclised Giese‐type amino‐indanes or doubly cyclised tetrahydroquinoline structures could feasibly result, the predominant outcome would depend on many competing factors, some of which we hoped to control in order to direct the reaction towards specific valuable products.[^9^, ^10^] Herein we wish to report our findings.

## Results and Discussion

**Scheme 2:**
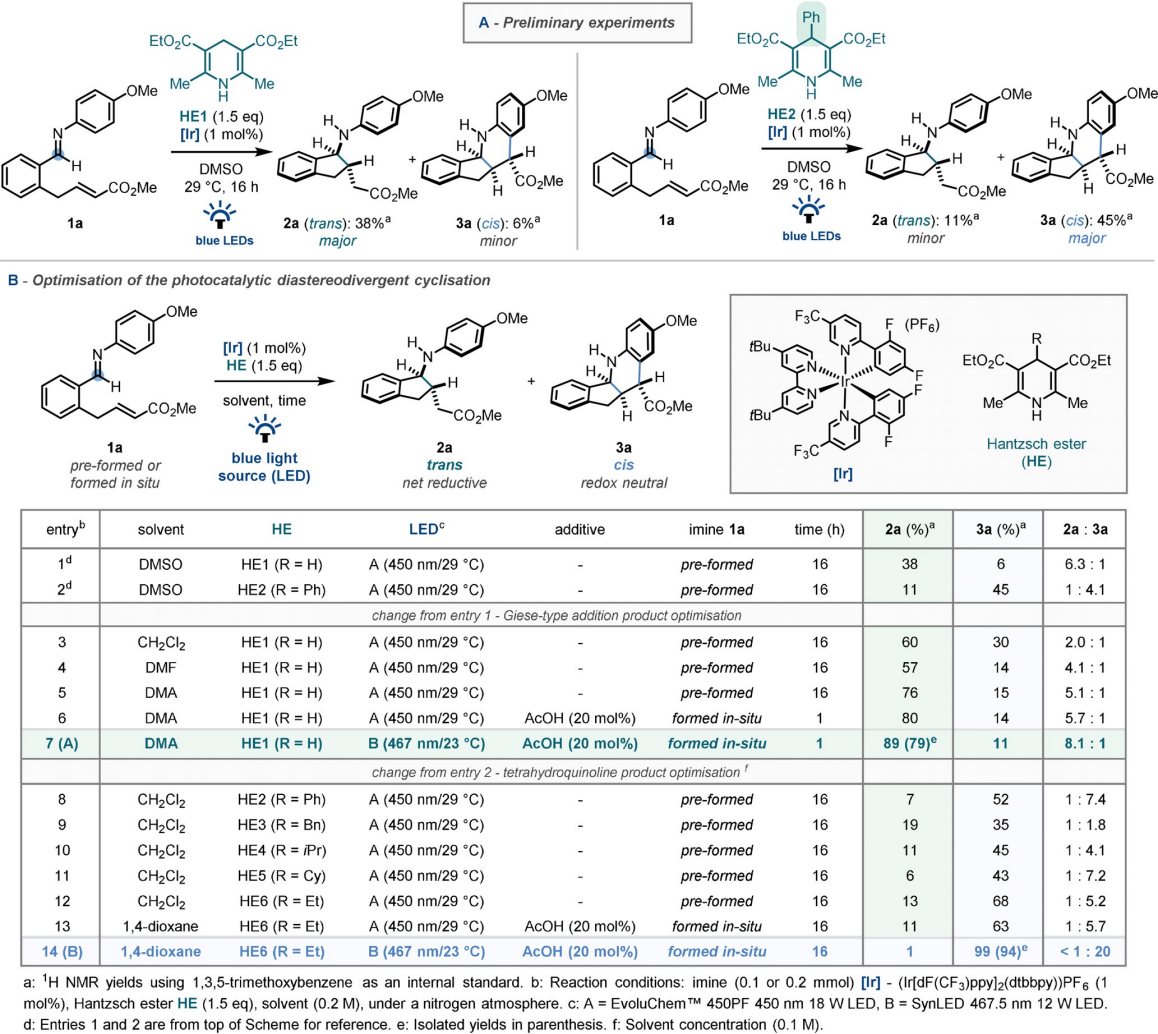
A) Preliminary experiments and B) optimisation for the diastereodivergent carbocyclisation.

Preliminary experiments were carried out using an α,β‐unsaturated ester tethered imine (**1 a**), (Ir[dF(CF_3_)ppy]_2_(dtbbpy))PF_6_ as photocatalyst, the commercial Hantzsch ester (**HE1**) as a stoichiometric reductant, in DMSO and under blue light irradiation (Scheme 2 A, left). Under these initial conditions, the net reductive C−C coupled Giese‐type amino‐indane product was pleasingly observed in modest yields (**2 a**, 38 %), with only the *trans*‐configured diastereomer present. Interestingly, the structurally complex *cis*‐fused tetracyclic tetrahydroquinoline structure (**3 a**) was also isolated from the reaction mixture, albeit in low yield. Following this, phenyl‐substituted Hantzsch ester (**HE2**, Scheme 2 A, right), which our group has found to be effective in related reductive coupling methodologies,[^7^, ^11^] was employed in the reaction system. In this case, the product distribution was notably reversed, with the tetrahydroquinoline structure (**3 a**) dominating under these conditions. The interlinked nature of these reaction products, coupled with the absence of the opposite diastereomer for each, suggested that potentially diastereodivergent pathways leading to the net‐reductive (**2 a**‐*trans*) and redox‐neutral products (**3 a** *‐cis*) were in operation.^[12]^

Having uncovered this unusual redox‐dependent switch in the product selectivity, we believed that establishing reliable condition‐controlled stereo‐ and redox‐divergence of a single imine starting material would be of synthetic value.[^13^, ^14^] To this end, detailed optimisation studies to selectively produce either **2 a** or **3 a** were conducted (Scheme 2 B; for full optimisation details see supporting information). In an effort to maximise the yield of the Giese‐type product (**2 a**), solvent effects were found to be key to increasing selectivity, and also in avoiding direct reduction of the imine to the corresponding uncyclised amine (Entries 3–5), with DMA providing the indane framework in good yield (76 %) and selectivity (5.1:1). Initially, in situ imine formation could not be achieved under these conditions. Fortunately, a sub‐stoichiometric acetic acid additive eliminated the need for imine pre‐formation, affording the desired product in similar yield and selectivity directly from the aldehyde and aniline precursors (Entry 6). Moving to a marginally lower‐intensity, lower‐temperature light source resulted in excellent yield and selectivity (Entry 7, *Conditions A*).^[15]^

Having identified that the 4‐substituent of the Hantzsch ester was a key factor in switching between the two products (Entry 2), with the substituted Hantzsch ester reductant favouring the redox‐neutral cyclisation product **3 a**, various Hantzsch ester derivatives were then investigated (Entries 8–12).^[16]^ These studies identified **HE6** (R=Et) as the optimal reductant, affording the fused tetrahydroquinoline product in 68 % yield. Furthermore, addition of an acid co‐catalyst allowed for in situ imine formation (Entry 13), and switching to the lower‐intensity, lower‐temperature light source resulted in a 99 % yield, with excellent selectivity (>20:1, Entry 14, *Conditions B*).

**Scheme 3:**
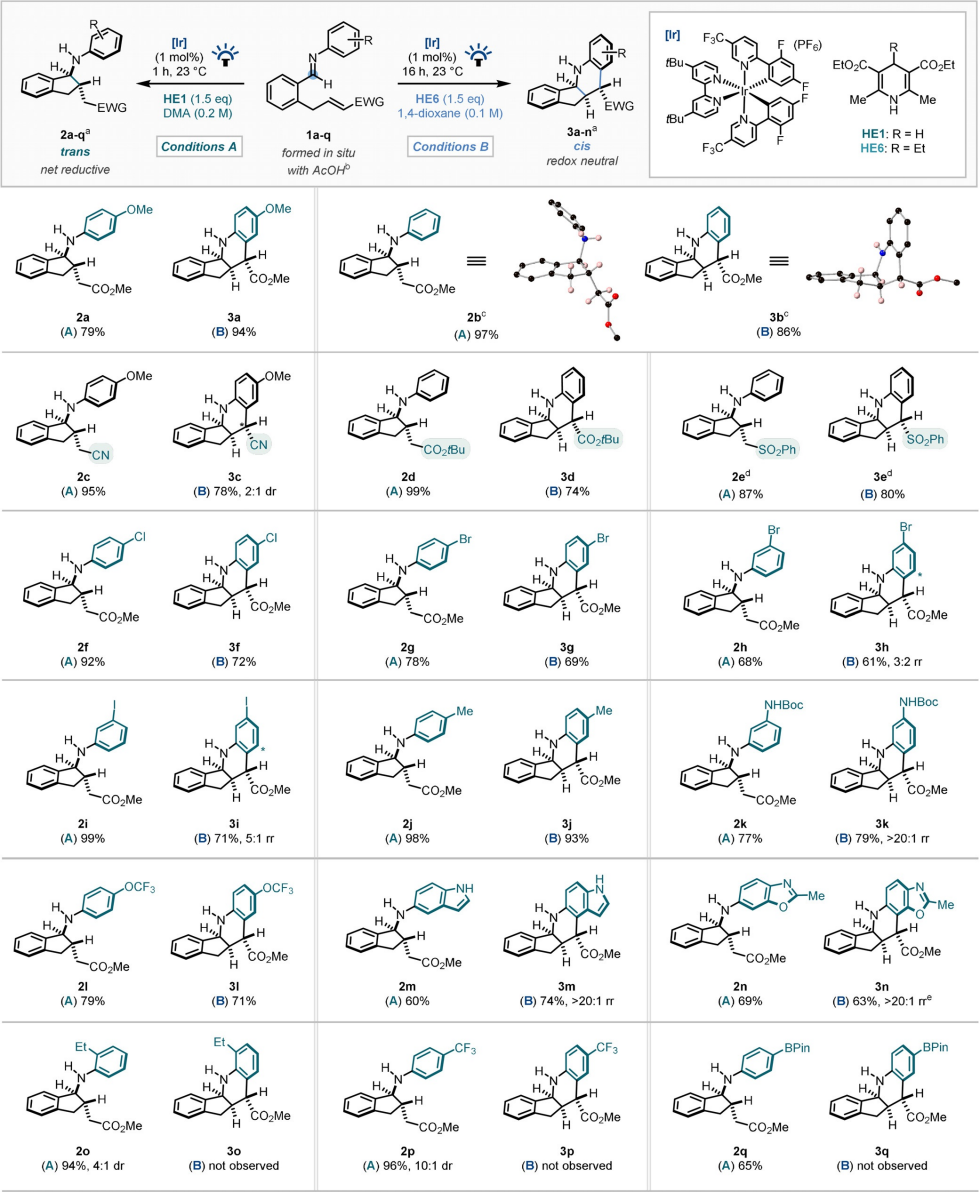
Scope of the diastereodivergent carbocyclisation. a) Product d.r. is >20:1 unless stated otherwise. In most cases, the majority of the remaining yield was identified as the product of the opposite diastereodivergent pathway; that is, 11 % (determined by ^1^H NMR analysis, see Scheme 2) of **3 a** was observed under *Conditions A* for the formation of **2 a**. Selectivity ratios based on ^1^H NMR analysis of the crude reaction mixture are given for each product in the SI. b) 20 mol % acetic acid added to facilitate in situ imine formation. c) Analysis by single crystal X‐ray diffraction; see SI (CIF) for details.^[17]^ Crystal structure of **2 b⋅HCl** shown. Aromatic CH, CH_3_ and chloride counterion (**2 b**) omitted for clarity. d) Temperature 29 °C (see SI). e) With 20 mol % **HE6** in DMA (see Scheme 4 A).

With optimal conditions now established for both pathways, the scope of the diastereodivergent carbocyclisation was explored (Scheme 3). Pleasingly, almost quantitative yield of the Giese‐type amino‐indane framework (**2 b**) was observed when unsubstituted aniline was employed in the reaction system.^[17]^ Notably, excellent selectivity for the mono‐cyclised product was observed consistently throughout the scope under *Conditions A*. When exchanging the electron‐withdrawing substituent on the alkene acceptor, α,β‐unsaturated nitrile (**2 c**), *tert*‐butyl ester (**2 d**), and alkenyl sulfone (**2 e**) analogues were all found to be amenable to the reaction conditions. An array of substituted aniline starting materials was then explored, with the reaction exhibiting tolerance to substitution in the *ortho*, *meta*, and *para* positions of the aniline fragment, including alkyl (**2 j**), trifluoromethoxy (**2 l**), various halogen substituents (**2 f**–**2 i**), and even a mono‐protected diaminoarene (**2 k**). Interestingly, amino‐indole and benzoxazole heteroaromatics were demonstrated to form the desired product in good yields (**2 m**–**2 n**). *Ortho*‐ethyl substituted aniline (**2 o**) and electron‐deficient anilines appended with trifluoromethyl (**2 p**) and pinacolboryl (**2 q**) substituents were also tolerated using *Conditions A*.

Our attention then turned to probing the scope of the dual cyclisation protocol leading to the *cis*‐fused tetrahydroquinoline products. Whilst an intermolecular reverse‐polarity Povarov reaction, which is thought to operate by a similar mechanism, has previously been reported,^[8]^ that chemistry could not be extended to α,β‐unsaturated esters, and therefore examining the utility of other Michael acceptors with *Conditions B* was of interest. Pleasingly, α,β‐unsaturated nitrile (**3 c**), *tert*‐butyl ester (**3 d**), and alkenyl sulfone (**3 e**) acceptors were all effective substrates in this methodology. The reaction was amenable to electronic variation on the aniline ring, with alkyl (**3 j**), trifluoromethoxy (**3 l**), protected amine (**3 k**), and various halogen substituents (**3 f**–**i**) tolerated. Notably, the amino‐indole (**3 m**) and benzoxazole (**3 n**) heteroaromatics were demonstrated to form the desired products in good yield, constructing complex fused pentacyclic architectures from simple starting materials, with absolute selectivity for the C4/C7 position of the benzenoid ring of the indole/benzoxazole heteroaromatics, respectively. Unsurprisingly, electron‐deficient^[18]^ and sterically demanding anilines failed to undergo the second cyclisation.

**Scheme 4:**
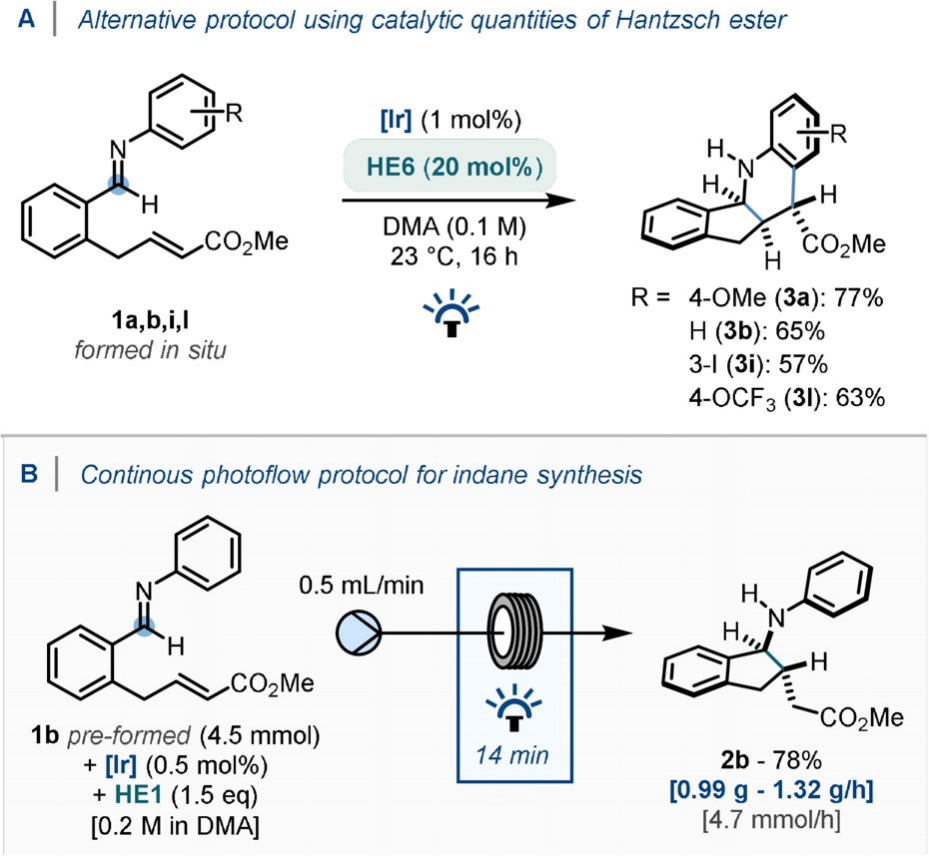
A) Use of sub‐stoichiometric quantities of reductant in the cascade cyclisation. B) Continuous photoflow adaptation.

Notably, most of the reactions exhibiting selectivity for the product of the redox‐neutral pathway (**3**) did not consume 1 equiv. of the Hantzsch ester (**HE6**), suggesting that sub‐stoichiometric quantities of the Hantzsch ester may still be able to lead to full conversion to the tetrahydroquinoline product. With this in mind, the reaction system was studied using 20 mol % of **HE6** (Scheme 4 A) on a subset of alkene‐tethered imine starting materials. Although generally in reduced yields compared with *Conditions B*, the desired products were still obtained in all cases, highlighting an alternative method with potential for scale‐up compatibility.

Furthermore, to demonstrate gram‐scale preparation of amino‐indane structure **2 b**, a continuous‐photoflow system was devised (Scheme 4 B). Using an inexpensive 3D‐printed insert for the photoreactor,^[19]^ a pre‐mixed solution of **1 b** and **HE1** in DMA [0.2 M] was shown, after a 45 minute run and 14 minute residence time, to deliver 0.99 g of **2 b** in a 78 % yield.

**Scheme 5:**
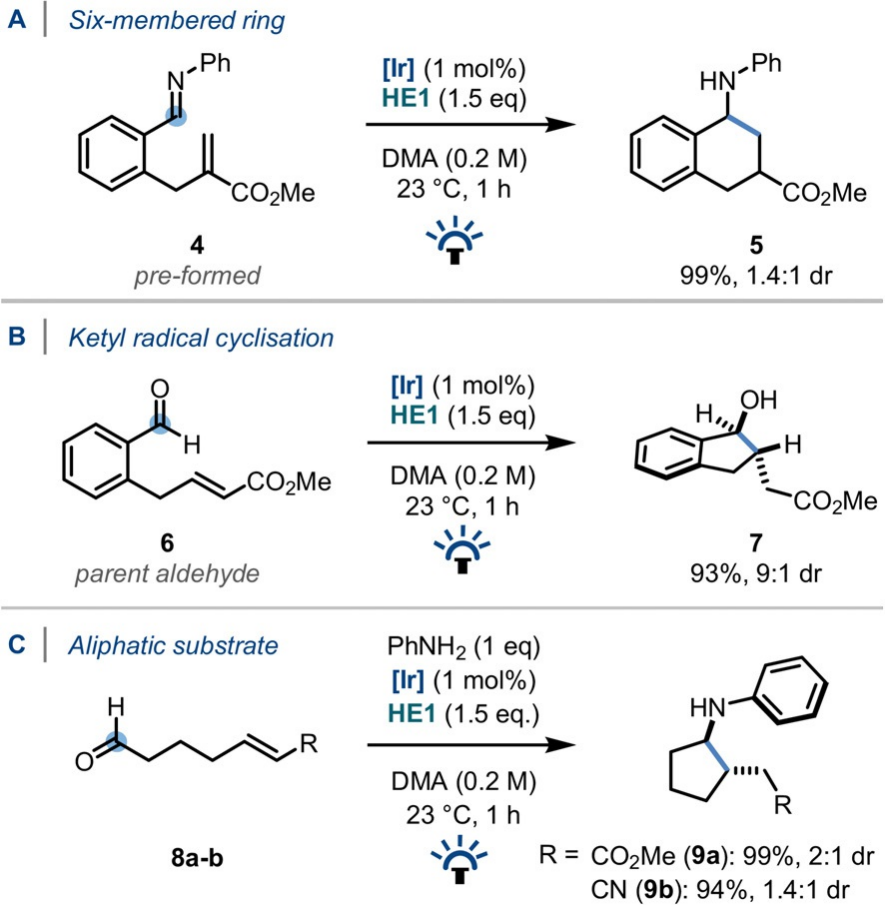
Extended scope of the net‐reductive pathway.

Following this, the homologous six‐membered carbocycle was prepared quantitatively from the analogous precursor (**4**, Scheme 5 A) using *Conditions A*. Notably, such 1‐aminotetrahydronaphthalene ring systems form the backbone of several selective seratonin reuptake inhibitor (SSRI) drugs, including the anti‐depressant Sertraline.^[20]^

Secondly, the precursor aldehyde **6** was subjected to the reaction conditions without the addition of the aniline, thereby forming the nucleophilic ketyl radical, which subsequently cyclised to form the substituted indanol (**7**) in excellent yield (Scheme 5 B), with a 9:1 dr in preference of the *trans* diastereomer.

Finally, substrates with linear aliphatic backbones (**8 a** & **8 b**) were successfully cyclised under photocatalytic *Conditions A*, affording the corresponding aminocyclopentanes (**9 a** & **9 b**) in excellent yield. Interestingly, however, under *Conditions B* or any variant thereof, no evidence of an operational dual cyclisation pathway was detected and only diastereomeric products of a single cyclisation pathway were isolated.^[21]^

**Scheme 6:**
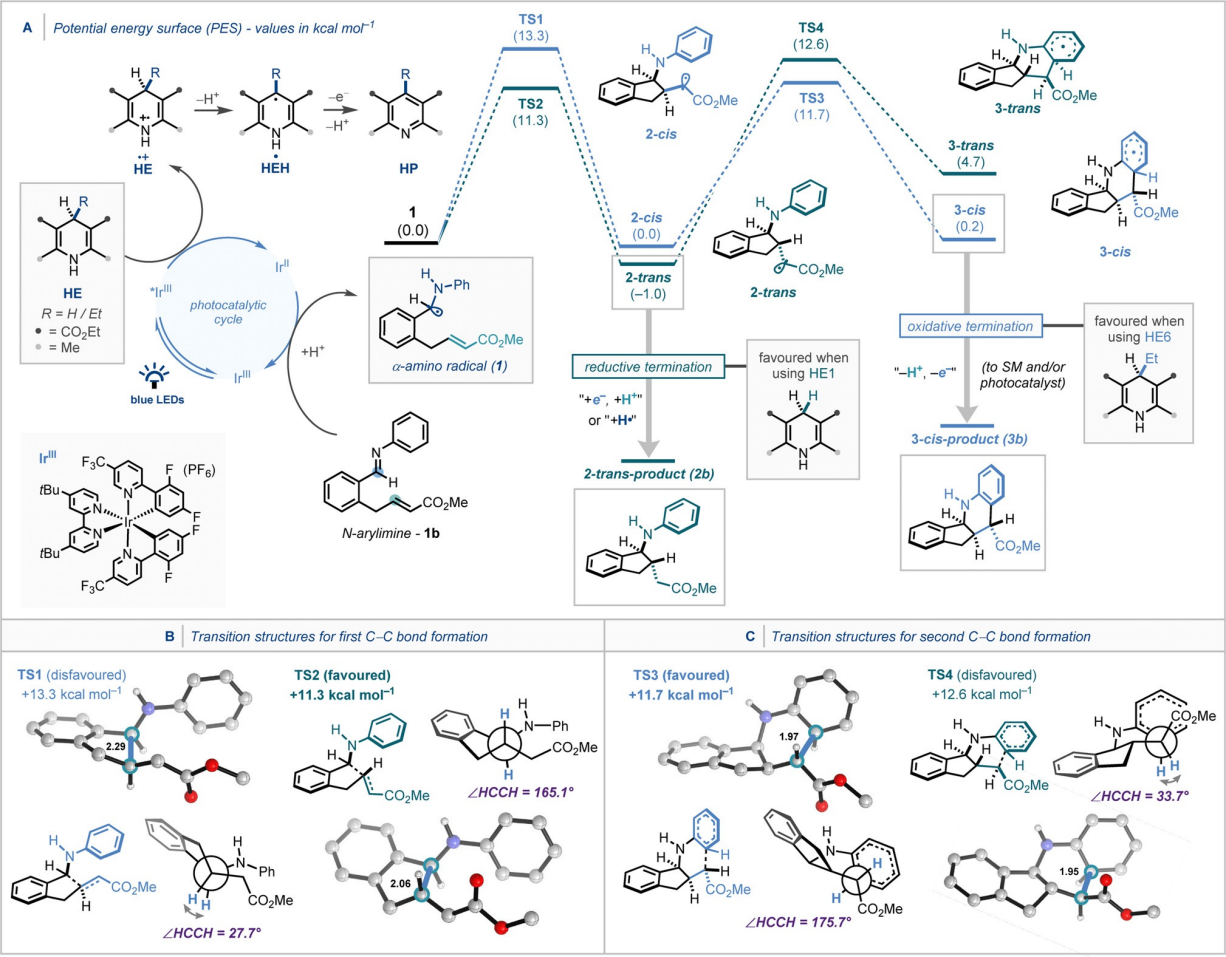
A) Computed potential energy surface (Δ*G* [kcal mol^−1^]) for the reagent‐controlled diastereodivergent photocatalytic cyclisation. B,C) The key C−C bond forming transition structures. All calculations computed at COSMO(DMF)‐ZORA‐(U)M06‐2X/TZ2P//COSMO(DMF)‐ZORA‐(U)BLYP‐D3(BJ)/TZ2P level of theory. Bond lengths [Å] and dihedral angles [°] of the TS geometries are provided in the insert.

From a mechanistic standpoint, and in order to elucidate the origin of the reagent‐controlled diastereodivergent photocatalytic cyclisation, in‐depth DFT (density functional theory) analysis (see Supporting Information for full computational details) was performed (Scheme 6). The α‐amino radical **1**, generated by the well‐established iridium photocatalyst mediated proton‐coupled electron transfer (PCET) of imine **1 b**,[^1a^, ^5a^, ^7^, ^8^] undergoes the first cyclisation through Giese addition to the tethered α,β‐unsaturated ester (Scheme 6 A).[^5c^, ^22^] Studies on the first cyclisation revealed that the formation of *trans* intermediate **2‐*trans*** through the transition structure (TS) **TS2** is kinetically favoured in comparison to **TS1** that forms **2‐*cis***. Following this, the second cyclisation for the *cis* isomer from **2‐*cis*** proceeds through **TS3** with a lower energy barrier than **TS1**, whereas the second cyclisation for the *trans* isomer from **2‐*trans*** proceeds through **TS4** with a higher energy barrier than **TS2** to form the energetically unfavourable intermediate **3‐*trans***.

In each case, the preferred TS (**TS2** and **TS3**) adopts a staggered conformation with a dihedral angle (∡HCCH) around the newly forming C−C bond of nearly 180° (Scheme 6 B and C). Taken altogether, our computational investigations suggest that product **2 b** results from facile termination of **2‐*trans*** by a Hantzsch ester‐derived species, and product **3 b** is formed from doubly cyclised **3‐*cis*** as a result of milder reducing conditions.[^8^, ^23^] Deuterium incorporation studies revealed that **2‐*trans*** could reasonably be reductively quenched via HAT, or by sequential electron transfer/proton transfer (ET/PT) from an oxidised Hantzsch species, with both pathways competing (see Supporting Information). However, this finding does not explain the origin of the different reaction outcomes when employing substituted vs. unsubstituted Hantzsch esters. Indeed, bond dissociation energy calculations reveal that oxidised forms of both **HE1** and **HE6** can feasibly act as HAT donors, and both can also feasibly reduce **2‐*trans*** by an ET/PT pathway (see Supporting Information for details). Instead, a possible explanation lies in the difference in oxidation potentials between **HE1** and **HE6** (+0.97 V and +1.10 V vs. SCE respectively; see Supporting Information for electrochemical measurements). With the unsubstituted Hantzsch ester being more readily oxidised, there is likely a higher concentration of oxidised Hantzsch species, capable of terminating the intermediate radical **2‐*trans***. This concurs with the observed difference in reaction rates between *Conditions A* and *B*, with the former being around five times faster in time‐course studies.^[24]^

To further validate the hypothesis that a higher concentration of active HAT agent favours termination of **2‐*trans***, and therefore formation of addition product (**2 b**) over the tetrahydroquinoline product (**3 b**), doping experiments with 1,4‐cyclohexadiene (1,4‐CHD, a commonplace hydrogen atom transfer donor) were conducted (Table 1).

**Table 1:**
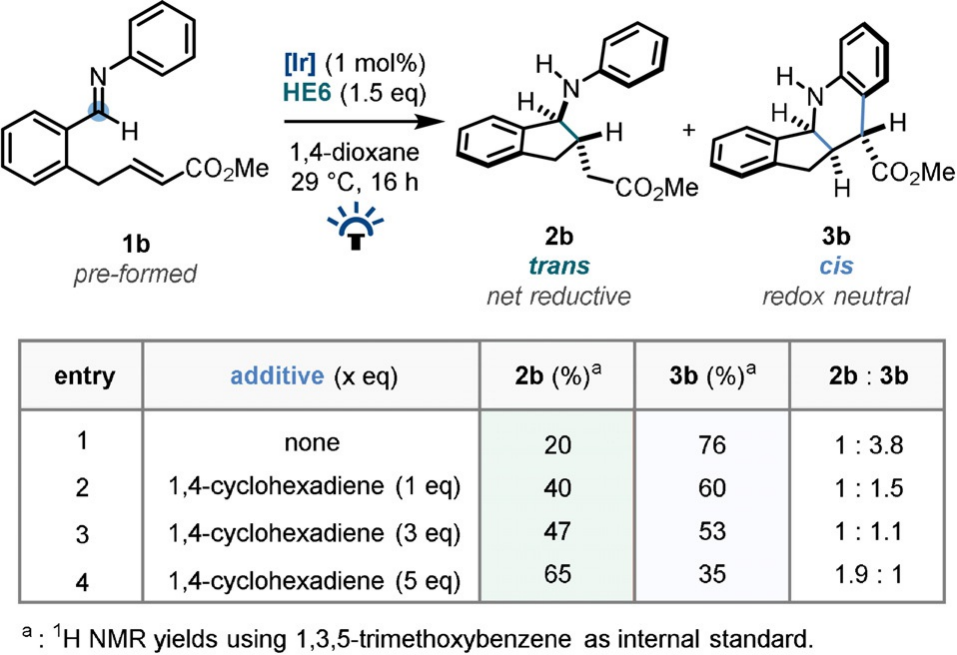
Termination mechanistic investigations.

Starting with a reaction system that afforded significant quantities of both products (**2 b**:**3 b**=1:3.8, Table 1 entry 1), the addition of 1 equivalent of 1,4‐CHD caused a distinct shift towards the single‐cyclisation product (**2 b**:**3 b**=1:1.5, Table 1 entry 2). Addition of a further 2 equivalents of the HAT donor increased this further, and addition of 5 equivalents was sufficient to cause the single‐cyclisation product to dominate (**2 b**:**3 b**=1.9:1, Table 1 entry 4). These experimental results are consistent with the existence of an HAT termination leading to the generation of **2 b**, which can be switchably controlled to allow either product of the diastereodivergent pathways to be selectively obtained.

## Conclusion

A switchable photocatalytic diastereodivergent carbocyclisation of imine‐derived α‐amino radicals has been developed. Two sets of reaction conditions, wherein both the stereochemical and reactivity outcomes are predictably determined by the judicious choice of Hantzsch ester and solvent, lead selectively to either a Giese‐type bicyclic amino‐indane structure or a fused tetracyclic tetrahydroquinoline architecture. The robust strategy for single cyclisation was also shown to be applicable to larger ring systems, ketyl radicals, unbiased aliphatic substrates, and to a continuous flow regime. DFT analysis, deuterium incorporation studies, electrochemical measurements, and HAT‐agent doping experiments aided in rationalising the diastereodivergent reaction pathways responsible for the reagent‐controlled switchability of the product outcome. Work is currently ongoing to uncover and develop further cascade cyclisations for the synthesis of complex amine frameworks.

## Conflict of interest

The authors declare no conflict of interest.

## Supporting information

As a service to our authors and readers, this journal provides supporting information supplied by the authors. Such materials are peer reviewed and may be re‐organized for online delivery, but are not copy‐edited or typeset. Technical support issues arising from supporting information (other than missing files) should be addressed to the authors.

Supporting Information
