# Supplementary material for: Switchable, Reagent‐Controlled Diastereodivergent Photocatalytic Carbocyclisation of Imine‐Derived α‐Amino Radicals
Source: Angew Chem Int Ed Engl. 2021 Oct 13;60(45):24116–23. doi: 10.1002/anie.202107253 (PMC8597041; doi:10.1002/anie.202107253)

Angewandte Chemie
Eine Zeitschrift der Gesellschaft Deutscher Chemiker

Supporting Information

Switchable, Reagent-Controlled Diastereodivergent Photocatalytic Carbocyclisation of Imine-Derived α-Amino Radicals

J. Andrew P. Maitland, Jamie A. Leitch, Ken Yamazaki, Kirsten E. Christensen, Doyle J. Cassar, Trevor A. Hamlin, and Darren J. Dixon**

anie_202107253_sm_miscellaneous_information.pdf

Contents

1. General S1
2. Photoreactor Setup S2
3. Extended Optimisation S4
4. Reagent Synthesis S7
5. General Procedures S8
6. Synthetic Procedures S10
7. Mechanistic Investigations S53
8. Photoflow Scale-Up S62
9. Computational Studies S63

References S94

NMR Spectra S95

1. General

Proton and carbon NMR spectra were recorded on Bruker AVIIIHD 400 nanobay, Bruker AVII 500 with ^{13}C cryoprobe, Bruker AVIII 600 with Prodigy N_2 broadband cryoprobe, or Bruker AVIII 700 with inverse TCI $^{1}H/^{13}C/^{15}N$ cryoprobe instruments, as indicated by the reported frequency for each recorded spectrum (^{1}H spectra: 400, 500, 600 or 700 MHz respectively; ^{13}C spectra: 101, 126, 151 or 176 MHz respectively). Fluorine NMR spectra were recorded on a Bruker AVIIIHD 400 nanobay instrument (377 MHz). Chemical shifts for protons are reported in parts per million downfield from $Si(CH_3)_4$ and are referenced to residual protium in the deuterated solvent ($CHCl_3$ at 7.26 ppm). Chemical shifts for fluorine atoms are reported in parts per million downfield from $CFCl_3$. NMR data are presented in the following format: chemical shift (multiplicity [app = apparent, br = broad, d = doublet, t = triplet, q = quartet, dd = doublet of doublets, dt = doublet of triplets, dq = doublet of quartets, ddd = doublet of doublet of doublets, dddd = doublet of doublet of doublet of doublets, m = multiplet], coupling constant [in Hz], number of equivalent nuclei by integration, assignment).

High-resolution mass spectra (ESI) were recorded on a Bruker µTOF mass spectrometer. Infrared spectra were recorded on a Bruker Tensor 27 FT-IR spectrometer as a thin film. Only selected maximum absorbances are reported (in $ν_{max}$ (cm^{-1})). Melting points were obtained on a Leica Galen III Hot-stage melting point apparatus and microscope and are reported uncorrected. Analytical thin-layer chromatography (TLC) was performed on Merck silica gel 60 F_{254} plates and visualised with UV light (254 nm), and with a $KMnO_4$ solution. Silica gel column chromatography was performed using 60 Å silica gel 40-63 µm purchased from VWR. Solvents used for chromatography were of technical quality and appropriate solvent mixtures are reported individually.

All reactions were performed using reagents obtained from Sigma-Aldrich, Acros Organics, Alfa Aesar, STREM or Fluorochem without further purification unless stated. All water used was purified through a Merck Millipore reverse osmosis purification system prior to use. Anhydrous solvents were used as supplied from Acros Organics (99.7+%, Extra Dry over Molecular Sieves, AcroSeal®). Reactions were performed under inert atmosphere unless otherwise stated. Solvents were removed under reduced pressure using Büchi Rotavapor apparatus.

2. Photoreactor Setup

i. Batch Reactions

Due to experimental work being carried out in two laboratories, two photoreactor setups were used in the course of this work. Each procedure listed herein details which setup was used, and the observed differences between the setups are discussed in the context of the optimisation. Both setups were equipped with an integral cooling fan, thereby maintaining a reaction temperature of 23-30 °C – temperature dependence is further discussed in the context of the mechanistic investigations (Section 7). Both photoreactors were placed atop a stirrer hotplate in order to facilitate reaction agitation.

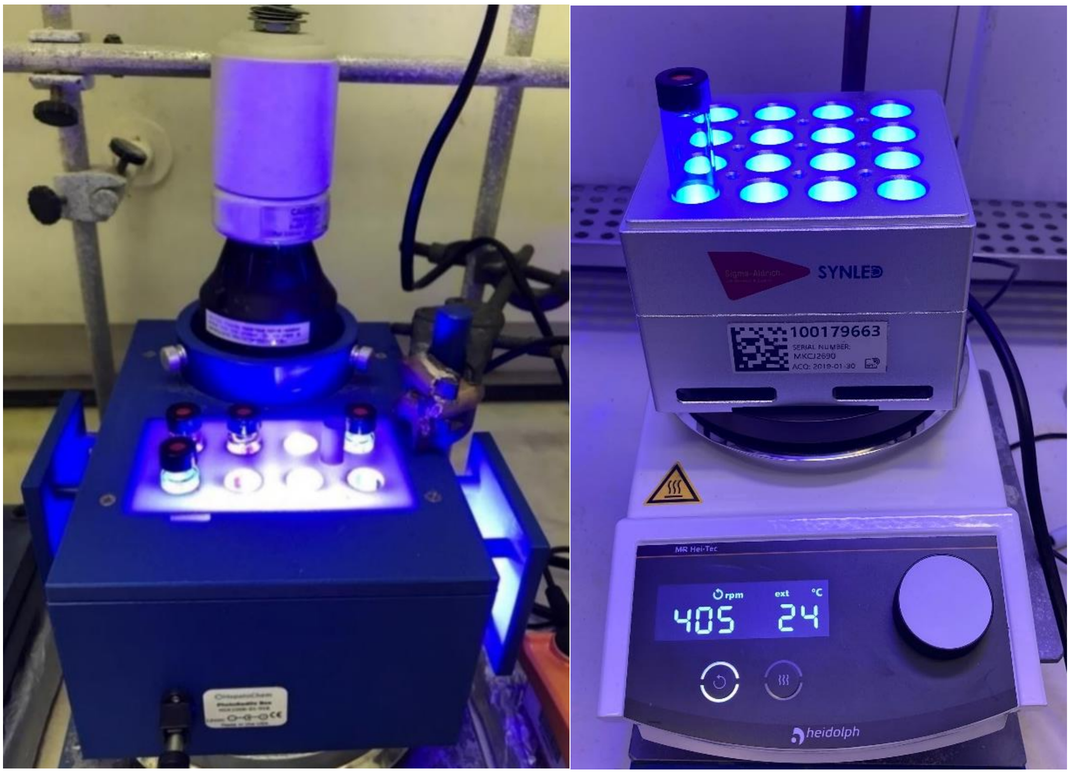

Setup 1 (Left): HepatoChem EvoluChem™ PhotoRedOx Box (Part No. HCK1006-01-016) equipped with an EvoluChem™ 450PF 450 nm 18 W blue LED light source (Part No. HCK1012-01-002). **Setup 2 (Right)**: Sigma Aldrich SynLED (Part No. Z742680) 16-well block with 465-470 nm 12 W bottom-lit blue LED light source.

ii. Flow Reactions

A custom 3D-printed tubing holder was designed for the EvoluChem™ PhotoRedOx Box, matching the external dimensions of the EvoluChem™ 8x2 mL vial holder (Part No. HCK1006-01-018). The insert was designed to support two coils of 1/16 inch outer diameter tubing, with a total exposed tubing length of 4.8 m. The design can be accessed at https://www.thingiverse.com/thing:4850887.

A 5.5 m length of 1/16 inch outer diameter, 1/32 inch inner diameter FEP tubing (Cole-Palmer Part No. 06406-60) was coiled around the custom-printed insert. As with Batch Setup 1, the insert was placed into a HepatoChem EvoluChem™ PhotoRedOx Box (Part No. HCK1006-01-016) equipped with an EvoluChem™ 450PF 450 nm 18 W blue LED light source (Part No. HCK1012-01-002).

The reactor was first purged with blank reaction solvent (in order to prevent cascading or siphoning), followed by the reaction mixture. Flow through the photoflow reactor was controlled using an HP Agilent 1050 HPLC pump, and the irradiated reaction mixture was collected in a round-bottom flask before being purified as detailed individually (Photoflow Scale-Up).

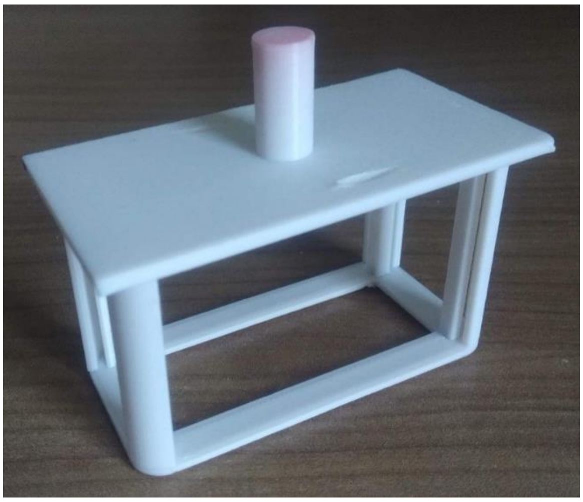

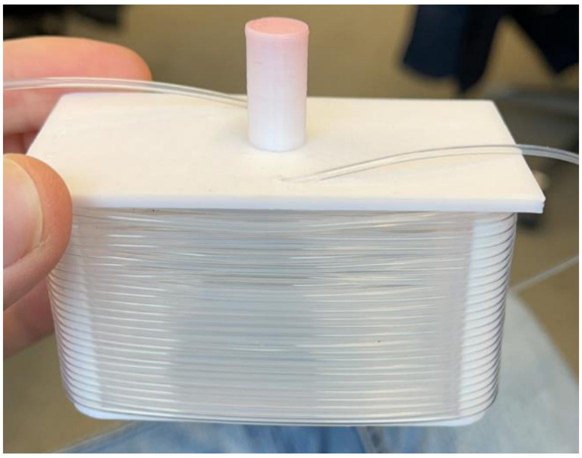

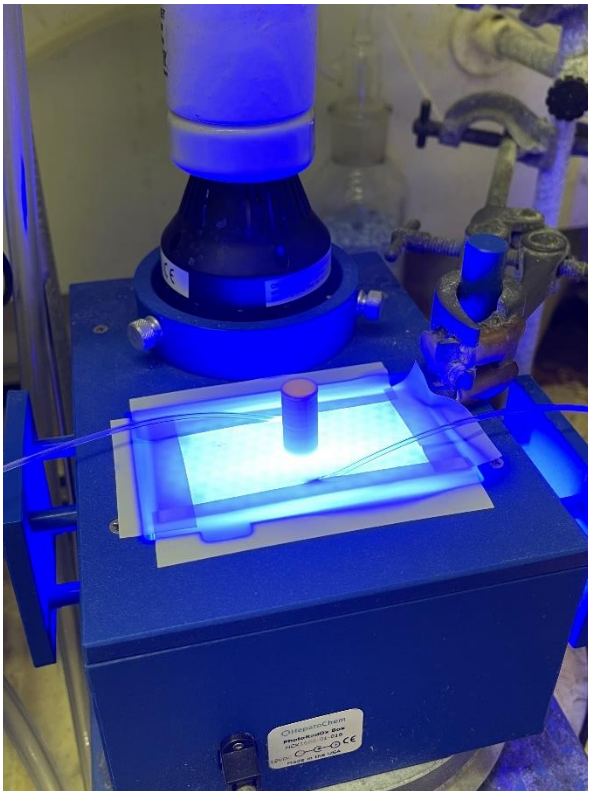

3. Extended Optimisation

Table S1: Extended optimisation

Entry[a]	LED[b]	Photocat[c]	Solvent	c / M	HE (R =)[c]	2a[d]	3a[d]	Total	Selectivity[e]	Comment
1	1	[Ir]	DMSO	0.1	HE1 (H)	38	6	44	6.3:1	
2	1	[Ir]	DMSO	0.1	HE2 (Ph)	11	45	56	4.1:1	
3	1	[Ir]	DMSO	0.2	HE1 (H)	57	6	63	9.5:1	
4	1	[Ir]	CH_2Cl_2	0.2	HE1 (H)	60	30	90	2:1	
5	1	[Ir]	DMF	0.1	HE1 (H)	57	14	71	4.1:1	
6	1	[Ir]	DMA	0.1	HE1 (H)	76	15	91	5.1:1	
7	1	[Ir]	DMA	0.2	HE1 (H)	84	3	87	>20:1	
8	1	[Ir]	CH_2Cl_2	0.1	HE2 (Ph)	7	52	59	7.4:1	
9	1	[Ir]	CH_2Cl_2	0.1	HE3 (Bn)	19	35	54	1.8:1	
10	1	[Ir]	CH_2Cl_2	0.1	HE4 (*i*Pr)	11	45	56	4.1:1	
11	1	[Ir]	CH_2Cl_2	0.1	HE5 (Cy)	6	43	49	7.2:1	
12	1	[Ir]	CH_2Cl_2	0.1	HE6 (Et)	13	68	81	5.2:1	
13	1	[Ir]	CH_2Cl_2	0.1	HE7 (Me)	7	66	73	9.4:1	
14	1	[Ir]	CH_2Cl_2	0.1	HE8 (*i*Pent)	28	41	69	1.5:1	
15	1	[Ir2]	CH_2Cl_2	0.1	HE7 (Me)	2	23	25	11.5:1	63% SM
16	1	[Ir3]	CH_2Cl_2	0.1	HE7 (Me)	0	0	0	-	100% SM
17	1	[Ir4]	CH_2Cl_2	0.1	HE7 (Me)	2	18	20	9:1	73% SM
18	1	[Ir5]	CH_2Cl_2	0.1	HE7 (Me)	6	66	72	11:1	
19	1	[Ru1]	CH_2Cl_2	0.1	HE7 (Me)	0	0	0	-	100% SM
20	1	[Ru2]	CH_2Cl_2	0.1	HE7 (Me)	0	0	0	-	100% SM
21	1	4-CzIPN	CH_2Cl_2	0.1	HE7 (Me)	3	17	20	5.7:1	70% SM
22	1	[Ir]	DMA	0.2	HE1 (H)	0	0	0	-	Control - No Light
23	1	[Ir]	DMA	0.2	HE1 (H)	82	6	88	13.7:1	Control - No Photocat[f]
24	1	[Ir]	DMA	0.2	HE1 (H)	0	0	0	-	Control - No Hantzsch
25	1	[Ir]	CH_2Cl_2	0.1	HE6 (Et)	0	0	0	-	Control - No Light
26	1	[Ir]	CH_2Cl_2	0.1	HE6 (Et)	0	0	0	-	Control - No Photocat
27	1	[Ir]	CH_2Cl_2	0.1	HE6 (Et)	0	0	0	-	Control - No Hantzsch
28	2	[Ir]	CH_2Cl_2	0.1	HE6 (Et)	0	0	0	-	
29[a]	2	[Ir]	CH_2Cl_2	0.1	HE6 (Et)	0	0	0	-	See footnote[g]
30	K	[Ir]	CH_2Cl_2	0.1	HE6 (Et)	20	14	34	1.4:1	
31	2	[Ir]	DME	0.1	HE6 (Et)	14	69	83	4.9:1	
32	2	[Ir]	THF	0.1	HE6 (Et)	12	68	80	5.7:1	
33	2	[Ir]	EtOAc	0.1	HE6 (Et)	8	58	66	7.3:1	
**34**	**2**	**[Ir]**	**1,4-Dioxane**	**0.1**	**HE6 (Et)**	**1**	**99**	**100**	**>20:1**	**Optimal conditions for double cyclisation**
35	2	[Ir]	Toluene	0.1	HE6 (Et)	0	0	0	-	
36	2	[Ir]	1,2-DCE	0.1	HE6 (Et)	0	0	0	-	
37	2	[Ir]	DMA	0.1	HE6 (Et)	40	38	78	1.1:1	
38[h]	2	[Ir]	DMA	0.2	HE6 (Et)	10	79	89	7.9:1	Catalytic in Hantzsch ester
**39[i]**	**2**	**[Ir]**	**DMA**	**0.2**	**HE1 (H)**	**89**	**11**	**100**	**8.1:1**	**Optimal conditions for single cyclisation**
40[j]	1	[Ir]	DMA	0.2	HE1 (H)	63	17	80	3.7:1	Acid additive poorly tolerated with higher-
41	1	[Ir]	1,4-Dioxane	0.1	HE6 (Et)	11	63	74	5.7:1	intensity higher-temp light source[g]

a: Reaction conditions – 1.5 eq. Hantzsch ester unless stated otherwise, 16 hours unless stated otherwise, 1 mol% photocatalyst, entries 29 and 31-41 include a 20 mol% acetic acid additive to enable *in situ* imine formation.

b: Light sources – 1 = HepatoChem PhotoRedOx Box equipped with EvoluChem 450PF 450 nm 18 W LED (Setup 1); 2 = Sigma Aldrich SynLED 16-well bottom-lit 465-470 nm 12 W LED (Setup 2); K = Kessil lamp.

c: See overleaf for Key.

d: ^{1}H NMR Yield using 1,3,5-trimethoxybenzene as an internal standard.

e: Selectivity ratio for major:minor product (where major is taken to be the desired product).

f: The control experiment using no photocatalyst resulted in significant conversion to the desired product. Whilst a small substrate scope revealed a similar reactivity profile to the optimised reaction, significantly lower yields were observed. Given that the reaction does not proceed in the absence of light, it was concluded that either an electron donor-acceptor (EDA) complex was forming between the Hantzsch ester and the substrate, or that the Hantzsch ester was directly photoexcited

by the light source.[1] UV-Vis experiments were undertaken in order to examine the potential presence of an EDA complex, but none was observed. Furthermore, this finding was only observed when using the unsubstituted Hantzsch ester (**HE1**).

g: The observed differences between the three light sources are discussed in Section 7, in the context of the reaction mechanism. While various factors may be at play, further investigations found reaction temperature to be significant, with the SynLED setup being significantly (~6 °C) lower in temperature than the PhotoRedOx Box.

h: 20 mol% Hantzsch ester employed.

i: Reaction time = 1 hour.

Key

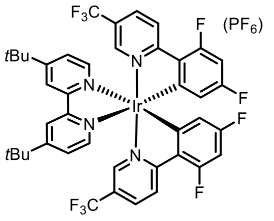

[Ir(dF(CF$_3$)ppy)$_2$(dtbbpy)]PF$_6$ [Ir]

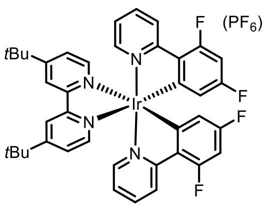

[Ir(dFppy)$_2$(dtbbpy)]PF$_6$ [Ir2]

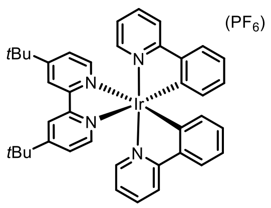

[Ir(ppy)$_2$(dtbbpy)]PF$_6$ [Ir3]

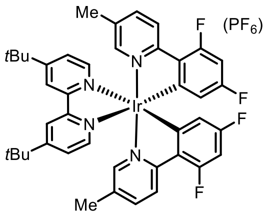

[Ir(dFMeppy)$_2$(dtbbpy)]PF$_6$ [Ir4]

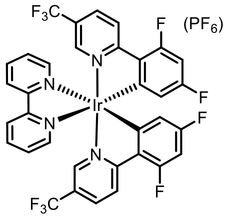

[Ir(dF(CF$_3$)ppy)$_2$(bpy)]PF$_6$ [Ir5]

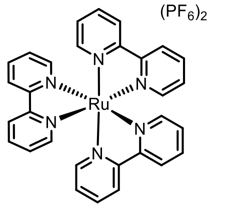

[Ru(bpy)$_3$](PF$_6$)$_2$ [Ru1]

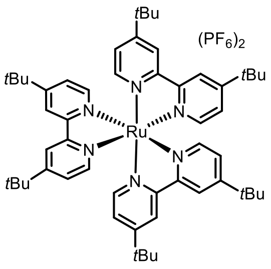

[Ru(dtbbpy)$_3$](PF$_6$)$_2$ [Ru2]

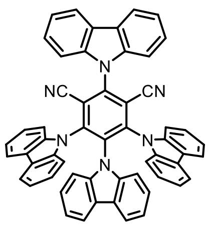

4-CzIPN

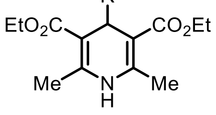

R	HE
H	**HE1**
Ph	**HE2**
Bn	**HE3**
*i*Pr	**HE4**
Cy	**HE5**
Et	**HE6**
Me	**HE7**
*i*Pent	**HE8**

4. Reagent Synthesis

i. Photocatalysts

[Ir] – (Ir[dF(CF_3)ppy)$_2$](dtbpy))PF_6 was prepared as previously reported.[2] All other photocatalysts were used as supplied by Strem Chemicals or Sigma Aldrich.

ii. Hantzsch esters

HE1 (R = H) was used as supplied by Fluorochem. **HE2** (R = Ph),[2] **HE3** (R = Bn),[3] **HE4** (R = *i*Pr),[2] **HE5** (R = Cy),[2] **HE6** (R = Et),[4] **HE7** (R = Me)[4] and **HE8** (R = *i*Pent)[5] were synthesised according to literature procedures.

1-d$_1$-HE1[6] (85% d-incorporation), **4,4-d$_2$-HE1**[7] (>99% d-incorporation) and **1,4,4-d$_3$-HE1**[8] (82% d-incorporation at 1-position, >99% d-incorporation at 4-position) were synthesised according to literature procedures.

Synthesis of **4-d$_1$-HE6**

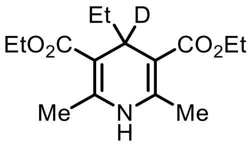

Propanal-d$_1$ was prepared according to a literature method, achieving 95% d-incorporation.[9]

Propanal-d$_1$ (3.6 mL, 50 mmol), ethyl 3-aminocrotonate (6.3 mL, 50 mmol), ethyl acetoacetate (6.4 mL) and TBAHS (2 g, 6 mmol, 12 mol%) were combined in ethylene glycol (20 mL) and heated to 100 °C for 3 h. A yellow precipitate was seen to form immediately. The reaction mixture was poured into brine (100 mL), extracted with ethyl acetate (2x 100 mL) and the combined organic layers were dried over magnesium sulfate and concentrated. The crude yellow solid was recrystallised three times from hot ethanol to afford light-yellow crystals of **4-d$_1$-HE6** (6.2 g, 44%), with 95% d-incorporation as determined by integration of the remaining proton peak in ^{1}H NMR.

^{1}H NMR (400 MHz, DMSO-d$_6$) δ = 8.57 (s, 1H), 4.17 – 3.97 (m, 4H), 3.76 (t, 1H, 5%), 2.20 (s, 6H), 1.28 – 1.12 (m, 8H), 0.65 (t, J=7.5, 3H); **^{13}C NMR** (101 MHz, DMSO-d$_6$) δ = 167.3, 146.1, 100.3, 58.7, 33.0 (m), 28.9, 18.1, 14.3, 8.9; **^{2}H NMR** (92 MHz, H-DMSO) δ = 8.26 (s, 1H), 3.72 (s, 1H); **FT-IR** (thin film) ν_{max} (cm^{-1}) 3337 (m), 2960 (w), 2361 (w), 1693 (s), 1676 (s), 1654 (s), 1484 (s); **HRMS** (ESI) *m/z* calcd for $C_{15}H_{22}DNO_4+H^+$: 283.1763 [*M*+H]$^+$; found: 283.1763; **m.p.** (from EtOH) 106-108 °C.

5. General Procedures

General Procedure A for Reaction Optimisation with EvoluChem™ 450PF LED

Imine **1a** was pre-formed by stirring aldehyde **6** (20.4 mg, 0.1 mmol) and 4-anisidine (12.3 mg, 0.1 mmol) with $MgSO_4$ (50 mg) in anhydrous CH_2Cl_2 (0.5 mL, 0.2 mol L^{-1}) for 16 hours. The mixture was filtered and transferred to a 1.6 mL glass vial equipped with stir bar. The solvent was evaporated by passing a stream of N_2 across its surface for 5 minutes. Hantzsch ester (**HE1**-**HE8**, *x* mmol), photocatalyst (0.001 mmol, 1 mol%) and anhydrous degassed reaction solvent (*y* mol L^{-1}) were added to the vial, and N_2 was bubbled through the reaction mixture for 2 minutes. The vial was then sealed and suspended in a HepatoChem PhotoRedOx Box equipped with an EvoluChem™ 450PF 450 nm 18 W LED light source (Setup 1) and irradiated for 16 h. 1,3,5-Trimethoxybenzene (5.6 mg, 0.033 mmol) was then added to the reaction mixture, and an aliquot was taken for analysis by quantitative ^{1}H NMR spectroscopy.

General Procedure B for Reaction Optimisation with SynLED

To an 8 mL Wheaton vial equipped with stir bar and rubber septum were charged aldehyde **6** (40.8 mg, 0.2 mmol, 1 eq.), 4-anisidine (24.6 mg, 0.2 mmol, 1 eq.), substituted Hantzsch ester (*x* mmol) and $[Ir(dF(CF_3)ppy)_2(dtbbpy)]PF_6$ (2.2 mg, 0.001 mmol, 1 mol%). The solids were degassed by repeated evacuation and refilling with N_2 *via* the septum. Anhydrous degassed reaction solvent (*y* mol L^{-1}) and acetic acid (2.5 µL, 0.04 mmol, 20 mol%) were added *via* the septum, and the mixture was stirred 15 minutes in darkness (in order to facilitate imine formation). The vial was placed in a Sigma Aldrich SynLED 467.5 nm 12 W LED 16-well array (Setup 2) and irradiated for *t* h. 1,3,5-Trimethoxybenzene (11.2 mg, 0.067 mmol) was added to the reaction mixture, and an aliquot of the mixture was taken for analysis by quantitative ^{1}H NMR spectroscopy.

General Procedure C for Single Cyclisation (Optimised Conditions A)

To an 8 mL Wheaton vial equipped with stir bar and rubber septum were charged aldehyde (0.2 mmol, 1 eq.), aniline derivative (when solid, 0.2 mmol, 1 eq.), **HE1** (unsubstituted Hantzsch ester, 74 mg, 0.3 mmol, 1.5 eq.) and $[Ir(dF(CF_3)ppy)_2(dtbbpy)]PF_6$ (2.2 mg, 0.002 mmol, 1 mol%). The solids were degassed by repeated evacuation and refilling with N_2 *via* the septum. Aniline (when liquid, 0.2 mmol, 1 eq.), anhydrous degassed *N*,*N*-dimethylacetamide (1 mL, 0.2 mol L^{-1}) and acetic acid (2.5 µL, 0.04 mmol, 20 mol%) were added *via* the septum, and the mixture was stirred 15 minutes in darkness (in order to facilitate imine formation). The vial was placed in a Sigma Aldrich SynLED 467.5 nm 12 W LED 16-well array (Setup 2) and irradiated for 1 h. The reaction mixture was diluted with ethyl acetate (20 mL) and the organic phase was washed with saturated aqueous $NaHCO_3$ solution (2x 10 mL) and brine (2x 10 mL). The organic phase was dried over $MgSO_4$, filtered, and concentrated under reduced pressure. The crude material was purified by column chromatography (see individual experiments).

General Procedure D for Stoichiometric Double Cyclisation (Optimised Conditions B)

To an 8 mL Wheaton vial equipped with stir bar and rubber septum were charged aldehyde (0.2 mmol, 1 eq.), substituted aniline (when solid, 0.2 mmol, 1 eq.), **HE6** (ethyl-substituted Hantzsch ester, 84 mg, 0.3 mmol, 1.5 eq.) and $[Ir(dF(CF_3)ppy)_2(dtbbpy)]PF_6$ (2.2 mg, 0.002 mmol, 1 mol%). The solids were degassed by repeated evacuation and refilling with N_2 *via* the septum. Substituted aniline (when liquid,

0.2 mmol, 1 eq.), anhydrous degassed 1,4-dioxane (2 mL, 0.1 mol L^{-1}) and acetic acid (2.5 µL, 0.04 mmol, 20 mol%) were added *via* the septum, and the mixture was stirred 30 minutes in darkness (in order to facilitate imine formation). The vial was placed in a Sigma Aldrich SynLED 467.5 nm 12 W LED 16-well array (Setup 2) and irradiated for 16 h. The reaction mixture was concentrated under reduced pressure, then diluted with ethyl acetate (20 mL) and the organic phase was washed with saturated aqueous $NaHCO_3$ solution (10 mL) and brine (10 mL). The organic phase was dried over $MgSO_4$, filtered, and concentrated under reduced pressure. The crude material was purified by column chromatography (see individual experiments).

General Procedure E for Catalytic Double Cyclisation (Optimised Conditions C)

To an 8 mL Wheaton vial equipped with stir bar and rubber septum were charged aldehyde (0.2 mmol, 1 eq.), substituted aniline (when solid, 0.2 mmol, 1 eq.), **HE6** (ethyl-substituted Hantzsch ester, 11 mg, 0.04 mmol, 20 mol%) and $[Ir(dF(CF_3)ppy)_2(dtbbpy)]PF_6$ (2.2 mg, 0.002 mmol, 1 mol%). The solids were degassed by repeated evacuation and refilling with N_2 *via* the septum. Substituted aniline (when liquid, 0.2 mmol, 1 eq.), anhydrous degassed *N*,*N*-dimethylacetamide (2 mL, 0.1 mol L^{-1}) and acetic acid (2.5 µL, 0.04 mmol, 20 mol%) were added *via* the septum, and the mixture was stirred 30 minutes in darkness (in order to facilitate imine formation). The vial was placed in a Sigma Aldrich SynLED 467.5 nm 12 W LED 16-well array (Setup 2) and irradiated for 16 h. The reaction mixture was diluted with ethyl acetate (20 mL) and the organic phase was washed with saturated aqueous $NaHCO_3$ solution (2x 10 mL) and brine (3x 10 mL). The organic phase was dried over $MgSO_4$, filtered, and concentrated under reduced pressure. The crude material was purified by column chromatography (see individual experiments).

6. Synthetic Procedures

Synthesis of **2a** (1 mmol scale)

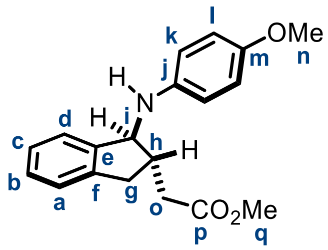

To an 8 mL Wheaton vial equipped with stir bar and rubber septum were charged aldehyde **6** (204 mg, 1 mmol, 1 eq.), 4-methoxyaniline (123 mg, 1 mmol, 1 eq.), **HE1** (unsubstituted Hantzsch ester, 296 mg, 1.2 mmol, 1.2 eq.) and [Ir(dF(CF_3)ppy)$_2$(dtbbpy)]PF_6 (5.5 mg, 0.005 mmol, 0.5 mol%). The solids were degassed by repeated evacuation and refilling with N_2 *via* the septum. Anhydrous degassed *N*,*N*-dimethylacetamide (5 mL, 0.2 mol L^{-1}) and acetic acid (12.5 µL, 0.2 mmol, 20 mol%) were added *via* the septum, and the mixture was stirred 15 minutes in darkness (in order to facilitate imine formation). The vial was placed in a Sigma Aldrich SynLED 467.5 nm 12 W LED 16-well array and irradiated for 2 h. The reaction mixture was diluted with ethyl acetate (100 mL) and the organic phase was washed with saturated aqueous $NaHCO_3$ solution (2x 50 mL) and brine (3x 20 mL). The organic phase was dried over $MgSO_4$, filtered, and concentrated under reduced pressure. ^{1}H NMR analysis of the crude reaction mixture revealed a ratio of singly-cyclised *trans* product **2a** to doubly-cyclised *cis*-fused product **3a** of 10.1:1. The crude material was purified by column chromatography (SiO_2: acetone/pentane, 5–15%), affording **2a** (267 mg, 86%) as a colourless oil.

^{1}H NMR (500 MHz, $CDCl_3$) δ 7.30 (d, *J* = 8.6 Hz, 3H, ***H*-a,c,d**), 7.20 – 7.15 (m, 1H, ***H*-b**), 6.86 – 6.79 (m, 2H, ***H*-k**), 6.75 – 6.68 (m, 2H, ***H*-l**), 4.67 (d, *J* = 7.4 Hz, 1H, ***H*-i**), 3.79 (s, 3H, ***H*-n**), 3.68 (s, 3H, ***H*-q**), 3.28 – 3.18 (m, 1H, ***H*-g'**), 2.85 – 2.76 (m, 1H, ***H*-o'**), 2.73 – 2.62 (m, 2H, ***H*-g'',h**), 2.56 (dd, *J* = 15.0, 8.1 Hz, 1H, ***H*-o''**).

^{13}C NMR (126 MHz, $CDCl_3$) δ 173.2 (***C*-p**), 152.2 (***C*-m**), 144.6 (***C*-e**), 142.2 (***C*-j**), 141.5 (***C*-f**), 127.8 (***C*-c**), 126.8 (***C*-b**), 124.9 (***C*-a**), 124.2 (***C*-d**), 115.0 (***C*-k**), 114.5 (***C*-l**), 64.4 (***C*-i**), 55.8 (***C*-n**), 51.7 (***C*-q**), 45.0 (***C*-h**), 37.8 (***C*-o**), 36.2 (***C*-g**).

FT-IR (thin film) ν_{max} (cm^{-1}) 3382, 2950, 1730 (s), 1509 (s).

HRMS (ESI) *m/z* calcd for $C_{19}H_{21}NO_3+H^+$: 312.1594 [*M*+H]$^+$; found: 312.1591.

Synthesis of 2b-*trans*

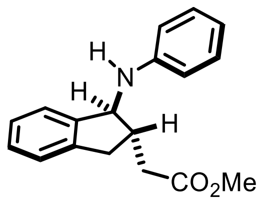

Compound **2b-*trans*** was synthesised according to General Procedure C using aldehyde **6** (40.8 mg, 0.2 mmol) and aniline (18 µL, 0.2 mmol). ^{1}H NMR analysis of the crude reaction mixture revealed a ratio of singly-cyclised *trans* product **2b** to doubly-cyclised *cis*-fused product **3b** of >20:1. Purification by column chromatography (SiO_2: acetone/pentane, 5–10%) afforded **2b-*trans*** (54 mg, 97%) as a colourless oil.

The stereochemical configuration of **2b-*trans*** was confirmed by single crystal x-ray diffraction analysis as its hydrochloride salt. **2b-*cis*** was separately prepared for comparison – see Section 7.

^{1}H NMR (400 MHz, $CDCl_3$) δ = 7.29 (dd, *J*=7.3, 1.1, 1H), 7.25 – 7.14 (m, 5H), 6.79 – 6.69 (m, 3H), 4.74 (d, *J*=7.0, 1H), 3.92 (br s, 1H), 3.65 (s, 3H), 3.27 – 3.17 (m, 1H), 2.79 (dd, *J*=14.9, 4.9, 1H), 2.73 – 2.62 (m, 2H), 2.56 (dd, *J*=14.9, 8.0, 1H).

^{13}C NMR (101 MHz, $CDCl_3$) δ = 173.2, 148.0, 144.4, 141.5, 129.4, 127.9, 126.8, 124.9, 124.2, 117.6, 113.0, 63.3, 51.6, 45.2, 37.7, 36.2.

FT-IR (thin film) ν_{max} (cm^{-1}) 3388, 2950, 1732, 1601, 1506.

HRMS (ESI) *m/z* calcd for $C_{18}H_{19}NO_2+H^+$: 282.1489 [*M*+H]$^+$; found: 282.1491.

Synthesis of **2c**

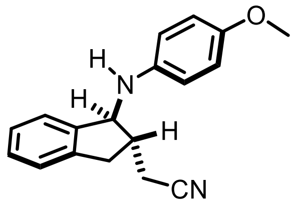

Compound **2c** was synthesised according to General Procedure C using aldehyde **S1** (34.2 mg, 0.2 mmol) and 4-methoxyaniline (25 mg, 0.2 mmol). ^{1}H NMR analysis of the crude reaction mixture revealed a ratio of singly-cyclised *trans* product **2c** to doubly-cyclised *cis*-fused product **3c** of >20:1. Purification by column chromatography (SiO_2: acetone/pentane, 5–10%) afforded **2c** (52 mg, 95%) as a colourless oil.

^{1}H NMR (400 MHz, $CDCl_3$) δ = 7.30 (dd, *J*=7.4, 1.2, 1H), 7.26 (d, *J*=1.2, 2H), 7.23 – 7.17 (m, 1H), 6.82 (d, *J*=8.8, 2H), 6.72 (d, *J*=8.9, 2H), 4.74 (d, *J*=6.8, 1H), 3.77 (s, 3H), 3.58 (br s, 1H), 3.25 (dd, *J*=15.7, 7.2, 1H), 2.82 (dd, *J*=15.7, 7.6, 1H), 2.76 – 2.52 (m, 3H).

^{13}C NMR (101 MHz, $CDCl_3$) δ = 152.6, 143.2, 141.2, 140.3, 128.2, 127.1, 124.9, 124.3, 118.3, 115.0, 114.7, 63.7, 55.6, 44.2, 35.3, 20.5.

FT-IR (thin film) ν_{max} (cm^{-1}) 3366, 2919, 1510.

HRMS (ESI) *m/z* calcd for $C_{18}H_{18}N_2O+H^+$: 279.1492 $[M+H]^+$; found: 279.1492.

Synthesis of **2d**

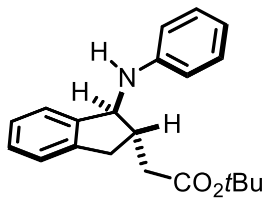

Compound **2d** was synthesised according to General Procedure C using aldehyde **S3** (49.2 mg, 0.2 mmol) and aniline (18 µL, 0.2 mmol). ^{1}H NMR analysis of the crude reaction mixture revealed a ratio of singly-cyclised *trans* product **2d** to doubly-cyclised *cis*-fused product **3d** of >20:1. Purification by column chromatography (SiO_2: acetone/pentane, 0–10%) afforded **2d** (64 mg, 99%) as a colourless oil.

^{1}H NMR (400 MHz, $CDCl_3$) δ = 7.37 (dd, *J*=7.4, 1.2, 1H), 7.34 – 7.20 (m, 5H), 6.86 – 6.77 (m, 3H), 4.80 (d, *J*=7.8, 1H), 4.05 (s, 1H), 3.34 – 3.23 (m, 1H), 2.85 – 2.66 (m, 3H), 2.54 (dd, *J*=14.8, 8.4, 1H), 1.54 (s, 9H).

^{13}C NMR (101 MHz, $CDCl_3$) δ = 172.1, 148.2, 144.7, 141.8, 129.5, 127.9, 126.8, 124.9, 124.2, 117.5, 113.1, 80.6, 63.4, 45.4, 39.3, 36.2, 28.2.

FT-IR (thin film) ν_{max} (cm^{-1}) 3390, 2976, 1721, 1601, 1505.

HRMS (ESI) *m/z* calcd for $C_{21}H_{25}NO_2+H^+$: 324.1958 $[M+H]^+$; found: 324.1960.

Synthesis of **2e**

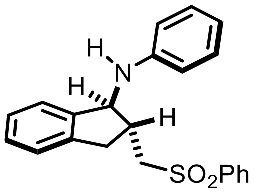

To a 1.6 mL vial equipped with stir bar were charged aldehyde **S4** (57.3 mg, 0.2 mmol, 1 eq.), aniline (18 µL, 0.2 mmol, 1 eq.), **HE1** (unsubstituted Hantzsch ester, 74 mg, 0.3 mmol, 1.5 eq.) and [Ir(dF(CF_3)ppy)$_2$(dtbbpy)]PF_6 (2.2 mg, 0.02 mmol, 1 mol%). Anhydrous degassed *N*,*N*-dimethylacetamide (1 mL, 0.2 mol L^{-1}) and acetic acid (2.5 µL, 0.04 mmol, 20 mol%) were added, and N_2 was bubbled through the reaction mixture for 2 minutes. The vial was then sealed and suspended in a HepatoChem PhotoRedOx Box equipped with an EvoluChem 450PF 450 nm 18 W LED light source (note: Setup 1 was used in this case due to lack of availability of equipment – no conclusions should be drawn as to the efficacy of this substrate under *Conditions A* with light Setup 2). The mixture was stirred 15 minutes in darkness (in order to facilitate imine formation) and then irradiated for 1 h. The reaction mixture was diluted with ethyl acetate (20 mL) and the organic phase was washed with saturated aqueous $NaHCO_3$ solution (2x 10 mL) and brine (2x 10 mL). The organic phase was dried over $MgSO_4$, filtered, and concentrated under reduced pressure. ^{1}H NMR analysis of the crude reaction mixture revealed a ratio of singly-cyclised *trans* product **2e** to doubly-cyclised *cis*-fused product **3e** of 9:1. The crude material was purified by column chromatography (SiO_2: ethyl acetate/pentane, 20–40%), affording **2e** (63 mg, 87%) as an off-white amorphous solid, a 9:1 inseparable mixture of **2e** and its double cyclisation analogue **3e**.

^{1}H NMR (400 MHz, $CDCl_3$) δ = 7.95 – 7.89 (m, 2H), 7.68 – 7.63 (m, 1H), 7.59 – 7.53 (m, 2H), 7.28 – 7.21 (m, 3H), 7.20 – 7.11 (m, 3H), 6.75 (tt, *J*=7.4, 1.1, 1H), 6.69 – 6.62 (m, 2H), 4.73 (d, *J*=8.5, 1H), 3.84 (br s, 1H), 3.60 (dd, *J*=13.9, 2.9, 1H), 3.44 – 3.26 (m, 2H), 2.85 (dd, *J*=16.0, 8.9, 1H), 2.71 – 2.61 (m, 1H).

^{13}C NMR (101 MHz, $CDCl_3$) δ = 147.6, 143.2, 141.2, 139.7, 133.9, 129.6, 129.5, 128.3, 128.1, 127.2, 125.1, 124.0, 118.3, 113.4, 63.6, 59.5, 43.3, 36.2.

FT-IR (thin film) ν_{max} (cm^{-1}) 3382, 3055, 2916, 1690, 1601, 1498, 1478.

HRMS (ESI) *m/z* calcd for $C_{22}H_{21}NO_2S+H^+$: 364.1366 $[M+H]^+$; found: 364.1363.

m.p. (from $CDCl_3$) 175-184 °C (decomp.)

Synthesis of **2f**

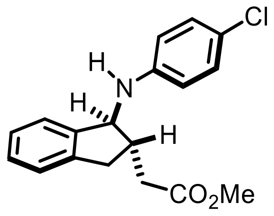

Compound **2f** was synthesised according to General Procedure C using aldehyde **6** (40.8 mg, 0.2 mmol) and 4-chloroaniline (26 mg, 0.2 mmol). ^{1}H NMR analysis of the crude reaction mixture revealed a ratio of singly-cyclised *trans* product **2f** to doubly-cyclised *cis*-fused product **3f** of >20:1. Purification by column chromatography (SiO_2: acetone/pentane, 5–15%) afforded **2f** (58 mg, 92%) as a colourless oil.

^{1}H NMR (400 MHz, $CDCl_3$) δ = 7.28 – 7.21 (m, 3H), 7.18 – 7.12 (m, 3H), 6.68 – 6.57 (m, 2H), 4.68 (dd, *J*=7.2, 7.1, 1H), 3.94 (br d, *J*=7.0, 1H), 3.65 (s, 3H), 3.26 – 3.14 (m, 1H), 2.74 (dd, *J*=14.4, 5.0, 1H), 2.71 – 2.62 (m, 2H), 2.56 (dd, *J*=14.4, 7.0, 1H).

^{13}C NMR (101 MHz, $CDCl_3$) δ = 173.2, 146.7, 144.1, 141.6, 129.4, 128.2, 127.0, 125.1, 124.2, 122.2, 114.3, 63.7, 51.8, 45.1, 37.8, 36.4.

FT-IR (thin film) ν_{max} (cm^{-1}) 3382 (w), 2950 (w), 1733 (s), 1599 (s), 1493.

HRMS (ESI) *m/z* calcd for $C_{18}H_{18}NO_2Cl+H^+$: 316.1099 $[M+H]^+$; found: 316.1102.

Synthesis of **2g**

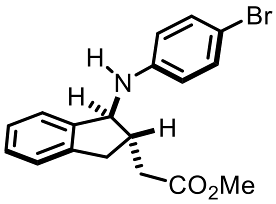

Compound **2g** was synthesised according to General Procedure C using aldehyde **6** (40.8 mg, 0.2 mmol) and 4-bromoaniline (23 µL, 0.2 mmol). ^{1}H NMR analysis of the crude reaction mixture revealed a ratio of singly-cyclised *trans* product **2g** to doubly-cyclised *cis*-fused product **3g** of >20:1. Purification by column chromatography (SiO_2: acetone/pentane, 0–15%) afforded **2g** (56 mg, 78%) as a yellow oil.

^{1}H NMR (400 MHz, $CDCl_3$) δ = 7.28 – 7.19 (m, 4H), 7.19 – 7.12 (m, 1H), 6.64 – 6.54 (m, 2H), 4.67 (dd, *J*=8.4, 8.1, 1H), 3.94 (d, *J*=8.4, 1H), 3.63 (s, 3H), 3.25 – 3.14 (m, 1H), 2.72 (dd, *J*=14.4, 5.0, 1H), 2.65 (dddd, *J*=10.2, 8.1, 5.5, 2.1, 2H), 2.54 (dd, *J*=14.4, 7.2, 1H).

^{13}C NMR (101 MHz, $CDCl_3$) δ = 173.2, 147.1, 144.0, 141.6, 132.2, 128.2, 127.0, 125.1, 124.2, 114.7, 109.2, 63.6, 51.8, 45.1, 37.8, 36.4.

FT-IR (thin film) ν_{max} (cm^{-1}) 3384, 2917, 1732, 1593, 1497.

HRMS (ESI) *m/z* calcd for $C_{18}H_{18}NO_2Br+H^+$: 360.0594 [*M*+H]$^+$; found: 360.0596.

Synthesis of **2h**

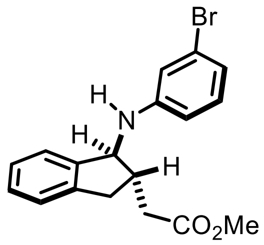

Compound **2h** was synthesised according to General Procedure C using aldehyde **6** (40.8 mg, 0.2 mmol) and 3-bromoaniline (22 µL, 0.2 mmol). ^{1}H NMR analysis of the crude reaction mixture revealed a ratio of singly-cyclised *trans* product **2h** to doubly-cyclised *cis*-fused products **3h-1** and **3h-2** of 4.5:1. Purification by column chromatography (SiO_2: acetone/pentane, 0–15%) afforded **2h** (49 mg, 68%) as a colourless oil.

^{1}H NMR (500 MHz, $CDCl_3$) δ = 7.28 – 7.21 (m, 3H), 7.17 (td, *J*=6.7, 2.5, 1H), 7.03 (dd, *J*=8.2, 8.0, 1H), 6.89 (s, 1H), 6.87 – 6.82 (m, 1H), 6.63 (dd, *J*=8.2, 2.5, 1H), 4.70 (d, *J*=7.3, 1H), 3.66 (s, 3H), 3.26 – 3.16 (m, 1H), 2.74 (dd, *J*=14.8, 5.3, 1H), 2.70 – 2.62 (m, 2H), 2.56 (dd, *J*=14.8, 7.2, 1H).

^{13}C NMR (126 MHz, $CDCl_3$) δ = 173.2, 149.2, 143.7, 141.6, 130.8, 128.3, 127.1, 125.1, 124.2, 123.6, 120.7, 115.9, 112.0, 63.5, 51.9, 45.1, 37.7, 36.4.

FT-IR (thin film) ν_{max} (cm^{-1}) 3381, 2917, 1727, 1592, 1479.

HRMS (ESI) *m/z* calcd for $C_{18}H_{18}NO_2Br+Na^+$: 382.0413 [*M*+Na]$^+$; found: 382.0415.

Synthesis of **2i**

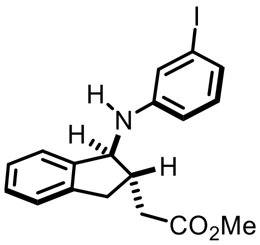

Compound **2i** was synthesised according to General Procedure C using aldehyde **6** (40.8 mg, 0.2 mmol) and 3-iodoaniline (44 mg, 0.2 mmol). ^{1}H NMR analysis of the crude reaction mixture revealed a ratio of singly-cyclised *trans* product **2i** to doubly-cyclised *cis*-fused product **3i** of >20:1. Purification by column chromatography (SiO_2: acetone/pentane, 0–10%) afforded **2i** (81 mg, 99%) as a yellow oil.

^{1}H NMR (400 MHz, $CDCl_3$) δ = 7.29 – 7.13 (m, 4H), 7.10 – 7.02 (m, 2H), 6.89 (dd, *J*=8.3, 7.9, 1H), 6.66 (ddd, *J*=8.3, 2.4, 0.9, 1H), 4.69 (dd, *J*=8.5, 7.7, 1H), 3.96 (d, *J*=8.5, 1H), 3.66 (s, 3H), 3.26 – 3.15 (m, 1H), 2.74 (dd, *J*=14.4, 4.9, 1H), 2.66 (m, 2H), 2.61 – 2.51 (m, 1H).

^{13}C NMR (101 MHz, $CDCl_3$) δ = 173.2, 149.3, 143.9, 141.5, 131.0, 128.2, 127.1, 126.6, 125.0, 124.2, 121.7, 112.3, 95.5, 63.2, 51.9, 45.3, 37.7, 36.3.

FT-IR (thin film) ν_{max} (cm^{-1}) 3388, 2950, 2918, 1732 (s), 1587, 1476.

HRMS (ESI) *m/z* calcd for $C_{18}H_{18}NO_2I+H^+$: 408.0455 $[M+H]^+$; found: 408.0457.

Synthesis of **2j**

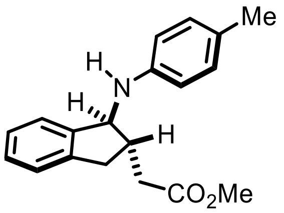

Compound **2j** was synthesised according to General Procedure C using aldehyde **6** (40.8 mg, 0.2 mmol) and *p*-toluidine (22 mg, 0.2 mmol). ^{1}H NMR analysis of the crude reaction mixture revealed a ratio of singly-cyclised *trans* product **2j** to doubly-cyclised *cis*-fused product **3j** of >20:1. Purification by column chromatography (SiO_2: acetone/pentane, 0–10%) afforded **2j** (58 mg, 98%) as a colourless oil.

^{1}H NMR (400 MHz, $CDCl_3$) δ = 7.29 (dd, *J*=7.4, 1.1, 1H), 7.25 – 7.20 (m, 2H), 7.16 (dq, *J*=7.4, 4.1, 1H), 7.02 (d, *J*=8.3, 2H), 6.66 (d, *J*=8.3, 2H), 4.70 (d, *J*=7.1, 1H), 3.79 (br s, 1H), 3.66 (s, 3H), 3.27 – 3.17 (m, 1H), 2.79 (dd, *J*=14.9, 4.7, 1H), 2.71 – 2.60 (m, 2H), 2.55 (dd, *J*=14.9, 8.1, 1H), 2.27 (s, 3H).

^{13}C NMR (101 MHz, $CDCl_3$) δ = 173.3, 145.9, 144.7, 141.6, 130.0, 127.9, 126.9, 126.9, 124.9, 124.3, 113.4, 63.9, 51.7, 45.3, 37.9, 36.4, 20.5.

FT-IR (thin film) ν_{max} (cm^{-1}) 3032, 2949, 1732, 1617, 1519.

HRMS (ESI) *m/z* calcd for $C_{19}H_{21}NO_2+H^+$: 296.1645 $[M+H]^+$; found: 296.1644.

Synthesis of **2k**

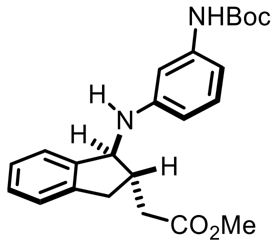

Compound **2k** was synthesised according to General Procedure C using aldehyde **6** (40.8 mg, 0.2 mmol) and *tert*-butyl (3-aminophenyl)carbamate (42 mg, 0.2 mmol). ^{1}H NMR analysis of the crude reaction mixture revealed a ratio of singly-cyclised *trans* product **2k** to doubly-cyclised *cis*-fused product **3k** of 5.5:1. Purification by column chromatography (SiO_2: acetone/pentane, 0–25%) afforded **2k** (61 mg, 77%) as a colourless crystalline solid.

^{1}H NMR (400 MHz, $CDCl_3$) δ = 7.27 (d, *J*=8.0, 1H), 7.21 (dd, *J*=3.9, 1.3, 2H), 7.15 (ddd, *J*=8.0, 3.9, 3.4, 1H), 7.08 (dd, *J*=8.2, 8.1, 1H), 6.94 (s, 1H), 6.60 (dd, *J*=8.1, 1.5, 1H), 6.45 (br s, 1H), 6.41 (dd, *J*=8.2, 1.5, 1H), 4.72 (d, *J*=7.3, 1H), 3.97 (br s, 1H), 3.65 (s, 3H), 3.20 (app q, *J*=10.7, 1H), 2.79 (dd, *J*=14.8, 4.4, 1H), 2.69 – 2.59 (m, 2H), 2.59 – 2.49 (m, 1H), 1.51 (s, 9H).

^{13}C NMR (101 MHz, $CDCl_3$) δ = 173.3, 152.8, 148.9, 144.3, 141.6, 139.7, 130.0, 128.0, 126.9, 124.9, 124.3, 107.9, 107.7, 103.2, 80.4, 63.3, 51.7, 45.4, 37.7, 36.3, 28.5.

FT-IR (thin film) ν_{max} (cm^{-1}) 3368, 2976, 1724, 1608, 1530.

HRMS (ESI) *m/z* calcd for $C_{23}H_{28}N_2O_4$+H^+: 397.2122 [*M*+H]$^+$; found: 397.2122.

m.p. (from $CDCl_3$) 50-52 °C

Synthesis of 2l

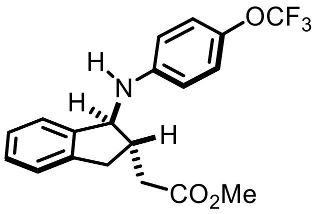

Compound **2l** was synthesised according to General Procedure C using aldehyde **6** (40.8 mg, 0.2 mmol) and 4-trifluoromethoxyaniline (27 µL, 0.2 mmol). ^{1}H NMR analysis of the crude reaction mixture revealed a ratio of singly-cyclised *trans* product **2l** to doubly-cyclised *cis*-fused product **3l** of >20:1. Purification by column chromatography (SiO_2: acetone/pentane, 0–10%) afforded **2l** (58 mg, 79%) as a colourless oil.

^{1}H NMR (400 MHz, $CDCl_3$) δ = 7.29 – 7.12 (m, 4H), 7.05 (d, *J*=4.6, 2H), 6.67 (d, *J*=4.6, 2H), 4.69 (dd, *J*=7.3, 7.0, 1H), 4.00 (d, *J*=7.0, 1H), 3.64 (s, 3H), 3.25 – 3.18 (m, 1H)., 2.77 – 2.62 (m, 3H), 2.59 – 2.53 (m, 1H).

^{19}F NMR (377 MHz, $CDCl_3$) δ = -58.5.

^{13}C NMR (126 MHz, $CDCl_3$) δ = 173.2, 146.8, 143.9, 141.6, 140.7, 128.2, 127.1, 125.1, 124.2, 122.7, 120.9 (q, *J*=255.8), 113.4, 63.8, 51.8, 45.0, 37.8, 36.4.

FT-IR (thin film) ν_{max} (cm^{-1}) 3384, 2955, 1730, 1611, 1512.

HRMS (ESI) *m/z* calcd for $C_{19}H_{18}NO_3F_3+H^+$: 366.1312 $[M+H]^+$; found: 366.1317.

Synthesis of **2m**

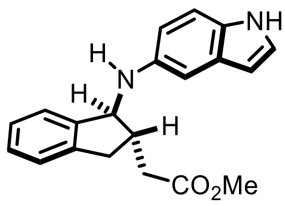

Compound **2m** was synthesised according to General Procedure C using aldehyde **6** (40.8 mg, 0.2 mmol) and 1*H*-indol-5-amine (26.4 mg, 0.2 mmol). ^{1}H NMR analysis of the crude reaction mixture revealed a ratio of singly-cyclised *trans* product **2m** to doubly-cyclised *cis*-fused product **3m** of 2.3:1. Purification by column chromatography (SiO_2: acetone/pentane, 0–25%) afforded **2m** (38.4 mg, 60%) as a colourless oil

^{1}H NMR (400 MHz, $CDCl_3$) δ = 7.90 (br s, 1H), 7.44 (d, *J*=8.4, 1H), 7.32 (dd, *J*=7.5, 1.1, 1H), 7.25 – 7.19 (m, 2H), 7.14 (ddd, *J*=7.5, 5.5, 3.1, 1H), 7.00 (dd, *J*=3.3, 2.3, 1H), 6.73 (d, *J*=2.0, 1H), 6.61 (dd, *J*=8.4, 2.0, 1H), 6.44 (ddd, *J*=3.3, 2.1, 0.9, 1H), 4.75 (d, *J*=7.8, 1H), 3.65 (s, 3H), 3.28 – 3.17 (m, 1H), 2.81 (dd, *J*=14.9, 5.1, 1H), 2.75 – 2.63 (m, 2H), 2.63 – 2.51 (m, 1H).

^{13}C NMR (101 MHz, $CDCl_3$) δ = 173.3, 144.8, 144.4, 141.5, 137.4, 127.8, 126.8, 124.8, 124.3, 121.8, 121.4, 120.6, 110.2, 102.5, 93.8, 64.3, 51.6, 45.1, 37.8, 36.3.

FT-IR (thin film) ν_{max} (cm^{-1}) 3400, 2950, 1721, 1629, 1513.

HRMS (ESI) *m/z* calcd for $C_{20}H_{20}N_2O_2+H^+$: 321.1598 $[M+H]^+$; found: 321.1596.

Synthesis of **2n**

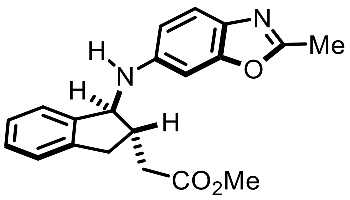

Compound **2n** was synthesised according to General Procedure C using aldehyde **6** (40.8 mg, 0.2 mmol) and 2-methylbenzo[*d*]oxazol-6-amine (30 mg, 0.2 mmol). ^{1}H NMR analysis of the crude reaction mixture revealed a ratio of singly-cyclised *trans* product **2n** to doubly-cyclised *cis*-fused product **3n** of 4.3:1. Purification by column chromatography (SiO_2: acetone/pentane, 0–15%) afforded **2n** (46 mg, 69%) as a colourless oil.

^{1}H NMR (400 MHz, $CDCl_3$) δ = 7.40 (d, *J*=8.5, 1H), 7.29 – 7.21 (m, 3H), 7.19 – 7.12 (m, 1H), 6.83 (d, *J*=2.2, 1H), 6.65 (dd, *J*=8.5, 2.2, 1H), 4.72 (br s, 1H), 4.08 (br s, 1H), 3.64 (s, 3H), 3.28 – 3.16 (m, 1H), 2.80 – 2.62 (m, 3H), 2.62 – 2.51 (m, 4H).

^{13}C NMR (101 MHz, $CDCl_3$) δ = 173.2, 161.5, 152.7, 146.3, 144.1, 141.6, 133.3, 128.1, 127.0, 125.1, 124.2, 119.7, 111.5, 93.9, 64.1, 51.8, 44.9, 37.8, 36.4, 14.5.

FT-IR (thin film) ν_{max} (cm^{-1}) 3377, 2951, 1733, 1628, 1497.

HRMS (ESI) *m/z* calcd for $C_{20}H_{20}N_2O_3$+H^+: 337.1547 [*M*+H]$^+$; found: 337.1549.

Synthesis of 2o

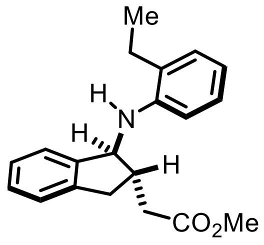

2o-*trans*

Compound **2o** was synthesised according to General Procedure C using aldehyde **6** (40.8 mg, 0.2 mmol) and 2-ethylaniline (25 µL, 0.2 mmol). ^{1}H NMR analysis of the crude reaction mixture revealed a dr of 4:1 for product **2o**, while doubly-cyclised product **3o** was not observed. Purification by column chromatography (SiO_2: acetone/pentane, 0–10%) afforded **2o** (58 mg, 94%, 4:1 dr) as a mixture of diastereomers. The diastereomers were separated by preparative thin-layer chromatography (ethyl acetate/hexane, 10%), affording **2o-*trans*** as a colourless crystalline solid. An insufficient amount of the minor diastereomer (*cis*) was obtained for complete characterisation.

^{1}H NMR (400 MHz, $CDCl_3$) δ = 7.31 – 7.22 (m, 3H), 7.21 – 7.08 (m, 3H), 6.85 (d, *J*=8.0, 1H), 6.74 (td, *J*=7.4, 1.2, 1H), 4.81 (d, *J*=7.2, 1H), 3.90 (br s, 1H), 3.65 (s, 3H), 3.30 – 3.18 (m, 1H), 2.83 – 2.76 (m, 1H), 2.76 – 2.65 (m, 2H), 2.56 (dd, *J*=14.8, 8.2, 1H), 2.49 (q, *J*=7.5, 2H), 1.24 (t, *J*=7.5, 3H).

^{13}C NMR (101 MHz, $CDCl_3$) δ = 173.3, 145.4, 144.7, 141.7, 128.3, 128.0, 127.6, 127.2, 127.0, 125.0, 124.3, 117.4, 110.6, 63.3, 51.7, 45.6, 37.8, 36.4, 24.1, 13.1.

FT-IR (thin film) ν_{max} (cm^{-1}) 3414, 2963, 1735, 1604, 1585, 1509.

HRMS (ESI) *m/z* calcd for $C_{20}H_{23}NO_2+H^+$: 310.1802 $[M+H]^+$; found: 310.1803.

m.p. (from MeOH) 86-88 °C

Synthesis of 2p

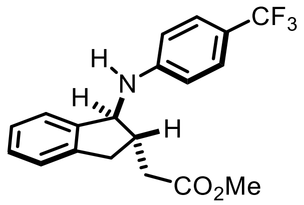

2p-*trans*

Compound **2p** was synthesised according to General Procedure C using aldehyde **6** (40.8 mg, 0.2 mmol) and 4-trifluoromethylaniline (26 µL, 0.2 mmol). ^{1}H NMR analysis of the crude reaction mixture revealed a dr of 10:1 for product **2p**, while doubly-cyclised product **3p** was not observed. Purification by column chromatography (SiO_2: acetone/pentane, 0–10%) afforded **2p** (67 mg, 96%, 10:1 dr) as a mixture of diastereomers. The diastereomers were separated by preparative thin-layer chromatography (ethyl acetate/hexane, 10%), affording **2p-*trans*** as a colourless oil. An insufficient amount of the minor diastereomer (*cis*) was obtained for complete characterisation.

^{1}H NMR (400 MHz, $CDCl_3$) δ = 7.42 (d, *J*=8.5, 2H), 7.26 – 7.22 (m, 2H), 7.18 (ddd, *J*=7.8, 5.1, 3.1, 1H), 6.73 (d, *J*=8.5, 2H), 4.78 (dd, *J*=8.7, 7.7, 1H), 4.27 (d, *J*=8.7, 1H), 3.64 (s, 3H), 3.30 – 3.17 (m, 1H), 2.76 – 2.65 (m, 3H), 2.64 – 2.53 (m, 1H).

^{19}F NMR (377 MHz, $CDCl_3$) δ = -61.1.

^{13}C NMR (176 MHz, $CDCl_3$) δ = 173.1, 150.6, 143.6, 141.6, 128.3, 127.1, 127.0 (q, *J*=3.8), 125.1, 125.0 (q, *J*=269.9), 124.1, 119.3 (q, *J*=32.6), 112.2, 63.1, 51.9, 45.1, 37.7, 36.4.

FT-IR (thin film) ν_{max} (cm^{-1}) 3382, 2954, 1728, 1615, 1532.

HRMS (ESI) *m/z* calcd for $C_{19}H_{18}NO_2F_3+H^+$: 350.1362 $[M+H]^+$; found: 350.1363.

Synthesis of **2q**

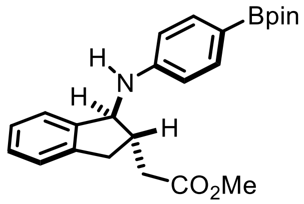

Compound **2q** was synthesised according to General Procedure C using aldehyde **6** (40.8 mg, 0.2 mmol) and 4-(4,4,5,5-tetramethyl-1,3,2-dioxaborolan-2-yl)aniline (44 mg, 0.2 mmol). ^{1}H NMR analysis of the crude reaction mixture revealed a dr of >20:1 for product **2q**, while doubly-cyclised product **3q** was not observed. Purification by column chromatography (SiO_2: acetone/pentane, 0–40%) afforded **2q** (53 mg, 65%) as a colourless oil.

^{1}**H NMR** (400 MHz, $CDCl_3$) δ = 7.66 (d, *J*=8.5, 2H), 7.27 – 7.24 (m, 1H), 7.22 (dd, *J*=3.7, 1.0, 2H), 7.18 – 7.12 (m, 1H), 6.70 (d, *J*=8.5, 2H), 4.79 (dd, *J*=6.8, 6.6, 1H), 4.15 (d, *J*=6.8, 1H), 3.64 (s, 3H), 3.27 – 3.14 (m, 1H), 2.77 (dd, *J*=14.7, 4.8, 1H), 2.71 – 2.61 (m, 2H), 2.56 (dd, *J*=14.7, 7.7, 1H), 1.33 (s, 12H).

^{13}**C NMR** (176 MHz, $CDCl_3$) δ = 173.2, 150.7, 144.2, 141.5, 136.7, 128.1, 127.0, 125.0, 124.2, 116.9, 112.2, 83.4, 62.9, 51.8, 45.5, 37.7, 36.4, 25.0, 25.0.

FT-IR (thin film) ν_{max} (cm^{-1}) 3377, 2977, 1733, 1603.

HRMS (ESI) *m/z* calcd for $C_{24}H_{30}NO_4B+H^+$: 408.2342 [*M*+H]$^+$; found: 408.2345.

Synthesis of 3a (1 mmol scale)

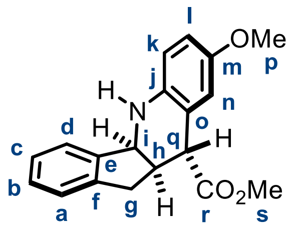

To an 8 mL Wheaton vial equipped with stir bar and rubber septum were charged aldehyde **6** (204 mg, 1 mmol, 1 eq.), 4-methoxyaniline (123 mg, 1 mmol, 1 eq.), **HE6** (ethyl-substituted Hantzsch ester, 55 mg, 0.2 mmol, 20 mol%) and $[Ir(dF(CF_3)ppy)_2(dtbbpy)]PF_6$ (5.5 mg, 0.005 mmol, 0.5 mol%). The solids were degassed by repeated evacuation and refilling with N_2 *via* the septum. Anhydrous degassed *N*,*N*-dimethylacetamide (5 mL, 0.2 mol L^{-1}) and acetic acid (12.5 µL, 0.2 mmol, 20 mol%) were added *via* the septum, and the mixture was stirred 30 minutes in darkness (in order to facilitate imine formation). The vial was placed in a Sigma Aldrich SynLED 467.5 nm 12 W LED 16-well array and irradiated for 16 h. The reaction mixture was diluted with ethyl acetate (100 mL) and the organic phase was washed with saturated aqueous $NaHCO_3$ solution (2x 50 mL) and brine (3x 20 mL). The organic phase was dried over $MgSO_4$, filtered, and concentrated under reduced pressure. ^{1}H NMR analysis of the crude reaction mixture revealed a ratio of doubly-cyclised *cis*-fused product **3a** to singly-cyclised *trans* product **2a** of 4.3:1. The crude material was purified by column chromatography (SiO_2: acetone/pentane, 5–15%), affording **3a** (238 mg, 77%) as a colourless oil.

^{1}H NMR (500 MHz, $CDCl_3$) δ 7.36 – 7.32 (m, 1H, ***H*-d**), 7.26 – 7.19 (m, 3H, ***H*-a,b,c**), 6.69 (d, *J* = 2.9 Hz, 1H, ***H*-n**), 6.66 (dd, *J* = 8.5, 2.9 Hz, 1H, ***H*-l**), 6.45 (d, *J* = 8.5 Hz, 1H, ***H*-k**), 4.91 (d, *J* = 6.9 Hz, 1H, ***H*-i**), 3.79 (s, 3H, ***H*-s**), 3.75 (s, 3H, ***H*-p**), 3.66 (d, *J* = 4.6 Hz, 1H, ***H*-q**), 3.27 – 3.18 (m, 1H, ***H*-h**), 3.05 (dd, *J* = 15.7, 7.6 Hz, 1H, ***H*-g'**), 2.77 (dd, *J* = 15.7, 7.8 Hz, 1H, ***H*-g''**).

^{13}C NMR (126 MHz, $CDCl_3$) δ 173.9 (***C*-r**), 151.7 (***C*-m**), 145.7 (***C*-e**), 141.9 (***C*-f**), 138.3 (***C*-j**), 128.0 (***C*-b**), 126.9 (***C*-c**), 125.2 (***C*-a**), 123.7 (***C*-d**), 118.0 (***C*-o**), 115.0 (***C*-n**), 114.9 (***C*-k**), 114.2 (***C*-l**), 57.4 (***C*-i**), 55.8 (***C*-p**), 52.2 (***C*-s**), 46.0 (***C*-q**), 40.3 (***C*-h**), 36.4 (***C*-g**).

FT-IR (thin film) ν_{max} (cm^{-1}) 3392, 2948, 1729, 1503.

HRMS (ESI) *m/z* calcd for $C_{19}H_{19}NO_3+H^+$: 310.1438 $[M+H]^+$; found: 310.1437.

Synthesis of 3b

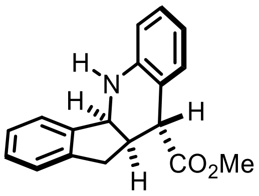

Compound **3b** was synthesised according to General Procedure D using aldehyde **6** (40.8 mg, 0.2 mmol) and aniline (18 µL, 0.2 mmol). ^{1}H NMR analysis of the crude reaction mixture revealed a ratio of doubly-cyclised *cis*-fused product **3b** to singly-cyclised *trans* product **2b** of 6.1:1. Purification by column chromatography (SiO_2: acetone/pentane, 5–10%) afforded **3b** (48 mg, 86%) as a colourless crystalline solid.

The stereochemical configuration of **3b** was confirmed by single crystal x-ray diffraction analysis.

^{1}H NMR (400 MHz, $CDCl_3$) δ = 7.35 – 7.30 (m, 1H), 7.21 (dd, *J*=4.6, 3.3, 3H), 7.05 (d, *J*=7.4, 1H), 7.00 (td, *J*=8.0, 1.2, 1H), 6.62 (td, *J*=7.4, 1.2, 1H), 6.46 (dd, *J*=8.0, 1.2, 1H), 4.92 (d, *J*=6.6, 1H), 3.76 (s, 3H), 3.67 (d, *J*=4.5, 1H), 3.23 – 3.14 (m, 1H), 3.01 (dd, *J*=15.7, 7.4, 1H), 2.76 (dd, *J*=15.7, 8.1, 1H).

^{13}C NMR (101 MHz, $CDCl_3$) δ = 174.2, 145.7, 144.1, 142.1, 129.7, 128.3, 128.2, 127.1, 125.4, 123.8, 117.2, 116.3, 114.1, 56.9, 52.3, 45.5, 40.3, 36.4.

FT-IR (thin film) ν_{max} (cm^{-1}) 3395, 2923, 1731, 1606, 1496.

HRMS (ESI) *m/z* calcd for $C_{18}H_{17}NO_2+H^+$: 280.1332 $[M+H]^+$; found: 280.1333.

m.p. (from $CDCl_3$) 156-160 °C

Synthesis of **3c**

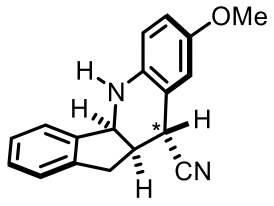

3c-major

Compound **3c** was synthesised according to General Procedure D using aldehyde **S1** (34.2 mg, 0.2 mmol) and 4-methoxyaniline (25 mg, 0.2 mmol). ^{1}H NMR analysis of the crude reaction mixture revealed a ratio of doubly-cyclised *cis*-fused products **3c** (with a 2:1 dr at the position indicated by an asterisk) to singly-cyclised *trans* product **2c** of 7.3:1. Purification by column chromatography (SiO_2: acetone/pentane, 5–10%) afforded **3c** (44 mg, 78%, 2:1 dr at the position indicated by an asterisk). The diastereomers were separated by preparative thin-layer chromatography (ethyl acetate/hexane, 10%), affording **3c-major** as a colourless crystalline solid. An insufficient amount of the minor diastereoisomer was obtained for full characterisation.

^{1}H NMR (400 MHz, $CDCl_3$) δ = 7.32 – 7.25 (m, 4H), 6.96 (d, *J*=2.9, 1H), 6.76 (dd, *J*=8.6, 2.9, 1H), 6.63 (d, *J*=8.6, 1H), 4.90 (d, *J*=7.1, 1H), 4.35 (br s, 1H), 3.77 (s, 3H), 3.48 (d, *J*=10.2, 1H), 3.34 (dd, *J*=16.3, 6.9, 1H), 3.13 (dd, *J*=16.3, 2.3, 1H), 2.87 (dddd, *J*=10.2, 7.1, 6.9, 2.3, 1H).

^{13}C NMR (101 MHz, $CDCl_3$) δ = 152.5, 144.6, 139.3, 137.3, 128.3, 127.5, 125.7, 124.1, 120.1, 116.8, 115.6, 115.4, 112.4, 58.7, 56.1, 41.4, 36.2, 33.1.

FT-IR (thin film) ν_{max} (cm^{-1}) 3376, 2923, 1619, 1504.

HRMS (ESI) *m/z* calcd for $C_{18}H_{17}N_2O+H^+$: 277.1335 $[M+H]^+$; found: 277.1333.

m.p. (from $CDCl_3$) 89-92 °C

Synthesis of **3d**

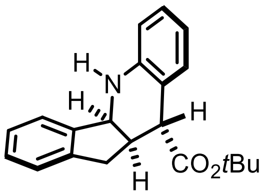

Compound **3d** was synthesised according to General Procedure D using aldehyde **S3** (49.2 mg, 0.2 mmol) and aniline (18 µL, 0.2 mmol). ^{1}H NMR analysis of the crude reaction mixture revealed a ratio of doubly-cyclised *cis*-fused product **3d** to singly-cyclised *trans* product **2d** of 3.5:1. Purification by column chromatography (SiO_2: acetone/pentane, 0–10%) afforded **3d** (47 mg, 74%) as a colourless crystalline solid.

^{1}H NMR (400 MHz, $CDCl_3$) δ = 7.35 – 7.32 (m, 1H), 7.23 – 7.20 (m, 3H), 7.07 (d, *J*=7.6, 1H), 7.00 (td, *J*=8.1, 1.2, 1H), 6.62 (td, *J*=7.6, 1.2, 1H), 6.49 (d, *J*=8.1, 1H), 4.90 (d, *J*=6.5, 1H), 3.52 (d, *J*=4.7, 1H), 3.15 – 2.98 (m, 2H), 2.77 (dd, *J*=15.2, 7.1, 1H), 1.48 (s, 9H).

^{13}C NMR (101 MHz, $CDCl_3$) δ = 173.1, 143.9, 142.2, 129.3, 128.1, 128.0, 127.0, 125.4, 123.8, 117.2, 114.0, 81.2, 57.0, 46.6, 40.4, 36.4, 28.3.

FT-IR (thin film) ν_{max} (cm^{-1}) 3397, 2976, 1720, 1605, 1503.

HRMS (ESI) *m/z* calcd for $C_{21}H_{23}NO_2+H^+$: 322.1802 $[M+H]^+$; found: 322.1800.

m.p. (from $CDCl_3$) 45-48 °C

Synthesis of **3e**

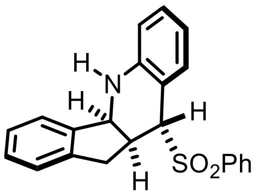

To an 8 mL glass vial equipped with stir bar were charged aldehyde **S4** (57.3 mg, 0.2 mmol, 1 eq.), aniline (18 µL, 0.2 mmol, 1 eq.), **HE6** (ethyl-substituted Hantzsch ester, 84 mg, 0.3 mmol, 1.5 eq.) and $[Ir(dF(CF_3)ppy)_2(dtbbpy)]PF_6$ (2.2 mg, 0.02 mmol, 1 mol%). Anhydrous degassed 1,4-dioxane (2 mL, 0.1 mol L^{-1}) and acetic acid (2.5 µL, 0.04 mmol, 20 mol%) were added, and N_2 was bubbled through the reaction mixture for 2 minutes. The vial was then sealed and suspended in a HepatoChem PhotoRedOx Box equipped with an EvoluChem 450PF 450 nm 18 W LED light source (note: Setup 1 was used in this case due to lack of availability of equipment – no conclusions should be drawn as to the efficacy of this substrate under *Conditions A* with light Setup 2). The mixture was stirred 15 minutes in darkness (in order to facilitate imine formation) and then irradiated for 16 h. The reaction mixture was diluted with ethyl acetate (20 mL) and the organic phase was washed with saturated aqueous $NaHCO_3$ solution (2x 10 mL) and brine (2x 10 mL). The organic phase was dried over $MgSO_4$, filtered, and concentrated under reduced pressure. ^{1}H NMR analysis of the crude reaction mixture revealed a ratio of doubly-cyclised *cis*-fused product **3e** to singly-cyclised *trans* product **2e** of 19:1. The crude material was purified by column chromatography (SiO_2: ethyl acetate/pentane, 20–40%), affording **3e** (58 mg, 80%) as a colourless crystalline solid.

^{1}H NMR (400 MHz, $CDCl_3$) δ = 7.75 – 7.68 (m, 2H), 7.68 – 7.61 (m, 1H), 7.52 – 7.45 (m, 2H), 7.33 – 7.28 (m, 1H), 7.25 – 7.13 (m, 3H), 7.00 (ddd, *J*=8.4, 7.2, 1.5, 1H), 6.61 (dd, *J*=7.7, 1.5, 1H), 6.41 (ddd, *J*=7.7, 7.2, 1.2, 1H), 6.32 (dd, *J*=8.4, 1.2, 1H), 4.85 (d, *J*=6.3, 1H), 4.32 (d, *J*=1.6, 1H), 3.67 (s, 1H), 3.63 – 3.54 (m, 1H), 2.97 (dd, *J*=15.3, 7.6, 1H), 2.80 (dd, *J*=15.3, 10.8, 1H).

^{13}C NMR (101 MHz, $CDCl_3$) δ = 145.5, 144.9, 142.0, 137.5, 133.9, 132.9, 130.1, 129.7, 129.0, 128.6, 127.3, 125.2, 123.8, 116.1, 113.8, 106.9, 66.9, 55.3, 37.2, 36.8.

FT-IR (thin film) ν_{max} (cm^{-1}) 3390, 3057, 2903, 1607, 1498, 1301, 1143.

HRMS (ESI) *m/z* calcd for $C_{22}H_{19}NO_2S+H^+$: 362.1209 $[M+H]^+$; found: 362.1203.

m.p. (from $CDCl_3$) 157-160 °C

Synthesis of **3f**

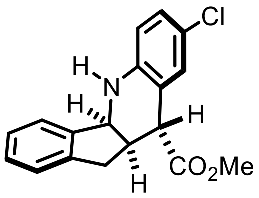

Compound **3f** was synthesised according to General Procedure D using aldehyde **6** (40.8 mg, 0.2 mmol) and 4-chloroaniline (26 mg, 0.2 mmol). ^{1}H NMR analysis of the crude reaction mixture revealed a ratio of doubly-cyclised *cis*-fused product **3f** to singly-cyclised *trans* product **2f** of 2.7:1. Purification by column chromatography (SiO_2: acetone/pentane, 5–15%) afforded **3f** (45 mg, 72%) as a white amorphous solid.

^{1}H NMR (400 MHz, $CDCl_3$) δ = 7.43 – 7.33 (m, 1H), 7.27 – 7.16 (m, 3H), 7.06 (d, *J*=2.4, 1H), 6.97 (dd, *J*=8.5, 2.4, 1H), 6.53 (d, *J*=8.5, 1H), 4.92 (d, *J*=6.8, 1H), 3.78 (s, 3H), 3.70 (d, *J*=4.4, 1H), 3.27 – 3.15 (m, 1H), 3.04 (dd, *J*=15.8, 7.7, 1H), 2.76 (dd, *J*=15.8, 8.1, 1H).

^{13}C NMR (126 MHz, $CDCl_3$) δ = 173.6, 145.1, 142.6, 142.1, 129.5, 128.4, 128.2, 127.2, 125.4, 123.8, 121.5, 117.6, 115.2, 56.9, 52.5, 45.3, 40.0, 36.2.

FT-IR (thin film) ν_{max} (cm^{-1}) 3391, 2951, 1732, 1607, 1495.

HRMS (ESI) *m/z* calcd for $C_{18}H_{16}NO_2Cl+H^+$: 314.0942 [*M*+H]$^+$; found: 314.0942.

m.p. (from $CDCl_3$) 53-54 °C

Synthesis of **3g**

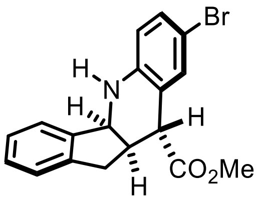

Compound **3g** was synthesised according to General Procedure D using aldehyde **6** (40.8 mg, 0.2 mmol) and 4-bromoaniline (23 µL, 0.2 mmol). ^{1}H NMR analysis of the crude reaction mixture revealed a ratio of doubly-cyclised *cis*-fused product **3g** to singly-cyclised *trans* product **2g** of 2.1:1. Purification by column chromatography (SiO_2: acetone/pentane, 0–15%) afforded **3g** (49 mg, 69%) as an off-white solid.

^{1}H NMR (500 MHz, $CDCl_3$) δ = 7.33 – 7.30 (m, 1H), 7.24 – 7.19 (m, 3H), 7.17 (d, *J*=2.3, 1H), 7.07 (dd, *J*=8.5, 2.3, 1H), 6.32 (d, *J*=8.5, 1H), 4.90 (d, *J*=6.5, 1H), 3.76 (s, 3H), 3.65 (d, *J*=4.0, 1H), 3.17 (dddd, *J*=8.6, 7.5, 6.5, 4.0, 1H), 2.99 (dd, *J*=15.6, 7.5, 1H), 2.72 (dd, *J*=15.6, 8.6, 1H).

^{13}C NMR (126 MHz, $CDCl_3$) δ = 173.6, 145.2, 143.2, 142.1, 132.4, 131.0, 128.4, 127.2, 125.4, 123.7, 117.8, 115.5, 108.2, 56.7, 52.5, 45.2, 40.0, 36.2.

FT-IR (thin film) ν_{max} (cm^{-1}) 3398, 2949, 1730, 1599, 1492.

HRMS (ESI) *m/z* calcd for $C_{18}H_{16}NO_2Br+H^+$: 358.0437 $[M+H]^+$; found: 358.0437.

m.p. (from $CDCl_3$) 56-58 °C

Synthesis of 3h

Compound **3h** was synthesised according to General Procedure D using aldehyde **6** (40.8 mg, 0.2 mmol) and 3-bromoaniline (22 µL, 0.2 mmol). ^{1}H NMR analysis of the crude reaction mixture revealed a ratio of doubly-cyclised *cis*-fused products **3h-1** & **3h-2** to singly-cyclised *trans* product **2h** of 1.6:1. Purification by column chromatography (SiO_2: acetone/pentane, 0–15%) afforded **3h-1** (26 mg) as an off-white solid and **3h-2** (18 mg) as an off-white solid, with a combined yield of 61% (3:2 rr).

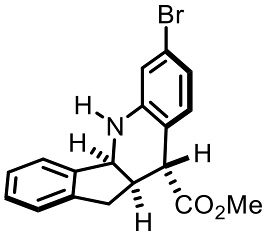

3h-1

^{1}H NMR (500 MHz, $CDCl_3$) δ = 7.35 – 7.27 (m, 1H), 7.25 – 7.18 (m, 3H), 6.91 (dd, *J*=8.0, 0.8, 1H), 6.71 (dd, *J*=8.1, 1.9, 1H), 6.59 (d, *J*=1.9, 1H), 4.90 (d, *J*=6.6, 1H), 3.75 (s, 3H), 3.64 (d, *J*=4.0, 1H), 3.18 (dddd, *J*=8.5, 7.4, 6.5, 3.9, 1H), 2.98 (dd, *J*=15.6, 7.5, 1H), 2.72 (dd, *J*=15.6, 8.5, 1H).

^{13}C NMR (126 MHz, $CDCl_3$) δ = 173.7, 145.3, 145.1, 142.1, 131.4, 128.4, 127.2, 125.5, 123.7, 121.8, 119.9, 116.4, 114.7, 56.6, 52.5, 45.0, 40.0, 36.2.

FT-IR (thin film) ν_{max} (cm^{-1}) 3396, 2918, 1727, 1597, 1490.

HRMS (ESI) *m/z* calcd for $C_{18}H_{16}NO_2Br+H^+$: 358.0437 $[M+H]^+$; found: 358.0439.

m.p. (from $CDCl_3$) 47-50 °C

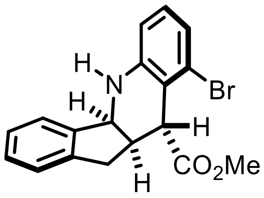

3h-2

^{1}H NMR (500 MHz, $CDCl_3$) δ = 7.33 – 7.29 (m, 1H), 7.25 – 7.15 (m, 3H), 6.89 – 6.79 (m, 2H), 6.33 (d, *J*=7.9, 1H), 4.76 (d, *J*=6.7, 1H), 4.26 (d, *J*=2.1, 1H), 3.74 (s, 3H), 3.39 (dddd, *J*=10.3, 7.9, 6.7, 2.1, 1H), 2.99 (dd, *J*=15.7, 7.9, 1H), 2.70 (dd, *J*=15.7, 10.3, 1H).

^{13}C NMR (126 MHz, $CDCl_3$) δ = 173.4, 146.0, 144.7, 142.9, 129.0, 128.4, 127.0, 126.7, 125.2, 123.6, 120.9, 115.7, 113.2, 56.7, 52.5, 45.7, 41.2, 36.3.

FT-IR (thin film) ν_{max} (cm^{-1}) 3383, 2949, 1726, 1595, 1471.

HRMS (ESI) *m/z* calcd for $C_{18}H_{16}NO_2Br+H^+$: 358.0437 $[M+H]^+$; found: 358.0438.

m.p. (from $CDCl_3$) 56-58 °C

Synthesis of 3i

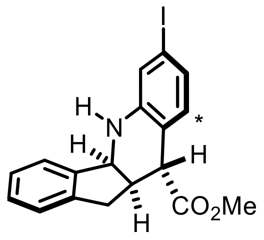

3i-major

Compound **3i** was synthesised according to General Procedure D using aldehyde **6** (40.8 mg, 0.2 mmol) and 3-iodoaniline (24 µL, 0.2 mmol). ^{1}H NMR analysis of the crude reaction mixture revealed a ratio of doubly-cyclised *cis*-fused products **3i** (5:1 rr at the position indicated by the asterisk) to singly-cyclised *trans* product **2i** of 2.9:1. Purification by column chromatography (SiO_2: acetone/pentane, 0–10%) afforded **3i** (58 mg, 71%, 5:1 rr at the position indicated by an asterisk). The regioisomers were separated by preparative thin-layer chromatography (ethyl acetate/hexane, 10%), affording **3i-major** as a yellow amorphous solid, a single regioisomer. An insufficient amount of the minor regioisomer was obtained for full characterisation.

^{1}H NMR (400 MHz, $CDCl_3$) δ = 7.33 (d, *J*=6.5, 1H), 7.25 – 7.17 (m, 3H), 6.95 (dd, *J*=8.0, 1.7, 1H), 6.84 (d, *J*=1.7, 1H), 6.78 (d, *J*=8.0, 1H), 4.89 (d, *J*=6.6, 1H), 3.75 (s, 3H), 3.65 (d, *J*=4.1, 1H), 3.24 – 3.13 (m, 1H), 3.00 (dd, *J*=15.7, 7.5, 1H), 2.73 (dd, *J*=15.7, 8.5, 1H).

^{13}C NMR (126 MHz, $CDCl_3$) δ = 173.7, 145.2, 144.9, 142.1, 131.5, 128.4, 127.2, 126.1, 125.4, 123.8, 122.5, 115.7, 93.5, 56.7, 52.5, 45.1, 39.9, 36.2.

FT-IR (thin film) ν_{max} (cm^{-1}) 3395, 2948, 1730, 1592, 1487.

HRMS (ESI) *m/z* calcd for $C_{18}H_{16}NO_2I+H^+$: 406.0298 [*M*+H]$^+$; found: 406.0298.

m.p. (from $CDCl_3$) 57-59 °C

Synthesis of **3j**

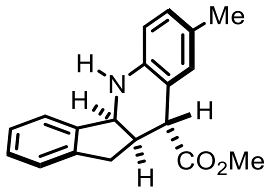

Compound **3j** was synthesised according to General Procedure D using aldehyde **6** (40.8 mg, 0.2 mmol) and *p*-toluidine (22 mg, 0.2 mmol). ^{1}H NMR analysis of the crude reaction mixture revealed a ratio of doubly-cyclised *cis*-fused product **3j** to singly-cyclised *trans* product **2j** of >20:1. Purification by column chromatography (SiO_2: acetone/pentane, 0–10%) afforded **3j** (54 mg, 93%) as a brown oil.

^{1}H NMR (400 MHz, $CDCl_3$) δ = 7.34 – 7.29 (m, 1H), 7.25 – 7.16 (m, 3H), 6.87 – 6.84 (m, 1H), 6.82 (d, *J*=8.0, 1H), 6.39 (d, *J*=8.0, 1H), 4.90 (d, *J*=6.8, 1H), 3.81 (br s, 1H), 3.77 (s, 3H), 3.63 (d, *J*=4.6, 1H), 3.16 (dddd, *J*=7.8, 7.4, 6.8, 4.6, 1H), 3.01 (dd, *J*=15.6, 7.4, 1H), 2.75 (dd, *J*=15.6, 7.8, 1H), 2.21 (s, 3H).

^{13}C NMR (151 MHz, $CDCl_3$) δ = 174.3, 145.8, 142.1, 141.9, 130.0, 129.0, 128.1, 127.0, 126.3, 125.4, 123.8, 116.5, 114.1, 57.2, 52.3, 45.7, 40.5, 36.4, 20.6.

FT-IR (thin film) ν_{max} (cm^{-1}) 3395, 2948, 1730, 1592, 1487, 1434.

HRMS (ESI) *m/z* calcd for $C_{19}H_{19}NO_2+H^+$: 294.1489 $[M+H]^+$; found: 294.1486.

Synthesis of 3k

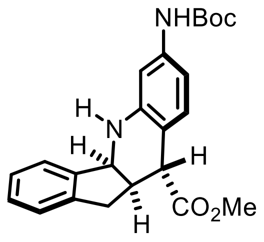

Compound **3k** was synthesised according to General Procedure D using aldehyde **6** (40.8 mg, 0.2 mmol) and *tert*-butyl (3-aminophenyl)carbamate (42 mg, 0.2 mmol). ^{1}H NMR analysis of the crude reaction mixture revealed a ratio of doubly-cyclised *cis*-fused product **3k** to singly-cyclised *trans* product **2k** of 3.5:1. Purification by column chromatography (SiO_2: acetone/pentane, 0–25%) afforded **3k** (62 mg, 79%) as a colourless crystalline solid.

^{1}H NMR (400 MHz, $CDCl_3$) δ = 7.41 – 7.34 (m, 1H), 7.24 – 7.13 (m, 3H), 6.96 (d, *J*=8.2, 1H), 6.85 (s, 1H), 6.53 (d, *J*=8.2, 1H), 6.34 (s, 1H), 4.93 (d, *J*=6.9, 1H), 3.75 (s, 3H), 3.67 (d, *J*=4.3, 1H), 3.21 (dddd, *J*=8.1, 7.7, 6.9, 4.3, 1H), 3.01 (dd, *J*=15.8, 7.7, 1H), 2.76 (dd, *J*=15.8, 8.1, 1H), 1.48 (s, 9H).

^{13}C NMR (176 MHz, $CDCl_3$) δ = 174.2, 152.8, 145.6, 144.7, 142.2, 138.4, 130.3, 128.2, 127.1, 125.4, 123.7, 120.1, 110.8, 107.4, 103.6, 56.8, 52.3, 45.0, 40.2, 36.3, 35.6, 28.5, 28.5, 28.4.

FT-IR (thin film) ν_{max} (cm^{-1}) 3378, 2976, 1725, 1619, 1530.

HRMS (ESI) *m/z* calcd for $C_{23}H_{26}N_2O_4$+H^+: 395.1965 [*M*+H]$^+$; found: 395.1969.

m.p. (from $CDCl_3$) 90-93 °C

Synthesis of 3l

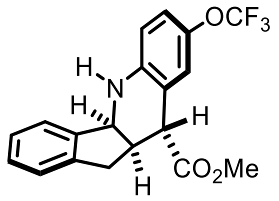

Compound **3l** was synthesised according to General Procedure D using aldehyde **6** (40.8 mg, 0.2 mmol) and 4-trifluoromethoxyaniline (27 µL, 0.2 mmol). ^{1}H NMR analysis of the crude reaction mixture revealed a ratio of doubly-cyclised *cis*-fused product **3l** to singly-cyclised *trans* product **2l** of 2.4:1. Purification by column chromatography (SiO_2: acetone/pentane, 0–15%) afforded **3l** (51 mg, 71%) as a white amorphous solid.

^{1}H NMR (400 MHz, $CDCl_3$) δ = 7.37 – 7.28 (m, 1H), 7.25 – 7.19 (m, 3H), 6.96 – 6.94 (m, 1H), 6.87 (dd, *J*=8.7, 2.7, 1H), 6.41 (d, *J*=8.7, 1H), 4.91 (d, *J*=6.6, 1H), 3.77 (s, 3H), 3.66 (d, *J*=4.3, 1H), 3.18 (dddd, *J*=8.4, 7.4, 6.6, 4.3, 1H), 3.02 (dd, *J*=15.7, 7.4, 1H), 2.75 (dd, *J*=15.7, 8.4, 1H).

^{19}F NMR (376 MHz, $CDCl_3$) δ = -58.5.

^{13}C NMR (101 MHz, $CDCl_3$) δ = 173.5, 145.2, 142.8, 142.0, 140.1, 128.4, 127.2, 125.5, 123.8, 122.9, 121.6, 116.6, 114.3, 56.9, 52.5, 45.3, 39.9, 36.3.

FT-IR (thin film) ν_{max} (cm^{-1}) 3396, 2953, 1728, 1507.

HRMS (ESI) *m/z* calcd for $C_{19}H_{16}NO_3F_3+H^+$: 364.1155 $[M+H]^+$; found: 364.1151.

m.p. (from $CDCl_3$) 88-90 °C

Synthesis of 3m

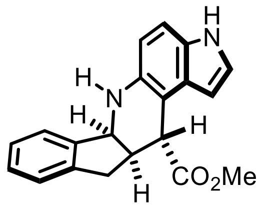

Compound **3m** was synthesised according to General Procedure D using aldehyde **6** (40.8 mg, 0.2 mmol) and 1*H*-indol-5-amine (26.4 mg, 0.2 mmol). ^{1}H NMR analysis of the crude reaction mixture revealed a ratio of doubly-cyclised *cis*-fused product **3m** to singly-cyclised *trans* product **2m** of 4.6:1. Purification by column chromatography (SiO_2: acetone/pentane, 0–25%) afforded **3m** (47 mg, 74%) as a colourless crystalline solid.

^{1}H NMR (400 MHz, $CDCl_3$) δ = 8.49 (s, 1H), 7.41 – 7.37 (m, 1H), 7.30 – 7.20 (m, 3H), 7.03 (dd, *J*=3.2, 2.3, 1H), 6.41 (dd, *J*=3.2, 2.0, 1H), 6.32 (d, *J*=8.4, 1H), 4.95 (d, *J*=5.5, 1H), 4.03 (d, *J*=1.7, 1H), 3.73 (s, 4H), 3.15 (dddd, *J*=10.8, 7.3, 5.5, 1.7, 1H), 2.99 – 2.75 (m, 3H).

^{13}C NMR (101 MHz, $CDCl_3$) δ = 174.0, 145.9, 143.1, 140.0, 136.8, 128.3, 127.0, 125.3, 123.6, 121.7, 120.9, 120.0, 109.9, 103.2, 95.2, 55.4, 52.6, 41.3, 40.3, 36.3.

FT-IR (thin film) ν_{max} (cm^{-1}) 3405, 2949, 1720, 1623, 1510.

HRMS (ESI) *m/z* calcd for $C_{20}H_{18}N_2O_2$+H^+: 319.1441 [*M*+H]$^+$; found: 319.1439.

m.p. (from $CDCl_3$) 103-106 °C

Synthesis of 3n

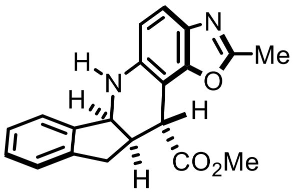

Compound **3n** was synthesised according to General Procedure D using aldehyde **6** (40.8 mg, 0.2 mmol) and 2-methylbenzo[*d*]oxazol-6-amine (30 mg, 0.2 mmol). ^{1}H NMR analysis of the crude reaction mixture revealed a ratio of doubly-cyclised *cis*-fused product **3n** to singly-cyclised *trans* product **2n** of 2.5:1. Purification by column chromatography (SiO_2: acetone/pentane, 0–15%) afforded **3n** (42 mg, 63%) as a colourless crystalline solid.

^{1}H NMR (600 MHz, $CDCl_3$) δ = 7.35 – 7.30 (m, 1H), 7.26 – 7.18 (m, 4H), 6.41 (d, *J*=8.5, 1H), 4.89 (d, *J*=6.4, 1H), 4.02 (d, *J*=3.8, 1H), 3.97 (br s, 1H), 3.76 (s, 3H), 3.28 – 3.20 (m, 1H), 3.03 (dd, *J*=15.5, 7.3, 1H), 2.79 (dd, *J*=15.5, 8.4, 1H), 2.56 (s, 3H).

^{13}C NMR (151 MHz, $CDCl_3$) δ = 173.7, 161.0, 151.0, 145.3, 142.2, 141.9, 132.5, 128.4, 127.2, 125.5, 123.7, 118.6, 111.2, 98.9, 56.8, 52.6, 40.3, 40.2, 36.3, 14.6, 1.2.

FT-IR (thin film) ν_{max} (cm^{-1}) 3386, 2951, 1733, 1617, 1505.

HRMS (ESI) *m/z* calcd for $C_{20}H_{18}N_2O_3+H^+$: 335.1390 $[M+H]^+$; found: 335.1390.

m.p. (from $CDCl_3$) 161-162 °C

Synthesis of 4-aldehyde

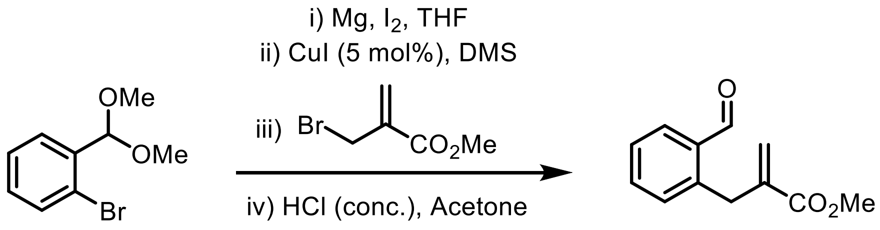

4-aldehyde, 67%

Methyl 2-(bromomethyl)acrylate was prepared according to a literature method.[10]

Freshly-burnished magnesium turnings (60.7 mg, 2.5 mmol) and iodine crystals (2 mg) were degassed and vigorously stirred for 10 min. A solution of 1-bromo-2-(dimethoxymethyl)benzene (510 mg, 2.2 mmol) in anhydrous THF (2 mL, 1 mol L^{-1}) was added dropwise and warmed to a gentle reflux. The mixture was stirred for 1 hour at r.t., before being cooled to –78 °C and diluted with THF (4 mL, 0.33 mol L^{-1}). A solution of copper (I) iodide (21.4 mg, 0.11 mmol) in dimethylsulfide (0.2 mL) and diethyl ether (0.2 mL) was added dropwise to the mixture, and stirred for 10 minutes. A solution of methyl 2-(bromomethyl)acrylate (626 mg, 3.5 mmol) in THF (1 mL) was pre-cooled, and then added swiftly in one portion to the reaction mixture. The mixture was slowly warmed to r.t. and stirred for 16 hours. The reaction mixture was then quenched with saturated aqueous NH_4Cl solution (20 mL) and extracted with EtOAc (2x 10 mL). The organic phase was washed with sat. aq. NH_4Cl (2x 5 mL), water (2x 5 mL) and brine (5 mL). The combined organic phases were dried over $MgSO_4$, filtered and concentrated under reduced pressure, affording methyl 2-(2-(dimethoxymethyl)benzyl)acrylate as a yellow oil. The crude yellow oil was dissolved in acetone (10 mL) and conc. HCl (37 µL, 0.44 mmol) was added. After 10 minutes, the mixture was quenched by the addition of aqueous Na_2CO_3 (5 mL, 1 mol L^{-1}) and the acetone was removed under reduced pressure. The residues were extracted with EtOAc (3x 5 mL), and the combined organic phases were washed with brine (5 mL), then dried over $MgSO_4$, filtered and concentrated under reduced pressure. The crude material was purified by column chromatography (SiO_2: EtOAc/pentane, 0–10%), affording **4-aldehyde** (341 mg, 67%) as a colourless oil.

^{1}H NMR (400 MHz, $CDCl_3$) δ = 10.21 (s, 1H), 7.87 (dd, *J*=7.7, 1.5, 1H), 7.54 (ddd, *J*=7.8, 7.5, 1.5, 1H), 7.43 (ddd, *J*=7.7, 7.5, 1.2, 1H), 7.29 (dd, *J*=7.8, 1.2, 1H), 6.25 (dt, *J*=1.5, 1.1, 1H), 5.27 (dd, *J*=1.5, 1.1, 1H), 4.07 (t, *J*=1.5, 2H), 3.77 (s, 3H).

^{13}C NMR (101 MHz, $CDCl_3$) δ = 192.1, 167.1, 140.7, 139.8, 134.1, 133.9, 131.9, 131.6, 127.3, 126.6, 52.0, 34.1.

FT-IR (thin film) ν_{max} (cm^{-1}) 2952, 1717, 1697.

HRMS (ESI) *m/z* calcd for $C_{12}H_{12}O_3$+H^+: 205.0859 [*M*+H]$^+$; found: 205.0859.

Synthesis of **5**

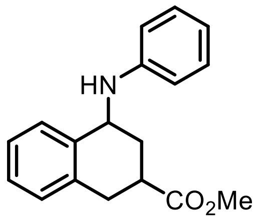

Imine **4** was pre-formed by stirring **4-aldehyde** (40.8 mg, 0.2 mmol) and aniline (18 µL, 0.2 mmol) with $MgSO_4$ (100 mg) in anhydrous CH_2Cl_2 (1 mL, 0.2 mol L^{-1}) for 16 hours. The mixture was filtered and transferred to an 8 mL Wheaton vial equipped with stir bar. The solvent was evaporated by passing a stream of N_2 across its surface for 5 minutes. **HE1** (unsubstituted Hantzsch ester, 74 mg, 0.3 mmol, 1.5 eq.) and [Ir(dF(CF_3)ppy)$_2$(dtbbpy)]PF_6 (2.2 mg, 0.002 mmol, 1 mol%) were added, and the solids were degassed by repeated evacuation and refilling with N_2 *via* the septum. Anhydrous degassed *N*,*N*-dimethylacetamide (1 mL, 0.2 mol L^{-1}) was added *via* the septum, and the mixture was placed in a Sigma Aldrich SynLED 467.5 nm 12 W LED 16-well array and irradiated for 4 h. The reaction mixture was diluted with ethyl acetate (20 mL) and the organic phase was washed with brine (4 x 10 mL). The organic phase was dried over $MgSO_4$, filtered, and concentrated under reduced pressure. The crude material was purified by column chromatography (SiO_2: acetone/pentane, 0–15%), affording **5** (56 mg, 99%) as a white amorphous solid, a 1.4:1 inseparable mixture of diastereoisomers. Note: treatment of the mixture with sodium hydride did not illicit lactamisation of either diastereoisomer (cf. **2b-*cis***).

^{1}H NMR (400 MHz, $CDCl_3$) δ = 7.56 (dd, *J*=6.9, 2.2, 1H, minor), 7.36 (dd, *J*=7.3, 2.2, 1H, major), 7.22 (m, 10H, major & minor), 6.83 – 6.74 (m, 2H, major & minor), 6.70 (dtt, *J*=8.6, 3.5, 1.8, 4H, major & minor), 4.77 (dd, *J*=10.2, 5.5, 1H, minor), 4.73 (dd, *J*=3.6, 1H, major), 3.72 (s, 3H, major), 3.70 (s, 3H, minor), 3.21 – 2.88 (m, 6H, major & minor), 2.67 – 2.61 (m, 1H minor), 2.55 – 2.47 (m, 1H, major), 1.97 (ddt, *J*=13.1, 7.7, 3.6, 1H, major), 1.86 (ddd, *J*=12.9, 11.2, 10.2, 1H, minor).

^{13}C NMR (101 MHz, $CDCl_3$) δ = 175.9, 175.2, 147.6, 146.9, 137.9, 136.6, 135.6, 135.3, 130.1, 129.5, 129.5, 129.4, 129.2, 129.1, 127.9, 127.5, 127.5, 126.8, 126.7, 118.8, 117.7, 117.6, 115.3, 113.2, 112.9, 52.1, 51.9, 50.4, 38.9, 35.7, 33.1, 31.7, 31.7, 30.6.

FT-IR (thin film) ν_{max} (cm^{-1}) 3396 (w), 2951 (w), 1727 (s), 1600 (s), 1502.

HRMS (ESI) *m/z* calcd for $C_{18}H_{19}NO_2+H^+$: 282.1489 [*M*+H]$^+$; found: 282.1486.

m.p. (from $CDCl_3$) 70-76 °C

Synthesis of **6**

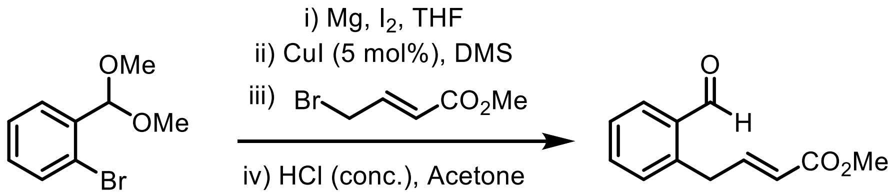

6, 74%

Freshly-burnished magnesium turnings (607 mg, 25 mmol) and iodine crystals (5 mg) were degassed and vigorously stirred for 10 min. A solution of 1-bromo-2-(dimethoxymethyl)benzene (5.1 g, 22 mmol) in anhydrous THF (22 mL, 1 mol L^{-1}) was added dropwise over 10 min, causing self-sustained heating. The mixture was stirred for 1 hour at r.t., before being cooled to –78 °C and diluted with THF (44 mL, 0.33 mol L^{-1}). A solution of copper (I) iodide (214 mg, 1.1 mmol) in dimethylsulfide (1.7 mL) and diethyl ether (1.7 mL) was added dropwise to the mixture, and stirred for 10 minutes. A solution of methyl 4-bromocrotonate (4.1 mL, 35 mmol) in THF (10 mL) was pre-cooled, and then added swiftly in one portion to the reaction mixture. The mixture was allowed to warm slowly to r.t. over 16 hours. The reaction mixture was then quenched with saturated aqueous NH_4Cl solution (200 mL) and extracted with EtOAc (2x 100 mL). The organic phase was washed with sat. aq. NH_4Cl (2x 20 mL), water (2x 20 mL) and brine (20 mL). The combined organic phases were dried over $MgSO_4$, filtered and concentrated under reduced pressure to afford methyl (*E*)-4-(2-(dimethoxymethyl)phenyl)but-2-enoate as a yellow oil. This material was dissolved in acetone (100 mL) and conc. HCl (0.37 mL, 4.4 mmol) was added in a single portion. After 10 minutes, the mixture was quenched by the addition of aqueous Na_2CO_3 (50 mL, 1 mol L^{-1}) and the acetone was removed under reduced pressure. The residues were extracted with EtOAc (3x 50 mL), and the combined organic phases were washed with brine (50 mL), then dried over $MgSO_4$, filtered and concentrated under reduced pressure. The crude material was purified by column chromatography (SiO_2: EtOAc/pentane, 0–10%), affording **6** (3.32 g, 74%) as a colourless oil. The spectroscopic data were in agreement with the literature.[11]

^{1}H NMR (400 MHz, $CDCl_3$) δ = 10.15 (s, 1H), 7.84 (dd, *J*=7.6, 1.6, 1H), 7.55 (td, *J*=7.5, 1.6, 1H), 7.46 (td, *J*=7.5, 1.3, 1H), 7.27 (d, *J*=7.7, 1H), 7.14 (dt, *J*=15.6, 6.4, 1H), 5.73 (dt, *J*=15.6, 1.8, 1H), 3.98 (dd, *J*=6.4, 1.8, 3H), 3.70 (s, 3H).

^{13}C NMR (101 MHz, $CDCl_3$) δ = 192.7, 166.9, 147.1, 139.8, 134.2, 134.0, 131.6, 127.7, 122.4, 51.6, 35.4.

MS (EI) m/z (%): 203 (100) [M–H]$^-$

Synthesis of 7

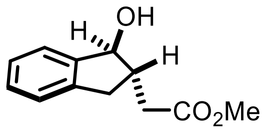

To an 8 mL Wheaton vial equipped with stir bar and rubber septum were charged aldehyde **6** (40.8 mg, 0.2 mmol, 1 eq.), **HE1** (unsubstituted Hantzsch ester, 74 mg, 0.3 mmol, 1.5 eq) and [Ir(dF(CF_3)ppy)$_2$(dtbbpy)]PF_6 (2.2 mg, 0.002 mmol, 1 mol%). The solids were degassed by repeated evacuation and refilling with N_2 *via* the septum. Anhydrous degassed *N*,*N*-dimethylacetamide (1 mL, 0.2 mol L^{-1}) was added *via* the septum, and the vial was placed in a Sigma Aldrich SynLED 467.5 nm 12 W LED 16-well array and irradiated for 4 h. The reaction mixture was diluted with ethyl acetate (20 mL) and the organic phase was washed with brine (5x 5 mL). The organic phase was dried over $MgSO_4$, filtered, and concentrated under reduced pressure. The crude material (9:1 *trans*:*cis*) was purified by column chromatography (SiO_2: acetone/pentane, 15–30%), affording **7** (38 mg, 92%) as a single diastereomer, a colourless crystalline solid. Melting point and IR data were in accordance with a previous report.[13] The assigned stereochemistry was confirmed as detailed in Section 7.

^{1}H NMR (400 MHz, $CDCl_3$) δ = 7.44 – 7.36 (m, 1H), 7.30 – 7.14 (m, 3H), 4.99 – 4.92 (m, 1H), 3.73 (s, 3H), 3.22 – 3.14 (m, 2H), 2.77 – 2.64 (m, 2H), 2.63 – 2.52 (m, 2H).

^{13}C NMR (101 MHz, $CDCl_3$) δ = 174.4, 144.4, 140.8, 128.3, 127.1, 124.6, 124.1, 81.1, 52.0, 46.6, 37.9, 36.4.

FT-IR (thin film) ν_{max} (cm^{-1}) 3442 (br), 2953 (w), 1730 (s).

HRMS (ESI) *m/z* calcd for $C_{12}H_{14}O_3$+Na^+: 229.0835 [*M*+Na]$^+$; found: 229.0836.

m.p. (from MeOH) 99-100 °C

Synthesis of 9a

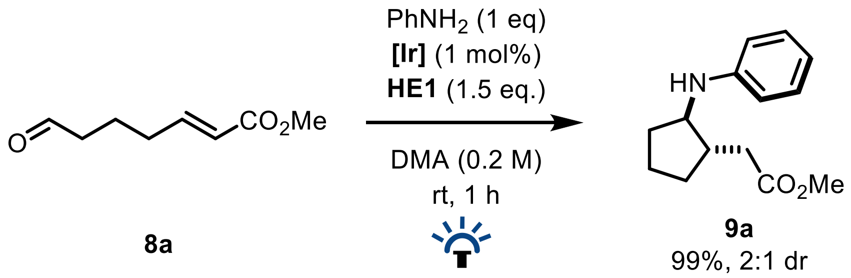

Aliphatic aldehyde **8a** was prepared as a single isomer according to a literature method.[12]

To an 8 mL Wheaton vial equipped with stir bar and rubber septum were charged aldehyde **8a** (31 mg, 0.2 mmol, 1 eq.), aniline (18 µL, 0.2 mmol, 1 eq.), **HE1** (unsubstituted Hantzsch ester, 74 mg, 0.3 mmol, 1.5 eq.) and [Ir(dF(CF$_3$)ppy)$_2$(dtbbpy)]PF$_6$ (2.2 mg, 0.002 mmol, 1 mol%). The solids were degassed by repeated evacuation and refilling with N_2 *via* the septum. Anhydrous degassed *N*,*N*-dimethylacetamide (1 mL, 0.2 mol L^{-1}) was added *via* the septum, and the mixture was stirred 15 minutes in darkness (in order to facilitate imine formation). The vial was placed in a Sigma Aldrich SynLED 467.5 nm 12 W LED 16-well array and irradiated for 1 h. The reaction mixture was diluted with ethyl acetate (20 mL) and the organic phase was washed with brine (2x 5 mL). The organic phase was dried over $MgSO_4$, filtered, and concentrated under reduced pressure. The crude material was purified by column chromatography (SiO_2: acetone/pentane, 5–15%), affording **9a-*trans*** (31 mg) as a colourless oil and **9a-*cis*** (15 mg) as a colourless oil in a combined yield of 99%, 2.0:1 dr. Note: on standing as an oil, **9a-*cis*** underwent lactamisation to afford **9a-*cis*-lactam**.

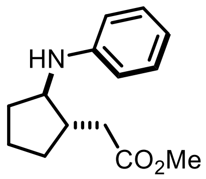

9a-*trans*

^{1}H NMR (400 MHz, $CDCl_3$) δ = 7.20 – 7.10 (m, 2H), 6.67 (tt, *J*=7.4, 1.1, 1H), 6.62 – 6.54 (m, 2H), 3.79 (s, 1H), 3.64 (s, 3H), 3.39 (q, *J*=7.3, 1H), 2.58 (dd, *J*=15.5, 6.2, 1H), 2.35 (dd, *J*=15.5, 7.9, 1H), 2.24 – 2.08 (m, 1H), 2.06 – 1.93 (m, 1H), 1.79 – 1.65 (m, 2H), 1.47 – 1.38 (m, 1H), 1.37 – 1.29 (m, 1H).

^{13}C NMR (101 MHz, $CDCl_3$) δ = 173.8, 148.3, 129.4, 117.2, 113.2, 60.3, 51.7, 43.6, 38.5, 32.9, 30.6, 22.4.

FT-IR (thin film) ν_{max} (cm^{-1}) 2954, 1687, 1597, 1386.

HRMS (ESI) *m/z* calcd for $C_{14}H_{19}NO_2+H^+$: 234.1489 [*M*+H]$^+$; found: 234.1488.

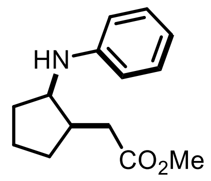

9a-*cis*

^{1}H NMR (400 MHz, $CDCl_3$) δ = 7.20 – 7.07 (m, 2H), 6.67 (tt, *J*=7.3, 1.1, 1H), 6.59 (dt, *J*=7.7, 1.1, 2H), 3.90 – 3.82 (m, 1H), 3.60 (s, 1H), 3.54 (s, 3H), 2.71 – 2.56 (m, 1H), 2.47 (dd, *J*=15.6, 6.6, 1H), 2.21 (dd, *J*=15.6, 8.4, 1H), 2.10 – 1.86 (m, 2H), 1.82 – 1.69 (m, 1H), 1.68 – 1.42 (m, 3H).

^{13}C NMR (101 MHz, $CDCl_3$) δ = 173.8, 147.8, 129.2, 117.1, 112.9, 56.6, 51.4, 38.6, 34.5, 32.1, 29.9, 21.2.

FT-IR (thin film) ν_{max} (cm^{-1}) 3393, 2952, 1731, 1601, 1505.

HRMS (ESI) *m/z* calcd for $C_{14}H_{19}NO_2+H^+$: 234.1489 $[M+H]^+$; found: 234.1490.

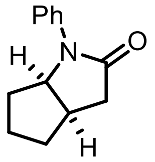

9a-*cis*-lactam

Data were in accordance with the literature.[14]

^{1}H NMR (400 MHz, $CDCl_3$) δ = 7.54 – 7.46 (m, 2H), 7.42 – 7.33 (m, 2H), 7.22 – 7.13 (m, 1H), 4.64 (ddd, *J*=7.8, 5.1, 3.5, 1H), 2.94 – 2.75 (m, 2H), 2.41 – 2.32 (m, 1H), 2.01 – 1.83 (m, 1H), 1.76 – 1.69 (m, 2H), 1.69 – 1.61 (m, 2H), 1.60 – 1.52 (m, 1H).

^{13}C NMR (101 MHz, $CDCl_3$) δ = 174.2, 138.4, 129.0, 125.3, 122.8, 65.2, 39.5, 34.4, 34.3, 32.4, 24.2.

MS (EI) m/z (%): 202 (74) $[M+H]^+$, 224 (100) $[M+Na]^+$

Synthesis of **9b**

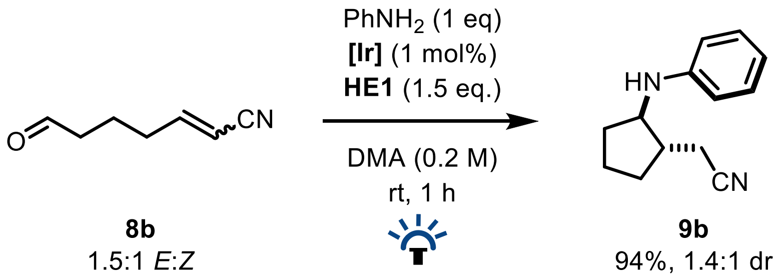

8b
1.5:1 *E*:*Z*

9b
94%, 1.4:1 dr

Aliphatic aldehyde **8b** was prepared as an inseparable mixture of *E*:*Z* isomers (1.5:1) according to a literature method.[12]

To an 8 mL Wheaton vial equipped with stir bar and rubber septum were charged aldehyde **8b** (25 mg, 0.2 mmol, 1 eq.), aniline (18 µL, 0.2 mmol, 1 eq.), **HE1** (unsubstituted Hantzsch ester, 74 mg, 0.3 mmol, 1.5 eq.) and [Ir(dF(CF_3)ppy)$_2$(dtbbpy)]PF_6 (2.2 mg, 0.002 mmol, 1 mol%). The solids were degassed by repeated evacuation and refilling with N_2 *via* the septum. Anhydrous degassed *N*,*N*-dimethylacetamide (1 mL, 0.2 mol L^{-1}) was added *via* the septum, and the mixture was stirred 15 minutes in darkness (in order to facilitate imine formation). The vial was placed in a Sigma Aldrich SynLED 467.5 nm 12 W LED 16-well array and irradiated for 1 h. The reaction mixture was diluted with ethyl acetate (20 mL) and the organic phase was washed with brine (2x 5 mL). The organic phase was dried over $MgSO_4$, filtered, and concentrated under reduced pressure. The crude material was purified by column chromatography (SiO_2: acetone/pentane, 5–15%), affording **9b-*trans*** (22 mg) as a colourless oil and **9b-*cis*** (15 mg) as a colourless oil in a combined yield of 94%, 1.4:1 dr. [Note: the observed diastereomeric ratio is presumed to originate from the inherent reactivity of the intermediate radical species, as with example **9a**, rather than from the mixed *E*:*Z* configuration of starting material **8b**.]

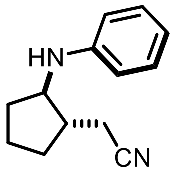

9b-*trans*

^{1}H NMR (400 MHz, $CDCl_3$) δ = 7.24 – 7.13 (m, 2H), 6.74 (tt, *J*=7.3, 1.1, 1H), 6.69 – 6.59 (m, 2H), 3.56 (q, *J*=7.2, 1H), 2.60 (dd, *J*=16.8, 4.8, 1H), 2.45 (dd, *J*=16.8, 7.0, 1H), 2.28 – 2.15 (m, 1H), 2.14 – 1.96 (m, 2H), 1.86 – 1.70 (m, 2H), 1.60 – 1.40 (m, 2H).

^{13}C NMR (101 MHz, $CDCl_3$) δ = 147.3, 129.6, 118.9, 118.3, 113.8, 59.6, 43.4, 32.6, 29.8, 22.1, 21.1.

FT-IR (thin film) ν_{max} (cm^{-1}) 3388, 2957, 1602, 1504.

HRMS (ESI) *m/z* calcd for $C_{13}H_{16}N_2$+H^+: 201.1386 [*M*+H]$^+$; found: 201.1384.

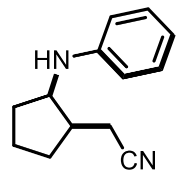

9b-*cis*

^{1}H NMR (400 MHz, $CDCl_3$) δ = 7.25 – 7.13 (m, 2H), 6.77 – 6.68 (m, 1H), 6.64 – 6.56 (m, 2H), 3.85 (q, *J*=7.2, 1H), 3.66 (s, 1H), 2.63 – 2.49 (m, 1H), 2.39 (dd, *J*=16.8, 5.0, 1H), 2.23 (dd, *J*=16.8, 8.8, 1H), 2.18 – 2.02 (m, 2H), 1.89 – 1.77 (m, 1H), 1.76 – 1.52 (m, 3H).

^{13}C NMR (101 MHz, $CDCl_3$) δ = 147.4, 129.6, 120.2, 117.9, 112.9, 56.8, 38.4, 32.6, 30.2, 21.7, 18.2.

FT-IR (thin film) ν_{max} (cm^{-1}) 3384, 2957, 1601, 1498.

HRMS (ESI) *m/z* calcd for $C_{13}H_{16}N_2$+H^+: 201.1386 [*M*+H]$^+$; found: 201.1387.

Synthesis of **S1**

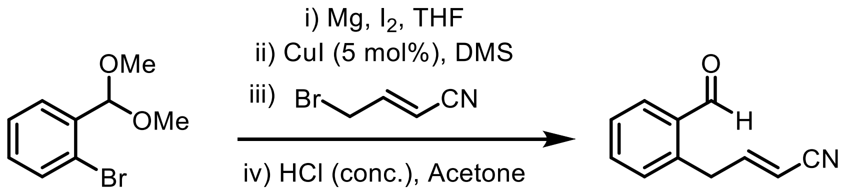

S1, 52%

(*E*)-4-bromo-2-butenenitrile was prepared according to a literature method.[15]

Freshly-burnished magnesium turnings (58 mg, 2.4 mmol) and iodine crystals (1 mg) were degassed and vigorously stirred for 10 min. A solution of 1-bromo-2-(dimethoxymethyl)benzene (513 mg, 2.2 mmol) in anhydrous THF (2.2 mL, 1 mol L^{-1}) was added dropwise over 10 min, causing self-sustained heating. The mixture was stirred for 1 hour at r.t., before being cooled to –78 °C and diluted with THF (4.4 mL, 0.33 mol L^{-1}). A solution of copper (I) iodide (21.4 mg, 0.11 mmol) in dimethylsulfide (0.25 mL) and diethyl ether (0.25 mL) was added dropwise to the mixture, and stirred for 10 minutes. A solution of (*E*)-4-bromo-2-butenenitrile (511 mg, 3.5 mmol) in THF (1 mL) was pre-cooled, and then added swiftly in one portion to the reaction mixture. The mixture was allowed to warm slowly to r.t. over 16 hours. The reaction mixture was then quenched with saturated aqueous NH_4Cl solution (20 mL) and extracted with EtOAc (2x 10 mL). The organic phase was washed with sat. aq. NH_4Cl (5 mL), water (2x 5 mL) and brine (5 mL). The combined organic phases were dried over $MgSO_4$, filtered and concentrated under reduced pressure to afford (*E*)-4-(2-(dimethoxymethyl)phenyl)but-2-enenitrile as a yellow oil. This material was dissolved in acetone (10 mL) and conc. HCl (37 µL, 0.4 mmol) was added in a single portion. After 10 minutes, the mixture was quenched by the addition of aqueous Na_2CO_3 (5 mL, 1 mol L^{-1}) and the acetone was removed under reduced pressure. The residues were extracted with EtOAc (3x 5 mL), and the combined organic phases were washed with brine (5 mL), then dried over $MgSO_4$, filtered and concentrated under reduced pressure. The crude material was purified by column chromatography (SiO_2: EtOAc/pentane, 0–10%), affording **S1** (196 mg, 52%) as a colourless oil.

^{1}H NMR (400 MHz, $CDCl_3$) δ = 10.08 (s, 1H), 7.83 (dd, *J*=7.4, 1.6, 1H), 7.62 – 7.47 (m, 2H), 7.29 – 7.22 (m, 1H), 6.89 (dt, *J*=16.3, 6.4, 1H), 5.24 (dt, *J*=16.3, 1.9, 1H), 3.97 (dd, *J*=6.4, 1.9, 2H).

^{13}C NMR (101 MHz, $CDCl_3$) δ = 192.9, 153.4, 137.9, 135.3, 134.2, 133.8, 131.6, 128.0, 117.3, 100.9, 36.6.

FT-IR (thin film) ν_{max} (cm^{-1}) 2921, 2360, 2342, 1697.

HRMS (ESI) *m/z* calcd for $C_{11}H_9NO_2+H^+$: 188.0706 $[M+H]^+$; found: 188.0708.

Synthesis of S3

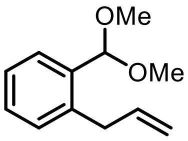

S2

Freshly-burnished magnesium turnings (4.2 g, 176 mmol) and iodine crystals (15 mg) were degassed and vigorously stirred for 10 min. A solution of 1-bromo-2-(dimethoxymethyl)benzene (37 g, 160 mmol) in anhydrous THF (100 mL) was added dropwise over 15 min, causing self-sustaining heating. The mixture was stirred for 30 min at r.t., and then cooled to –78 °C. Allyl bromide (19.4 g, 160 mmol) was added dropwise over 15 min. The mixture was allowed to warm to r.t. overnight, and was then quenched with saturated aqueous NH_4Cl solution (3 mL), stirring for 5 min. The product was extracted by washing the solid precipitate with EtOAc (2x 100 mL). The organic phase was washed with water (2x 50 mL) and brine (50 mL). The combined organic phases were dried over $MgSO_4$, filtered and concentrated under reduced pressure. The crude material was distilled (67 °C, 0.5 mbar) affording **S2** (21.3 g, 69%) as a colourless oil.

^{1}H NMR (400 MHz, $CDCl_3$) δ = 7.56 (dd, *J*=7.4, 1.7, 1H), 7.33 – 7.16 (m, 3H), 6.05 – 5.91 (m, 1H), 5.49 (s, 1H), 5.15 – 4.96 (m, 2H), 3.50 (dt, *J*=6.4, 1.7, 2H), 3.32 (s, 6H).

^{13}C NMR (101 MHz, $CDCl_3$) δ = 138.1, 137.4, 135.7, 130.1, 128.8, 126.9, 126.1, 115.8, 101.7, 53.3, 36.5.

FT-IR (thin film) ν_{max} (cm^{-1}) 2933, 1451.

HRMS (ESI) *m/z* mass not found.

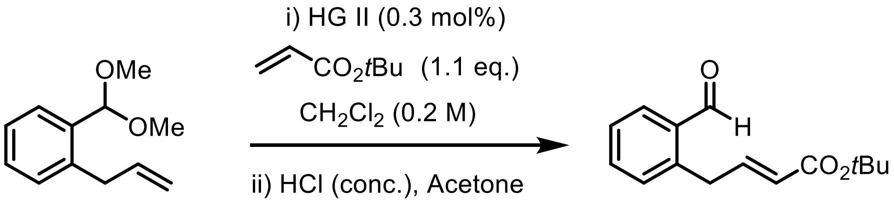

Acetal **S2** (960 mg, 5 mmol) and *tert*-butyl acrylate (0.8 mL, 5.5 mmol) were dissolved in anh. degassed DCM (30 mL) under Ar and warmed to 30 °C. Hoveyda-Grubbs 2nd Generation catalyst (10 mg, 0.3 mol%) was dissolved in anh. degassed toluene (1 mL) and added dropwise to the reaction over 1 hour. The DCM was evaporated under reduced pressure, and then the crude residue was dissolved in acetone (20 mL) and conc. HCl (80 µL, 1 mmol) was added in a single portion. After 10 minutes, the mixture was quenched by the addition of aqueous Na_2CO_3 (10 mL, 1 mol L^{-1}) and the acetone was removed under reduced pressure. The residues were extracted with EtOAc (3x 10 mL), and the combined organic phases were washed with brine (10 mL), then dried over $MgSO_4$, filtered and concentrated under reduced pressure. Purification by column chromatography (SiO_2: EtOAc/pentane, 0–10%) afforded **S3** (1.04 g, 85%) as a colourless oil.

^{1}H NMR (400 MHz, $CDCl_3$) δ = 10.17 (s, 1H), 7.84 (dd, *J*=7.7, 1.5, 1H), 7.55 (ddd, *J*=7.9, 7.5, 1.5, 1H), 7.45 (ddd, *J*=7.7, 7.5, 1.3, 1H), 7.27 (dd, *J*=7.9, 1.3, 1H), 7.02 (dt, *J*=15.6, 6.4, 1H), 5.63 (dt, *J*=15.6, 1.7, 1H), 3.95 (dd, *J*=6.4, 1.7, 2H), 1.45 (s, 9H).

^{13}C NMR (101 MHz, $CDCl_3$) δ = 192.4, 165.7, 145.3, 140.1, 134.0, 133.9, 133.3, 131.5, 127.4, 124.5, 80.3, 35.0, 28.1.

FT-IR (thin film) ν_{max} (cm^{-1}) 2978, 1697.

HRMS (ESI) *m/z* mass not found.

Synthesis of **S4**

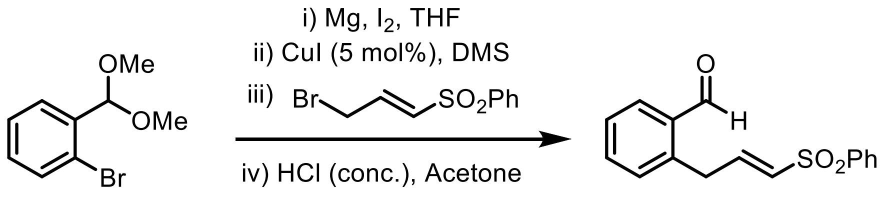

S4, 35%

(*E*)-((3-bromoprop-1-en-1-yl)sulfonyl)benzene was prepared according to a literature method.[15]

Freshly-burnished magnesium turnings (25 mg, 1.05 mmol) and iodine crystals (0.5 mg) were degassed and vigorously stirred for 10 min. A solution of 1-bromo-2-(dimethoxymethyl)benzene (231 mg, 1 mmol) in anhydrous THF (1 mL, 1 mol L^{-1}) was added dropwise over 10 min, causing self-sustained heating. The mixture was stirred for 1 hour at r.t., before being cooled to –78 °C and diluted with THF (2 mL, 0.33 mol L^{-1}). A solution of copper (I) iodide (9.5 mg, 0.05 mmol) in dimethylsulfide (0.2 mL) and diethyl ether (0.2 mL) was added dropwise to the mixture, and stirred for 10 minutes. A solution of (*E*)-((3-bromoprop-1-en-1-yl)sulfonyl)benzene (274 mg, 1.05 mmol) in THF (1 mL) was added swiftly in one portion to the reaction mixture. [Note: it is expected that a higher yield could be achieved using an increased amount of the bromide electrophile. Only 1.05 eq. were used herein due to a limited amount of material being available]. The mixture was allowed to warm slowly to r.t. over 16 hours. The reaction mixture was then quenched with saturated aqueous NH_4Cl solution (10 mL) and extracted with EtOAc (2x 5 mL). The organic phase was washed with sat. aq. NH_4Cl (3 mL), water (2x 3 mL) and brine (3 mL). The combined organic phases were dried over $MgSO_4$, filtered and concentrated under reduced pressure to afford (*E*)-1-(dimethoxymethyl)-2-(3-(phenylsulfonyl)allyl)benzene as a yellow amorphous solid. This material was dissolved in acetone (10 mL) and conc. HCl (28 µL, 0.3 mmol) was added in a single portion. After 10 minutes, the mixture was quenched by the addition of aqueous Na_2CO_3 (5 mL, 1 mol L^{-1}) and the acetone was removed under reduced pressure. The residues were extracted with EtOAc (3x 5 mL), and the combined organic phases were washed with brine (5 mL), then dried over $MgSO_4$, filtered and concentrated under reduced pressure. The crude material was purified by column chromatography (SiO_2: EtOAc/pentane, 20–40%), affording **S4** (100 mg, 35%) as a colourless crystalline solid.

^{1}H NMR (400 MHz, $CDCl_3$) δ = 10.02 (s, 1H), 7.87 – 7.74 (m, 3H), 7.63 – 7.43 (m, 5H), 7.23 (dd, *J*=7.5, 1.3, 1H), 7.15 (dt, *J*=15.1, 6.4, 1H), 6.21 (dt, *J*=15.1, 1.7, 1H), 4.00 (dd, *J*=6.4, 1.7, 2H).

^{13}C NMR (101 MHz, $CDCl_3$) δ = 192.8, 145.1, 140.5, 138.2, 135.2, 134.2, 133.8, 133.4, 131.7, 131.5, 129.3, 128.0, 127.6, 34.9.

FT-IR (thin film) ν_{max} (cm^{-1}) 3024, 2767, 1694, 1575, 1315, 1157.

HRMS (ESI) *m/z* calcd for $C_{16}H_{14}O_3S+H^+$: 287.0736 [*M*+H]$^+$; found: 287.0737.

m.p. (from $CDCl_3$) 121-123 °C

7. Mechanistic Investigations

i. Confirmation of Stereochemical Configuration and Observed Diastereoselectivity

Synthesis of 2b-*cis*

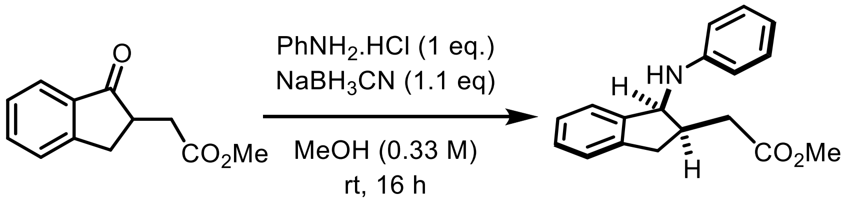

2b-*cis*
35%, 9:1 cis:trans

To aid in the initial assignment of single cyclisation products **2** as *trans*, and in order to verify the absence of the singly-cyclised *cis* diastereomer product under both the single and dual photo-cyclisation conditions, the *cis* diastereomer of **2b** was separately prepared.

S5 was first prepared from 1-indanone and methyl bromoacetate according to a literature method.[16] To a solution of **S5** (1.00 g, 4.9 mmol) in methanol (15 mL, 0.33 mol L^{-1}) was added aniline hydrochloride (635 mg, 4.9 mmol, 1 eq.), and the mixture was stirred at room temperature for 30 minutes. Sodium cyanoborohydride (339 mg, 5.4 mmol, 1.1 eq.) was added, and the reaction mixture stirred for a further 15 hours. The reaction mixture was poured into aqueous sodium carbonate solution (50 mL, 1 mol L^{-1}) and extracted with ethyl acetate (2x 50 mL). The combined organic extracts were washed with brine, dried over $MgSO_4$ and concentrated. Purification by column chromatography (SiO_2: acetone/pentane, 5–20%) afforded **2b** (481 mg, 35%, 9:1 *cis*:*trans*) as a colourless crystalline solid.

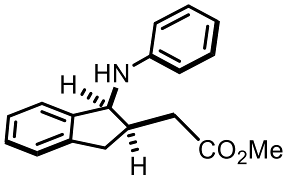

2b-*cis*

^{1}H NMR (400 MHz, $CDCl_3$) δ = 7.34 – 7.13 (m, 6H), 6.80 – 6.69 (m, 3H), 5.11 (d, *J*=6.7, 1H), 3.47 (s, 3H), 3.38 – 3.27 (m, 1H), 3.15 (dd, *J*=16.0, 7.3, 1H), 2.82 (dd, *J*=16.0, 3.6, 1H), 2.50 (dd, J=15.6, 6.3, 1H), 2.24 (dd, *J*=15.6, 8.5, 1H).

^{13}C NMR (101 MHz, $CDCl_3$) δ = 173.7, 147.4, 143.1, 141.4, 129.6, 128.2, 127.1, 125.3, 124.1, 117.9, 113.2, 60.6, 51.6, 40.2, 36.5, 34.4.

FT-IR (thin film) ν_{max} (cm^{-1}) 3392, 3024, 2948, 1728, 1600, 1500, 1435.

HRMS (ESI) *m/z* calcd for $C_{18}H_{19}O_2N+H^+$: 282.1489 [*M*+H]$^+$; found: 282.1489.

m.p. (from $CDCl_3$) 71-74 °C

Synthesis of 2b-*cis*-lactam

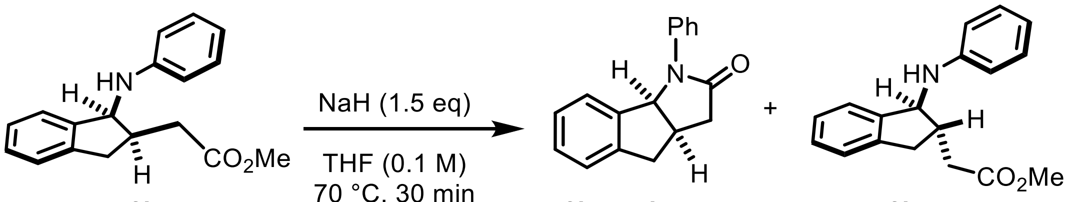

2b (46 mg, 0.16 mmol, 9:1 cis:trans) was dissolved in THF (1.6 mL, 0.1 mol L^{-1}) and sodium hydride (10 mg, 0.25 mmol, 60% dispersion in mineral oil) was added. The mixture was stirred at reflux for 30 minutes before being quenched by the addition of methanol (0.5 mL). The mixture was concentrated under reduced pressure, and purification by column chromatography (SiO_2: acetone/pentane, 5–10%) afforded **2b-*cis*-lactam** (36 mg, 88%) and unreacted **2b-*trans*** (4 mg, 9%).

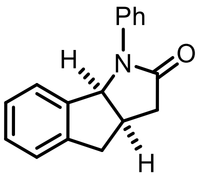

2b-*cis*-lactam

^{1}H NMR (400 MHz, $CDCl_3$) δ = 7.54 – 7.40 (m, 4H), 7.33 – 7.23 (m, 3H), 7.05 (td, *J*=7.7, 1.8, 1H), 6.89 (d, *J*=7.7, 1H), 5.67 (d, *J*=7.1, 1H), 3.45 – 3.31 (m, 2H), 3.04 – 2.90 (m, 2H), 2.53 (dd, *J*=17.5, 5.3, 1H).

^{13}C NMR (101 MHz, $CDCl_3$) δ = 173.4, 142.8, 140.4, 138.1, 129.2, 129.0, 126.8, 126.1, 125.5, 125.5, 124.5, 69.0, 39.0, 38.3, 35.1.

FT-IR (thin film) ν_{max} (cm^{-1}) 3067, 2951, 2932, 1681 (s), 1498.

HRMS (ESI) *m/z* calcd for $C_{17}H_{15}ON+H^+$: 250.1226 $[M+H]^+$; found: 250.1222.

m.p. (from $CDCl_3$) 128-131 °C

2b-*cis* is observable as only a trace impurity (<1%) in the photocyclisation reaction (Synthesis of **2b-*trans***), supporting the observed diastereoselectivity of >20:1 for all 1-aminoindane single cyclisation examples. Whilst the *cis*-fused double cyclisation product **3b** is observed under the photocyclisation conditions in the synthesis of **2b-*trans***, it was not observed under the reductive amination conditions above, supporting the posited radical-based mechanism for the formation of **3**. Lactamisation of **2b-*cis*** supports the assigned stereochemical configuration.

Synthesis of 7-*cis*-lactone

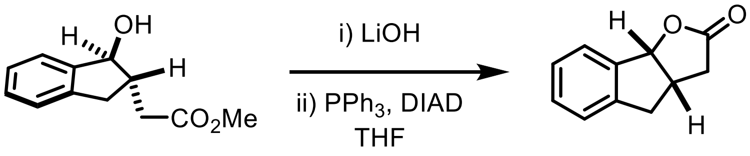

7 **7-*cis*-lactone** 82%

Alcohol **7** (20.6 mg, 0.1 mmol) was dissolved in THF (1 mL, 0.1 mol L^{-1}). A solution of LiOH (3.6 mg, 0.15 mmol, 1.5 eq.) in water (0.3 mL) was added, and the mixture stirred 30 min at rt. The mixture was diluted with CH_2Cl_2 (10 mL) and acidified with aqueous HCl solution (2 N, 5 mL). The aqueous phase was extracted with CH_2Cl_2 (3x 2 mL), and the combined organic extracts were dried over $MgSO_4$ and concentrated.

The resulting acid was dissolved in anh. THF (1 mL, 0.1 mol L^{-1}), and triphenylphosphine (28 mg, 0.1 mmol) was added. Diisopropyl azodicarboxylate (DIAD, 20 µL, 0.1 mmol) was added dropwise over 1 min, and the reaction was stirred 30 min at rt. The reaction mixture was concentrated under reduced pressure, and the crude mixture was purified by column chromatography (SiO_2: EtOAc/pentane, 20–30%), affording **7-*cis*-lactone** (14 mg, 82%) as a white amorphous solid. Spectral data were in accordance with the literature.[17] This result serves to support the assigned stereochemical configuration of **7** as *trans*.

^{1}H NMR (400 MHz, $CDCl_3$) δ = 7.49 (dd, *J*=7.4, 1.1, 1H), 7.39 – 7.26 (m, 3H), 5.89 (d, *J*=6.9, 1H), 3.42 – 3.27 (m, 2H), 2.96 – 2.85 (m, 2H), 2.44 – 2.35 (m, 1H).

^{13}C NMR (101 MHz, $CDCl_3$) δ = 177.0, 142.6, 138.9, 130.1, 127.7, 126.5, 125.5, 87.8, 38.1, 37.5, 35.8.

MS (EI) m/z (%): 197 (100) $[M+Na]^+$

NB. Treatment of alcohol **7** with sodium hydride in THF did not illicit lactonisation (cf. **2b-*cis*-lactam** above), further supporting the assigned *trans* configuration.

ii. HAT Termination Studies

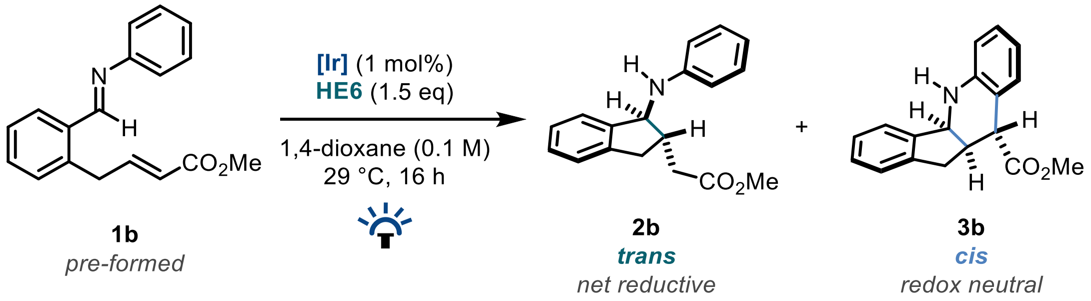

Imine **1b** was pre-formed by stirring aldehyde **6** (20.4 mg, 0.1 mmol) and aniline (9 µL, 0.1 mmol) with $MgSO_4$ (50 mg) in anhydrous CH_2Cl_2 (0.5 mL, 0.2 mol L^{-1}) for 16 hours. The mixture was filtered and transferred to a 1.6 mL glass vial equipped with stir bar. The solvent was evaporated by passing a stream of N_2 across its surface for 5 minutes. **HE6** (ethyl-substituted Hantzsch ester, 42 mg, 0.15 mmol, 1.5 eq.), $[Ir(dF(CF_3)ppy)_2(dtbbpy)]PF_6$ (1.1 mg, 0.001 mmol, 1 mol%) and anhydrous degassed 1,4-dioxane (1 mL, 0.1 mol L^{-1}) were added to the vial. 1,4-Cyclohexadiene (x eq., see table) was added, and N_2 was bubbled through the reaction mixture for 2 minutes. The vial was then sealed and suspended in a HepatoChem PhotoRedOx Box equipped with an EvoluChem™ 450PF 450 nm 18 W LED light source (Setup 1) and irradiated for 16 h. 1,3,5-Trimethoxybenzene (5.6 mg, 0.033 mmol) was then added to the reaction mixture, and an aliquot was taken for analysis by quantitative ^{1}H NMR spectroscopy by comparison of the C*H*NH peaks corresponding to **2b** (4.74 ppm) and **3b** (4.92 ppm).

Table S2: Termination mechanistic investigations with 1,4-cyclohexadiene as an HAT donor

Entry	1,4-CHD eq.	1,4-CHD volume / µL	2b (%)*	3b (%)*	2b:3b
1	0	0	20	76	1 : 3.8
2	1	9.5	40	60	1 : 1.5
3	3	28.5	47	53	1 : 1.1
4	5	47.5	65	35	1.9 : 1

*^{1}H NMR yields *versus* 1,3,5-trimethoxybenzene internal standard.

iii. Time-Course Experiments

Single Cyclisation (*Conditions A*)

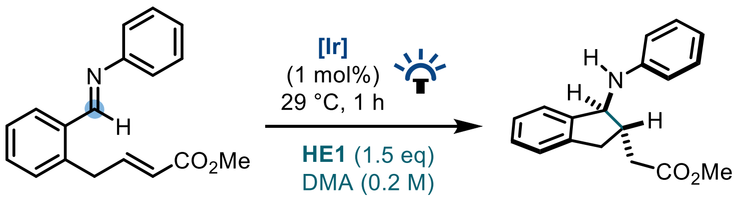

Imine **1a** was pre-formed by stirring aldehyde **6** (40.8 mg, 0.2 mmol) and aniline (18 µL, 0.2 mmol) with $MgSO_4$ (100 mg) in anhydrous 1,4-dioxane (1 mL, 0.2 mol L^{-1}) for 16 hours. The mixture was filtered and the solvent was evaporated under reduced pressure. The imine was dissolved in anhydrous DMA (1 mL, 0.2 mol L^{-1}) and transferred to a mass spectrometry vial equipped with stir bar and septum. Hantzsch ester (**HE1**, 74 mg, 0.3 mmol) and [Ir] (2.2 mg, 0.002 mmol, 1 mol%) were added to the vial, and Ar was bubbled through the reaction mixture for 2 minutes. The vial was then sealed and suspended in a HepatoChem PhotoRedOx Box equipped with an EvoluChem™ 450PF 450 nm 18 W LED light source (Setup 1) and irradiated. Aliquots of ~0.05 mL were taken for analysis by ^{1}H NMR spectroscopy at 4 minute intervals. The conversions indicated are based on comparison of the two product peaks at 4.60 ppm and 4.92 ppm with the starting material alkene peak at 5.60 ppm.

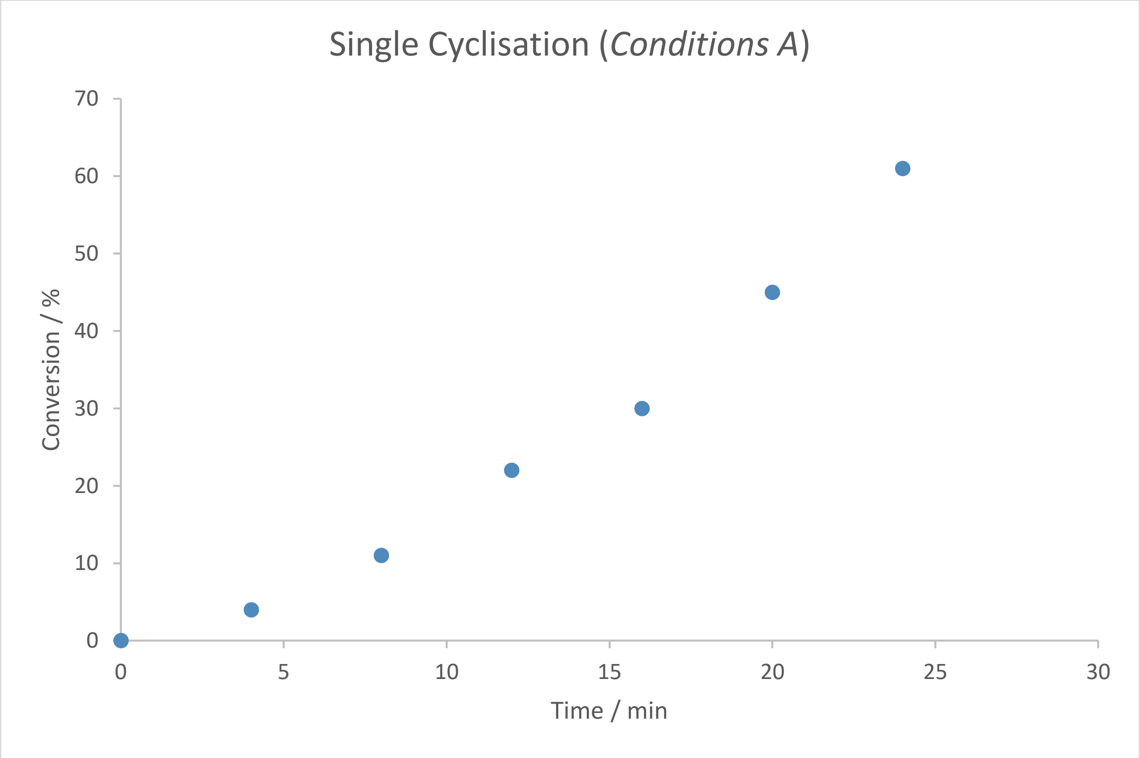

Figure S1: Single cyclisation time-course experiment

Double Cyclisation (*Conditions B*)

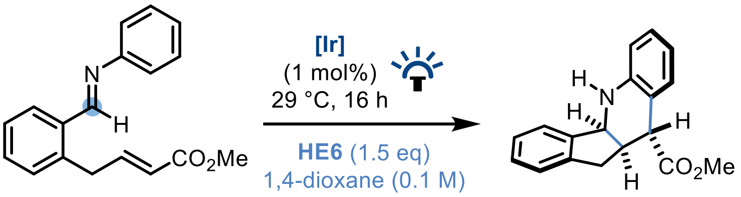

Imine **1a** was pre-formed by stirring aldehyde **6** (40.8 mg, 0.2 mmol) and aniline (18 µL, 0.2 mmol) with $MgSO_4$ (100 mg) in anhydrous 1,4-dioxane (1 mL, 0.2 mol L^{-1}) for 16 hours. The mixture was filtered and transferred to a mass spectrometry vial equipped with stir bar and septum. Hantzsch ester (**HE6**, 84 mg, 0.3 mmol) and [Ir] (2.2 mg, 0.002 mmol, 1 mol%) were added to the vial, and Ar was bubbled through the reaction mixture for 2 minutes. The vial was then sealed and suspended in a HepatoChem PhotoRedOx Box equipped with an EvoluChem™ 450PF 450 nm 18 W LED light source (Setup 1) and irradiated. Aliquots of ~0.05 mL were taken for analysis by ^{1}H NMR spectroscopy at 30 minute intervals. The conversions indicated are based on comparison of the two product peaks at 4.72 ppm and 4.91 ppm with the starting material alkene peak at 5.73 ppm.

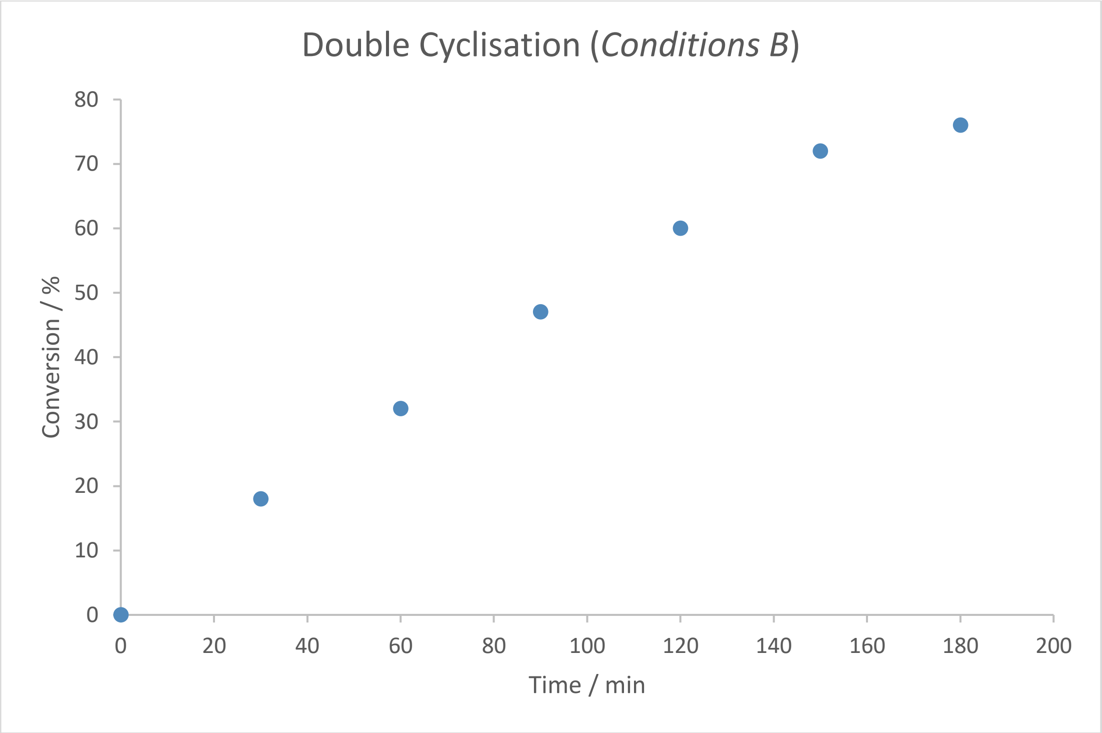

Figure S2: Double cyclisation time-course experiment

iv. Temperature & Stirring Dependence Studies

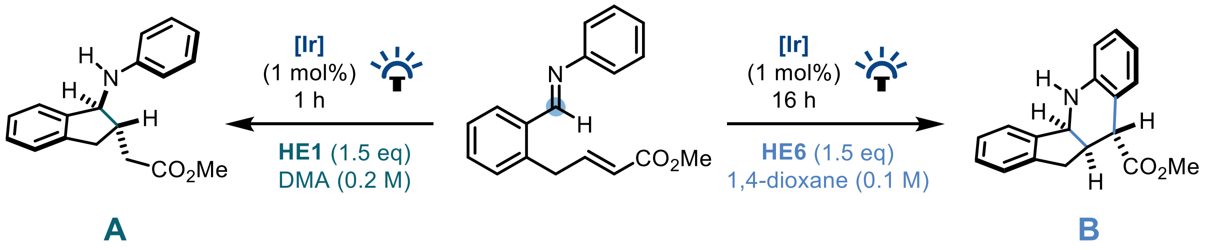

All reactions were conducted according to General Procedure A, using the Hantzsch esters and solvents detailed in the Scheme above (A1-4: HE1 in DMA; B1-4: HE6 in 1,4-dioxane).

The standard temperature of photobox Setup A, the HepatoChem PhotoRedOx Box equipped with an EvoluChem 450 nm 18 W LED, was recorded as 29 °C. A temperature of 23 °C was achieved by blowing a stream of N_2 gas through the photoreactor. A consistent temperature of 40 °C was achieved by turning off the photobox's integral cooling fan.

Table S4: Temperature and stirring dependence studies.

Entry	Temperature / °C	Yield / %	Selectivity A/B	Comment
**A1**	23	72	10:1	
**A2**	29	73	11:1	
**A3**	40	26	>20:1	Major reaction pathway is direct reduction of imine
**A4**	29	62	1:1	No stirring
**B1**	23	63	1:10	
**B2**	29	66	1:3	
**B3**	40	35	>20:1	No double cyclisation product was observed
**B4**	29	42	1:1.5	No stirring

Entries A1-A3 highlight a relatively low temperature dependence for *Conditions A*, although higher temperatures lead to significant over-reduction of starting material. Entries B1-3 are significant, showing a strong temperature dependence on the selectivity for the double cyclisation. Notably, the double cyclisation product can be more selectively achieved at lower temperatures, and is completely eliminated at higher temperatures.

Crucially, photobox Setup B with the Sigma Aldrich SynLED, cooled by an integral fan and a stream of gas, was recorded as operating at ~23 °C. Therefore, the observed selectivity differences between the two light setups can largely be attributed to temperature differences. This is in accordance with a recent review by König et al.[18]

Entries A4 and B4 serve to demonstrate that stirring differences (and by analogy also reaction vial size and shape differences) can have a significant impact on reaction outcome. This finding may be influenced by partial insolubility of the parent Hantzsch species **HE1** and **HE6** in the reaction solvents.

v. Deuterium Labelling Studies

All reactions were conducted according to General Procedure A, using the Hantzsch esters and solvents detailed in the Schemes below. Deuterated Hantzsch esters were synthesised as described in Section 4. Where a non-zero deuterium incorporation is given, the figure pertains to the indicated position only, with no other positions undergoing observable deuterium incorporation. Data are not given for the NH deuterium incorporation due to the likelihood of facile exchange.

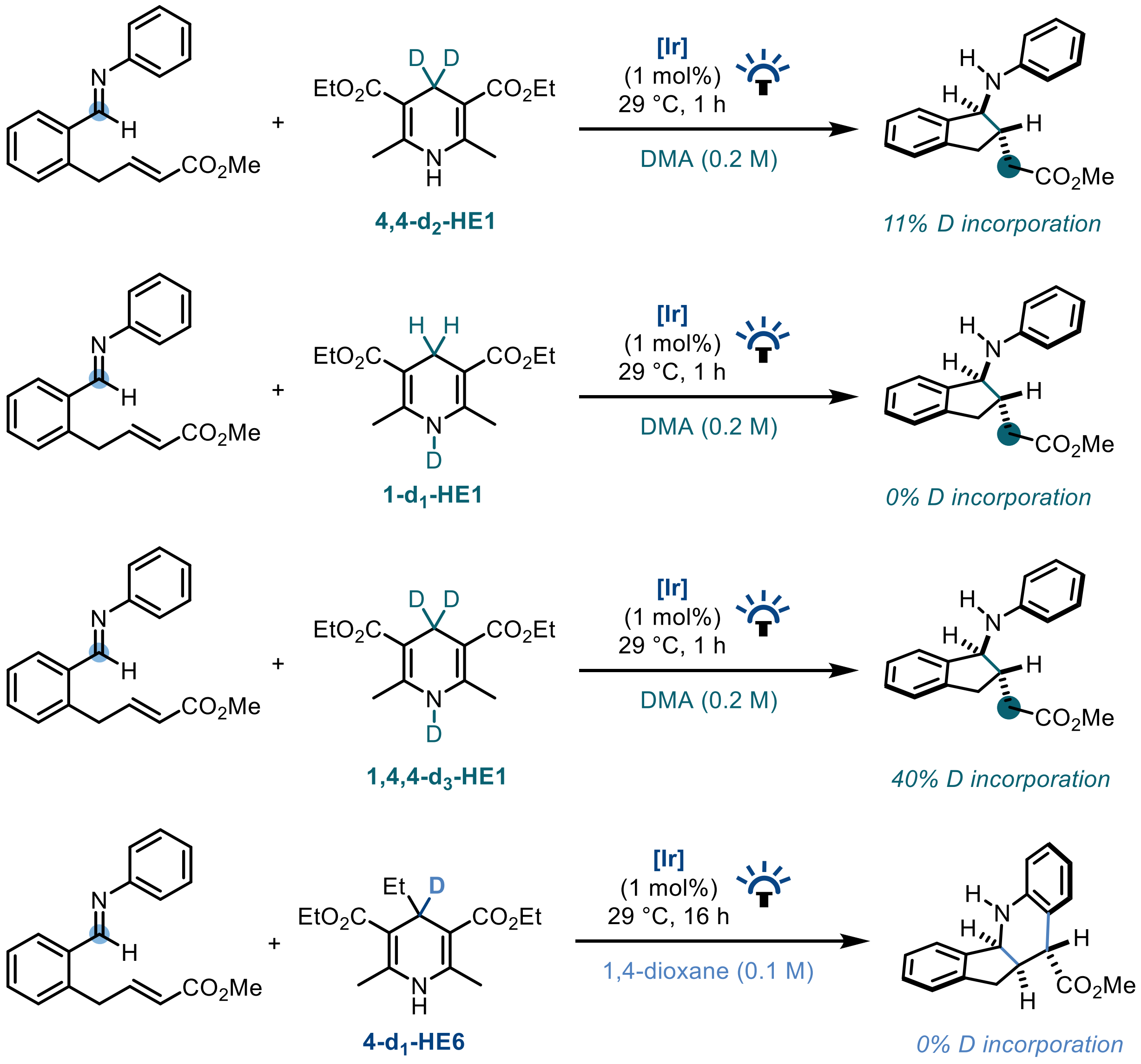

The observed deuterium incorporations with **4,4-d$_2$-HE1** and **1,4,4-d$_3$-HE1** suggest competitive HAT and ET/PT termination pathways for the single cyclisation are in operation. The relatively low deuterium incorporation with **4,4-d$_2$-HE1** and zero deuterium incorporation with **1-d$_1$-HE1** may suggest a kinetic isotope effect is causing a swing between HAT and ET/PT pathways.

vi. Electrochemical Experiments

Electrochemical oxidation potentials were measured for **HE1** and **HE6** using an IKA ElectraSyn 2.0 apparatus. For each substrate, 15 mg was dissolved in a solution of $TBAPF_6$ (4 mL, 0.5 M) in anhydrous degassed MeCN. The cyclic voltammograms shown below are for the first run of three per substrate, with the parameters as indicated. The numerical values given are the average of three runs, with potentials read at half-maximum amplitude, and corrected versus ferrocene.

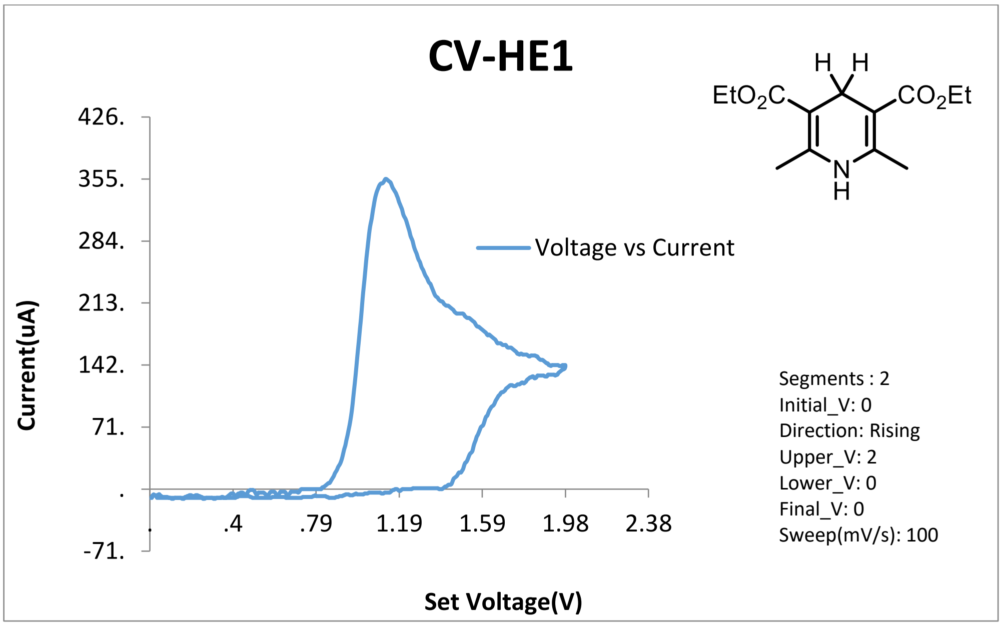

Average potential E_{ox}= +0.97 V vs SCE (corr vs ferrocene)

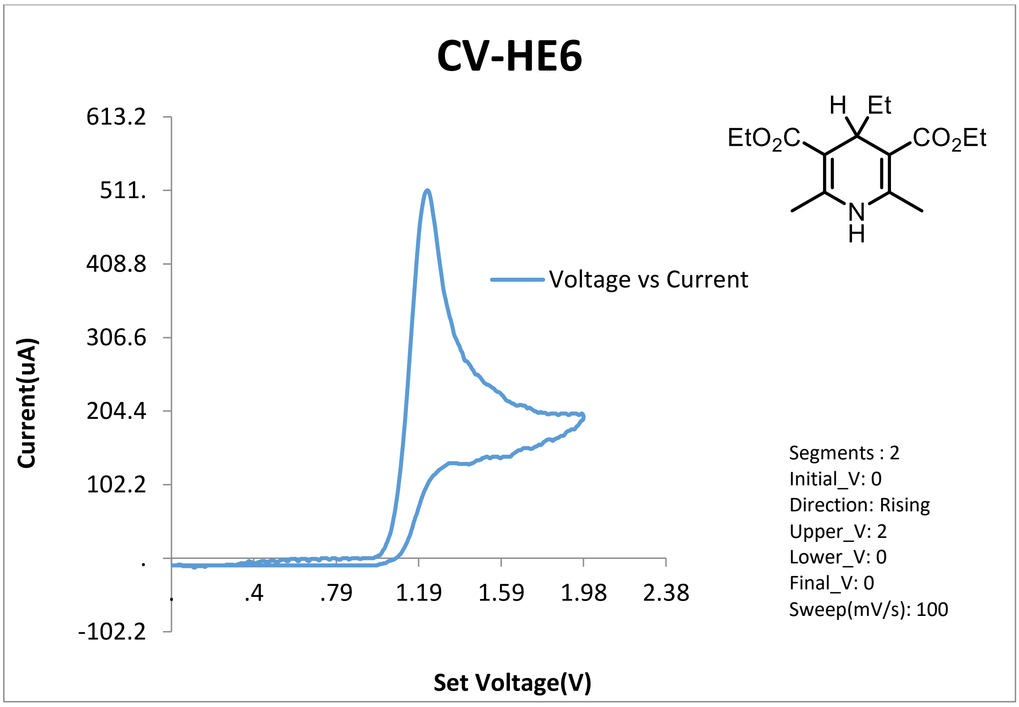

Average potential E_{ox}= +1.10 V vs SCE (corr vs ferrocene)

8. Photoflow Scale-Up

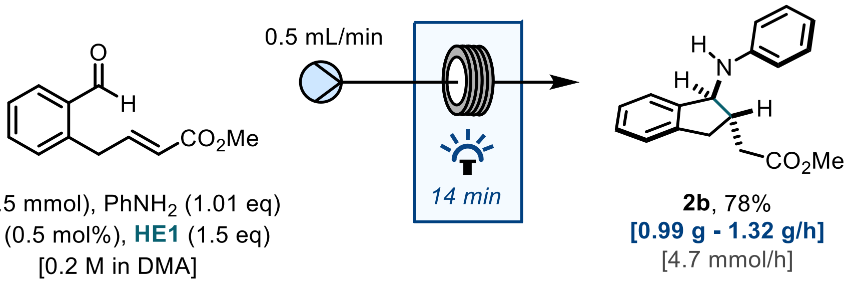

6 (4.5 mmol), $PhNH_2$ (1.01 eq)
[Ir] (0.5 mol%), **HE1** (1.5 eq)
[0.2 M in DMA]

2b, 78%
[0.99 g - 1.32 g/h]
[4.7 mmol/h]

Aldehyde **6** (918 mg, 4.5 mmol, 1 eq.), aniline (414 µL, 4.55 mmol, 1.01 eq.), **HE1** (unsubstituted Hantzsch ester, 1.71 g, 6.75 mmol, 1.5 eq.) and $[Ir(dF(CF_3)ppy)_2(dtbbpy)]PF_6$ (25 mg, 22.5 µmol, 0.5 mol%) were combined in a 50 mL round-bottom flask under inert atmosphere. Anhydrous degassed *N*,*N*-dimethylacetamide (22.5 mL, 0.2 mol L^{-1}) and acetic acid (56 µL, 0.2 mmol, 20 mol%) were added *via* the septum, and the mixture was stirred 15 minutes in darkness (in order to facilitate imine formation). A line of 1/16 inch outer diameter FEP tubing (connected to an HP Agilent 1050 HPLC pump) was inserted through the septum, and the mixture was then passed through a HepatoChem PhotoRedOx Box equipped with an EvoluChem™ 450PF 450 nm 18 W LED light source (see Section 2 for details of the photoflow setup) at a rate of 0.5 mL/min, giving a residence time of 14 minutes. The irradiated reaction mixture was collected in a round-bottom flask, and the solvent was then removed by distillation (75 °C, 50 mbar). The crude material was purified by column chromatography (SiO_2: acetone/pentane, 5–10%), affording **2b-*trans*** (990 mg, 78%) as a colourless oil. Spectral data matched the batch synthesis of **2b-*trans***.

9. Computational Studies

i. Computational Methods

All calculations reported in this paper were performed using the Amsterdam Density Functional (ADF) software.[19] Equilibrium structures and transition structure geometries were optimised using the unrestricted BLYP functional[20] and the TZ2P basis set.[21] Solvent effects of dimethylformamide (DMF) were accounted for using the conductor-like screening model (COSMO) of solvation.[23] Dispersion interactions were included using Grimme's DFT-D3 correction with Becke-Johnson damping.[24] The zeroth-order regular approximation (ZORA) was used to account for scalar relativistic effects.[25] This level is referred to as COSMO(DMF)-ZORA-(U)BLYP-D3(BJ)/TZ2P. All stationary points have been verified, through vibrational analysis, to be minima (zero imaginary frequencies) or transition structures (one imaginary frequency). The character of the normal mode associated with the imaginary frequency has been analysed to ensure it resembles the reaction under consideration. Optimised structures were illustrated using CYLview.[26] Potential energies were refined by means of single point calculations using the unrestricted M06-2X functional[27] and the TZ2P basis set.[21] This level is denoted COSMO(DMF)-ZORA-(U)M06-2X/TZ2P//COSMO(DMF)-ZORA-(U)BLYP-D3(BJ)/TZ2P.

ii. Results

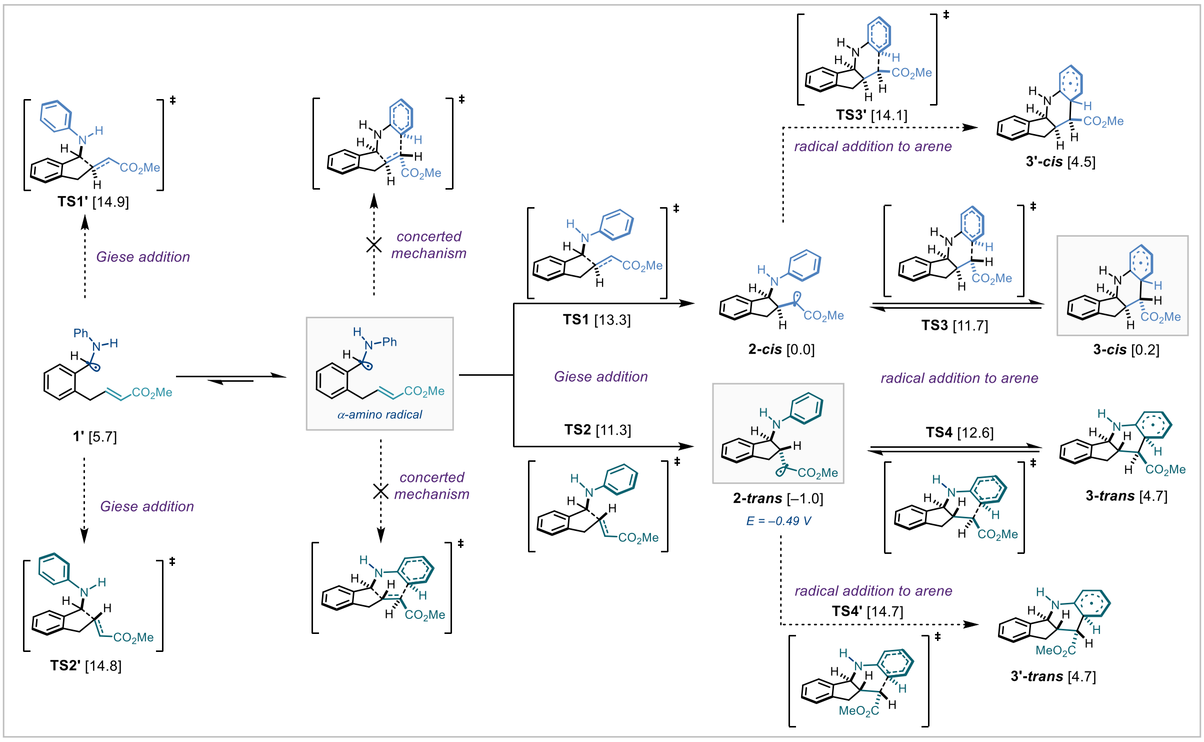

Scheme S1: Possible pathways and their associated energies (ΔG [kcal mol^{-1}]) for the redox-controlled diastereodivergent photocatalytic cyclisation computed at COSMO(DMF)-ZORA-(U)M06-2X/TZ2P//COSMO(DMF)-ZORA-(U)BLYP-D3(BJ)/TZ2P level of theory.

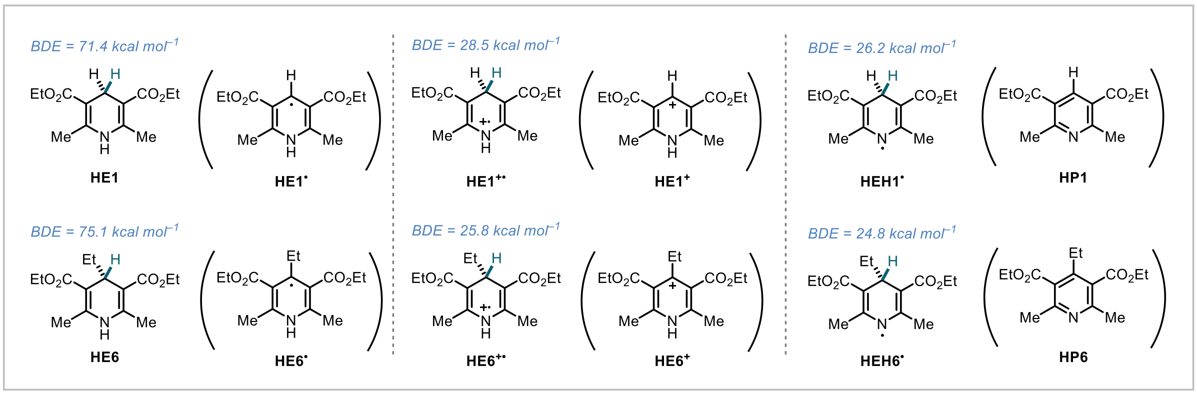

Scheme S2. Bond dissociation energies of Hantzsch esters and oxidised forms (in kcal mol^{-1}) computed at ZORA-(U)BLYP-D3(BJ)/TZ2P level of theory.

The calculated BDEs reveal that neither **HE1** nor **HE6** parent Hantzsch species is as efficient an HAT donor as any of the related oxidised Hantzsch forms. For both Hantzsch esters, the oxidised forms are likely efficient HAT agents.

The calculated reduction potential of radical intermediate **2-*trans*** (Scheme S1, –0.49 V) compared with previously-reported[22] reduction potentials for HEH• to HP^{+} with various substituted and unsubstituted Hantzsch esters (–1.32 to –1.56 V) suggests that ET/PT should also be a feasible termination pathway with both **HE1** and **HE6**.

iii. Computational Details

Table S5: Cartesian coordinates (in Å), energies (in kcal mol^{-1}), and number of imaginary frequencies of all stationary points, computed at COSMO(DMF)-ZORA-(U)BLYP-D3(BJ)/TZ2P. Energies (in kcal mol^{-1}) at COSMO(DMF)-ZORA-(U)M06-2X/TZ2P//COSMO(DMF)-ZORA-(U)BLYP-D3(BJ)/TZ2P are also provided.

1

COSMO(DMF)-ZORA-(U)M06-2X/TZ2P//COSMO(DMF)-ZORA-(U)BLYP-D3(BJ)/TZ2P

E = -7831.37

G = -7670.68

COSMO(DMF)-ZORA-(U)BLYP-D3(BJ)/TZ2P

E = -5510.84

G = -5350.15

N_{imag} = 0

C	-1.37803500	-1.15348600	-0.47314500
C	-2.39567900	-1.43208800	0.43600400
C	-2.50270900	-2.67930400	1.07524700
C	-1.55123200	-3.67242500	0.79465800
C	-0.52123300	-3.42580000	-0.10467300
C	-0.39751300	-2.16710600	-0.77369100
H	-3.12883400	-0.65684000	0.65283800
H	-3.30975700	-2.86757100	1.77865900

H	-1.61340500	-4.64154500	1.28556700
H	0.20530700	-4.21414400	-0.28745600
C	0.64217000	-1.90031600	-1.69866200
C	-1.30966900	0.22174300	-1.12350500
C	1.90077000	2.40584500	-0.98247900
H	-2.22038900	0.77057000	-0.84542100
H	-1.30894400	0.14199000	-2.21921900
C	-0.12313400	1.03117500	-0.67033500
H	0.04534100	1.08514400	0.40590700
C	0.73040400	1.66335900	-1.49017600
H	0.60817600	1.62763000	-2.57118100
O	2.19773200	2.58476400	0.19638700
O	2.65735500	2.87012200	-2.02064900
C	3.87159000	3.59735800	-1.65352300
H	3.61461900	4.48775700	-1.07364600
H	4.33554000	3.87231100	-2.60056000
H	4.53219400	2.95110100	-1.06936100
N	1.59592600	-2.80936600	-2.07095300
H	3.22173400	-4.65320100	-3.00001600
H	1.48431000	-3.77214700	-1.76903100
C	2.72993700	-2.55138100	-2.84529400
C	3.11826400	-1.24180300	-3.20361600
C	4.25781700	-1.04489000	-3.98708300
C	5.03338400	-2.12784000	-4.41925100
C	4.65330000	-3.42846300	-4.05168900
C	3.51579500	-3.64226900	-3.27647100
H	2.55341800	-0.38145500	-2.86007500
H	4.54549800	-0.03026900	-4.25367100
H	5.92001100	-1.96360600	-5.02600700
H	5.24595100	-4.28170700	-4.37410000
H	0.72368800	-0.93278900	-2.16587200

TS1

COSMO(DMF)-ZORA-(U)M06-2X/TZ2P//COSMO(DMF)-ZORA-(U)BLYP-D3(BJ)/TZ2P

E = -7819.76

G = -7657.34

COSMO(DMF)-ZORA-(U)BLYP-D3(BJ)/TZ2P

E = -5503.10

G = -5340.68

$\boldsymbol{N}_{\mathbf{imag}}$ = 1, 95*i* cm^{-1}

C	-3.02905700	-0.09286100	-0.98102900
C	-4.33941900	-0.37806900	-1.38612900
C	-5.15222700	-1.21294700	-0.61280000
C	-4.66154100	-1.76986000	0.57553000
C	-3.35609600	-1.48739800	0.98943200
C	-2.53653200	-0.64906200	0.21516500
H	-4.72608900	0.05871500	-2.30501900
H	-6.16968200	-1.42592300	-0.93309900
H	-5.29465800	-2.41586100	1.17925400
H	-2.96975800	-1.91331300	1.91277000
C	-1.14705700	-0.30881000	0.61461600

C	-2.08429200	0.78875800	-1.77520000
C	1.27868100	2.25824000	-0.55259500
H	-1.61611400	0.20074400	-2.57835400
H	-2.65710800	1.58514800	-2.27211300
C	-1.01459700	1.39981700	-0.89892100
H	-1.36040100	2.08509000	-0.12604800
C	0.30246700	1.49889000	-1.29943000
H	0.66753700	0.91552900	-2.14162700
O	1.06927700	2.96086300	0.44673500
O	2.54927000	2.10788400	-1.08189300
C	3.61582500	2.78528000	-0.36177200
H	3.67552300	2.41013200	0.66356600
H	4.52921100	2.54642300	-0.90846400
H	3.44776000	3.86665100	-0.35061400
N	-0.15668500	-1.21026600	0.32195700
H	1.76731500	-2.63327000	-0.76135500
H	-0.36865000	-1.88655200	-0.40740700
C	1.19902300	-1.09850000	0.65431600
C	1.66148500	-0.19542200	1.63278100
C	3.02288500	-0.13482600	1.92974400
C	3.94262100	-0.96146100	1.27207400
C	3.48021600	-1.86329500	0.30229500
C	2.12334300	-1.93661100	-0.00467800
H	0.97391500	0.47104800	2.14107800
H	3.36767800	0.57009400	2.68265300
H	5.00196400	-0.90223200	1.50756400
H	4.18088200	-2.51050000	-0.22035600
H	-0.98153500	0.30611200	1.49233900

TS2
COSMO(DMF)-ZORA-(U)M06-2X/TZ2P//COSMO(DMF)-ZORA-(U)BLYP-D3(BJ)/TZ2P
E = -7822.59
G = -7659.33
COSMO(DMF)-ZORA-(U)BLYP-D3(BJ)/TZ2P
E = -5504.06
G = -5340.80
$\boldsymbol{N}_{\mathbf{imag}}$ = 1, 281*i* cm^{-1}

C	-3.02571100	-0.20305600	-1.16931100
C	-4.17905600	-0.57708500	-1.85816000
C	-4.49425000	-1.93685900	-2.00500100
C	-3.65246400	-2.91893100	-1.46887900
C	-2.49579500	-2.55037900	-0.76903000
C	-2.19000700	-1.19229800	-0.61009300
H	-4.82001700	0.18254300	-2.30163900
H	-5.38758300	-2.22852200	-2.55269300
H	-3.89550300	-3.97163800	-1.59390300
H	-1.84865700	-3.31121000	-0.33819900
C	-1.01632500	-0.65241300	0.12116600
C	-2.50869900	1.21035500	-0.98346500
C	1.19066000	2.20937400	-0.36926300
H	-2.82163200	1.85899100	-1.81253800

H	-2.91676500	1.65446900	-0.06220800
C	-0.98682500	1.13740500	-0.88831600
H	-0.50337000	0.73591600	-1.78115500
C	-0.22950700	2.07565200	-0.16856500
H	-0.67672900	2.63044600	0.65329300
O	1.87422300	1.65394200	-1.24290600
O	1.76225400	3.05976900	0.56718300
C	3.21119500	3.17014200	0.50543400
H	3.52988800	3.56056000	-0.46584800
H	3.48192100	3.86417600	1.30266500
H	3.66895500	2.19130900	0.67813800
N	0.17840100	-1.34287100	0.06627300
H	2.56344900	-2.32981700	-0.41063600
H	0.34168300	-1.88975300	-0.77527400
C	1.32220500	-1.05972500	0.82354500
C	1.29648300	-0.19218500	1.93292800
C	2.47345800	0.07206800	2.63539300
C	3.68513000	-0.51836600	2.25760800
C	3.70718100	-1.39116600	1.15987300
C	2.54067700	-1.66236700	0.44873400
H	0.37465300	0.29028400	2.23788600
H	2.44082900	0.75457600	3.48132900
H	4.59783100	-0.30107500	2.80623400
H	4.64028100	-1.85746600	0.85196000
H	-1.23246300	-0.22479200	1.09744100

2-cis
COSMO(DMF)-ZORA-(U)M06-2X/TZ2P//COSMO(DMF)-ZORA-(U)BLYP-D3(BJ)/TZ2P
E = -7834.95
G = -7670.69
COSMO(DMF)-ZORA-(U)BLYP-D3(BJ)/TZ2P
E = -5508.99
G = -5344.73
N_{imag} = 0

C	-2.78055056	-0.28632191	-1.27463151
C	-3.98507719	-0.67035842	-1.87016530
C	-4.69215318	-1.75764585	-1.33767620
C	-4.20013247	-2.45486775	-0.22408898
C	-2.99082795	-2.07146249	0.37205133
C	-2.29456484	-0.98388921	-0.15878611
H	-4.37381954	-0.13258382	-2.73256644
H	-5.63398010	-2.06123029	-1.78947185
H	-4.76216406	-3.29321025	0.18100077
H	-2.60486582	-2.60958508	1.23520545
C	-0.96172361	-0.42816143	0.29945814
C	-1.84494857	0.83908040	-1.66192114
C	0.89820790	2.62964784	0.28873476
H	-1.21962713	0.54764569	-2.51817574
H	-2.37011308	1.75756893	-1.94466280
C	-0.97374622	1.05212974	-0.38889083
H	-1.53795737	1.65765606	0.33060853

C	0.33733558	1.67037217	-0.61820193
H	0.92932351	1.38808976	-1.48405122
O	0.38241365	3.03304499	1.34463321
O	2.13668033	3.07433177	-0.13660986
C	2.81979974	3.98784416	0.76589439
H	2.98838853	3.50718364	1.73472728
H	3.77004908	4.21507709	0.28124135
H	2.23261999	4.90021539	0.90813929
N	0.11564705	-1.29765229	-0.13870388
H	2.15762525	-2.19459662	-1.55174580
H	-0.05212463	-1.75812897	-1.02909962
C	1.45201758	-1.02578771	0.12945079
C	1.84789049	-0.22685731	1.22678726
C	3.20202496	0.01519565	1.46662224
C	4.18666457	-0.52833333	0.63582187
C	3.79868975	-1.33109000	-0.44994674
C	2.45330126	-1.57967386	-0.70362934
H	1.10455758	0.20425585	1.88763026
H	3.48522326	0.64030004	2.31027235
H	5.23874390	-0.33163428	0.82502639
H	4.55288660	-1.75936749	-1.10659985
H	-0.90110624	-0.30192582	1.38326949

2-*trans*
COSMO(DMF)-ZORA-(U)M06-2X/TZ2P//COSMO(DMF)-ZORA-(U)BLYP-D3(BJ)/TZ2P
E = -7836.06
G = -7671.69
COSMO(DMF)-ZORA-(U)BLYP-D3(BJ)/TZ2P
E = -5509.72
G = -5345.35
N_{imag} = 0

C	-2.99570700	-0.17355100	-1.27798200
C	-4.12783700	-0.46830500	-2.03887900
C	-4.50305300	-1.80993500	-2.21076400
C	-3.75475500	-2.84036500	-1.62501100
C	-2.62059500	-2.54397100	-0.85378900
C	-2.25226000	-1.20848000	-0.69045100
H	-4.70739000	0.32734900	-2.50287800
H	-5.37721400	-2.05269000	-2.81094900
H	-4.05289400	-3.87601000	-1.77163200
H	-2.03730700	-3.34024700	-0.39685600
C	-1.09846500	-0.64374000	0.11234900
C	-2.39190300	1.17605200	-0.94804900
C	1.09892600	2.21523800	0.12700200
H	-2.42018100	1.88390300	-1.78287000
H	-2.92302200	1.64064900	-0.10422000
C	-0.92651200	0.82901300	-0.53578400
H	-0.32895500	0.67555100	-1.44192700
C	-0.25357900	1.78565700	0.34803800
H	-0.76345700	2.14556000	1.23836700
O	1.81712400	1.91527100	-0.83879700

O	1.54697700	3.03826100	1.14568100
C	2.94357000	3.43670200	1.05656200
H	3.12406000	4.01072200	0.14264500
H	3.12406000	4.05384600	1.93772000
H	3.58613600	2.55114400	1.06909100
N	0.09919300	-1.44744100	0.07669300
H	2.58356700	-2.07970600	-0.54320300
H	0.32395200	-1.79814700	-0.85180900
C	1.22204700	-1.12774100	0.84245300
C	1.12480900	-0.41733300	2.05895600
C	2.27911100	-0.10188700	2.78198100
C	3.54112700	-0.49072000	2.32530900
C	3.63889200	-1.21807400	1.12740200
C	2.49925000	-1.53457000	0.39520600
H	0.15778100	-0.11469100	2.44405100
H	2.18365000	0.45921000	3.70863200
H	4.43505100	-0.23491000	2.88821800
H	4.61374600	-1.52808900	0.75718700
H	-1.41074100	-0.51654600	1.15563500

TS3
COSMO(DMF)-ZORA-(U)M06-2X/TZ2P//COSMO(DMF)-ZORA-(U)BLYP-D3(BJ)/TZ2P
E = -7824.53
G = -7658.99
COSMO(DMF)-ZORA-(U)BLYP-D3(BJ)/TZ2P
E = -5498.30
G = -5332.76
N_{imag} = 1, 485*i* cm^{-1}

C	-1.61994825	-0.75378778	-3.16045271
C	-2.17872730	-1.35103467	-4.29289942
C	-2.61030639	-2.68354341	-4.21815577
C	-2.48629348	-3.41071047	-3.02526330
C	-1.92734565	-2.81175444	-1.88724800
C	-1.50107629	-1.48498109	-1.96746860
H	-2.28221621	-0.79281866	-5.22121608
H	-3.05125662	-3.15673855	-5.09284497
H	-2.83165206	-4.44099216	-2.98138795
H	-1.83434891	-3.37078069	-0.95845854
C	-0.84696936	-0.64998320	-0.88882206
C	-1.07342815	0.64773483	-2.98865854
C	-0.46556437	3.08535110	-0.41229333
H	-0.07190869	0.72603496	-3.43564698
H	-1.69748907	1.41537017	-3.45874347
C	-1.00187186	0.82504833	-1.44072680
H	-1.98493143	1.15690304	-1.08792966
C	0.03265454	1.82705170	-0.96887221
H	0.89129358	1.95721209	-1.62477865
O	-1.54077583	3.24139851	0.18070411
O	0.46738358	4.08908505	-0.51898258
C	0.13162323	5.34272620	0.14660024
H	-0.02339185	5.17479251	1.21638271

H	0.99032665	5.99400563	-0.01966822
H	-0.77139699	5.77683894	-0.29213446
N	0.56000393	-1.05145061	-0.72108273
H	3.22127376	-1.20368099	-0.85459353
H	0.92330745	-1.71550891	-1.39814138
C	1.46155031	-0.20281953	-0.12667546
C	0.91962757	0.96603395	0.56382354
C	1.88060973	1.82691594	1.22392856
C	3.23843411	1.65072635	1.05032103
C	3.73106255	0.56006297	0.29305917
C	2.84510518	-0.35953120	-0.27916500
H	-0.01888036	0.81576037	1.09803682
H	1.50536290	2.64188226	1.83687896
H	3.93952380	2.33499584	1.52352697
H	4.80232112	0.42559145	0.16634468
H	-1.35651587	-0.74350784	0.07789660

TS4
COSMO(DMF)-ZORA-(U)M06-2X/TZ2P//COSMO(DMF)-ZORA-(U)BLYP-D3(BJ)/TZ2P
E = -7823.88
G = -7658.04
COSMO(DMF)-ZORA-(U)BLYP-D3(BJ)/TZ2P
E = -5496.31
G = -5330.47
$\boldsymbol{N}_{\mathbf{imag}}$ = 1, 479*i* cm^{-1}

C	-2.92215700	0.75626500	1.09804600
C	-4.14321200	0.58871700	1.75105900
C	-4.17572500	-0.10962500	2.96910600
C	-3.00041400	-0.63271000	3.52464500
C	-1.76841400	-0.45847800	2.87418800
C	-1.74350000	0.23121500	1.66306900
H	-5.06164500	0.98409700	1.32150100
H	-5.12416200	-0.25449200	3.48169700
H	-3.04239400	-1.17675300	4.46541600
H	-0.85516900	-0.85901600	3.30947500
C	-0.57291900	0.58119200	0.77566500
C	-2.61954500	1.45430200	-0.21955900
C	-0.46293700	1.13453000	-2.99252600
H	-3.39108100	1.30147900	-0.98117500
H	-2.51191500	2.53873500	-0.06705500
C	-1.25314200	0.82155300	-0.60813000
H	-1.45800200	-0.17788200	-1.01299100
C	-0.35626400	1.54122500	-1.58308300
H	-0.29906000	2.62165900	-1.45351400
O	-0.83323300	0.03141500	-3.40005500
O	0.00898000	2.10999200	-3.83316100
C	0.12805400	1.73258900	-5.23772300
H	-0.85260200	1.47580400	-5.64795200
H	0.53522000	2.61218600	-5.73712400
H	0.80697300	0.88112700	-5.33896700
N	0.49894100	-0.41400100	0.66236100

H	1.78129400	-2.38442900	-0.62463700
H	0.21476200	-1.36639900	0.88404900
C	1.32090400	-0.29410500	-0.45280900
C	1.44833000	1.04547400	-1.04141300
C	2.41783500	1.17611000	-2.12041400
C	3.01402300	0.06911100	-2.68460200
C	2.78065600	-1.22553300	-2.15739500
C	1.94641100	-1.39277200	-1.04343300
H	1.47994600	1.85333400	-0.30892200
H	2.62597900	2.17180300	-2.50196800
H	3.69807900	0.19049800	-3.52219700
H	3.25484800	-2.09194800	-2.61086700
H	-0.13193400	1.52631800	1.13108300

3-*cis*
COSMO(DMF)-ZORA-(U)M06-2X/TZ2P//COSMO(DMF)-ZORA-(U)BLYP-D3(BJ)/TZ2P
E = -7836.13
G = -7670.43
COSMO(DMF)-ZORA-(U)BLYP-D3(BJ)/TZ2P
E = -5503.17
G = -5337.47
N_{imag} = 0

C	-2.93408400	0.01184300	-1.07584300
C	-4.25227300	-0.39086300	-1.30432900
C	-4.88159500	-1.22708700	-0.37064000
C	-4.20233000	-1.65445200	0.77963200
C	-2.88045900	-1.24779700	1.01110700
C	-2.25845200	-0.41786500	0.07658400
H	-4.78821000	-0.05945000	-2.19159000
H	-5.90938900	-1.54261000	-0.53645300
H	-4.70625200	-2.29613400	1.49865000
H	-2.35461100	-1.56928800	1.90819500
C	-0.84649300	0.12696500	0.11344100
C	-2.04371900	0.88799500	-1.93146300
C	0.75241200	2.84154200	-2.09569100
H	-1.69269800	0.32940000	-2.81067900
H	-2.55607800	1.78246500	-2.30276700
C	-0.85541800	1.24117400	-0.98864100
H	-1.08181400	2.17923000	-0.47138200
C	0.50452500	1.39767900	-1.70908400
H	0.53014700	0.78541900	-2.61516600
O	0.66249200	3.79408300	-1.32994600
O	1.13229200	2.95813300	-3.39311200
C	1.45593800	4.31401200	-3.84909600
H	2.28828900	4.71387300	-3.26439900
H	1.73405600	4.20116900	-4.89636900
H	0.58100000	4.96025500	-3.74411200
N	0.13259100	-0.91666600	-0.22186400
H	2.20407100	-2.58093400	-0.42932200
H	-0.07997500	-1.86924500	0.05727200
C	1.43354000	-0.59085100	-0.51066000

C	1.67860000	0.88286300	-0.76819700
C	3.06477500	1.15572100	-1.29108100
C	4.01287900	0.17323200	-1.41073800
C	3.73277400	-1.17929000	-1.07379400
C	2.43850000	-1.53816600	-0.64177700
H	1.53037000	1.44463200	0.17521500
H	3.30872700	2.18614500	-1.53868400
H	5.00706900	0.43394400	-1.77136600
H	4.50328300	-1.93915600	-1.16969400
H	-0.60603600	0.56012800	1.09623200

3-*trans*
COSMO(DMF)-ZORA-(U)M06-2X/TZ2P//COSMO(DMF)-ZORA-(U)BLYP-D3(BJ)/TZ2P
E = -7832.54
G = -7666.01
COSMO(DMF)-ZORA-(U)BLYP-D3(BJ)/TZ2P
E = -5499.33
G = -5332.80
N_{imag} = 0

C	-2.79546000	-0.11034000	-1.25715200
C	-3.91110500	-0.33648100	-2.06268500
C	-4.45242900	-1.63063000	-2.13200300
C	-3.88632000	-2.68234300	-1.39932000
C	-2.76940700	-2.45641700	-0.57879600
C	-2.23128600	-1.17244500	-0.52075500
H	-4.35188800	0.47324200	-2.64097200
H	-5.31579700	-1.81997700	-2.76603500
H	-4.31365200	-3.68013500	-1.46735200
H	-2.33486100	-3.27269000	-0.00534600
C	-1.07292000	-0.64030600	0.28959300
C	-2.01726300	1.17925400	-1.02193300
C	1.22005300	2.29499400	-0.57091500
H	-1.91732700	1.79471100	-1.92195500
H	-2.50989900	1.79201500	-0.25170600
C	-0.66779000	0.62713500	-0.50314200
H	-0.10583600	0.25925000	-1.37164700
C	0.27763100	1.50764100	0.31736800
H	-0.27277800	2.20587100	0.95437700
O	1.76853000	1.85969200	-1.57434500
O	1.42470700	3.54926600	-0.09077400
C	2.40571000	4.36025200	-0.81630400
H	2.08152900	4.50428300	-1.85001000
H	2.43885700	5.31007100	-0.28333400
H	3.38039600	3.86588500	-0.79518600
N	0.09599700	-1.50798100	0.48647100
H	2.58585400	-2.38748200	0.03382800
H	0.16381900	-2.29359600	-0.15775900
C	1.29320200	-0.84251600	0.74105400
C	1.14236600	0.54941400	1.31913000
C	2.45270900	1.15123300	1.74175200
C	3.65259600	0.54619800	1.47626200

C	3.72349700	-0.72179300	0.83641300
C	2.53506000	-1.40014800	0.49248800
H	0.47185500	0.48492800	2.19264000
H	2.41501700	2.11546400	2.24230900
H	4.57628100	1.04070700	1.77392600
H	4.68791800	-1.17537000	0.62519100
H	-1.45126200	-0.34412200	1.28241600

1’
COSMO(DMF)-ZORA-(U)M06-2X/TZ2P//COSMO(DMF)-ZORA-(U)BLYP-D3(BJ)/TZ2P
E = -7827.50
G = -7664.98
COSMO(DMF)-ZORA-(U)BLYP-D3(BJ)/TZ2P
E = -5506.42
G = -5343.90
N_{imag} = 0

C	-1.21564392	-1.17355808	-0.47541640
C	-2.28772745	-1.25795320	0.41369631
C	-2.39754772	-2.29995531	1.34662318
C	-1.39338234	-3.28037598	1.39412782
C	-0.31747903	-3.22721910	0.51825430
C	-0.18837531	-2.18074726	-0.44717490
H	-3.06258132	-0.49408689	0.37170567
H	-3.24802281	-2.34359196	2.02244953
H	-1.45156326	-4.08579573	2.12353062
H	0.45721722	-3.98349185	0.58382754
C	0.92876948	-2.10057007	-1.32703686
C	-1.14934392	-0.00288160	-1.44786929
C	1.67109918	2.64258040	-1.06325064
H	-2.15846373	0.42282336	-1.53831445
H	-0.84383904	-0.33330272	-2.44784592
C	-0.23041518	1.08082921	-0.95188583
H	-0.43628178	1.46482402	0.04807244
C	0.81115897	1.58460789	-1.63122024
H	1.06135617	1.23535850	-2.63103251
O	1.55172661	3.16398780	0.04321575
O	2.65628569	2.98663014	-1.94482666
C	3.58031291	4.02762331	-1.49581702
H	3.03349106	4.95316791	-1.29722497
H	4.28114717	4.16027511	-2.31986468
H	4.10060530	3.70143132	-0.59126147
N	1.84461180	-3.10269003	-1.57579768
H	3.76002308	-4.90279474	-1.55514003
H	2.75908479	-2.78379033	-1.88176071
C	1.64328079	-4.48684507	-1.71595794
C	0.36462768	-5.04842188	-1.91096780
C	0.22820452	-6.42950948	-2.06292518
C	1.34720646	-7.27257413	-2.03599311
C	2.62094806	-6.71107048	-1.86199081
C	2.77118150	-5.33402109	-1.70018186
H	-0.50819877	-4.40659052	-1.95020030

H	-0.76432102	-6.84898856	-2.21372538
H	1.23040475	-8.34694493	-2.15411968
H	3.50174580	-7.34924081	-1.84618313
H	1.20124046	-1.14914226	-1.76658735

TS1’

COSMO(DMF)-ZORA-(U)M06-2X/TZ2P//COSMO(DMF)-ZORA-(U)BLYP-D3(BJ)/TZ2P

E = -7819.19

G = -7655.73

COSMO(DMF)-ZORA-(U)BLYP-D3(BJ)/TZ2P

E = -5503.32

G = -5339.86

$\boldsymbol{N}_{\mathbf{imag}}$ = 1, 55i cm^{-1}

C	-3.12620807	1.02652008	0.76535164
C	-4.49931527	1.28733686	0.80361788
C	-5.41380296	0.35751030	0.29299393
C	-4.95415131	-0.84500900	-0.26176556
C	-3.58566000	-1.11887741	-0.29657960
C	-2.66315501	-0.19114186	0.22251368
H	-4.85561179	2.23218034	1.21003868
H	-6.47877843	0.57724605	0.31238328
H	-5.66134167	-1.56564592	-0.66613665
H	-3.22339047	-2.05095339	-0.72383009
C	-1.21333498	-0.43628111	0.23820469
C	-2.07980711	2.01977138	1.25378213
C	0.76094118	2.17256363	-1.37885566
H	-2.45970955	3.04106335	1.11938495
H	-1.92467342	1.88234890	2.33308381
C	-0.75305936	1.84113485	0.53592444
H	0.10957309	1.54746557	1.12823796
C	-0.51860127	2.34016666	-0.72296161
H	-1.32798372	2.75779816	-1.31641235
O	1.78383369	1.66752583	-0.89303536
O	0.72363793	2.63299304	-2.68156134
C	1.91857299	2.38413611	-3.47247970
H	2.10954199	1.30885531	-3.53255867
H	1.69770619	2.78686871	-4.46190848
H	2.78474464	2.89124767	-3.03667817
N	-0.51451994	-0.95691038	-0.82044716
H	1.13321814	-1.86522568	-2.61642643
H	0.38116508	-1.36814465	-0.57470699
C	-0.66079595	-0.72919127	-2.19739566
C	-1.70537054	0.03076644	-2.76275456
C	-1.74763254	0.23123771	-4.14274642
C	-0.77185188	-0.31535259	-4.98526402
C	0.26419460	-1.07549352	-4.42421781
C	0.32113522	-1.28361554	-3.04879635
H	-2.46182064	0.47676456	-2.13258421
H	-2.55293264	0.83058884	-4.56173729
H	-0.81438017	-0.14945119	-6.05858802
H	1.03592003	-1.50284475	-5.06039095

H	-0.74665308	-0.64990314	1.19545913

TS2’

COSMO(DMF)-ZORA-(U)M06-2X/TZ2P//COSMO(DMF)-ZORA-(U)BLYP-D3(BJ)/TZ2P

E = -7818.73

G = -7655.86

COSMO(DMF)-ZORA-(U)BLYP-D3(BJ)/TZ2P

E = -5501.53

G = -5338.66

N_{imag} = 1, 93*i* cm^{-1}

C	-3.10762422	0.98009579	0.74201547
C	-4.45939741	1.32945069	0.79148465
C	-5.42630704	0.46039650	0.26859357
C	-5.04112708	-0.75943805	-0.30420323
C	-3.68990623	-1.11680988	-0.35248154
C	-2.72331060	-0.24902994	0.17612225
H	-4.75827033	2.28518193	1.21792618
H	-6.47704999	0.73986583	0.29548471
H	-5.79291563	-1.42931622	-0.71534598
H	-3.38504811	-2.06079918	-0.79779208
C	-1.26474029	-0.54469863	0.20283046
C	-1.96135622	1.84269259	1.23223807
C	1.77779781	1.48467346	0.30104836
H	-2.19618531	2.90825977	1.10123236
H	-1.80293337	1.68706246	2.31029550
C	-0.69489480	1.47546728	0.47261307
H	-0.71633481	1.68436950	-0.59774344
C	0.56480409	1.53242295	1.07568464
H	0.65746416	1.51761939	2.15954706
O	1.87096305	1.45598458	-0.93505795
O	2.90599659	1.47340788	1.11755201
C	4.18252211	1.44387315	0.42031171
H	4.29352391	2.32818782	-0.21520436
H	4.94056869	1.44080635	1.20495362
H	4.26366254	0.54182641	-0.19421034
N	-0.63577781	-1.17212654	-0.85449086
H	0.67708947	-2.46027792	-2.70484888
H	0.18688980	-1.71460924	-0.61154020
C	-0.78069857	-0.93504917	-2.22924933
C	-1.64941087	0.03942958	-2.75952590
C	-1.73242379	0.21612373	-4.14289530
C	-0.96253894	-0.55935453	-5.01779438
C	-0.09272347	-1.52415375	-4.48911917
C	-0.00010361	-1.71138142	-3.11160415
H	-2.25506965	0.65250946	-2.10478553
H	-2.40603572	0.97360040	-4.53737937
H	-1.03542395	-0.41375166	-6.09252680
H	0.51774606	-2.13257890	-5.15244294
H	-0.88025243	-0.90269446	1.15550859

TS3’

COSMO(DMF)-ZORA-(U)M06-2X/TZ2P//COSMO(DMF)-ZORA-(U)BLYP-D3(BJ)/TZ2P
E = -7822.46
G = -7656.55
COSMO(DMF)-ZORA-(U)BLYP-D3(BJ)/TZ2P
E = -5495.84
G = -5329.93
N_{imag} = 1, 481*i* cm^{-1}

C	-2.97911104	1.11098138	-0.98942403
C	-4.37510771	1.17501893	-1.03420575
C	-5.10533761	1.01511363	0.15165002
C	-4.44917263	0.79174478	1.37145102
C	-3.04981264	0.73082244	1.41858199
C	-2.32757115	0.89406103	0.23391900
H	-4.89174703	1.34842133	-1.97617029
H	-6.19172796	1.06682113	0.12685176
H	-5.02855575	0.67314394	2.28411288
H	-2.53666288	0.56607032	2.36384283
C	-0.82692989	0.85452390	0.05610936
C	-1.99131687	1.22441846	-2.12889195
C	0.55017193	-0.22998938	-3.05615624
H	-2.00192457	0.30849387	-2.72825496
H	-2.21204647	2.06323871	-2.79861296
C	-0.61355239	1.41654045	-1.41759890
H	-0.47440981	2.49426945	-1.27518636
C	0.63020536	0.91200520	-2.14179743
H	1.27027597	1.70309055	-2.52545872
O	-0.24534473	-1.17119581	-2.98987009
O	1.58616610	-0.20559776	-3.96644522
C	1.76608698	-1.42458942	-4.74289215
H	0.87302128	-1.64023317	-5.33625687
H	2.62000466	-1.22915526	-5.39268847
H	1.97704421	-2.26349531	-4.07212790
N	-0.30566564	-0.50117797	0.26129865
H	0.72382945	-2.95785101	0.07663638
H	-0.99106278	-1.24792493	0.32034781
C	0.95282302	-0.83554920	-0.17190070
C	1.77972007	0.24928715	-0.70149530
C	3.11318183	-0.10944726	-1.14765933
C	3.50444192	-1.42998066	-1.23505905
C	2.64491569	-2.46531648	-0.79352896
C	1.38731568	-2.16220993	-0.25764076
H	1.71091204	1.20171105	-0.17626276
H	3.78344162	0.68989712	-1.45321087
H	4.49129907	-1.68005792	-1.61906430
H	2.96408205	-3.50217134	-0.86238674
H	-0.32114967	1.52147217	0.76642306

TS4’
COSMO(DMF)-ZORA-(U)M06-2X/TZ2P//COSMO(DMF)-ZORA-(U)BLYP-D3(BJ)/TZ2P
E = -7822.05
G = -7655.99

COSMO(DMF)-ZORA-(U)BLYP-D3(BJ)/TZ2P
E = -5495.30
G = -5329.24
$\boldsymbol{N}_{\mathbf{imag}}$ = 1, 489*i* cm^{-1}

C	-2.96801887	0.64085785	1.12130996
C	-4.18003605	0.40589310	1.77170694
C	-4.17481256	-0.25982014	3.00783532
C	-2.97018517	-0.68091289	3.58736529
C	-1.74869731	-0.43866148	2.93982890
C	-1.76066874	0.21658188	1.70881557
H	-5.12010274	0.72398265	1.32503723
H	-5.11530070	-0.45605937	3.51801137
H	-2.98182150	-1.19908330	4.54351197
H	-0.81315774	-0.76108909	3.39235445
C	-0.60422288	0.62153697	0.82734339
C	-2.71074077	1.33340259	-0.20785793
C	-0.38961369	3.05091782	-1.46738359
H	-3.45026625	1.08357600	-0.97586018
H	-2.71242977	2.42285923	-0.07790890
C	-1.28440237	0.82346107	-0.56765188
H	-1.40590282	-0.19044477	-0.97126986
C	-0.41511377	1.58577646	-1.54680462
H	-0.44714356	1.21213074	-2.56734868
O	-0.52566525	3.71893884	-0.43705871
O	-0.07844297	3.62103545	-2.67979550
C	0.10438503	5.06963981	-2.67100981
H	0.91330012	5.34479722	-1.98813895
H	0.36084006	5.33155587	-3.69775261
H	-0.82054880	5.56687735	-2.36549657
N	0.50331641	-0.34216115	0.72164740
H	1.74053712	-2.31566096	-0.60566491
H	0.23685691	-1.29984540	0.94544023
C	1.29491905	-0.22293263	-0.41619828
C	1.41159005	1.11557825	-1.00534089
C	2.32316505	1.24097314	-2.13167186
C	2.89469626	0.13157706	-2.71777770
C	2.68059821	-1.16132014	-2.17818456
C	1.89279727	-1.32474809	-1.03086493
H	1.46980747	1.91735604	-0.26673681
H	2.51456707	2.23554308	-2.52598138
H	3.54055105	0.24892684	-3.58559441
H	3.13669140	-2.02937552	-2.64683078
H	-0.20178369	1.58320363	1.17347694

3'-*cis*
COSMO(DMF)-ZORA-(U)M06-2X/TZ2P//COSMO(DMF)-ZORA-(U)BLYP-D3(BJ)/TZ2P
E = -7833.29
G = -7666.22
COSMO(DMF)-ZORA-(U)BLYP-D3(BJ)/TZ2P
E = -5500.34
G = -5333.27

N_{imag} = 0

C	-2.97739858	-0.08058224	-1.02888988
C	-4.29814508	-0.50214894	-1.20529579
C	-4.91335727	-1.25839508	-0.19728371
C	-4.21849307	-1.58702071	0.97610428
C	-2.89529307	-1.15937232	1.15586841
C	-2.28724404	-0.40935677	0.14720772
H	-4.84548827	-0.24856320	-2.11101389
H	-5.94207618	-1.58894627	-0.32364876
H	-4.71103667	-2.16824080	1.75220289
H	-2.35723917	-1.40386652	2.06975346
C	-0.87643346	0.13686392	0.12105998
C	-2.09900888	0.70871662	-1.97582054
C	0.62701758	0.61457195	-3.12163798
H	-1.78398551	0.06504540	-2.80517675
H	-2.61211945	1.57340165	-2.41076177
C	-0.89330699	1.14219085	-1.08626954
H	-1.14367407	2.11583817	-0.65237599
C	0.49574886	1.31254216	-1.77721816
H	0.67705797	2.37601873	-1.94822039
O	0.53193441	-0.58788329	-3.31544309
O	0.89774354	1.50941234	-4.11350964
C	1.08547012	0.93990753	-5.45039443
H	0.17784646	0.41832824	-5.76516144
H	1.28775432	1.79325926	-6.09710065
H	1.93031326	0.24638663	-5.44317010
N	0.10306453	-0.93382469	-0.08977775
H	2.15647540	-2.63754178	-0.23465871
H	-0.13518541	-1.86881939	0.22310745
C	1.39416060	-0.65332307	-0.44407109
C	1.64310771	0.79432144	-0.81337392
C	3.02840802	1.03241165	-1.35554467
C	3.96420601	0.03332272	-1.43393384
C	3.67829844	-1.29246785	-1.00773626
C	2.39107444	-1.61322137	-0.52332414
H	1.49553633	1.41345709	0.09340819
H	3.26083418	2.04431760	-1.68065528
H	4.95382417	0.25938344	-1.82948478
H	4.44124738	-2.06363147	-1.07102661
H	-0.63939175	0.67453665	1.05237581

3’-*trans*

COSMO(DMF)-ZORA-(U)M06-2X/TZ2P//COSMO(DMF)-ZORA-(U)BLYP-D3(BJ)/TZ2P

E = -7832.37

G = -7665.95

COSMO(DMF)-ZORA-(U)BLYP-D3(BJ)/TZ2P

E = -5499.30

G = -5332.88

N_{imag} = 0

C	-2.76195126	-0.09754391	-1.25857486
C	-3.85576067	-0.31523217	-2.09600509

C	-4.40414347	-1.60525378	-2.18076775
C	-3.86613142	-2.66098202	-1.43268690
C	-2.77129995	-2.44304253	-0.58115674
C	-2.22588085	-1.16273934	-0.50705586
H	-4.27417635	0.49788525	-2.68594694
H	-5.25064627	-1.78844510	-2.83887134
H	-4.29877547	-3.65558052	-1.51241762
H	-2.35934836	-3.26256959	0.00421702
C	-1.08554872	-0.64271119	0.33602553
C	-1.98908487	1.18999308	-0.99768593
C	-0.46685843	2.48524492	1.28163780
H	-1.85341572	1.80468705	-1.89295627
H	-2.51781068	1.80138201	-0.25187049
C	-0.65865442	0.63362443	-0.43258535
H	-0.06947795	0.27666352	-1.28760587
C	0.29755970	1.49443740	0.42432396
H	0.97009028	2.05219068	-0.23199961
O	-1.10394247	2.20069863	2.28880237
O	-0.38567757	3.74469416	0.77961265
C	-1.14378121	4.77372104	1.50029116
H	-0.78242356	4.84990739	2.52870097
H	-0.95856971	5.69661545	0.95212888
H	-2.20723502	4.52208603	1.49491554
N	0.07736218	-1.52047979	0.53494561
H	2.53885395	-2.45023401	0.06104153
H	0.12728496	-2.31870127	-0.09513956
C	1.28677441	-0.87164807	0.76608926
C	1.17010867	0.52157318	1.35601811
C	2.50565756	1.12993230	1.68441187
C	3.68942430	0.49716996	1.41078464
C	3.72265659	-0.79425877	0.81518164
C	2.51462043	-1.45541103	0.50551979
H	0.56131946	0.43846934	2.27450599
H	2.49776583	2.11710519	2.14296740
H	4.62885596	0.99201541	1.65343406
H	4.67366640	-1.27138742	0.59539543
H	-1.47755285	-0.35556834	1.32425037

Table S6. Cartesian coordinates (in Å), energies (in kcal mol^{-1}), and number of imaginary frequencies of all stationary points, computed at ZORA-(U)BLYP-D3(BJ)/TZ2P.

H$^{•}$
ZORA-(U)BLYP-D3(BJ)/TZ2P
E = -22.14
H = -20.66
G = -28.42
$\boldsymbol{N}_{\mathbf{imag}}$ = 0

H	0.00000000	0.00000000	0.00000000

HE1
ZORA-(U)BLYP-D3(BJ)/TZ2P
E = -4932.72
H = -4731.97
G = -4776.94
$\boldsymbol{N}_{\mathbf{imag}}$ = 0

C	-0.43456612	-2.42517930	-0.72913661
C	-1.71897892	-3.18201639	-0.41643604
C	-1.70694778	-4.54394699	-0.38068451
N	-0.47676054	-5.19658229	-0.49765007
C	0.76058534	-4.57558910	-0.30806733
C	0.80990485	-3.21450669	-0.34261036
C	-2.90521683	-2.33036868	-0.23591584
C	-2.87169534	-5.48891881	-0.20832256
H	-0.49146421	-6.20713921	-0.44228154
C	1.88978225	-5.54922924	-0.06985320
C	2.00605296	-2.39428408	-0.09520116
H	-7.28339333	-2.49835514	0.89748089
O	2.04418752	-1.18302783	-0.27599240
O	3.09699274	-3.09855189	0.37912402
H	-6.14035071	-3.60318209	1.69208617
C	4.29563191	-2.29719354	0.61767010
C	5.38383485	-3.24202972	1.10470954
H	4.05874017	-1.52464164	1.35788468
H	4.57320473	-1.79170250	-0.31418159
O	-2.89856091	-1.11754410	-0.41055148
O	-4.04237468	-3.00627448	0.16490302
C	-5.23079781	-2.17280547	0.33663643
H	-5.01718429	-1.41044244	1.09419064
C	-6.37143261	-3.08968575	0.75192677
H	-5.43849901	-1.65588964	-0.60703666
H	6.29985676	-2.67392718	1.30659170
H	5.61147473	-4.00394283	0.35051141
H	5.08083225	-3.74631990	2.02936302
H	-6.57072515	-3.84422118	-0.01758039
H	-0.43713647	-1.46327881	-0.21156202

H	-0.40005418	-2.16612971	-1.80181901
H	-2.58931211	-6.50054861	-0.52527201
H	-3.73690282	-5.16600272	-0.78795139
H	-3.18553481	-5.53202741	0.84145139
H	2.79104926	-5.25481496	-0.60857062
H	1.59692089	-6.55642446	-0.39131249
H	2.15040971	-5.58957501	0.99437050

HE1•

ZORA-(U)BLYP-D3(BJ)/TZ2P

E = -4831.26

H = -4639.91

G = -4683.44

N_{imag} = 0

C	-0.43171327	-2.54078952	-0.28505863
C	-1.69279830	-3.22626088	-0.26089210
C	-1.71993814	-4.60612045	-0.23508402
N	-0.49071903	-5.27351903	-0.19456300
C	0.77551156	-4.65135310	-0.20827858
C	0.78887421	-3.26947095	-0.24088391
C	-2.90208909	-2.35558835	-0.29126136
C	-2.89892826	-5.53562030	-0.25426056
H	-0.50912975	-6.27911784	-0.31925833
C	1.91376952	-5.62510363	-0.22028941
C	2.03874283	-2.44608191	-0.26005525
H	-7.29985507	-2.36584999	0.76385996
O	2.11357438	-1.33785530	-0.76167295
O	3.09080739	-3.03831041	0.39634359
H	-6.15709311	-3.28419093	1.76882034
C	4.33185499	-2.25807394	0.41841207
C	5.36089707	-3.06569249	1.19291923
H	4.12356538	-1.29094159	0.88861008
H	4.64310382	-2.06837567	-0.61504072
O	-2.88822769	-1.19283954	-0.66294612
O	-4.04059712	-2.96425859	0.18903591
C	-5.24369460	-2.12932493	0.17603212
H	-5.04945123	-1.23141724	0.77274946
C	-6.38051089	-2.96343725	0.74513443
H	-5.43776510	-1.81051663	-0.85438971
H	6.30288294	-2.50647283	1.24337376
H	5.55833582	-4.02705734	0.70517499
H	5.01875656	-3.25772690	2.21597786
H	-6.56299254	-3.85396840	0.13282339
H	-0.41416835	-1.46162056	-0.34700296
H	2.86222112	-5.13589688	-0.42889131
H	1.73771040	-6.40190473	-0.98138634
H	2.00482859	-6.13739686	0.75103146

H	-2.67417767	-6.41131274	-0.88057956
H	-3.79352689	-5.04710969	-0.63305530
H	-3.12278493	-5.90260525	0.75870978

HE6

ZORA-(U)BLYP-D3(BJ)/TZ2P

E = -5667.22

H = -5430.17

G = -5478.55

$\boldsymbol{N}_{\text{imag}}$ = 0

C	-0.50442861	-2.08615614	0.61972375
C	-1.86156165	-2.59928670	0.15274579
C	-2.28225706	-3.81556881	0.60392203
N	-1.35212211	-4.61040861	1.28025167
C	0.03037166	-4.44954236	1.15905656
C	0.48681382	-3.24237192	0.71403953
C	-2.63897249	-1.67419467	-0.68738315
C	-3.63599225	-4.46433832	0.45402252
H	-1.67724821	-5.51345463	1.60329025
C	0.81806982	-5.68949208	1.50623941
C	1.87047943	-2.94657855	0.30966150
H	-6.49790705	-1.39100314	-3.03469822
O	2.23503591	-1.87342690	-0.15440782
O	2.74876452	-4.00096742	0.48145119
H	-6.46735636	-2.31465295	-1.51736876
C	4.12850758	-3.73052216	0.08338299
C	4.93668085	-4.98707217	0.36765459
H	4.49535798	-2.86642388	0.64877330
H	4.14131643	-3.46290253	-0.97920367
O	-2.28086564	-0.53459545	-0.95861471
O	-3.81954241	-2.20438431	-1.17300727
C	-4.61120447	-1.29783552	-2.00240532
H	-4.82472047	-0.38950929	-1.42773847
C	-5.87963256	-2.03700326	-2.39991301
H	-4.01212668	-1.00802234	-2.87308307
H	5.98196567	-4.82532819	0.07797410
H	4.55041888	-5.84044237	-0.20146212
H	4.91111104	-5.23925301	1.43398010
H	-5.64398374	-2.94784166	-2.96205980
C	-0.66460903	-1.34013428	1.97808893
H	-0.12897216	-1.36833955	-0.11195545
H	-1.06702872	-2.04509495	2.71992224
H	-1.42468136	-0.56184666	1.83132265
C	0.62953539	-0.70510963	2.50822331
H	0.43660399	-0.15852749	3.43937930
H	1.05219209	-0.00721489	1.77733040
H	1.39235172	-1.46380948	2.71873944

H	1.16772619	-6.19616759	0.59882409
H	0.19216052	-6.39102887	2.07175289
H	1.70147601	-5.44790639	2.09839783
H	-4.44045139	-3.73378069	0.53665760
H	-3.77328420	-5.23384237	1.22436538
H	-3.73189719	-4.94337418	-0.52850260

HE6•

ZORA-(U)BLYP-D3(BJ)/TZ2P

E = -5562.12

H = -5334.42

G = -5381.73

$\boldsymbol{N}_{\mathbf{imag}}$ = 0

C	-0.30421455	-2.71191647	-0.46048663
C	-1.66071554	-3.09825142	-0.72956454
C	-2.07615036	-4.40737466	-0.66179686
N	-1.13278873	-5.38624741	-0.30666417
C	0.21574589	-5.08438923	-0.06143143
C	0.61331000	-3.76923629	-0.13059373
C	-2.65424272	-2.04638938	-1.13218950
C	-3.44459873	-4.94533755	-0.95095360
H	-1.35860913	-6.35078348	-0.52988149
C	1.06964300	-6.28276520	0.22456684
C	2.05687081	-3.43590657	0.10759544
H	-6.47136331	-0.20384093	0.45171560
O	2.80360303	-2.91044181	-0.69920931
O	2.44757045	-3.78842084	1.37478046
H	-5.18067559	-0.66985449	1.58233580
C	3.85665850	-3.53174992	1.69592098
C	4.08575654	-3.99166259	3.12640673
H	4.05112492	-2.46159145	1.56606151
H	4.48140152	-4.07543464	0.97792677
O	-2.59512789	-1.36792881	-2.14208161
O	-3.65555367	-1.92834359	-0.20279377
C	-4.69368182	-0.93757994	-0.51280144
H	-4.21161439	0.03627613	-0.65004189
C	-5.67928051	-0.93749386	0.64419022
H	-5.16555899	-1.21531048	-1.46244113
H	5.13050579	-3.81183657	3.40717339
H	3.87921173	-5.06251261	3.23324688
H	3.44148606	-3.44245092	3.82181086
H	-6.14352459	-1.92255656	0.76611108
C	0.12576662	-1.26420966	-0.48694615
H	-0.16725152	-0.86641123	-2.61315186
H	0.92121402	-1.10886797	0.25424990
H	-0.71421518	-0.63258982	-0.16658527
C	0.62577184	-0.76766962	-1.86677206

H	1.49451348	-1.35090306	-2.18389286
H	0.91836584	0.28909513	-1.80660646
H	-3.87375904	-5.42532261	-0.05807046
H	-3.39910850	-5.71082837	-1.74260361
H	-4.12891136	-4.16154532	-1.27569979
H	2.12163373	-6.01839243	0.32796772
H	0.97828098	-7.02097571	-0.58854511
H	0.74929934	-6.77937118	1.15318630

HE1$^{+\bullet}$

ZORA-(U)BLYP-D3(BJ)/TZ2P

E = -4775.97

H = -4575.95

G = -4621.19

$\boldsymbol{N}_{\mathbf{imag}}$ = 0

C	-0.45731611	-2.42535026	0.09894316
C	-1.73828602	-3.18431522	0.08135278
C	-1.72886709	-4.56550749	0.04470246
N	-0.49321826	-5.18529829	0.03775421
C	0.75794042	-4.59833654	0.06540267
C	0.80339458	-3.21773367	0.09512298
C	-2.96127086	-2.31238441	0.12327452
C	-2.90747686	-5.49766401	0.01631103
H	-0.50663013	-6.20396536	0.01129673
C	1.91275892	-5.56030529	0.05295794
C	2.04935110	-2.37782236	0.09344786
H	-7.46332713	-2.53174535	-0.03213118
O	1.98678611	-1.16686183	-0.02430830
O	3.18431113	-3.09366350	0.24126173
H	-6.56372360	-3.82757385	0.77747148
C	4.44361107	-2.30069299	0.26244729
C	5.58898160	-3.27154652	0.46583186
H	4.35433359	-1.56883045	1.07103961
H	4.50353981	-1.76046575	-0.68748400
O	-2.86328360	-1.10287525	0.23204613
O	-4.11976448	-2.99912438	0.02761874
C	-5.35767524	-2.17338106	0.06123902
H	-5.34937286	-1.61280872	1.00110624
C	-6.53722067	-3.11727596	-0.05593169
H	-5.29596219	-1.46056172	-0.76680029
H	6.53000034	-2.71012910	0.48586669
H	5.64563347	-3.99902821	-0.35105020
H	5.49304771	-3.80675107	1.41667571
H	-6.51117729	-3.67249185	-0.99973632
H	-0.43734892	-1.69433635	-0.73476230
H	-0.45824427	-1.71312217	0.94815772
H	-2.58137510	-6.54276820	0.00117607

H	-3.52789527	-5.30568416	-0.86377782
H	-3.54492391	-5.33804817	0.89087432
H	2.58320317	-5.34649996	-0.78415086
H	1.56196839	-6.59437186	-0.02810470
H	2.50704514	-5.45710612	0.96576722

HE1$^+$

ZORA-(U)BLYP-D3(BJ)/TZ2P

E = -4721.35

H = -4526.78

G = -4570.92

N_{imag} = 0

C	-0.45690466	-2.54023712	0.03802583
C	-1.69590811	-3.19080457	0.00774258
C	-1.71140963	-4.59176570	-0.02729805
N	-0.49156569	-5.21048202	-0.02452135
C	0.74349062	-4.62447579	0.01803556
C	0.76437902	-3.22370202	0.05228270
C	-2.91661532	-2.28619497	0.00244045
C	-2.90420645	-5.49796639	-0.07260140
H	-0.50468814	-6.23005363	-0.05852552
C	1.91368056	-5.56067337	0.01413092
C	2.00940382	-2.35359429	0.08389369
H	-7.41866446	-2.50287412	0.14957041
O	1.94193600	-1.14827160	-0.04403061
O	3.13718425	-3.06485941	0.27462602
H	-6.46508040	-3.68619204	1.06270769
C	4.39728508	-2.26931522	0.33118862
C	5.53642939	-3.24144085	0.56202881
H	4.28537484	-1.54198761	1.14045465
H	4.48155465	-1.72784927	-0.61555767
O	-2.80841018	-1.07802650	-0.04632427
O	-4.07442871	-2.97193519	0.06156991
C	-5.31336136	-2.14180752	0.06631723
H	-5.24860397	-1.46753048	0.92516447
C	-6.49297509	-3.08965959	0.14448145
H	-5.30537167	-1.54286976	-0.84902357
H	6.47631926	-2.67984856	0.61055083
H	5.61631921	-3.96505479	-0.25639112
H	5.41475680	-3.78060515	1.50765748
H	-6.52309949	-3.76186410	-0.71984158
H	-0.44331237	-1.45300758	0.04915686
H	2.59179947	-5.32159634	-0.81042295
H	1.58743873	-6.60166288	-0.07944474
H	2.49347068	-5.44692746	0.93562827
H	-2.60084200	-6.54868406	-0.12418588
H	-3.53045756	-5.26195271	-0.93818302

H	-3.52964154	-5.34740998	0.81285560

HE6$^{+\bullet}$

ZORA-(U)BLYP-D3(BJ)/TZ2P

E = -5511.13

H = -5274.35

G = -5322.86

***N*$_{imag}$** = 0

C	-0.45795690	-2.36334928	0.17018775
C	-1.87711775	-2.75722804	-0.06459702
C	-2.30582117	-4.02463331	0.28352809
N	-1.33481456	-4.92854097	0.67166579
C	0.03494104	-4.75791110	0.61804000
C	0.49431715	-3.49676480	0.30172960
C	-2.74436740	-1.66981611	-0.62571069
C	-3.70763722	-4.56976370	0.26740729
H	-1.66348489	-5.86069304	0.91863216
C	0.85687329	-5.98861608	0.88423327
C	1.93601856	-3.18353168	0.02622991
H	-6.92922264	-0.91101008	-2.12883588
O	2.25155750	-2.37303237	-0.82675201
O	2.78738166	-3.87757582	0.80899409
H	-6.69618453	-2.00632320	-0.75392914
C	4.23527457	-3.60664026	0.59005008
C	5.00845074	-4.45081350	1.58319173
H	4.39080823	-2.53267728	0.73066135
H	4.46092721	-3.85599059	-0.45110069
O	-2.30301392	-0.55158488	-0.83784719
O	-4.01204765	-2.05999115	-0.87759476
C	-4.89918467	-1.02106327	-1.46571732
H	-4.93444502	-0.18445212	-0.76117503
C	-6.25534577	-1.65808509	-1.69425798
H	-4.42835978	-0.67454044	-2.39079785
H	6.08021741	-4.26537835	1.44878090
H	4.82643238	-5.51932649	1.42542063
H	4.74453820	-4.19259158	2.61416857
H	-6.18733865	-2.50019530	-2.39130571
C	-0.42025669	-1.47309155	1.54136009
H	-0.11992950	-1.66155207	-0.60085866
H	-0.74246885	-2.12768779	2.35905244
H	-1.17857307	-0.70322201	1.38219635
C	0.93637923	-0.83611944	1.83218092
H	0.82183128	-0.15880540	2.68777041
H	1.29699504	-0.24772949	0.98285006
H	1.70027193	-1.57223688	2.10089553
H	1.64598016	-6.09547266	0.13569539
H	0.23519325	-6.88992120	0.86712566

H	1.35041467	-5.91588242	1.85936680
H	-4.39617702	-3.89055979	0.77442243
H	-3.74953276	-5.54998836	0.75448010
H	-4.06542942	-4.67436739	-0.76246756

HE6⁺

ZORA-(U)BLYP-D3(BJ)/TZ2P

E = -5458.14

H = -5227.90

G = -5275.60

$\boldsymbol{N}_{\mathbf{imag}}$ = 0

C	-0.49096379	-2.91077672	-0.39002529
C	-1.75122844	-3.37680915	-0.83113576
C	-1.96509100	-4.73503305	-1.06085978
N	-0.92794197	-5.58448787	-0.81773712
C	0.31168136	-5.21503958	-0.38586815
C	0.53783620	-3.85760952	-0.18204909
C	-2.88752127	-2.43071739	-1.17723026
C	-3.24581013	-5.33053152	-1.56593896
H	-1.09182194	-6.57804458	-0.97763311
C	1.32950289	-6.29737653	-0.17478349
C	1.93472347	-3.39864916	0.19214843
H	-5.61538034	0.53006330	0.86207650
O	2.64432957	-2.78682118	-0.58050899
O	2.24820287	-3.77139045	1.44579594
H	-3.94731947	0.34568531	1.43825231
C	3.60947222	-3.38614987	1.92252823
C	3.74604916	-3.88129271	3.34801787
H	3.68671810	-2.29811828	1.83975510
H	4.33372677	-3.84013434	1.23953878
O	-3.31195266	-2.32955727	-2.31195097
O	-3.33994861	-1.77777402	-0.09460027
C	-4.44574756	-0.80341390	-0.33187200
H	-4.08037420	-0.07016962	-1.05706946
C	-4.80039053	-0.18850372	1.00668829
H	-5.27593159	-1.35545721	-0.78192889
H	4.73683494	-3.60468395	3.72650955
H	3.65327327	-4.97124982	3.40252611
H	2.99187571	-3.42729500	3.99928347
H	-5.13830721	-0.95097511	1.71625334
C	-0.23040507	-1.43829779	-0.20337324
H	-0.74260006	-0.85988093	-2.26504233
H	0.61097470	-1.29477761	0.48024617
H	-1.10906233	-0.97649254	0.25388925
C	0.08493936	-0.74514608	-1.55746555
H	0.99073452	-1.16758352	-2.00071935
H	0.24532415	0.32348799	-1.38496360

H	-4.09539594	-4.97711719	-0.97202963
H	-3.22476063	-6.42405586	-1.52663873
H	-3.42124109	-5.01201793	-2.59994465
H	2.25680025	-6.05652491	-0.70636793
H	0.96944186	-7.26688182	-0.53253544
H	1.57354243	-6.38172229	0.89020445

HEH1$^\bullet$

ZORA-(U)BLYP-D3(BJ)/TZ2P

E = -4826.14

H = -4634.45

G = -4679.11

N_{imag} = 0

C	-0.45889236	-2.39178561	0.11854247
C	-1.73208102	-3.17274815	0.07549543
C	-1.67903929	-4.56592355	0.04719242
N	-0.49348246	-5.23953656	0.05784951
C	0.70782757	-4.59598015	0.10051183
C	0.79476115	-3.20451572	0.13492970
C	-2.94429558	-2.32001050	0.06918776
C	-2.88334761	-5.48113907	0.00331839
H	-3.49526938	-5.28948789	-0.88410123
C	1.88971784	-5.54094523	0.10434002
C	2.02786760	-2.38300709	0.17926657
H	-7.45516056	-2.53395770	-0.03884887
O	2.00768042	-1.15725029	0.16948108
O	3.19117180	-3.10385311	0.23585535
H	-6.54505684	-3.78236957	0.83942530
C	4.42024417	-2.30718583	0.28196379
C	5.58486952	-3.27956594	0.37194666
H	4.36749070	-1.63781235	1.14769422
H	4.46398165	-1.68599075	-0.61967868
O	-2.89232553	-1.09570636	0.10544490
O	-4.12677785	-3.00966616	0.01999940
C	-5.33504412	-2.18019274	0.01702538
H	-5.33089409	-1.55873104	0.91931660
C	-6.52838057	-3.12004200	-0.03327693
H	-5.29395663	-1.51177243	-0.85023825
H	6.52690037	-2.71958358	0.40909806
H	5.61259008	-3.94279982	-0.49977254
H	5.51410094	-3.89469696	1.27590185
H	-6.50571835	-3.73687566	-0.93858999
H	-3.53412076	-5.32270989	0.86949042
H	2.55789904	-5.34597867	-0.74063891
H	1.51027852	-6.56322957	0.04916749
H	2.49155662	-5.41838308	1.01084668
H	-2.52796020	-6.51340662	-0.00832659

H	-0.42967823	-1.67126634	-0.71976659
H	-0.47106877	-1.69780270	0.97902786

HP1

ZORA-(U)BLYP-D3(BJ)/TZ2P

E = -4773.66

H = -4587.60

G = -4631.59

$\boldsymbol{N}_{\mathbf{imag}}$ = 0

C	-0.44825609	-2.51600392	-0.10955725
C	-1.67726371	-3.18129054	-0.10998046
C	-1.65655615	-4.60014439	-0.12611464
N	-0.48816102	-5.27226035	-0.14453334
C	0.69794872	-4.63358590	-0.12002300
C	0.75931536	-3.21717763	-0.09243306
C	-2.88983649	-2.29635712	-0.10844102
C	-2.88219633	-5.48183237	-0.14435242
H	-3.55575236	-5.21406262	-0.96514199
C	1.90165222	-5.54452007	-0.15323475
C	2.00179374	-2.37591751	-0.07519788
H	-7.37150826	-2.50003157	0.40957325
O	2.02182127	-1.19323387	-0.37447793
O	3.10801587	-3.06139310	0.33665137
H	-6.33564472	-3.60157300	1.34294842
C	4.35176785	-2.28313965	0.38573760
C	5.45055917	-3.21829512	0.86241936
H	4.20110135	-1.43590159	1.06338671
H	4.54751893	-1.88382511	-0.61531409
O	-2.84427461	-1.08834681	-0.27649530
O	-4.05358419	-2.97253892	0.11762522
C	-5.26863192	-2.14898011	0.13579634
H	-5.16100523	-1.39464214	0.92270407
C	-6.44233811	-3.08172098	0.38433825
H	-5.34521226	-1.62675914	-0.82392412
H	6.39899766	-2.67045594	0.91438105
H	5.57695038	-4.06119383	0.17396919
H	5.22578926	-3.61256158	1.85966690
H	-6.52627228	-3.83029337	-0.41121700
H	-3.46000492	-5.36945550	0.77968788
H	2.61393309	-5.24250885	-0.92791135
H	1.55799142	-6.56462834	-0.33713210
H	2.44327803	-5.50810810	0.79888665
H	-2.55794824	-6.51916619	-0.25095525
H	-0.43387170	-1.43083119	-0.11900633

HEH6•

ZORA-(U)BLYP-D3(BJ)/TZ2P

E = -5558.06
H = -5329.75
G = -5377.78
$\boldsymbol{N}_{\mathbf{imag}}$ = 0

C	-0.58669025	-2.28937705	-0.05544178
C	-1.84732366	-3.10180198	-0.05672596
C	-1.75514706	-4.47373357	-0.30808677
N	-0.55727057	-5.11896438	-0.29979057
C	0.59232591	-4.47991491	0.06531963
C	0.63590274	-3.10210653	0.23696114
C	-3.08734588	-2.31640210	0.12191775
C	-2.92594657	-5.38647038	-0.60203853
H	-3.63234301	-4.93439121	-1.30286277
C	1.76343265	-5.41317318	0.28880202
C	1.80574974	-2.34235213	0.74122439
H	-7.53519097	-2.82250751	0.74100321
O	1.70714839	-1.34123341	1.43874217
O	3.00810159	-2.85957290	0.34081451
H	-6.41427437	-4.01798727	1.42896078
C	4.19539711	-2.15288660	0.83325513
C	5.41236946	-2.86655423	0.26842671
H	4.17544877	-2.16417930	1.92866681
H	4.13553342	-1.10867410	0.50694573
O	-3.10743846	-1.09081359	0.18730461
O	-4.22001159	-3.07823083	0.24608969
C	-5.46111934	-2.32981709	0.45641446
H	-5.34939212	-1.71884929	1.35905340
C	-6.58498021	-3.34572195	0.58076718
H	-5.60543523	-1.65100870	-0.39174153
H	6.32411097	-2.36276389	0.61100842
H	5.40155178	-2.85225215	-0.82716811
H	5.44804288	-3.90921759	0.60293719
H	-6.67298156	-3.94878561	-0.32979767
H	-3.48522351	-5.60625774	0.31516395
H	2.46786226	-5.35772548	-0.54955392
H	1.38462011	-6.43501975	0.36441703
H	2.32236557	-5.15504862	1.19272612
H	-2.53342006	-6.32261115	-1.00633878
C	-0.45310187	-1.60831155	-1.49911216
H	-0.66400472	-1.45680212	0.65207380
H	-1.39923948	-1.08818690	-1.67180568
H	-0.36657183	-2.41706541	-2.23517897
C	0.71311280	-0.62399150	-1.63708083
H	0.65627732	-0.12625886	-2.61318353
H	1.68593330	-1.12380997	-1.58028355
H	0.67914508	0.14599565	-0.85912601

HP6

ZORA-(U)BLYP-D3(BJ)/TZ2P

E = -5506.51

H = -5284.33

G = -5333.10

N_{imag} = 0

C	-0.25370095	-2.66058567	-0.46179963
C	-1.54041229	-3.20665699	-0.64317087
C	-1.68082176	-4.52468107	-1.11937753
N	-0.61212013	-5.29568752	-1.39294438
C	0.62567509	-4.80820044	-1.21087543
C	0.84156260	-3.49305489	-0.74618883
C	-2.76064512	-2.37154234	-0.37972144
C	-3.03069750	-5.15459469	-1.38006264
H	-3.73593121	-4.44168494	-1.82117930
C	1.77045301	-5.74285941	-1.52364422
C	2.25246651	-3.00042147	-0.60037100
H	-6.45158422	-2.53080985	2.22690000
O	3.05042743	-2.89368438	-1.51416541
O	2.54452329	-2.70474444	0.70187797
H	-4.90475665	-3.12035793	2.87487324
C	3.89130866	-2.17262272	0.94995393
C	3.98248098	-1.84600071	2.43132881
H	4.03390957	-1.28892500	0.31822947
H	4.62205342	-2.92863471	0.64281012
O	-3.04341606	-1.33548207	-0.95620682
O	-3.53130056	-2.92720751	0.60070318
C	-4.78438976	-2.22265922	0.90501000
H	-4.53519409	-1.21211856	1.24717683
C	-5.51093048	-3.03081993	1.96675725
H	-5.36210124	-2.13291808	-0.02123300
H	4.98030406	-1.45241330	2.65866789
H	3.81727885	-2.74099244	3.04133953
H	3.24102518	-1.09032679	2.71288784
H	-5.74451225	-4.03728321	1.60268417
H	-3.47228004	-5.51626860	-0.44301768
H	2.31403642	-5.40103344	-2.41240243
H	1.37533085	-6.74487821	-1.70444539
H	2.49484840	-5.77921268	-0.70063164
H	-2.90146682	-6.00422927	-2.05482987
C	-0.07427552	-1.24190456	0.04235286
H	-1.30546477	-1.45514365	1.84523620
H	-0.78242019	-0.59158205	-0.47930796
H	0.93062601	-0.88698680	-0.20465425
C	-0.29599163	-1.13119076	1.56812305
H	-0.17084502	-0.09293135	1.89680460
H	0.42256393	-1.75758068	2.10589127

Table S7. Cartesian coordinates (in Å), energies (in kcal mol^{-1}), and number of imaginary frequencies of all stationary points, computed at COSMO(DMF)-ZORA-(U)BLYP-D3(BJ)/TZ2P. Energies (in kcal mol^{-1}) at COSMO(toluene)-PBE0-D3/TZ2P//BP86/TZ2P are also provided.

2-trans anion

COSMO(DMF)-ZORA-(U)M06-2X/TZ2P//COSMO(DMF)-ZORA-(U)BLYP-D3(BJ)/TZ2P

E = -7924.54

G = -7760.92

COSMO(DMF)-ZORA-(U)BLYP-D3(BJ)/TZ2P

E = -5590.31

G = -5426.69

N_{imag} = 0

C	-3.08247221	-0.16911528	-1.22930354
C	-4.23017126	-0.55884356	-1.92267020
C	-4.50056168	-1.92747190	-2.08780576
C	-3.62940025	-2.89361038	-1.56455057
C	-2.47514199	-2.50299868	-0.86654678
C	-2.21239423	-1.14333920	-0.70320986
H	-4.90548345	0.18580752	-2.34138949
H	-5.38877097	-2.24067909	-2.63306073
H	-3.84709948	-3.95055171	-1.70485988
H	-1.78988896	-3.24723553	-0.46529897
C	-1.05981034	-0.46286890	0.01079349
C	-2.55586589	1.22092894	-0.92792916
C	1.00874468	2.34083645	-0.19975444
H	-2.72628444	1.93774013	-1.74012837
H	-3.04080784	1.62703688	-0.02588923
C	-1.03988828	0.97484557	-0.64168449
H	-0.54201447	0.84458125	-1.61435676
C	-0.28231886	1.99483662	0.14062314
H	-0.63178849	2.23419851	1.14350611
O	1.69559934	2.04702311	-1.22670721
O	1.63446882	3.21710416	0.78607819
C	3.05501520	3.00209519	0.92861717
H	3.58674008	3.17128290	-0.01516980
H	3.39972224	3.72236995	1.67748451
H	3.26074328	1.98017794	1.27528683
N	0.20945617	-1.18314347	-0.06076789
H	2.77251996	-1.39354001	-0.64550739
H	0.53522478	-1.27197532	-1.02123208
C	1.25946282	-0.93386302	0.82912959
C	1.05130201	-0.56661880	2.17738425
C	2.13299858	-0.39849382	3.04301203
C	3.45065819	-0.58902459	2.60374077
C	3.66452642	-0.95808748	1.26833311
C	2.59114437	-1.12668481	0.39436042

H	0.04577079	-0.39639027	2.54789977
H	1.94160290	-0.10475618	4.07367287
H	4.28837236	-0.44797850	3.28233087
H	4.67828024	-1.10327605	0.89905175
H	-1.32498825	-0.35333919	1.06984583

References

[1] C. K. Prier, D. A. Rankic, D. W. C. MacMillan, *Chem. Rev.* **2013**, *113*, 5322-5363.

[2] J. A. Leitch, Angel L. Fuentes de Arriba, J. Tan, O. Hoff, C. M. Martínez, D. J. Dixon, *Chem. Sci.* **2018**, *9*, 6653-6658.

[3] J. Dong, F. Yue, W. Xu, H. Song, Y. Liu, Q. Wang, *Green Chem.* **2020**, *22*, 5599-5604.

[4] W. Guan-Wu, X. Jing-Jing, M. Chun-Bao, W. Xue-Liang, *Bull. Chem. Soc. Jpn.* **2006**, *79*, 454-459.

[5] Á. Gutiérrez-Bonet, C. Remeur, J. K. Matsui, G. A. Molander, *J. Am. Chem. Soc.* **2017**, *139*, 12251-12258.

[6] D. Zhang, L. -Z. Wu, L. Zhou, X. Han, Q. –Z. Yang, L. –P. Zhang, C. –H. Tung, *J. Am. Chem. Soc.* **2004**, *126*, 3440-3441.

[7] M. –H. Larraufie, R. Pellet, L. Fensterbank, J. –P. Goddard, E. Lacote, M. Malacria, C. Ollivier, *Angew. Chem. Int. Ed.* **2011**, *50*, 4463-4466.

[8] U. K. Pandit, J. B. Steevens, F. R. Mas Cabre, *Bioorg. Chem.* **1973**, *2*, 293-300.

[9] H. Cai, J. C. Fishbein, *Tetrahedron* **1997**, *53*, 10671-10676.

[10] T. Kippo, T. Fukuyama, I. Ryu, *Org. Lett.* **2011**, *13*, 3864-3867.

[11] W. F. K. Schnatter, Ö. Almarsson, T. C. Bruice, *Tetrahedron* **1991**, *47*, 8687-8700.

[12] J. M. Aurrecoechea, B. López, A. Fernández, A. Arrieta, F. P. Cossío, *J. Org. Chem.* **1997**, *62*, 1125-1135.

[13] H. O. House, H. Babad, R. B. Toothill, A. W. Noltes, *J. Org. Chem.* **1962**, *27*, 4141-4146.

[14] D. C. Miller, G. J. Choi, H. S. Orbe, R. R. Knowles, *J. Am. Chem. Soc.* **2015**, *137*, 13492-13495.

[15] J. Zindel, A. de Meijere, *Synthesis* **1994**, *1994*, 190-194.

[16] S. Alazet, F. Le Vaillant, S. Nicolai, T. Courant, J. Waser, *Chem. Eur. J.* **2017**, *23*, 9501-9504.

[17] S. Sathyamoorthi, J. Du Bois, *Org. Lett.* **2016**, *18*, 6308-6311.

[18] T. D. Svejstrup, A. Chatterjee, D. Schekin, T. Wagner, J. Zach, M. J. Johansson, G. Bergonzini, B. König, *ChemPhotoChem* **2021**, DOI: 10.1002/cptc.202100059.

[19] a) G. te Velde, F. M. Bickelhaupt, E. J. Baerends, C. Fonseca Guerra, S. J. A. van Gisbergen, J. G. Snijders, T. Ziegler, *J. Comput. Chem.* **2001**, *22*, 931-967; b) C. Fonseca Guerra, J. G. Snijders, G. te Velde, E. J. Baerends, *Theor. Chem. Acc.* **1998**, *99*, 391-403; ADF2018.105, SCM Theoretical Chemistry, Vrije Universiteit: Amsterdam (The Netherlands), **2017**. http://www.scm.com.

[20] a) J. C. Slater, *Quantum Theory of Molecules and Solids*, McGraw-Hill, New York, **1974**; b) A. D. Becke, *J. Chem. Phys.* **1986**, *84*, 4524-4529; c) A. D. Becke, *Phys. Rev. A* **1988**, *38*, 3098-3100; d) C. Lee, W. Yang, R. G. Parr, *Phys. Rev. B* **1988**, *37*, 785-789.

[21] E. van Lenthe, E. J. Baerends, *J. Comput. Chem.* **2003**, *24*, 1142-1156.

[22] T. Rogova, P. Gabriel, S. Zavitsanou, J. A. Leitch, F. Duarte, D. J. Dixon, *ACS Catal.* **2020**, *10*, 11438-11447.

[23] a) A. Klamt, G. Schüürmann, *J. Chem. Soc. Perkin Trans. 2* **1993**, 799-805; b) A. Klamt, *J. Phys. Chem.* **1995**, *99*, 2224-2235; c) A. Klamt, V. Jonas, *J. Chem. Phys.* **1996**, *105*, 9972-9981; d) C. C. Pye, T. Ziegler, *Theor. Chem. Acc.* **1999**, *101*, 396-408.

[24] a) S. Grimme, J. Antony, S. Ehrlich, H. Krieg, *J. Chem. Phys.* **2010**, *132*, 154104; b) A. D. Becke, E. R. Johnson, *J. Chem. Phys.* **2005**, *123*, 154101.

[25] a) E. van Lenthe, E. J. Baerends, J. G. Snijders, *J. Chem. Phys.* **1993**, *99*, 4597-4610; b) E. van Lenthe, E. J. Baerends, J. G. Snijders, *J. Chem. Phys.* **1994**, *101*, 9783-9792.

[26] C. Y. Legault, *CYLView* (Unversité de Sherbrooke, Sherbrooke, QC, Canada), 1.0b, **2009**.

[27] Y. Zhao, D. G. Truhlar, *Theor. Chem. Acc.* **2008**, *120*, 215-241.

NMR Spectra

4-d1-HE6

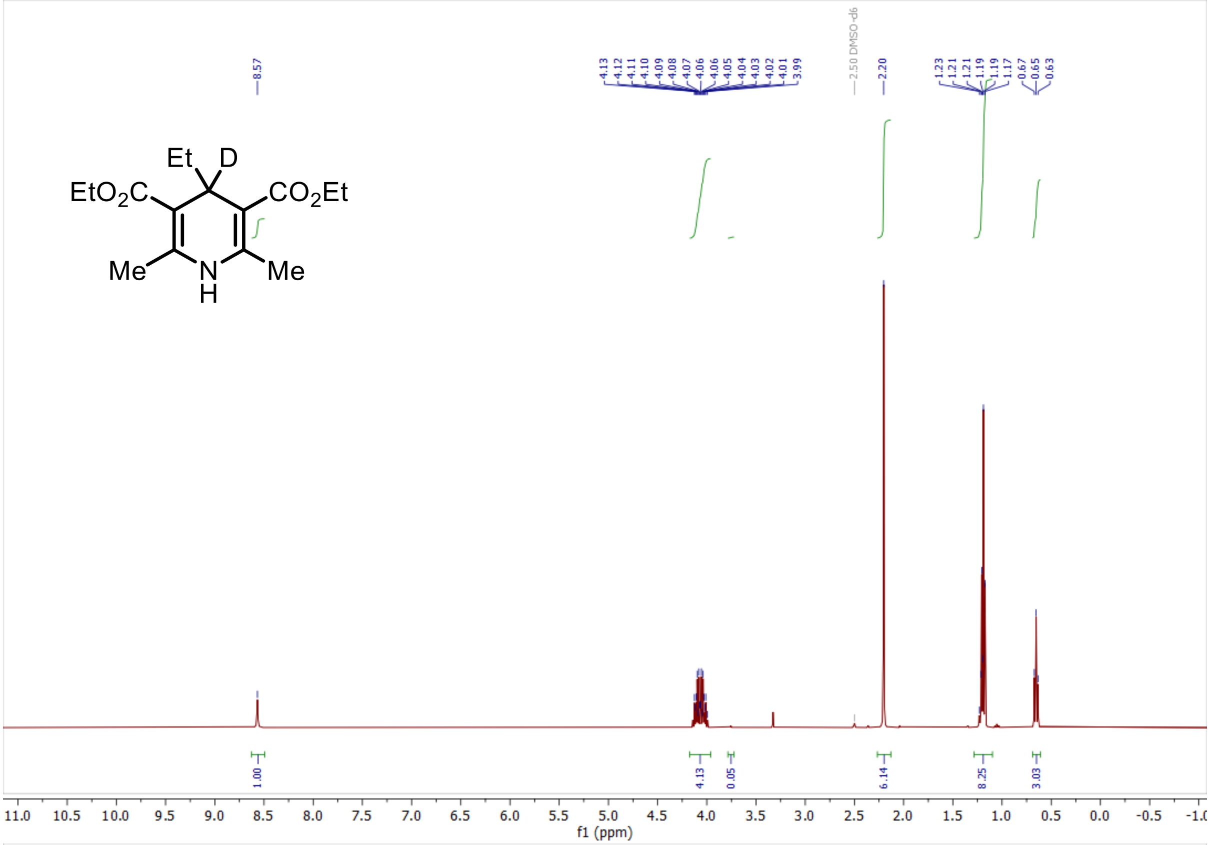

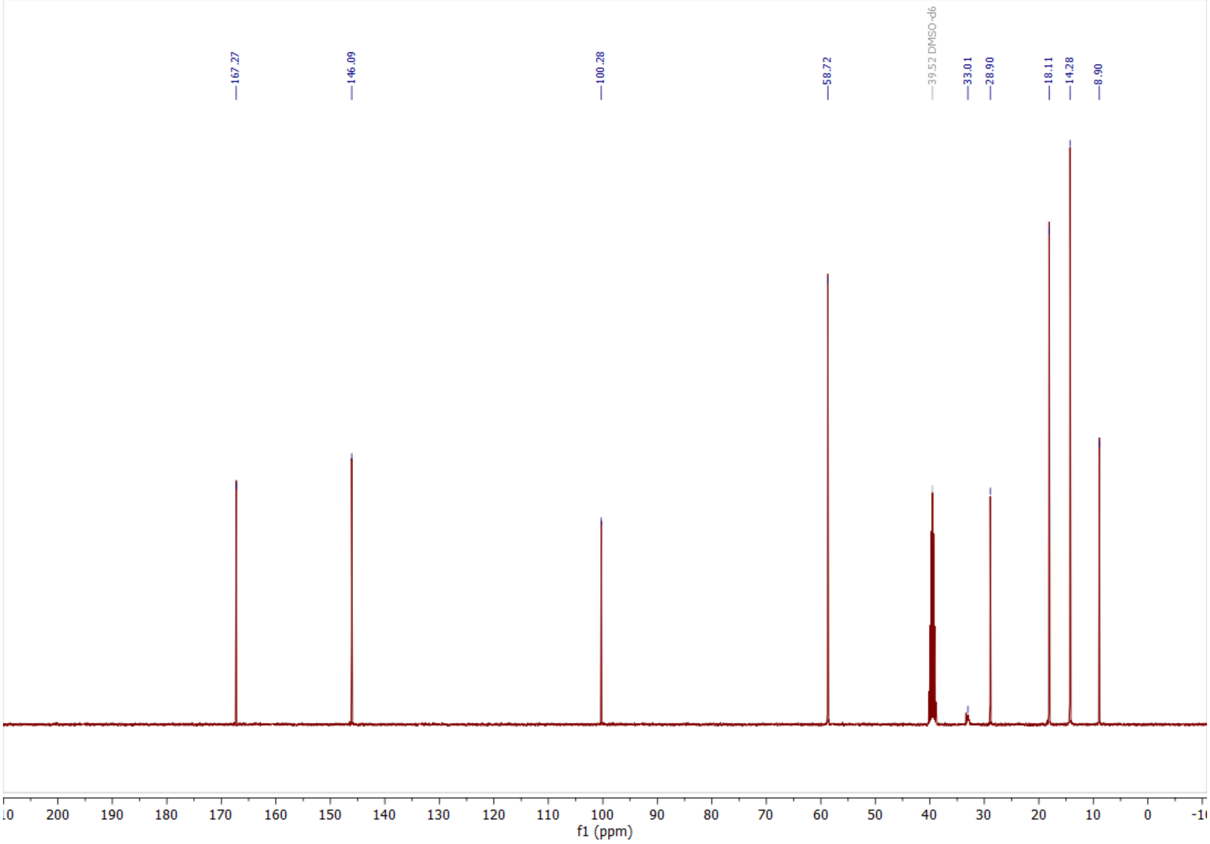

^{2}H NMR (H-DMSO):

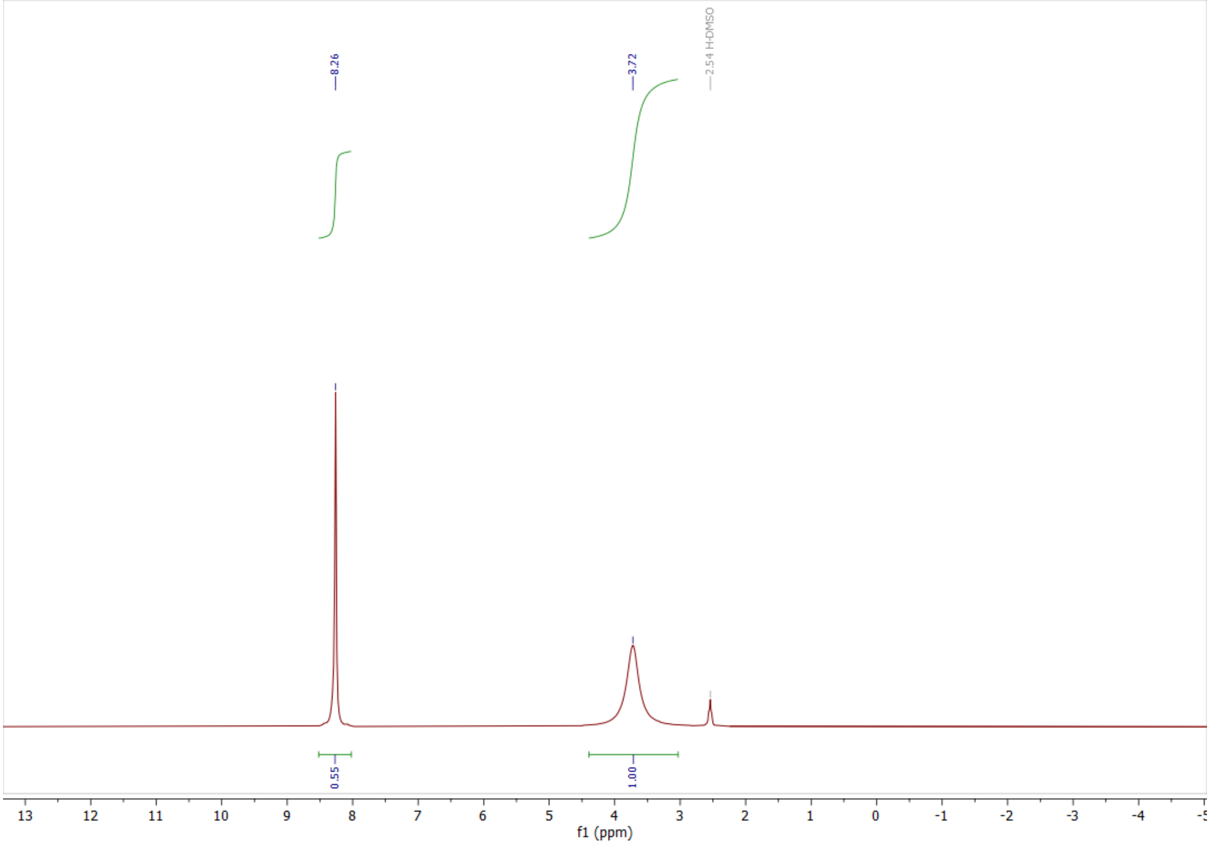

2a

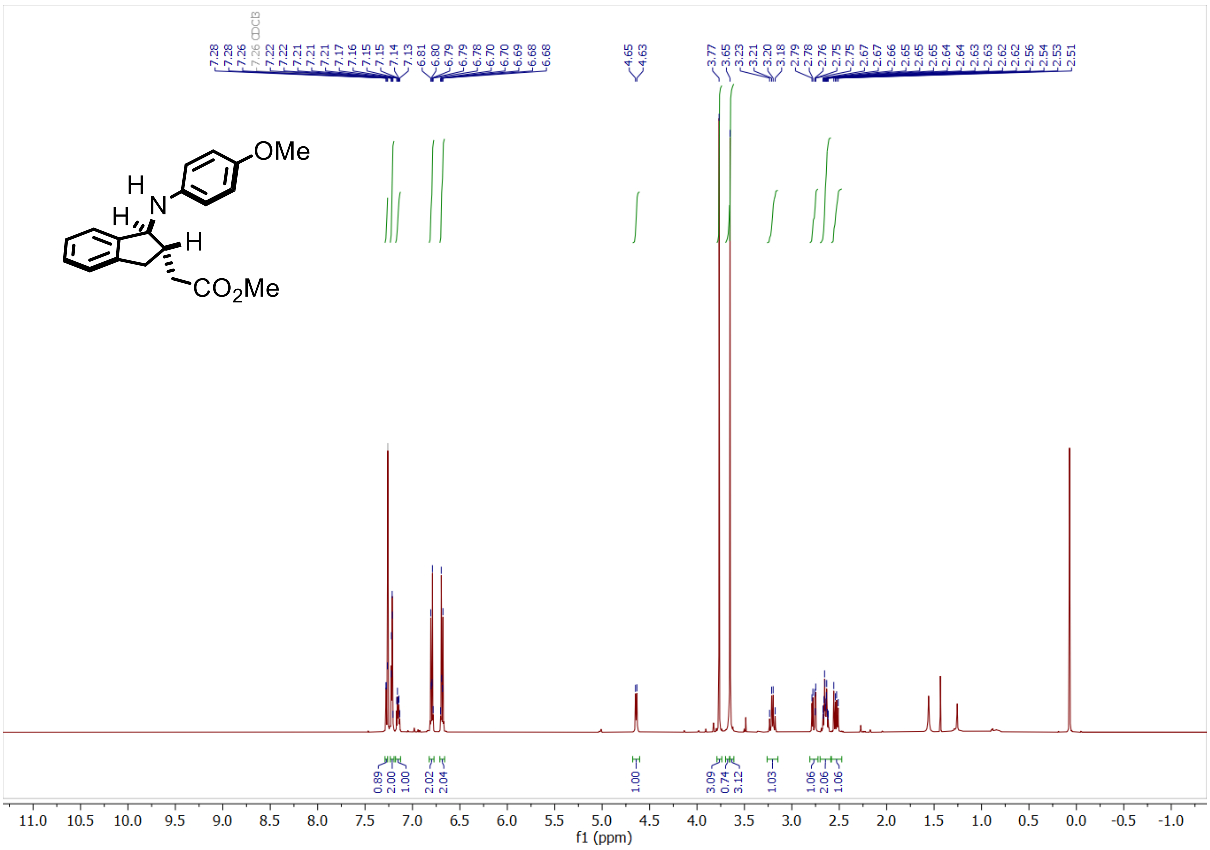

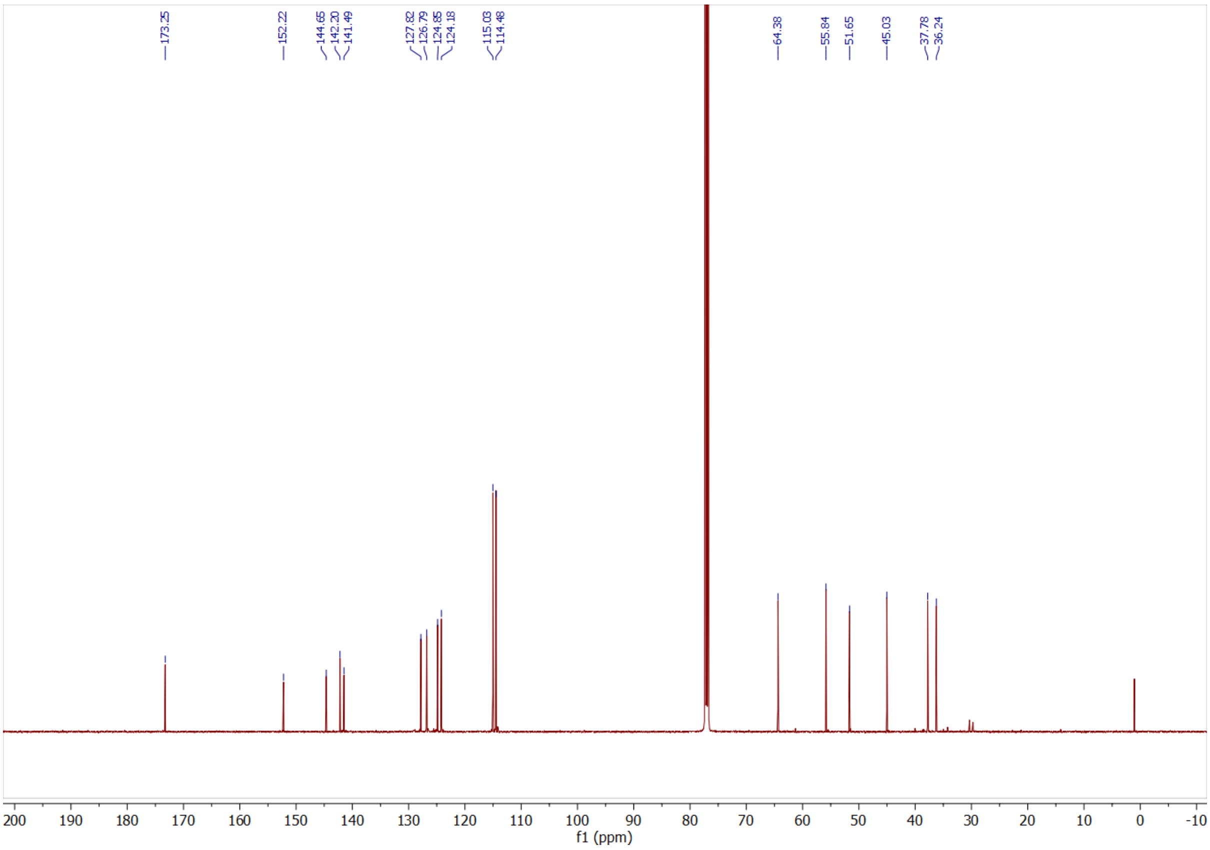

2b-*trans*

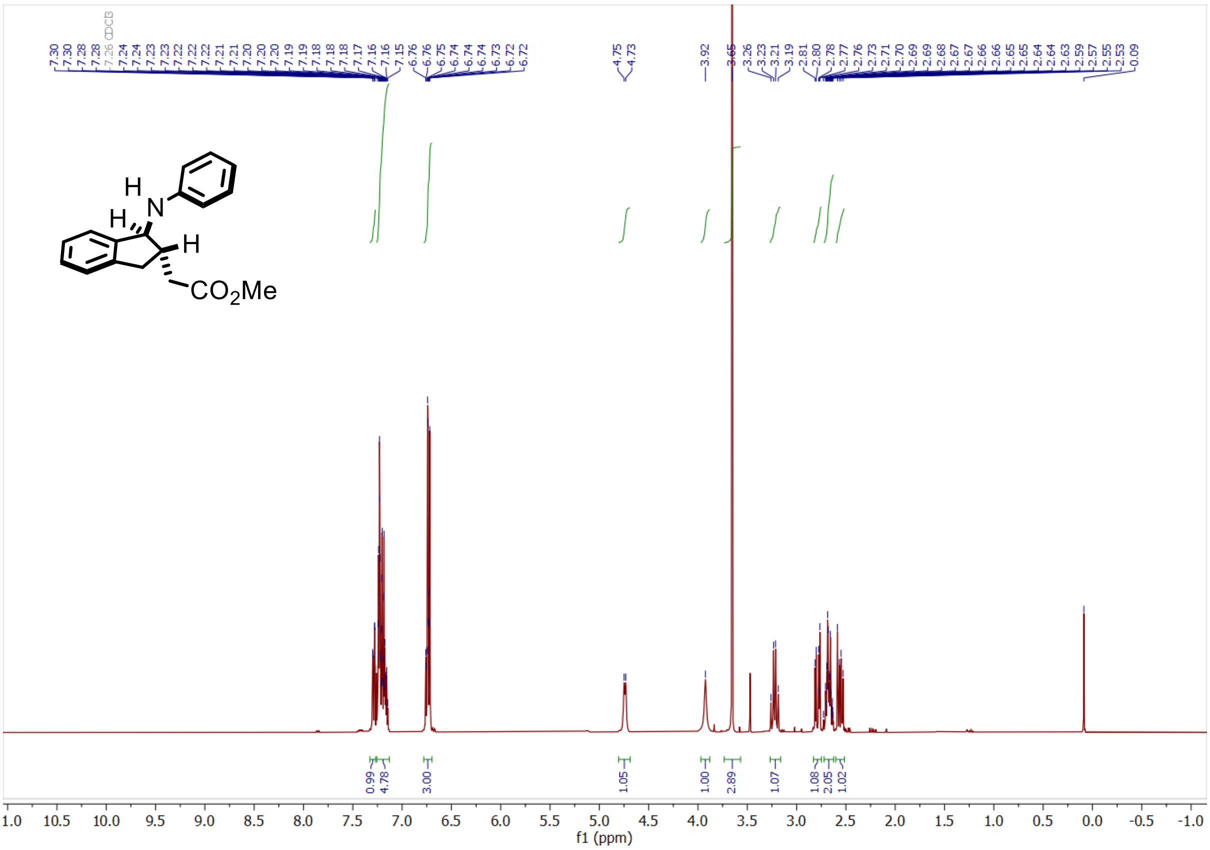
7.30
7.30
7.28
7.26 CDCl3
7.24
7.24
7.23
7.23
7.22
7.22
7.22
7.21
7.21
7.20
7.20
7.19
7.19
7.18
7.18
7.18
7.17
7.16
7.16
7.15
6.76
6.76
6.75
6.74
6.74
6.74
6.73
6.72
6.72
4.75
4.73
3.92
3.65
3.26
3.23
3.21
3.19
2.81
2.80
2.78
2.77
2.76
2.73
2.71
2.70
2.69
2.69
2.68
2.67
2.67
2.66
2.66
2.65
2.65
2.64
2.64
2.63
2.59
2.57
2.55
2.53
0.09
H
N
H
H
CO2Me
0.99
4.78
3.00
1.05
1.00
2.89
1.07
1.08
2.05
1.02
f1 (ppm)

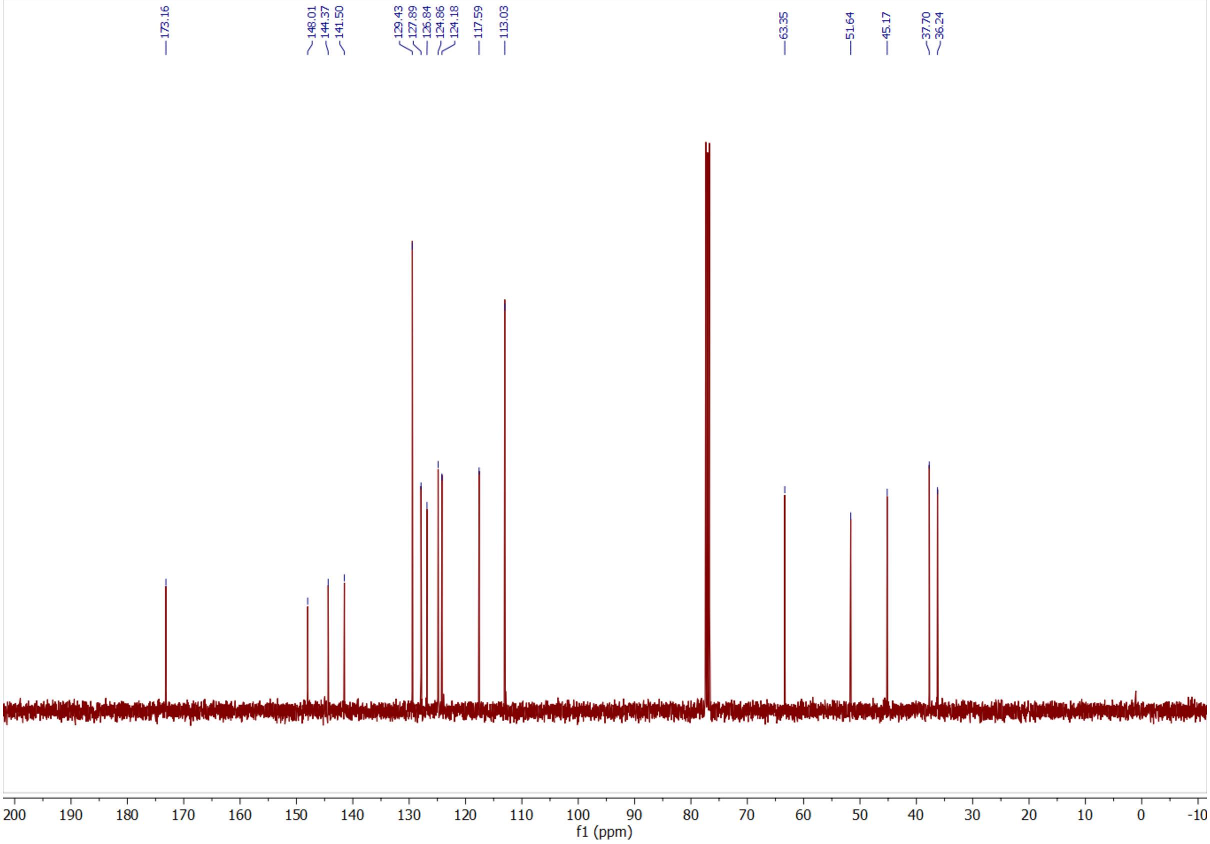
173.16
148.01
144.37
141.50
129.43
127.89
126.84
124.86
124.18
117.59
113.03
63.35
51.64
45.17
37.70
36.24
f1 (ppm)

2b-*trans*-HCl

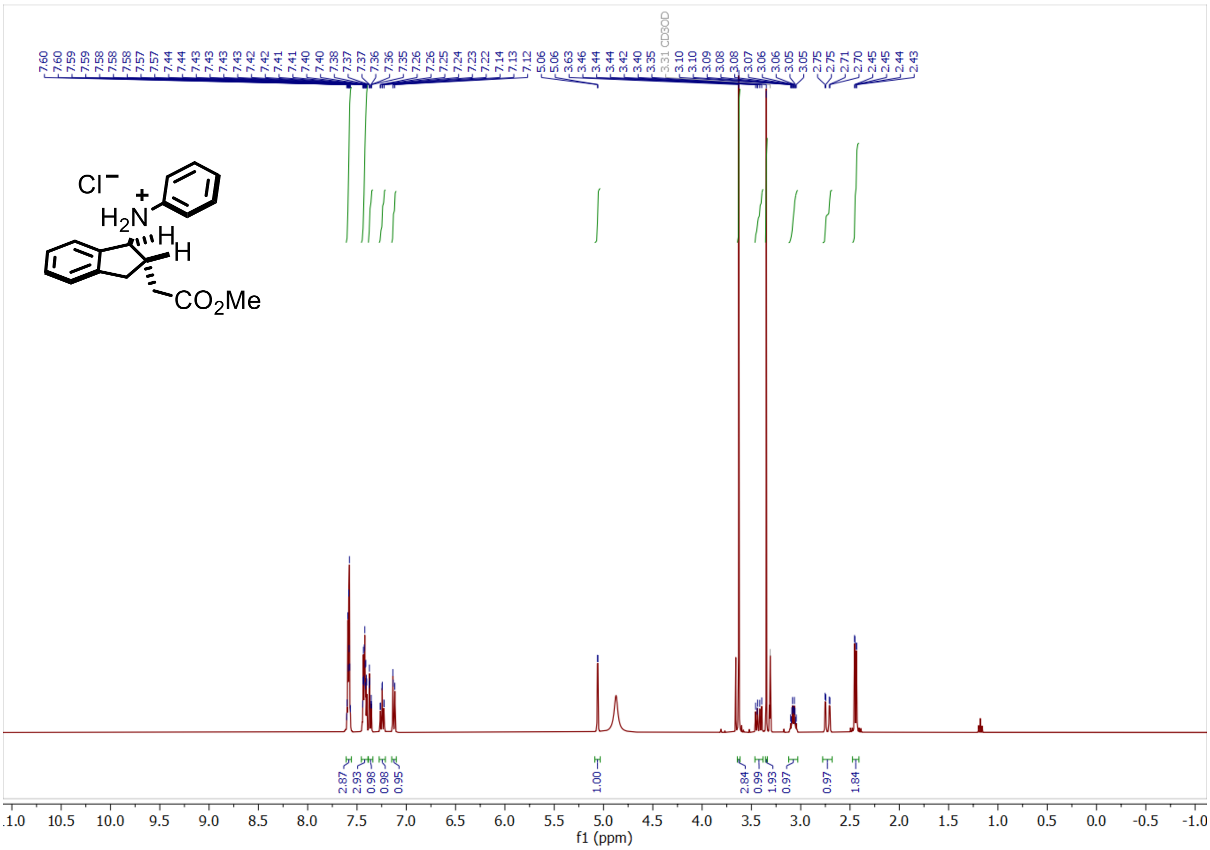

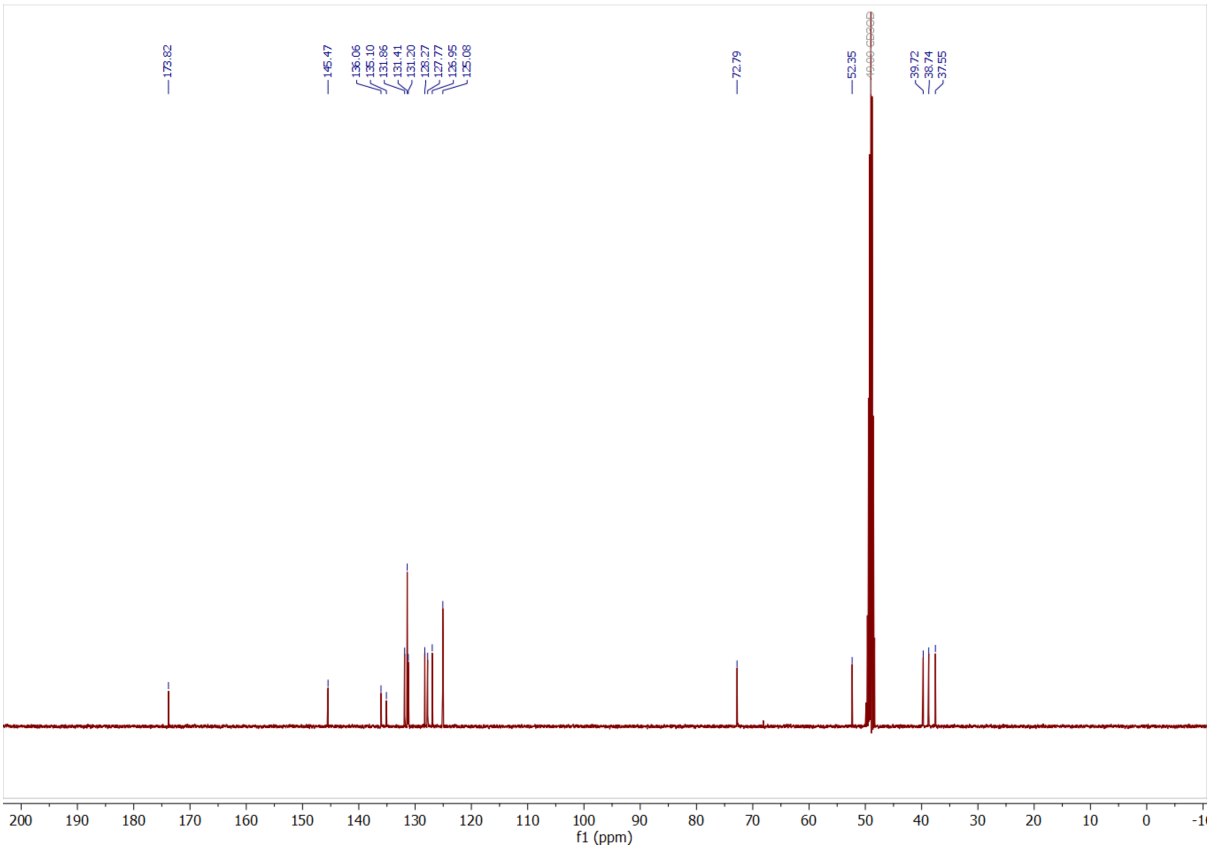

2b-*cis*

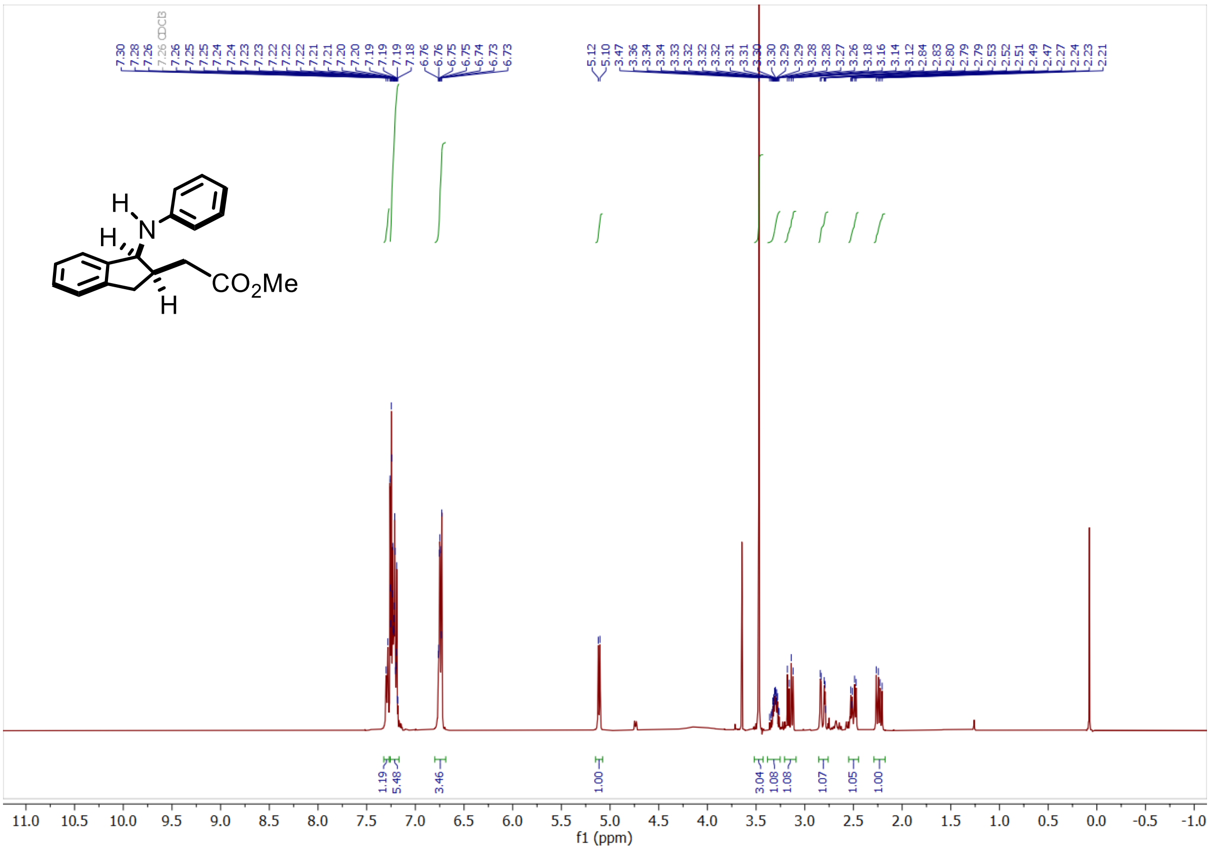

7.30 7.28 7.26 CDCl3 7.26 7.25 7.25 7.24 7.24 7.23 7.23 7.22 7.22 7.22 7.21 7.21 7.20 7.20 7.19 7.19 7.19 7.18 6.76 6.76 6.75 6.75 6.74 6.73 6.73
5.12 5.10 3.47 3.36 3.34 3.34 3.33 3.32 3.32 3.32 3.31 3.31 3.30 3.30 3.29 3.29 3.28 3.28 3.27 3.26 3.18 3.16 3.14 3.12 2.84 2.83 2.80 2.79 2.79 2.53 2.52 2.51 2.49 2.47 2.27 2.24 2.23 2.21
H
H
N
CO2Me
H
1.19 5.48 3.46 1.00 3.04 1.08 1.08 1.07 1.05 1.00
11.0 10.5 10.0 9.5 9.0 8.5 8.0 7.5 7.0 6.5 6.0 5.5 5.0 4.5 4.0 3.5 3.0 2.5 2.0 1.5 1.0 0.5 0.0 -0.5 -1.0
f1 (ppm)

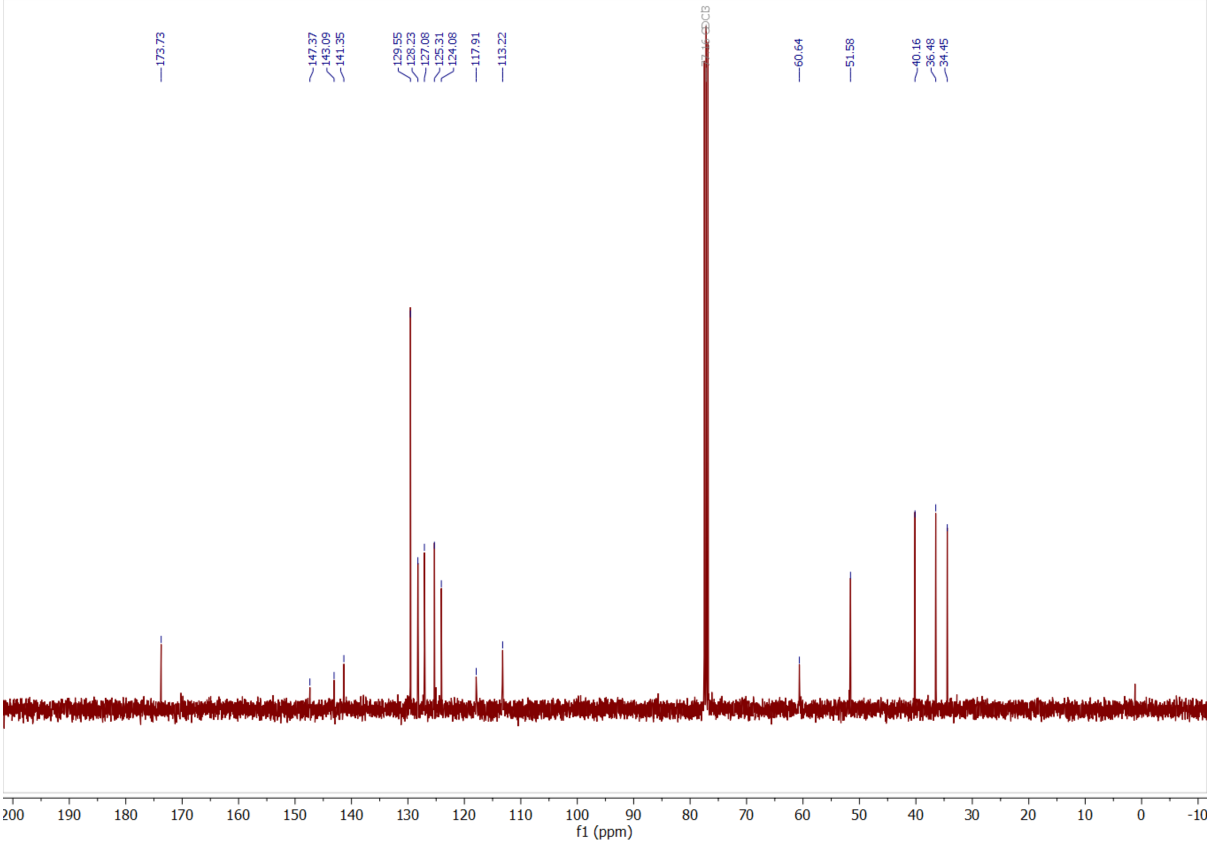

173.73
147.37 143.09 141.35
129.55 128.23 127.08 125.31 124.08
117.91
113.22
CDCl3
60.64
51.58
40.16 36.48 34.45
200 190 180 170 160 150 140 130 120 110 100 90 80 70 60 50 40 30 20 10 0 -10
f1 (ppm)

2b-*cis*-lactam

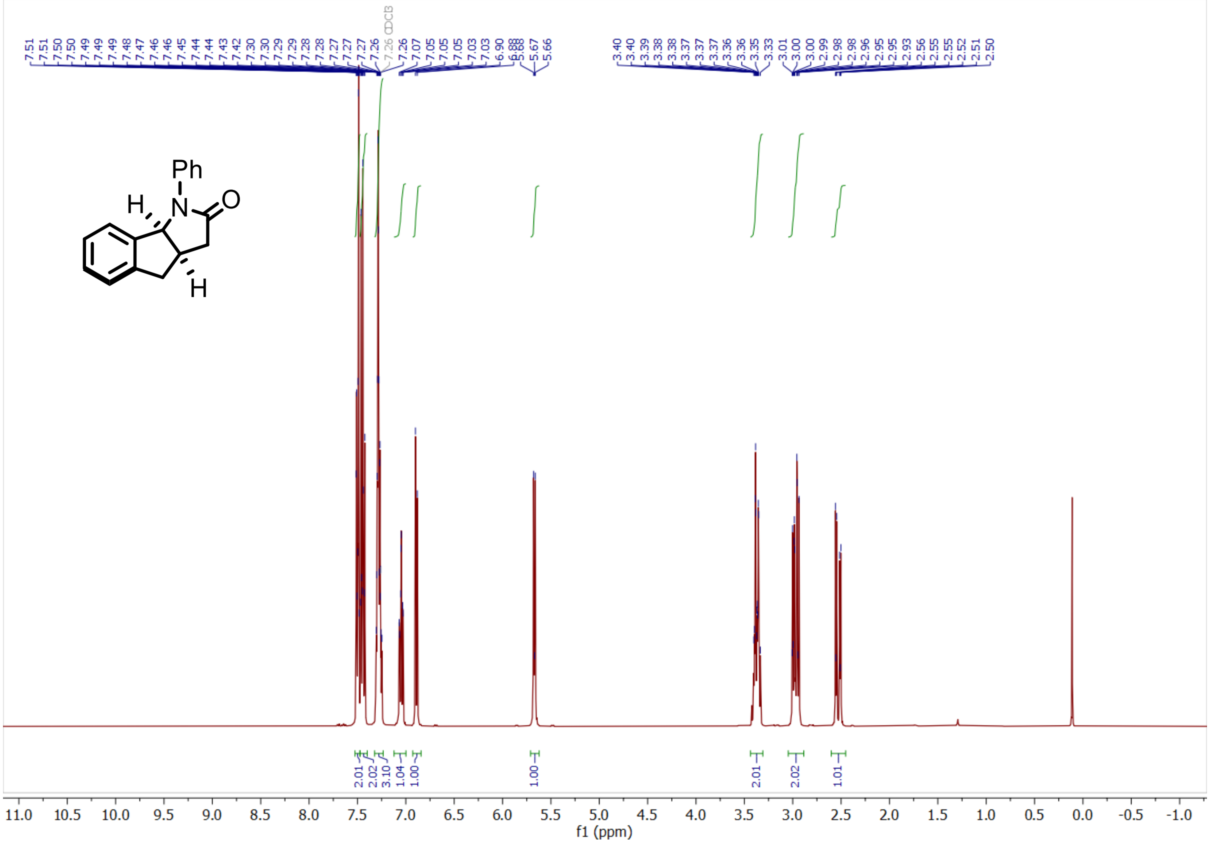

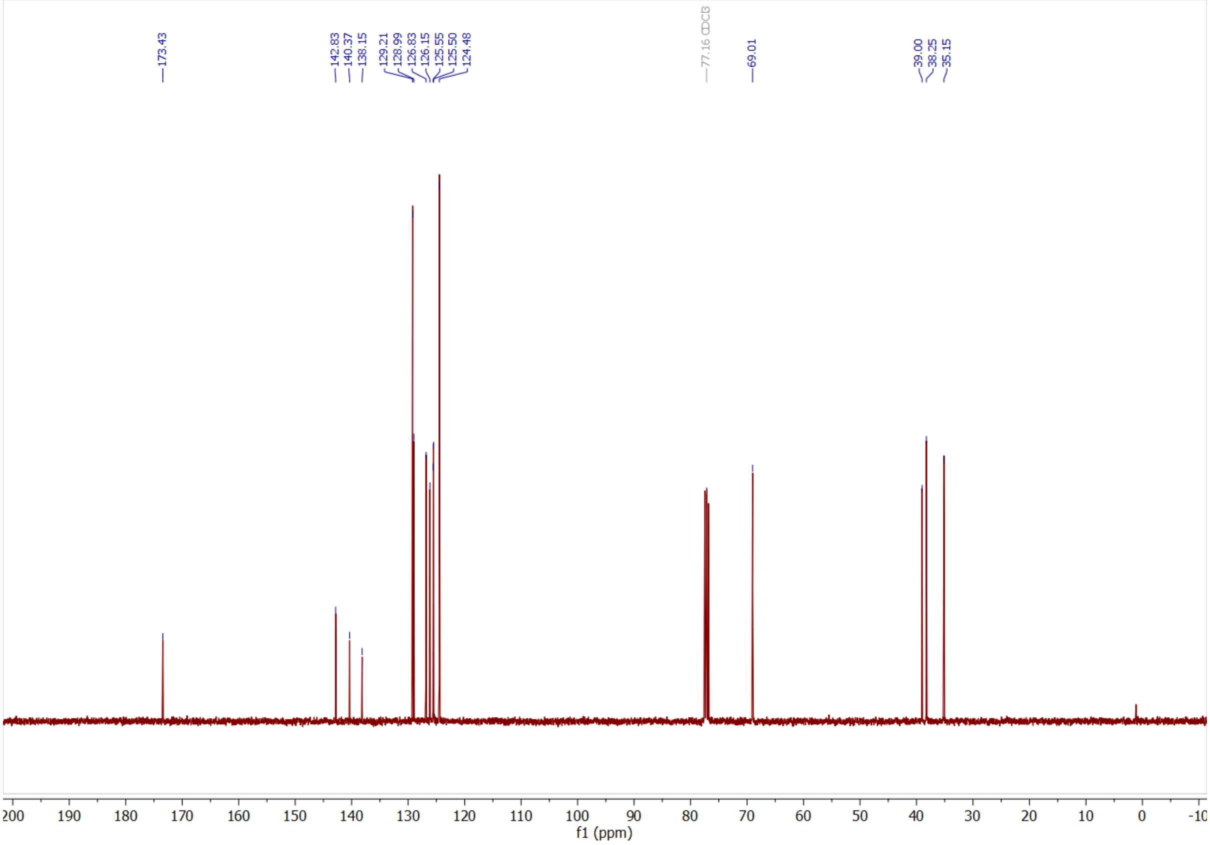

2c

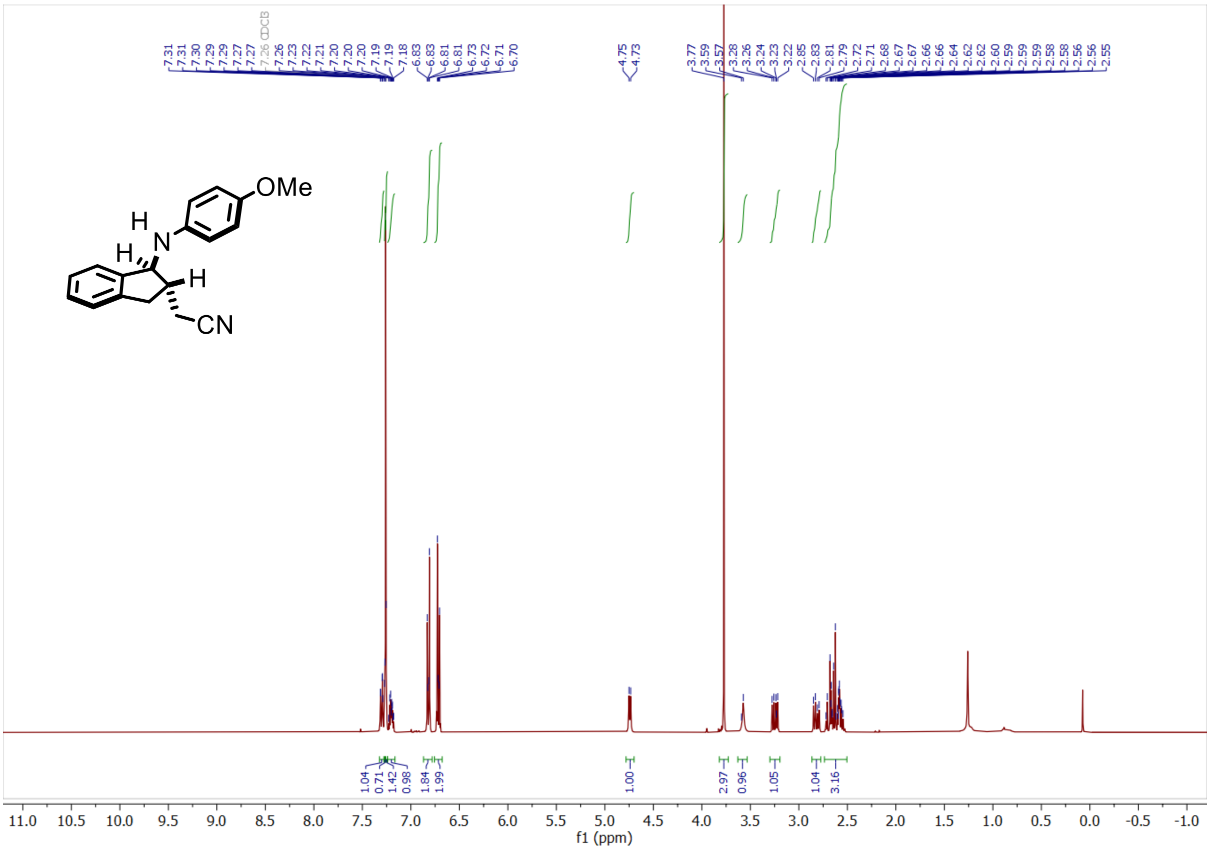
OMe
H
N
H
H
CN
7.31 7.31 7.30 7.29 7.29 7.27 7.27 7.26 CDCl3 7.26 7.23 7.22 7.21 7.20 7.20 7.20 7.19 7.19 7.18 6.83 6.83 6.81 6.81 6.73 6.72 6.71 6.70
4.75 4.73
3.77 3.59 3.57 3.28 3.26 3.24 3.23 3.22 2.85 2.83 2.81 2.79 2.72 2.71 2.68 2.67 2.67 2.66 2.66 2.64 2.62 2.62 2.60 2.59 2.59 2.59 2.58 2.58 2.56 2.56 2.55
1.04 0.71 1.42 0.98 1.84 1.99 1.00 2.97 0.96 1.05 1.04 3.16
11.0 10.5 10.0 9.5 9.0 8.5 8.0 7.5 7.0 6.5 6.0 5.5 5.0 4.5 4.0 3.5 3.0 2.5 2.0 1.5 1.0 0.5 0.0 -0.5 -1.0
f1 (ppm)

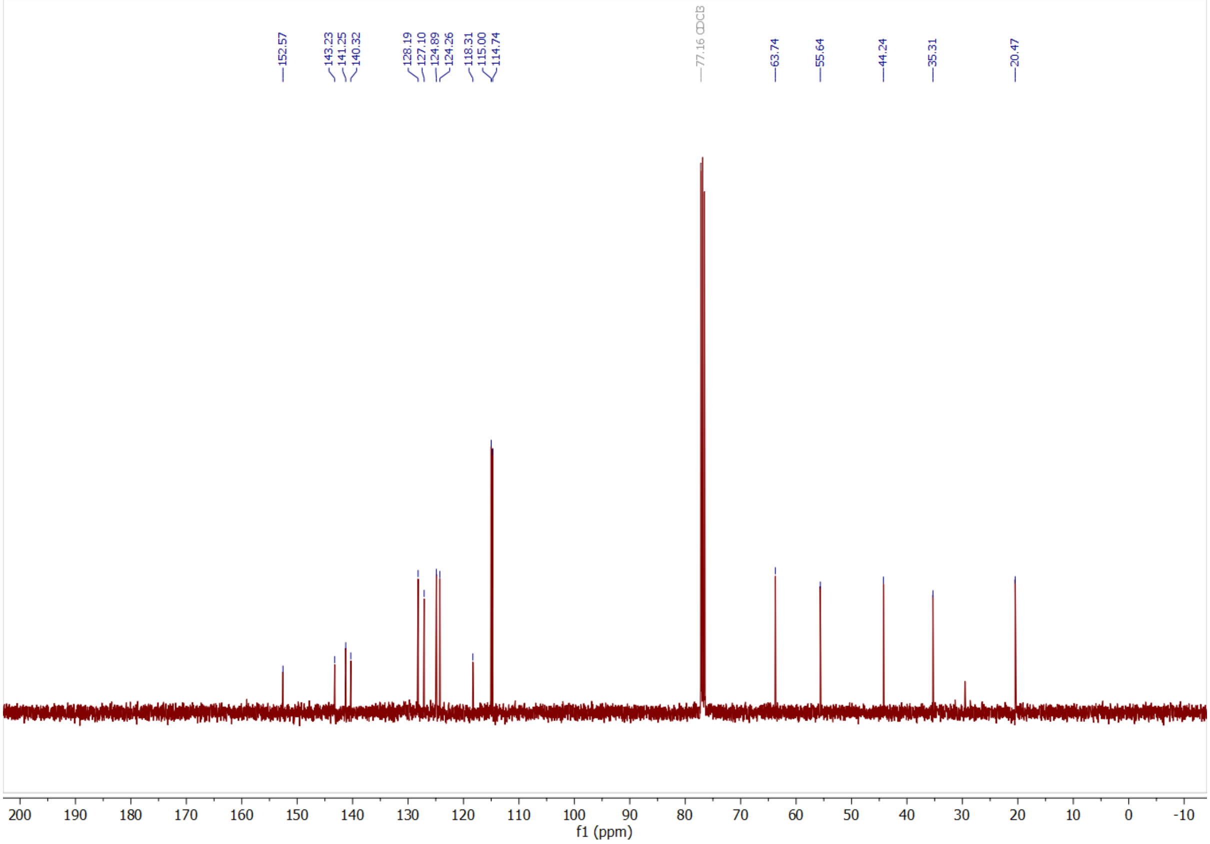
152.57
143.23 141.25 140.32
128.19 127.10 124.89 124.26 118.31 115.00 114.74
77.16 CDCl3
63.74
55.64
44.24
35.31
20.47
200 190 180 170 160 150 140 130 120 110 100 90 80 70 60 50 40 30 20 10 0 -10
f1 (ppm)

2d

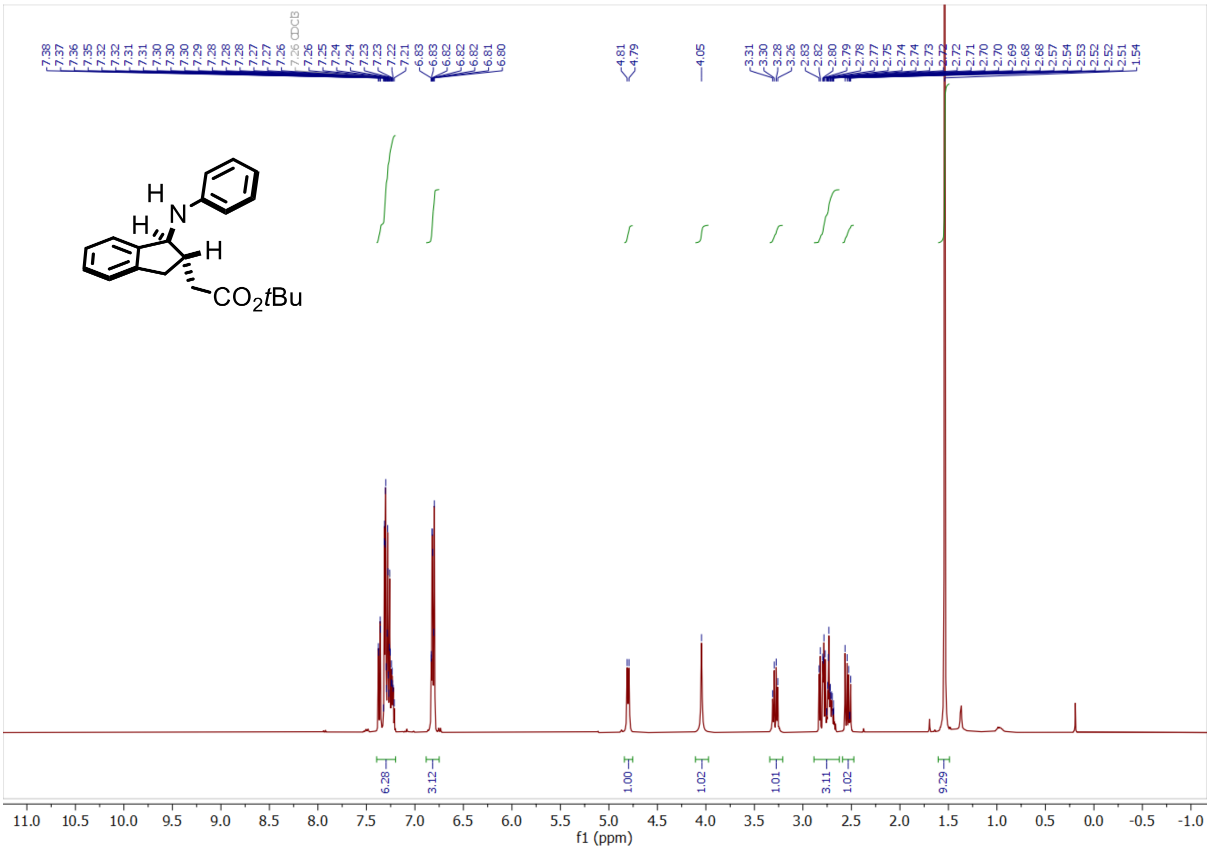

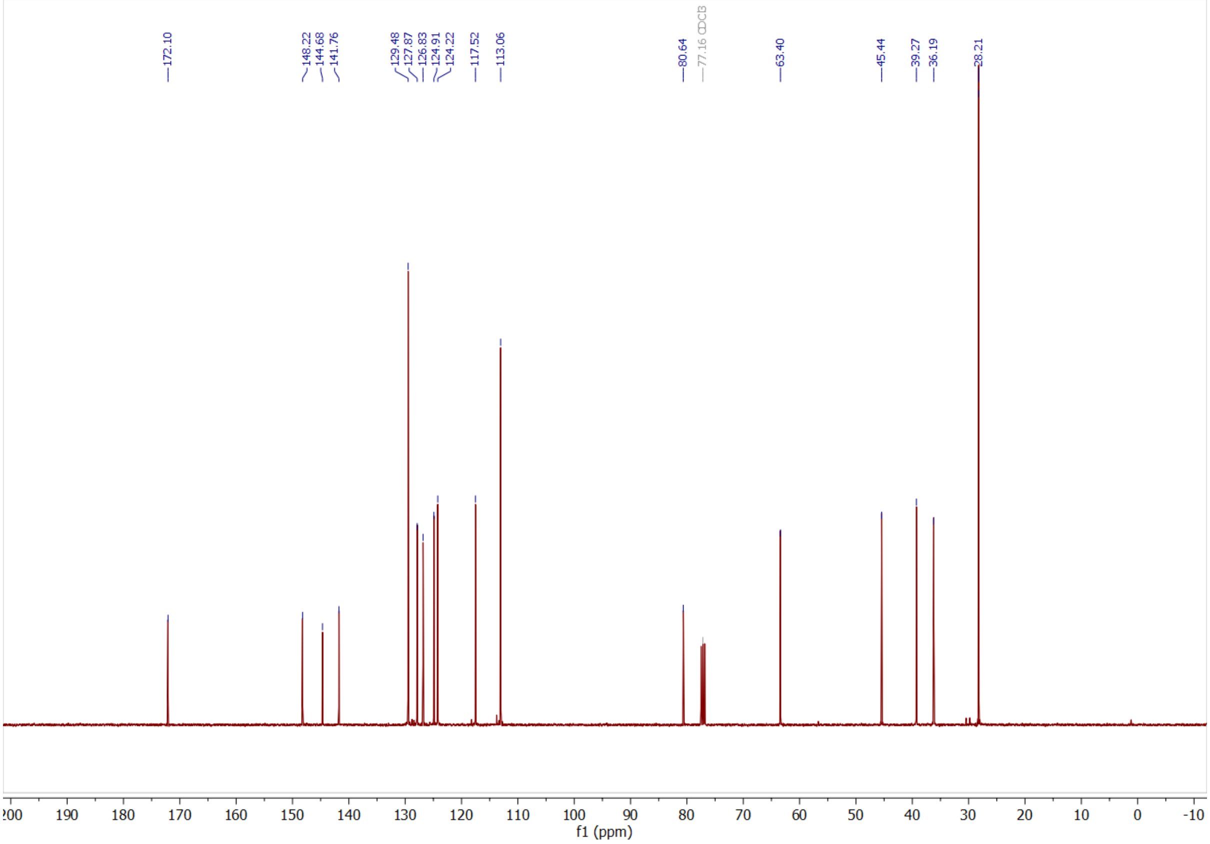

2e

Note: the product was obtained as a 9:1 inseparable mixture of **2e** and its analogue **3e**.

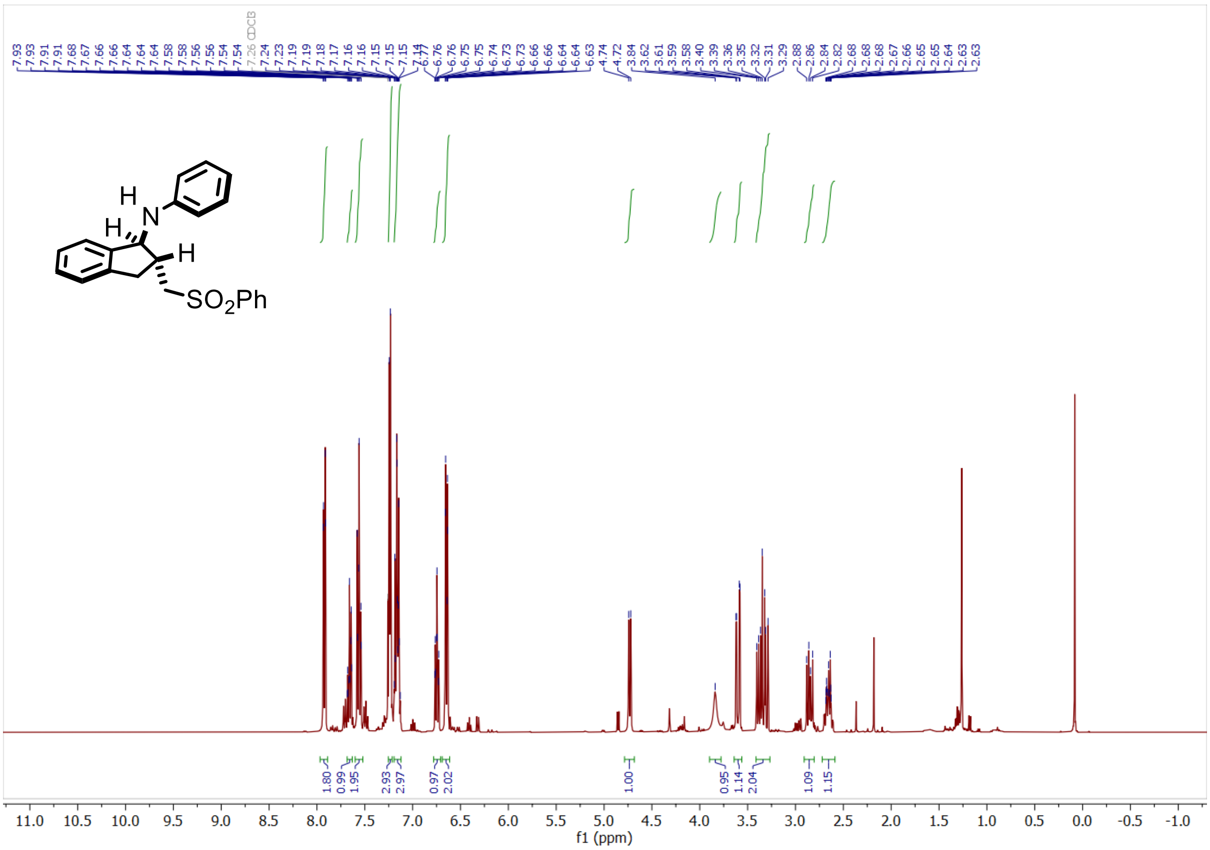

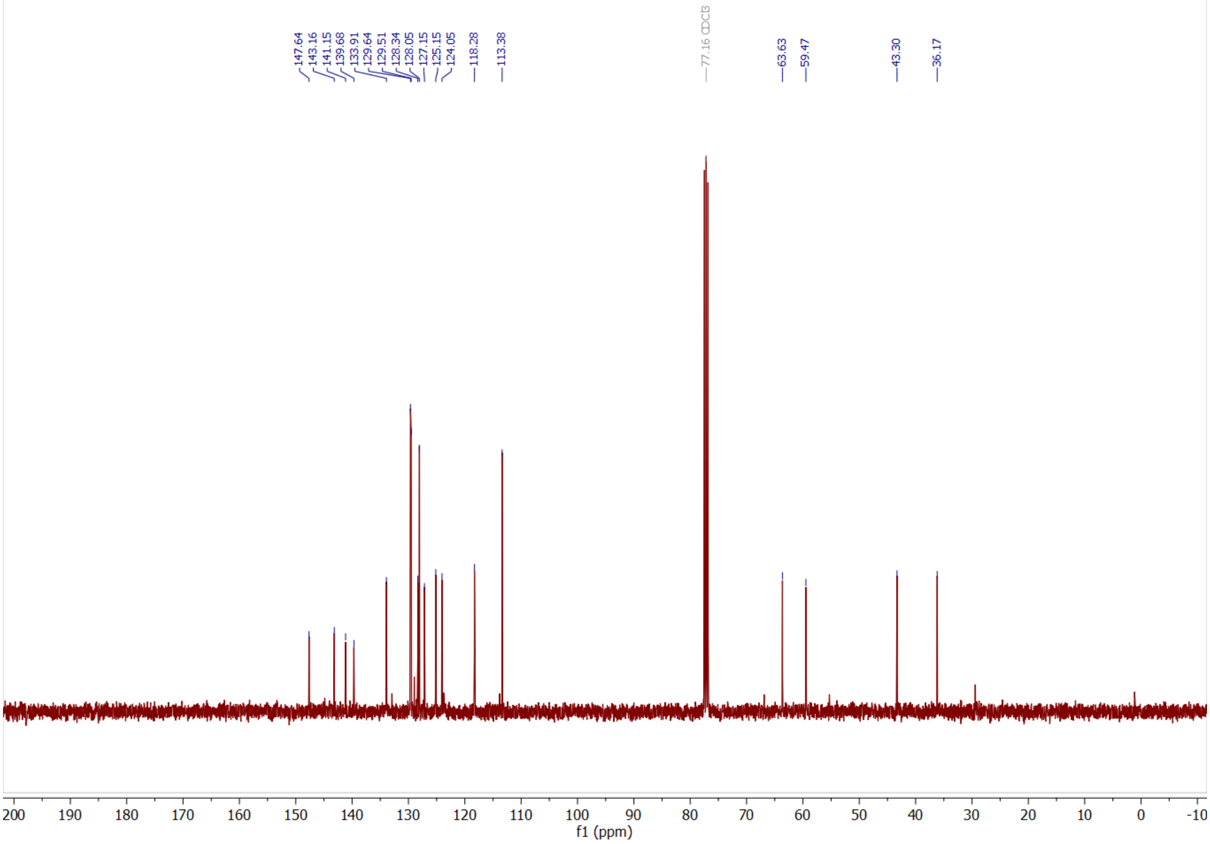

2f

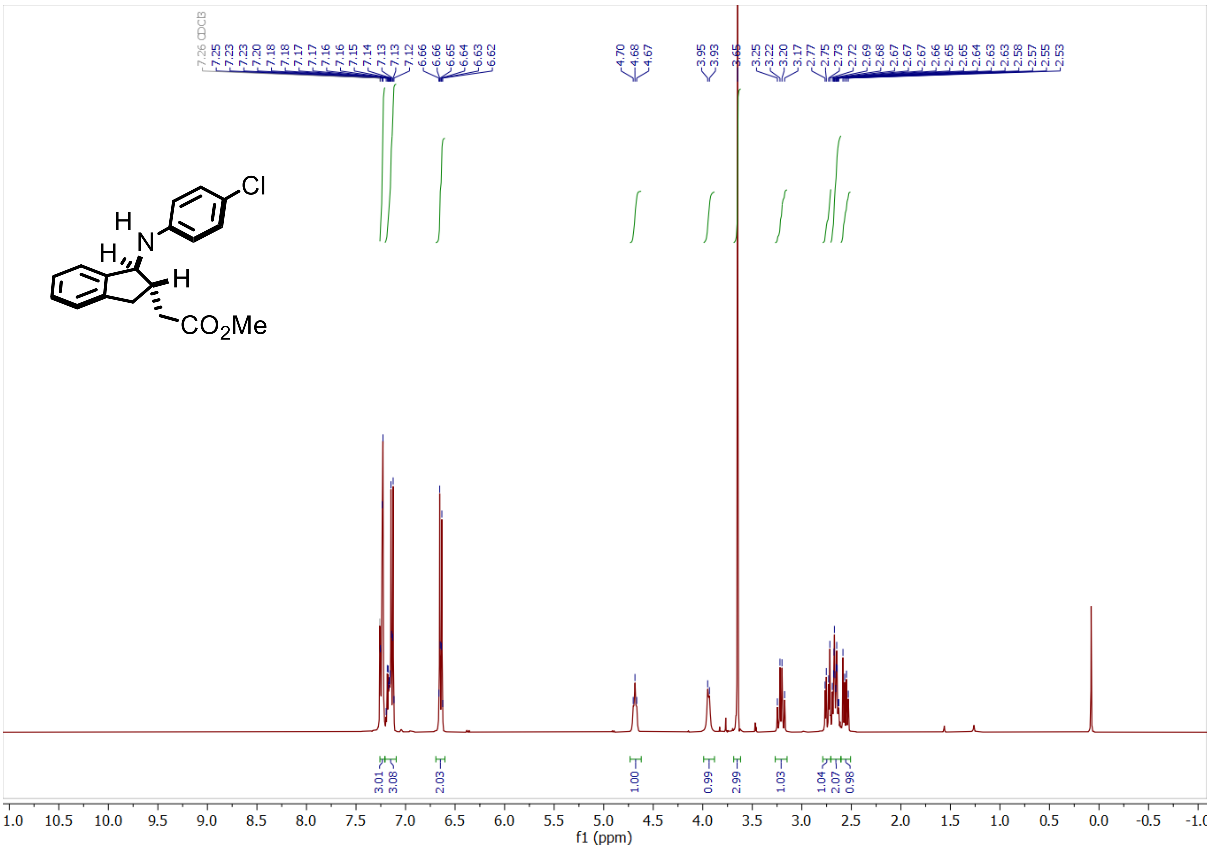
7.26 CDCl3
7.25 7.23 7.23 7.20 7.18 7.18 7.17 7.17 7.16 7.16 7.15 7.14 7.13 7.13 7.12 6.66 6.66 6.65 6.64 6.63 6.62
4.70 4.68 4.67
3.95 3.93
3.65
3.25 3.22 3.20 3.17 2.77 2.75 2.73 2.72 2.69 2.68 2.67 2.67 2.67 2.66 2.65 2.65 2.64 2.63 2.63 2.58 2.57 2.55 2.53
Cl
H
N
H
H
CO2Me
3.01 3.08 2.03 1.00 0.99 2.99 1.03 1.04 2.07 0.98
f1 (ppm)

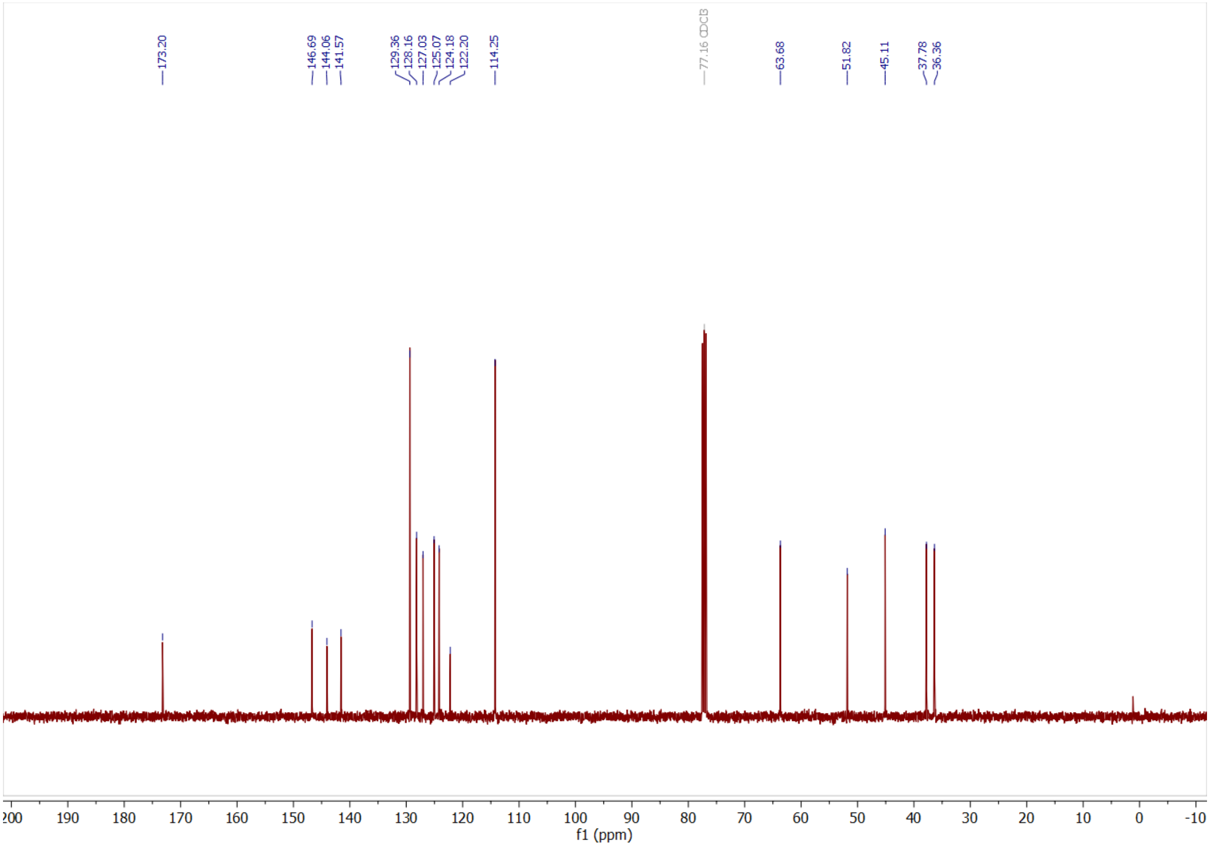
173.20
146.69 144.06 141.57
129.36 128.16 127.03 125.07 124.18 122.20
114.25
77.16 CDCl3
63.68
51.82
45.11
37.78 36.36
f1 (ppm)

2g

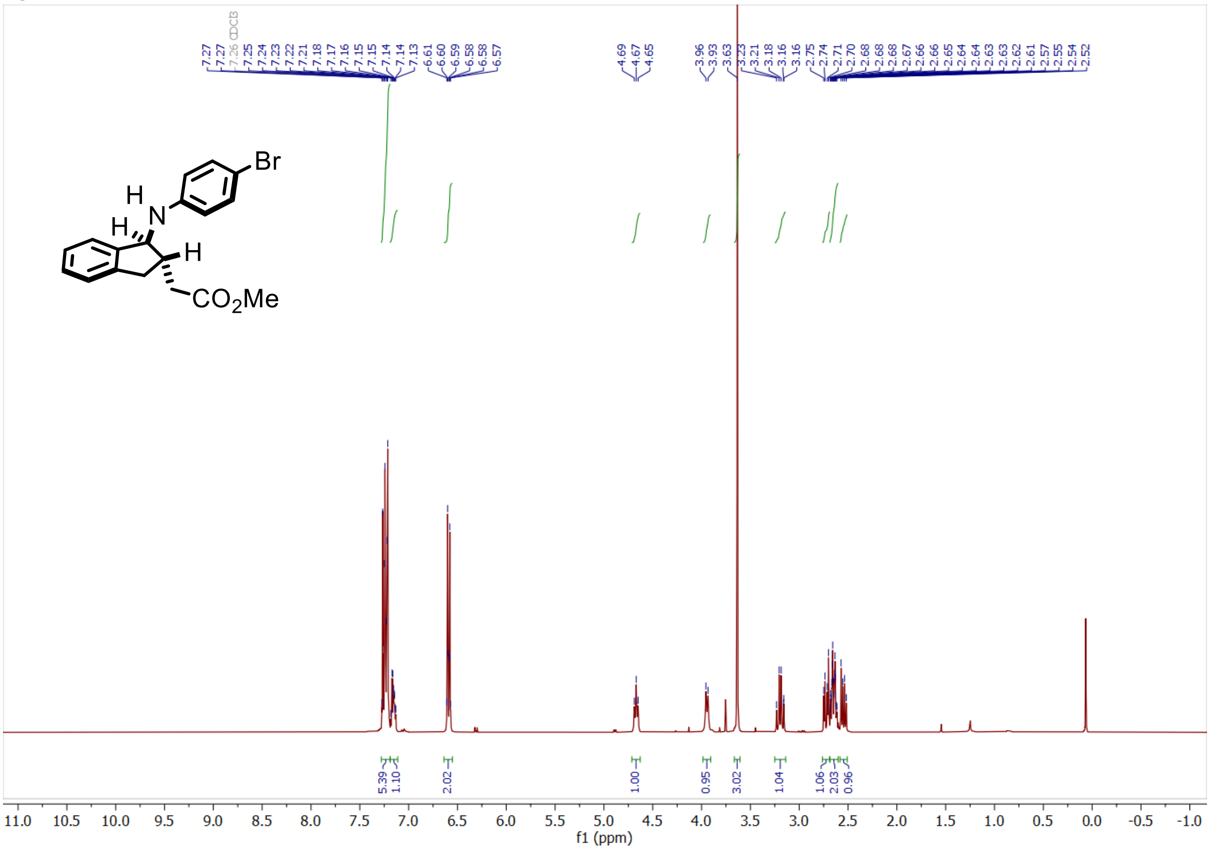
7.27
7.27
7.26 CDCl3
7.25
7.24
7.23
7.22
7.21
7.18
7.17
7.16
7.15
7.15
7.14
7.14
7.13
6.61
6.60
6.59
6.58
6.58
6.57
4.69
4.67
4.65
3.96
3.93
3.63
3.23
3.21
3.18
3.16
3.16
2.75
2.74
2.71
2.70
2.68
2.68
2.68
2.67
2.66
2.66
2.65
2.64
2.64
2.63
2.63
2.62
2.61
2.57
2.55
2.54
2.52
Br
H
N
H
H
CO2Me
5.39
1.10
2.02
1.00
0.95
3.02
1.04
1.06
2.03
0.96
f1 (ppm)

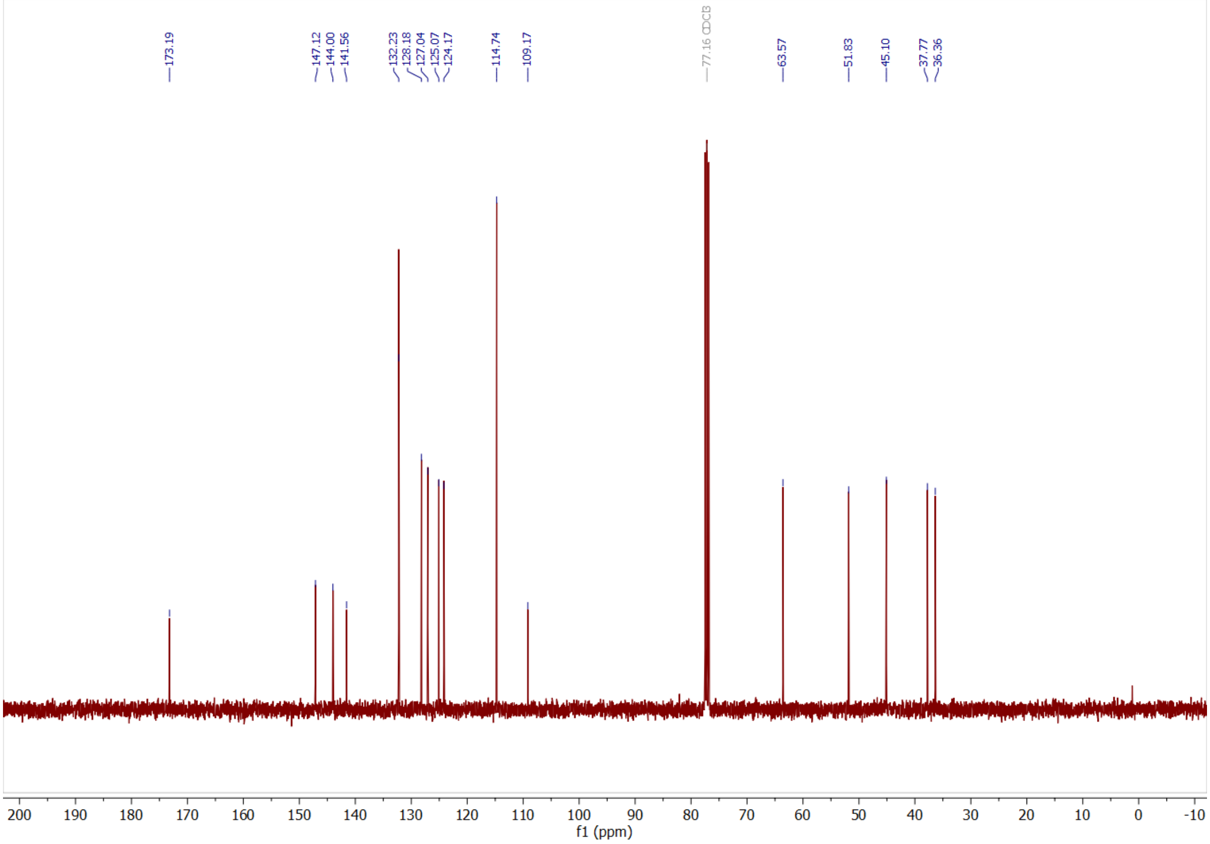
173.19
147.12
144.00
141.56
132.23
128.18
127.04
125.07
124.17
114.74
109.17
77.16 CDCl3
63.57
51.83
45.10
37.77
36.36
f1 (ppm)

2h

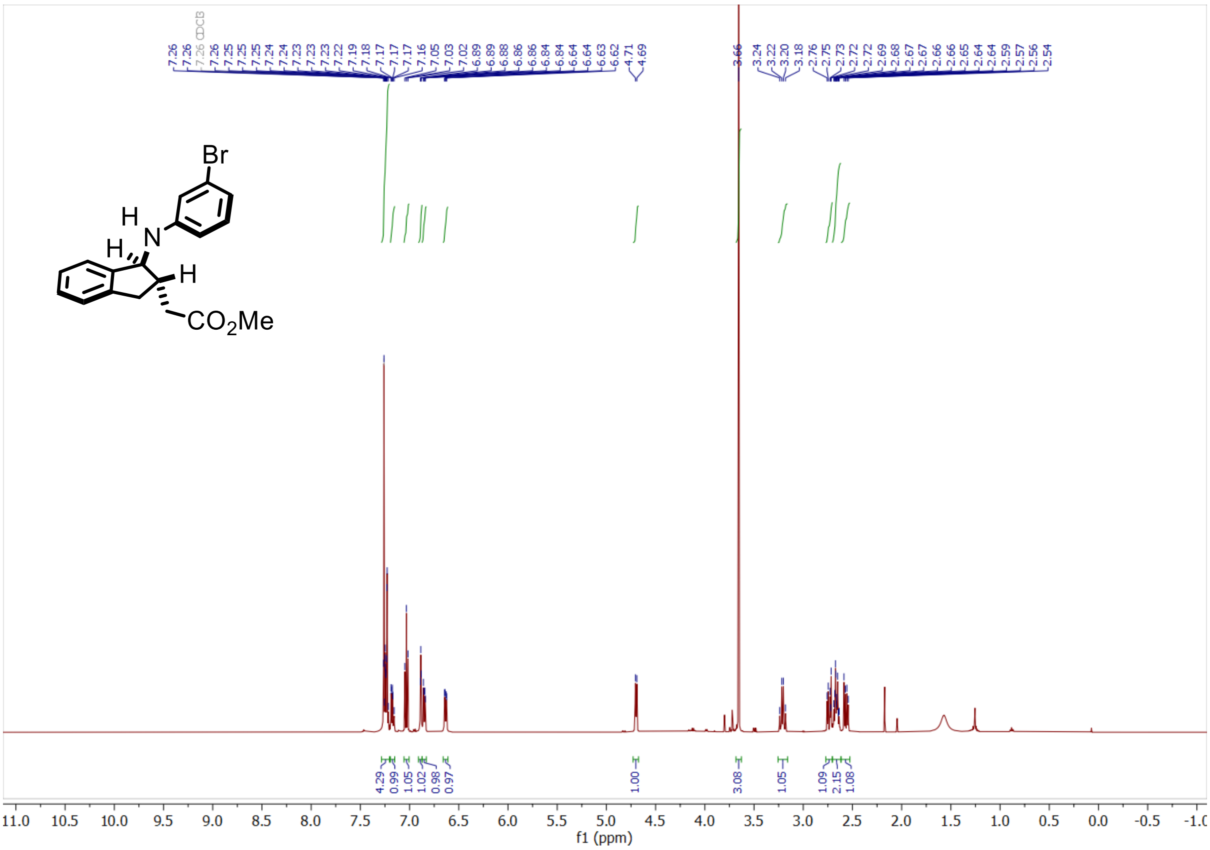
7.26 7.26 7.26 CDCl3 7.26 7.25 7.25 7.25 7.24 7.24 7.23 7.23 7.23 7.22 7.19 7.18 7.17 7.17 7.17 7.16 7.05 7.03 7.02 6.89 6.89 6.88 6.86 6.86 6.84 6.84 6.64 6.63 6.62 4.71 4.69
3.66 3.24 3.22 3.20 3.18 2.76 2.75 2.73 2.72 2.72 2.69 2.68 2.67 2.67 2.66 2.66 2.65 2.64 2.64 2.59 2.57 2.56 2.54
Br
H
N
H
H
CO2Me
4.29 0.99 1.05 1.02 0.98 0.97 1.00 3.08 1.05 1.09 2.15 1.08
f1 (ppm)

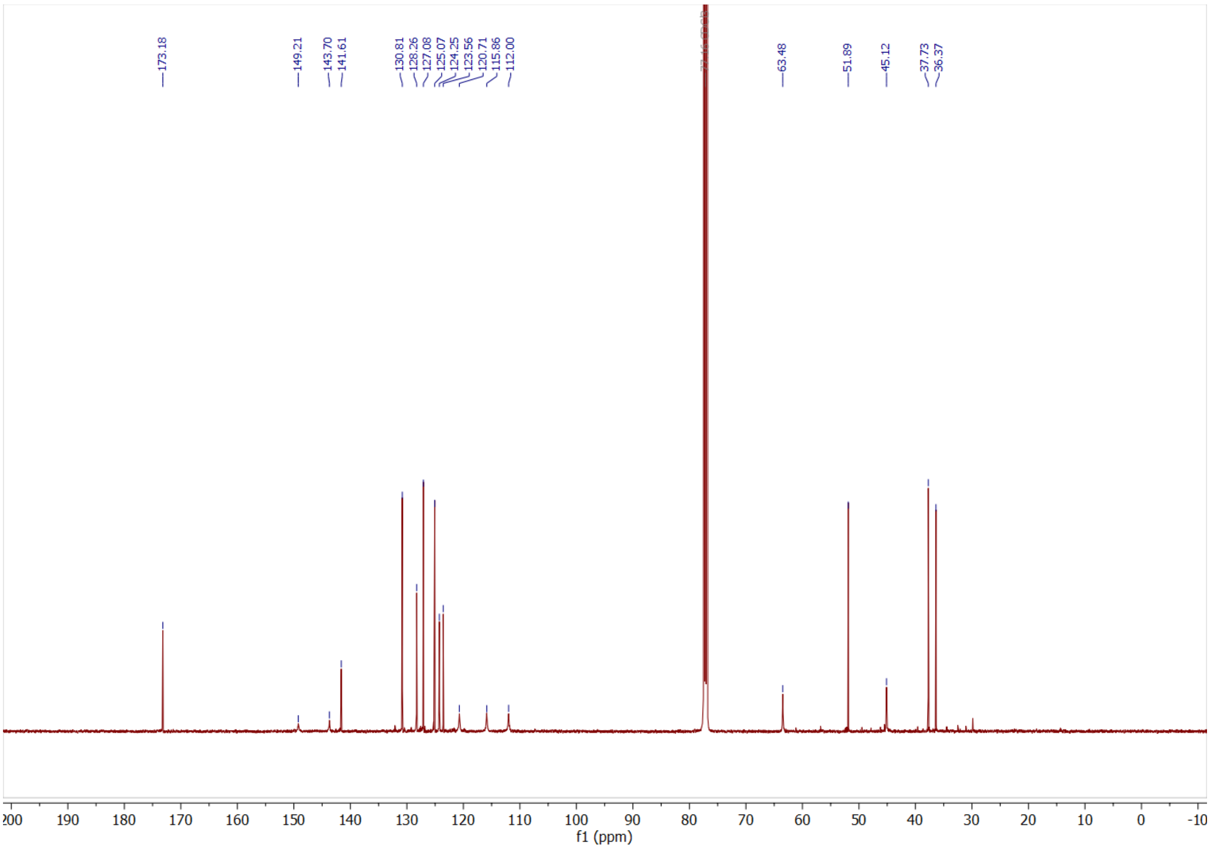
173.18 149.21 143.70 141.61 130.81 128.26 127.08 125.07 124.25 123.56 120.71 115.86 112.00 63.48 51.89 45.12 37.73 36.37
f1 (ppm)

2i

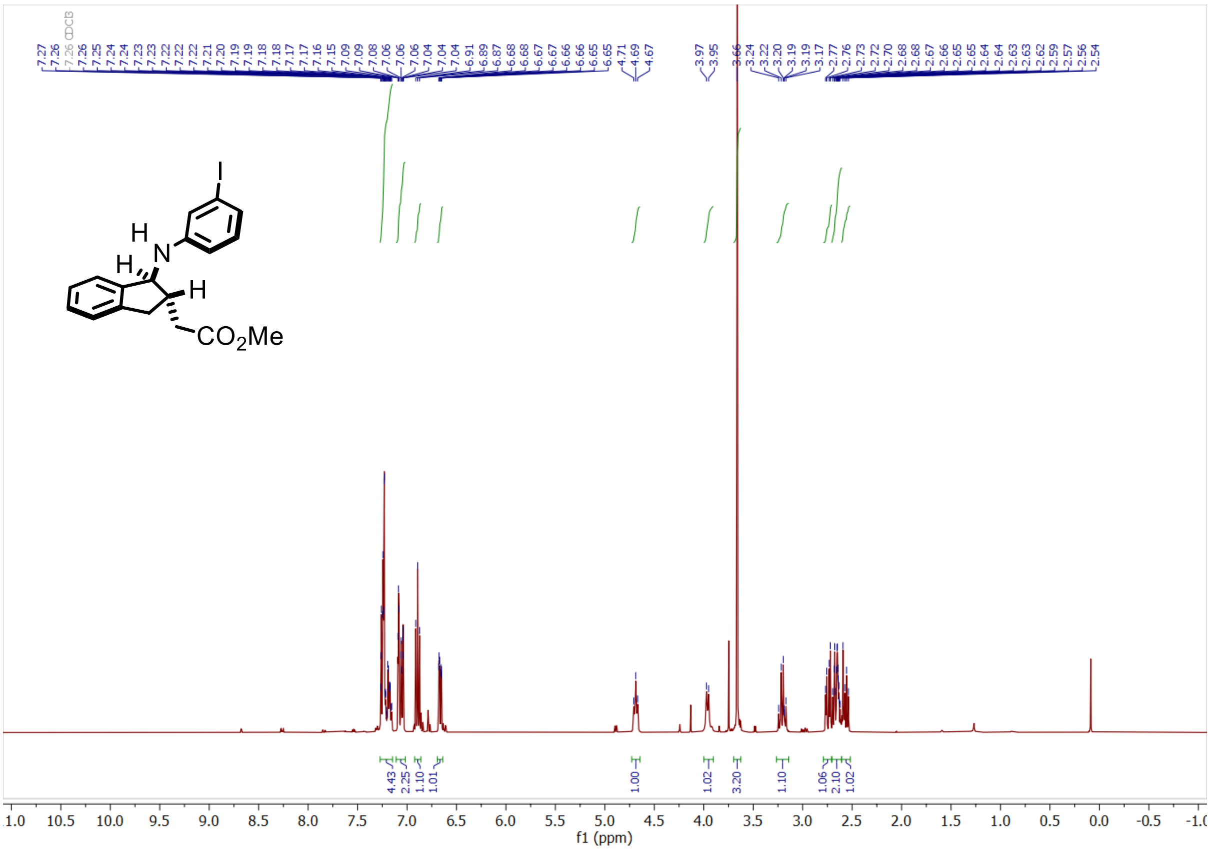
H
N
H
H
CO2Me
7.27 7.26 7.26 CDCl3 7.26 7.25 7.24 7.24 7.23 7.23 7.22 7.22 7.22 7.21 7.20 7.19 7.19 7.18 7.18 7.17 7.17 7.16 7.15 7.09 7.09 7.08 7.06 7.06 7.06 7.04 7.04 7.04 6.91 6.89 6.87 6.68 6.68 6.67 6.67 6.66 6.66 6.65 6.65 4.71 4.69 4.67 3.97 3.95 3.66 3.24 3.22 3.20 3.19 3.19 3.17 2.77 2.76 2.73 2.72 2.70 2.68 2.68 2.67 2.66 2.65 2.65 2.64 2.64 2.63 2.63 2.62 2.59 2.57 2.56 2.54
4.43 2.25 1.10 1.01 1.00 1.02 3.20 1.10 1.06 2.10 1.02
f1 (ppm)

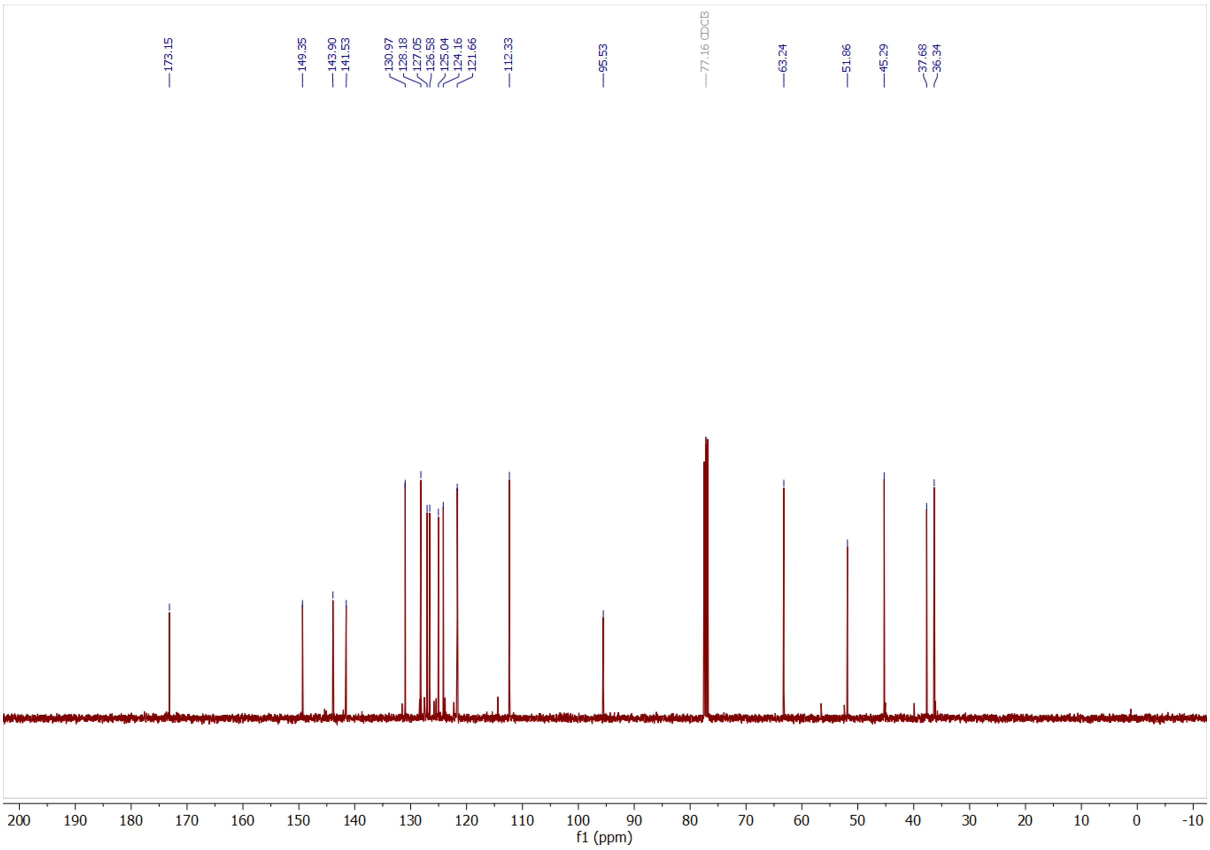
173.15
149.35 143.90 141.53
130.97 128.18 127.05 126.58 125.04 124.16 121.66
112.33
95.53
77.16 CDCl3
63.24
51.86
45.29
37.68 36.34
f1 (ppm)

2j

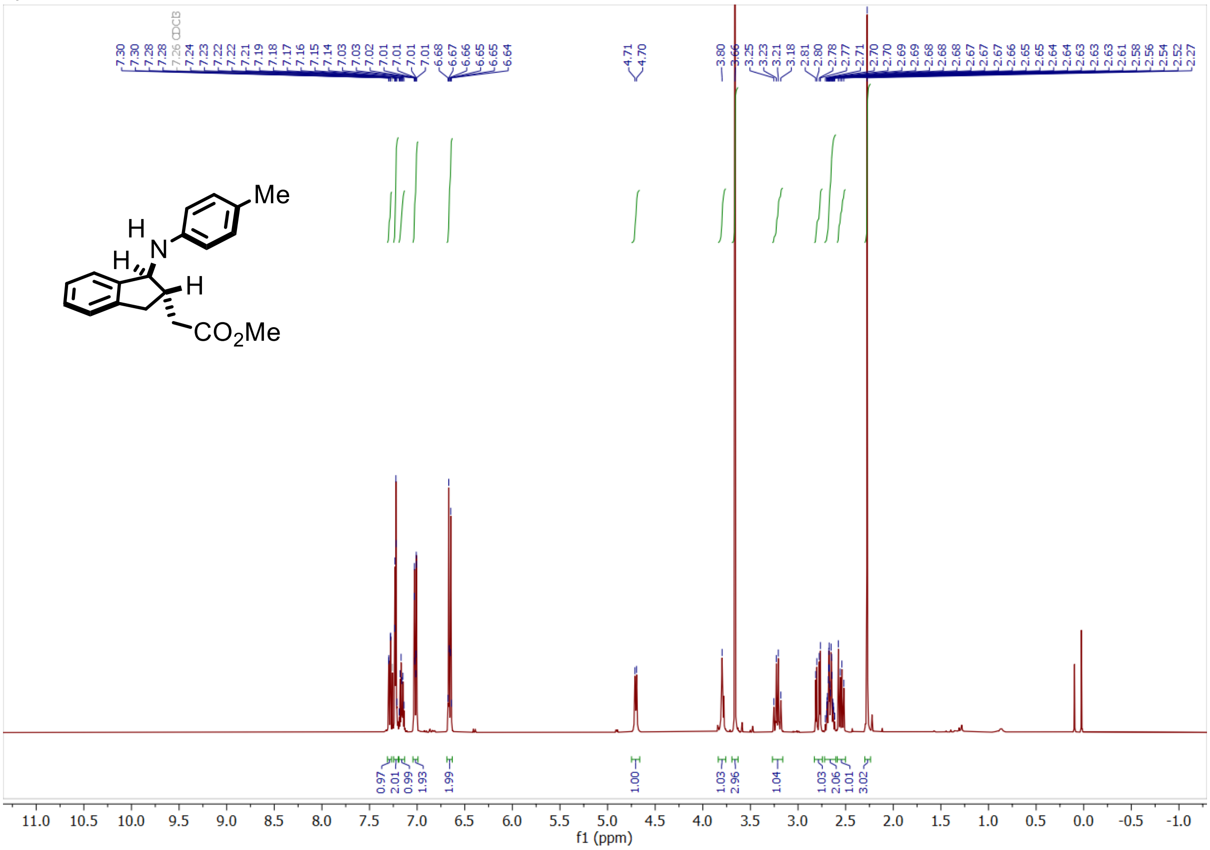
7.30 7.30 7.28 7.28 7.26 CDCl3 7.24 7.23 7.22 7.22 7.21 7.19 7.18 7.17 7.16 7.15 7.14 7.03 7.03 7.02 7.01 7.01 7.01 7.01 6.68 6.67 6.66 6.65 6.65 6.64
4.71 4.70
3.80 3.66 3.25 3.23 3.21 3.18 2.81 2.80 2.78 2.77 2.71 2.70 2.70 2.69 2.69 2.68 2.68 2.67 2.67 2.66 2.65 2.65 2.64 2.63 2.63 2.63 2.61 2.58 2.56 2.54 2.52 2.27
Me
H
N
H
H
CO2Me
0.97 2.01 0.99 1.93 1.99 1.00 1.03 2.96 1.04 1.03 2.06 1.01 3.02
f1 (ppm)

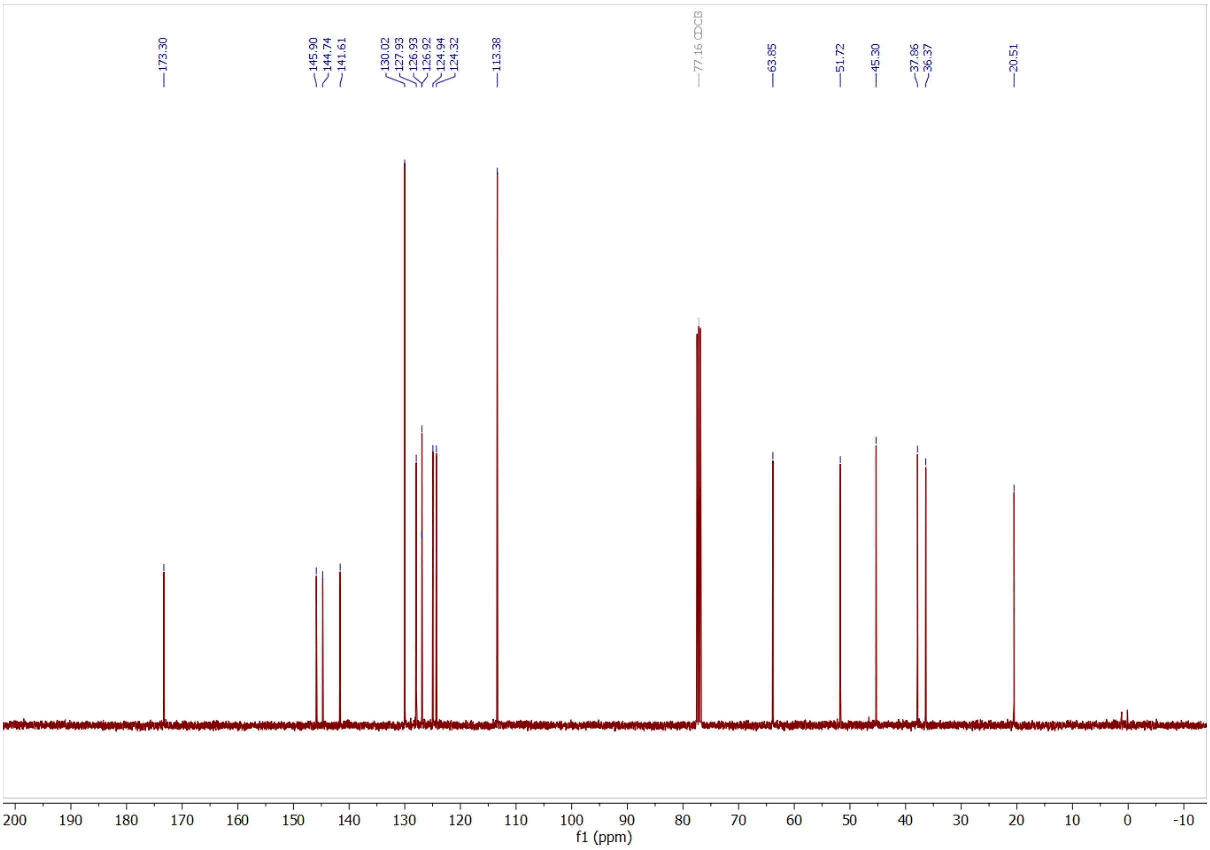
173.30
145.90 144.74 141.61
130.02 127.93 126.93 126.92 124.94 124.32
113.38
77.16 CDCl3
63.85
51.72
45.30
37.86 36.37
20.51
f1 (ppm)

2k

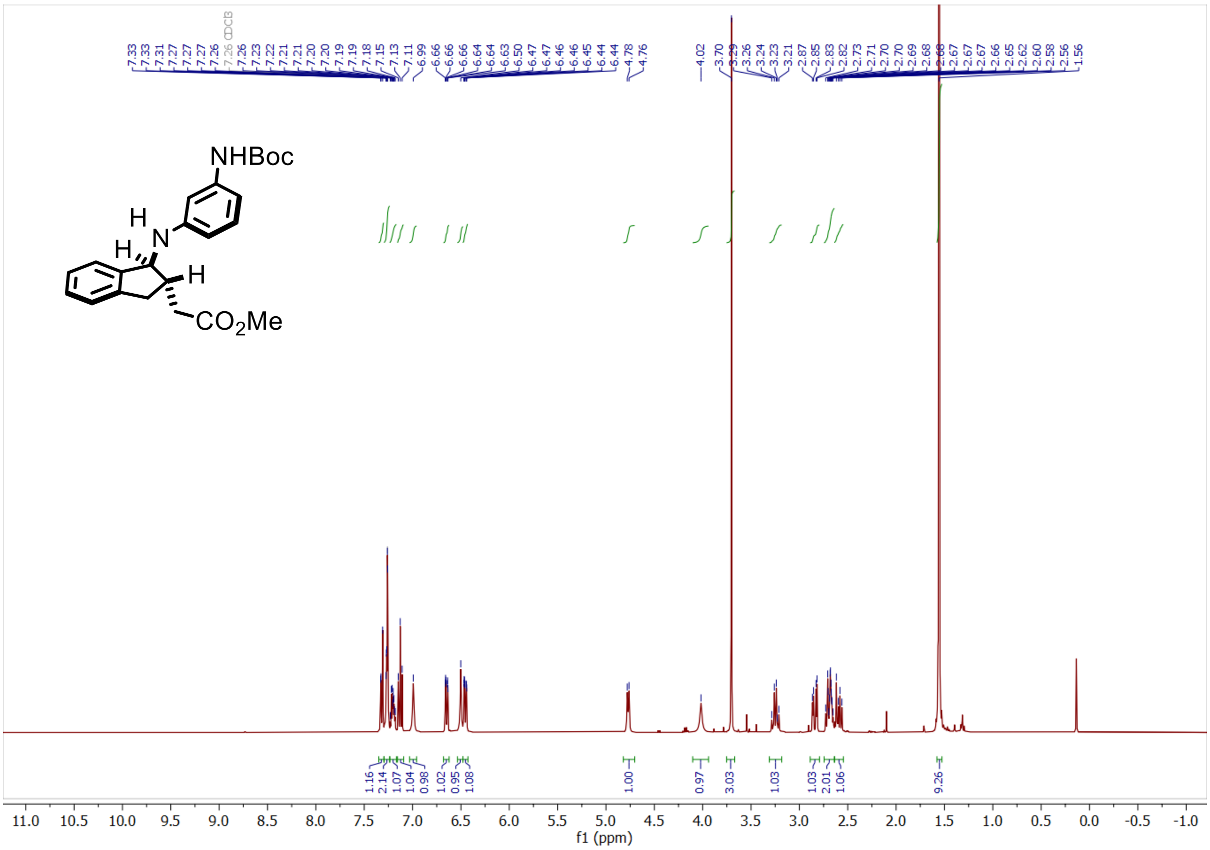
7.33
7.33
7.31
7.27
7.27
7.26
7.26 CDCl3
7.26
7.23
7.22
7.21
7.21
7.20
7.20
7.19
7.19
7.18
7.15
7.13
7.11
6.99
6.66
6.66
6.64
6.64
6.63
6.50
6.47
6.47
6.46
6.46
6.45
6.44
6.44
4.78
4.76
4.02
3.70
3.29
3.26
3.24
3.23
3.21
2.87
2.85
2.83
2.82
2.73
2.71
2.70
2.70
2.69
2.68
2.68
2.67
2.67
2.66
2.65
2.62
2.60
2.58
2.56
1.56
NHBoc
H
N
H
H
CO2Me
1.16
2.14
1.07
1.04
0.98
1.02
0.95
1.08
1.00
0.97
3.03
1.03
1.03
2.01
1.06
9.26
f1 (ppm)

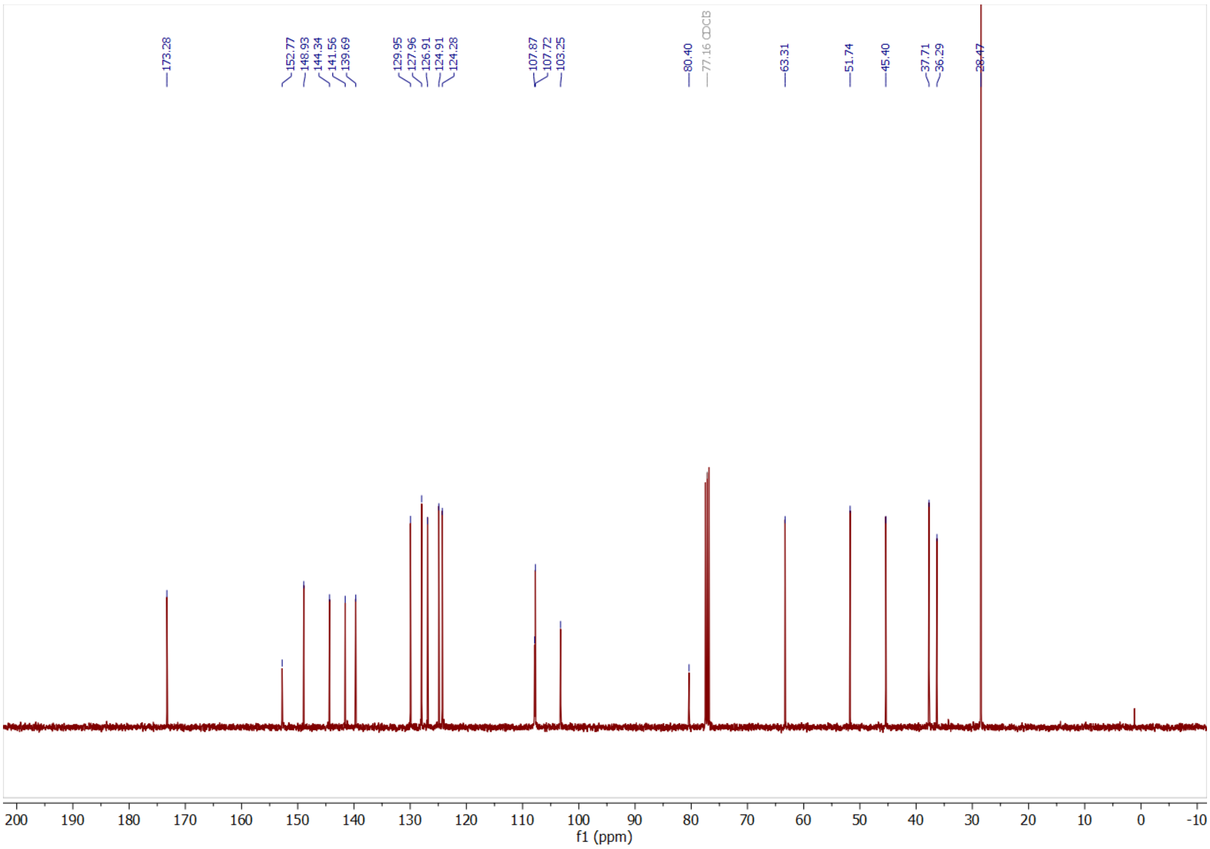
173.28
152.77
148.93
144.34
141.56
139.69
129.95
127.96
126.91
124.91
124.28
107.87
107.72
103.25
80.40
77.16 CDCl3
63.31
51.74
45.40
37.71
36.29
28.47
f1 (ppm)

2l

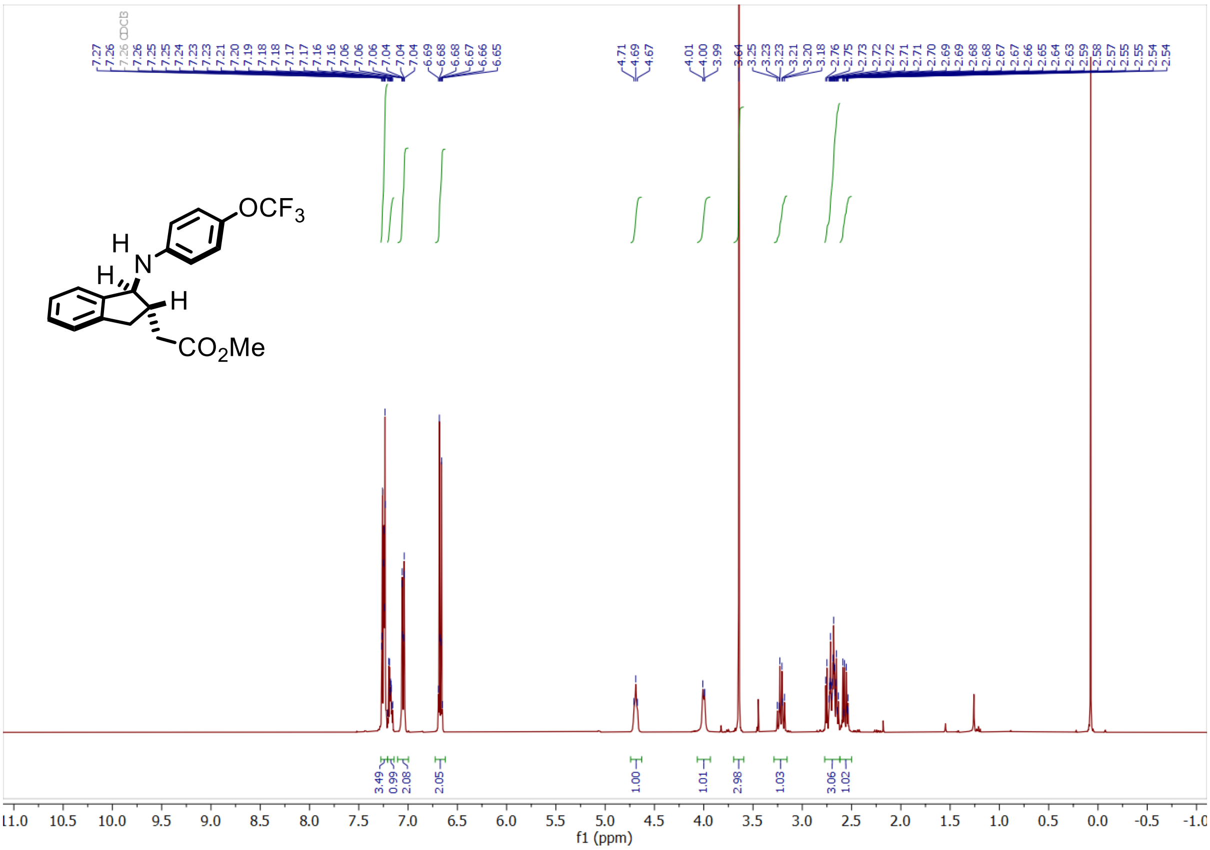
7.27
7.26
7.26 CDCl3
7.26
7.25
7.25
7.24
7.23
7.23
7.21
7.20
7.19
7.18
7.18
7.17
7.17
7.16
7.06
7.06
7.04
7.04
7.04
6.69
6.68
6.68
6.67
6.66
6.65
4.71
4.69
4.67
4.01
4.00
3.99
3.64
3.25
3.23
3.21
3.20
3.18
2.76
2.75
2.73
2.72
2.72
2.71
2.70
2.69
2.69
2.68
2.68
2.67
2.67
2.66
2.65
2.64
2.63
2.59
2.58
2.57
2.55
2.55
2.54
2.54
OCF3
H
N
H
H
CO2Me
3.49
0.99
2.08
2.05
1.00
1.01
2.98
1.03
3.06
1.02
11.0 10.5 10.0 9.5 9.0 8.5 8.0 7.5 7.0 6.5 6.0 5.5 5.0 4.5 4.0 3.5 3.0 2.5 2.0 1.5 1.0 0.5 0.0 -0.5 -1.0
f1 (ppm)

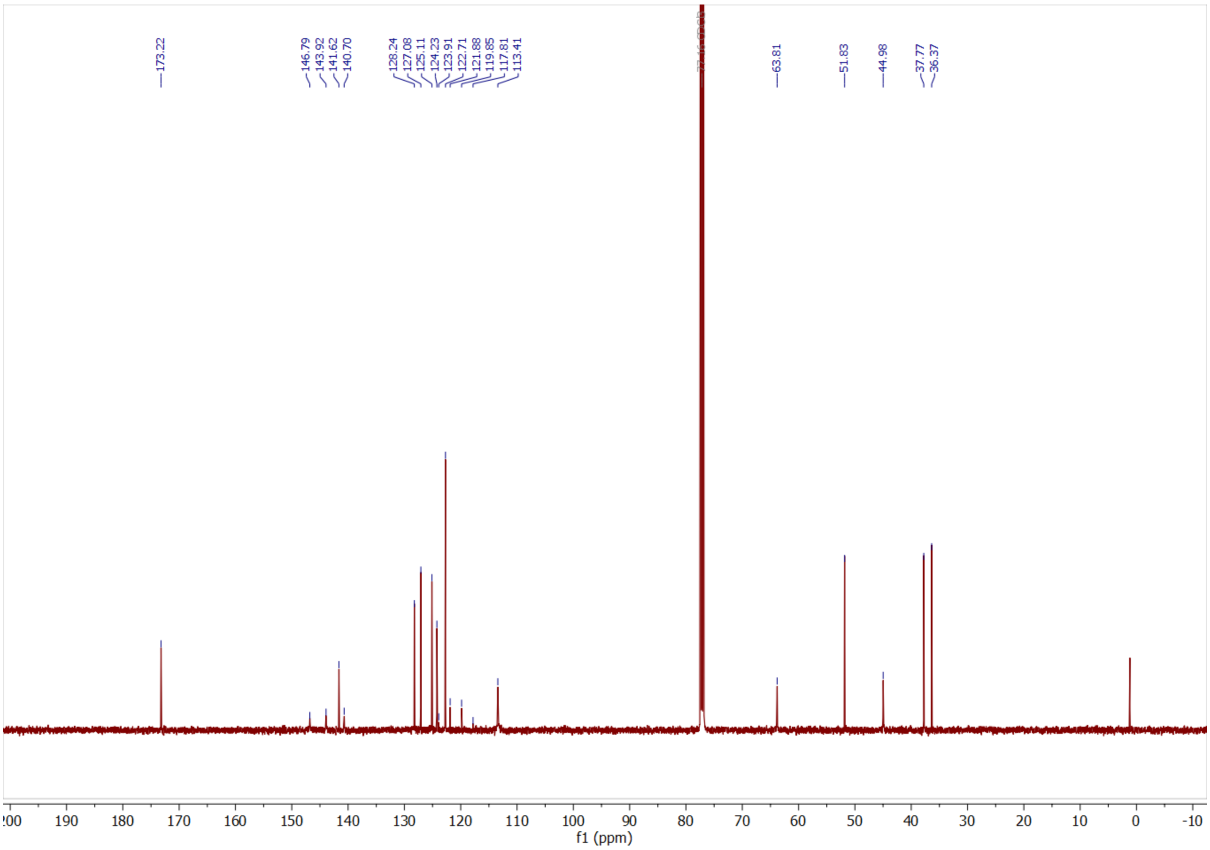
173.22
146.79
143.92
141.62
140.70
128.24
127.08
125.11
124.23
123.91
122.71
121.88
119.85
117.81
113.41
63.81
51.83
44.98
37.77
36.37
200 190 180 170 160 150 140 130 120 110 100 90 80 70 60 50 40 30 20 10 0 -10
f1 (ppm)

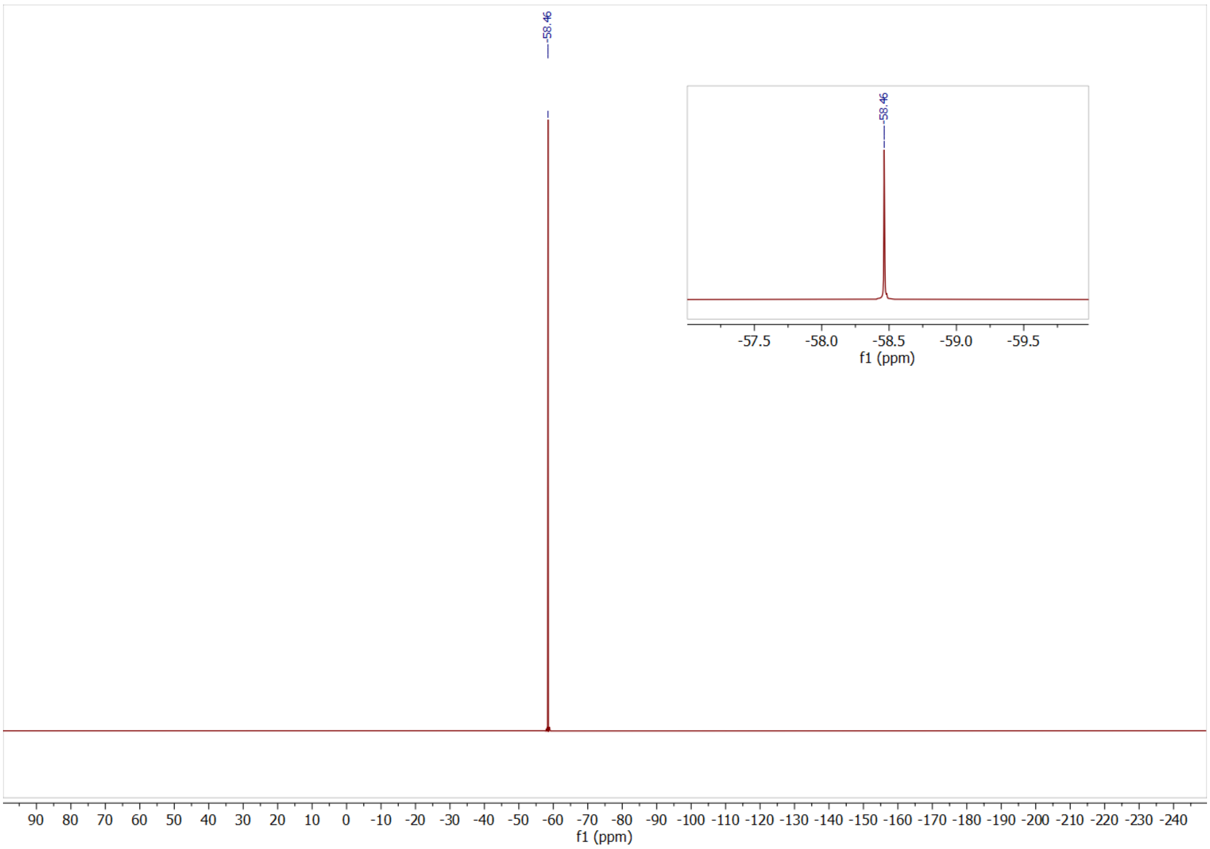

-58.46
-57.5
-58.0
-58.5
-59.0
-59.5
f1 (ppm)
90 80 70 60 50 40 30 20 10 0 -10 -20 -30 -40 -50 -60 -70 -80 -90 -100 -110 -120 -130 -140 -150 -160 -170 -180 -190 -200 -210 -220 -230 -240
f1 (ppm)

2m

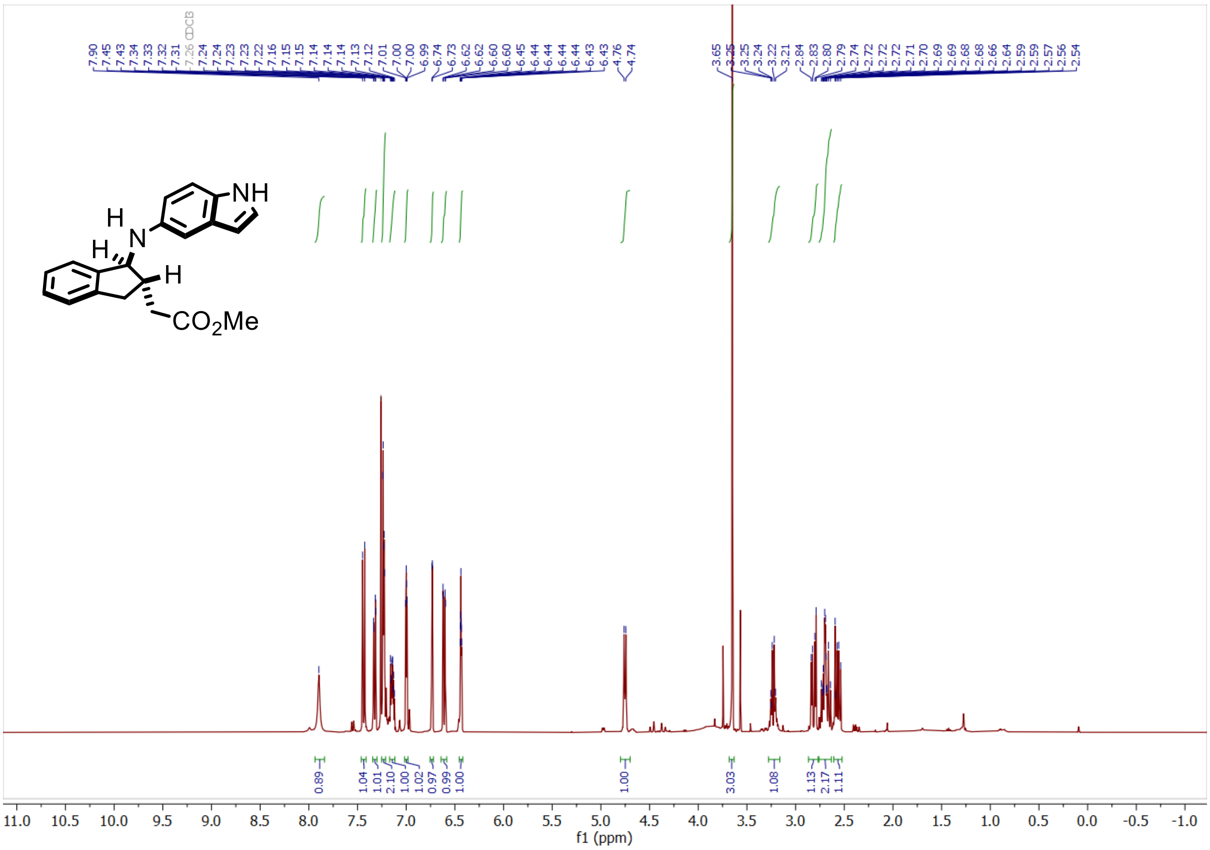

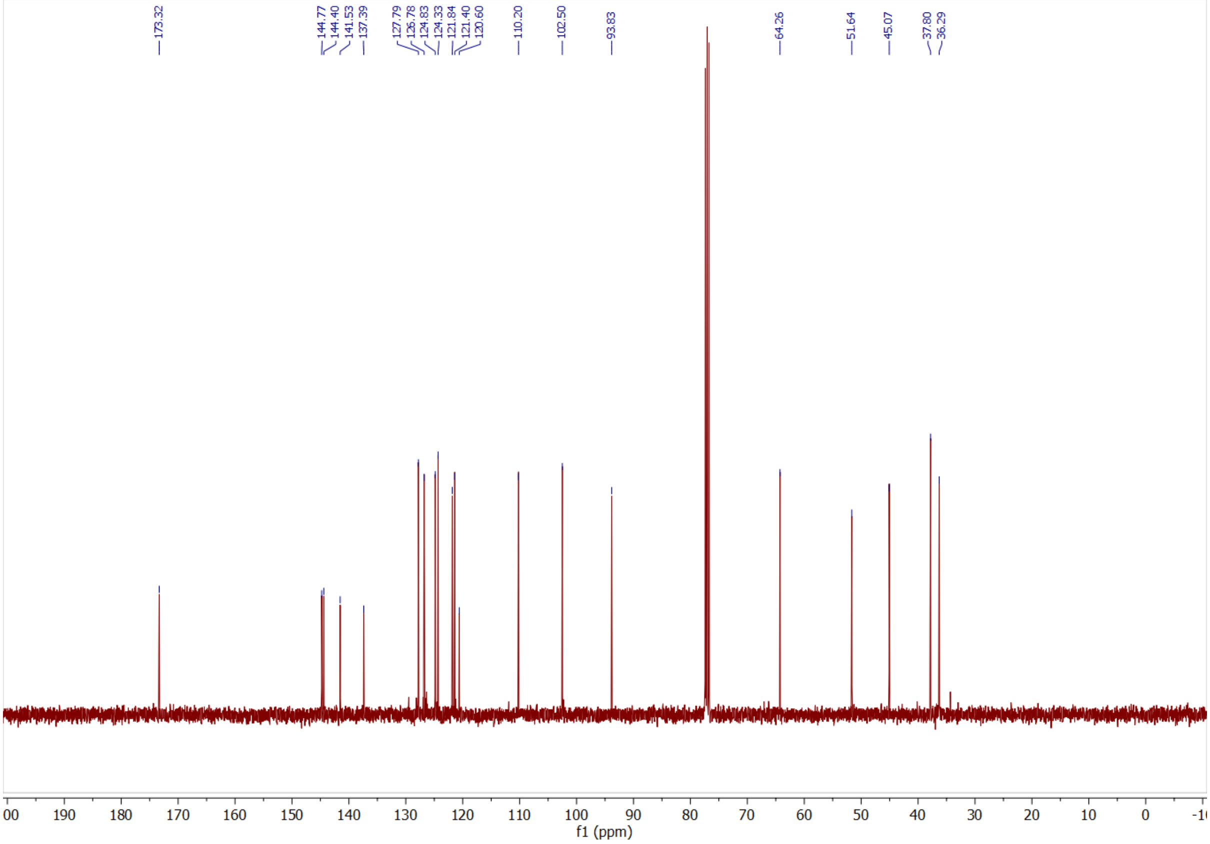

2n

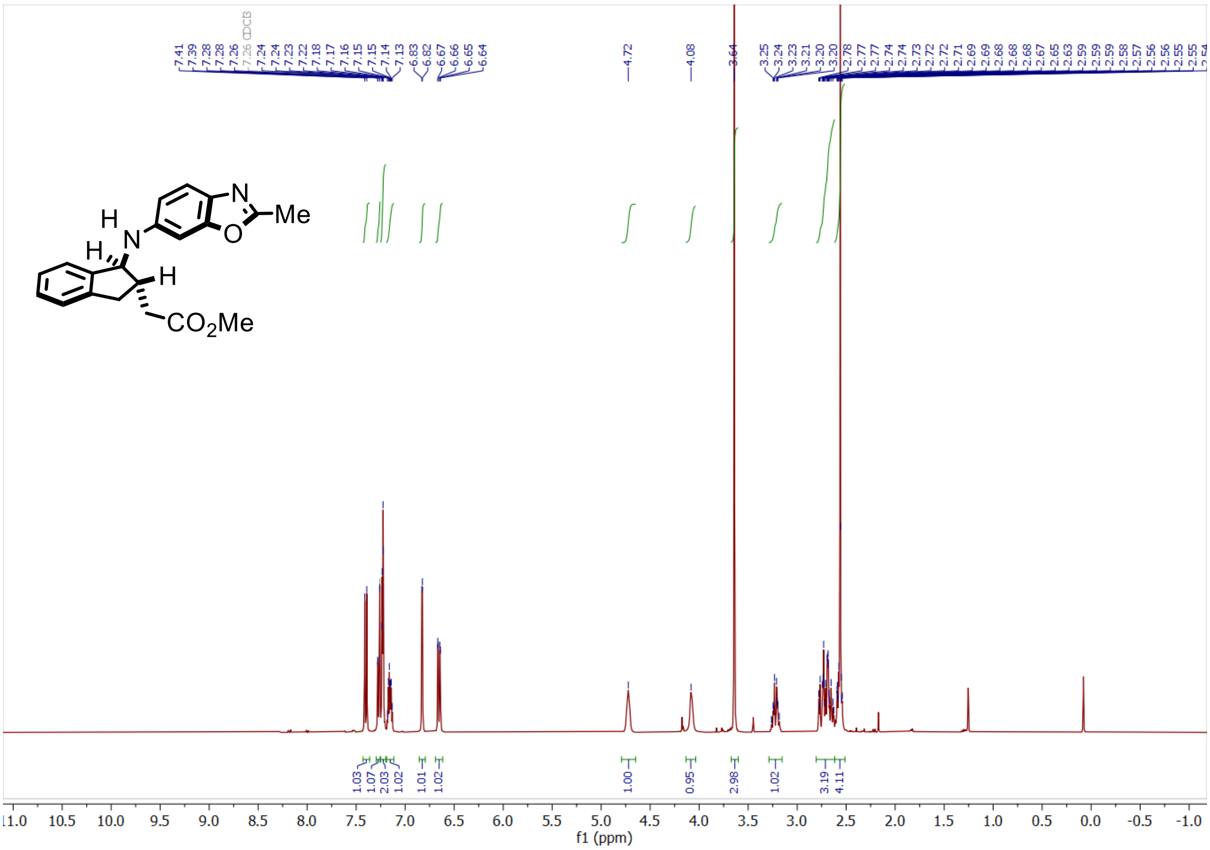

H
N
H
H
N
O
Me
CO2Me
7.41
7.39
7.28
7.26
7.26 CDCl3
7.24
7.24
7.23
7.22
7.18
7.17
7.16
7.15
7.14
7.13
6.83
6.82
6.67
6.66
6.65
6.64
4.72
4.08
3.64
3.25
3.24
3.23
3.21
3.20
3.20
2.78
2.77
2.74
2.74
2.73
2.72
2.72
2.71
2.69
2.69
2.68
2.68
2.67
2.65
2.63
2.59
2.59
2.59
2.58
2.57
2.56
2.56
2.55
1.03
1.07
2.03
1.02
1.01
1.02
1.00
0.95
2.98
1.02
3.19
4.11
f1 (ppm)

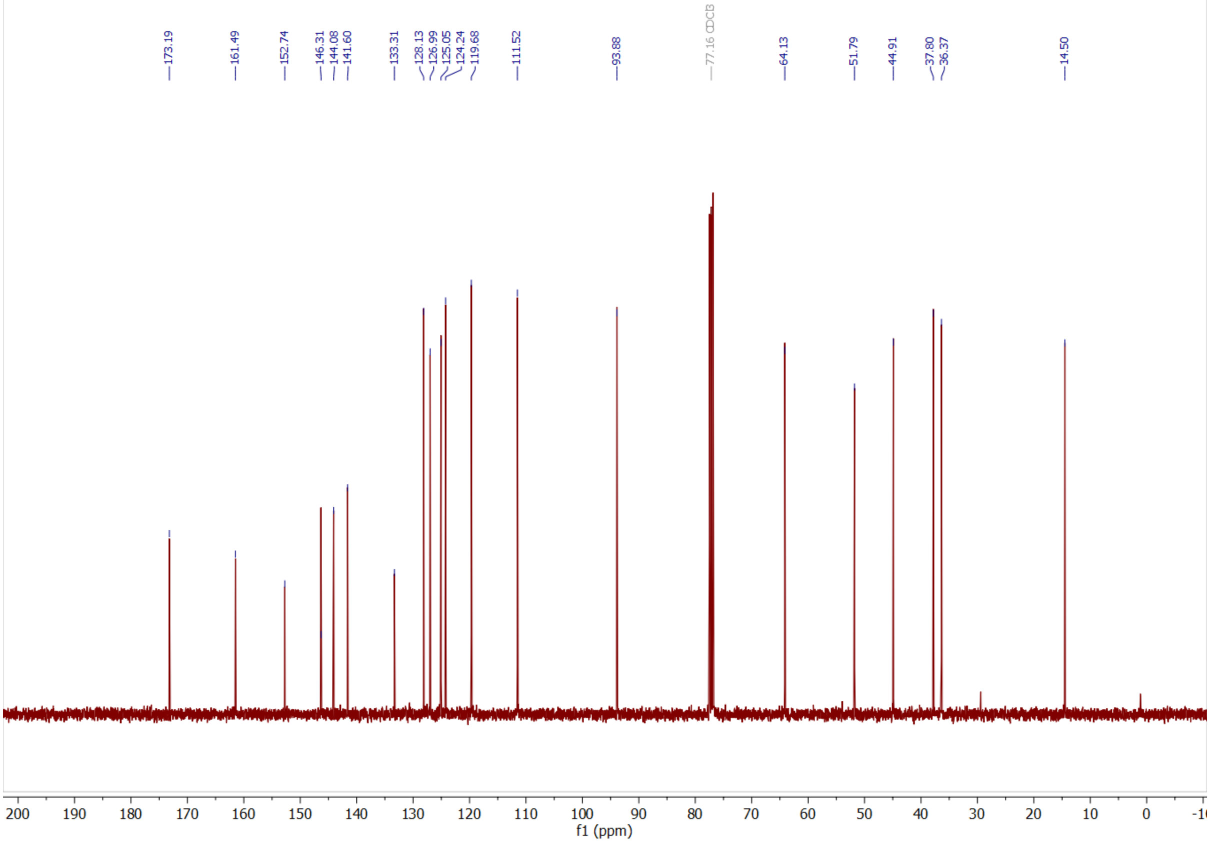

173.19
161.49
152.74
146.31
144.08
141.60
133.31
128.13
126.99
125.05
124.24
119.68
111.52
93.88
77.16 CDCl3
64.13
51.79
44.91
37.80
36.37
14.50
f1 (ppm)

2o

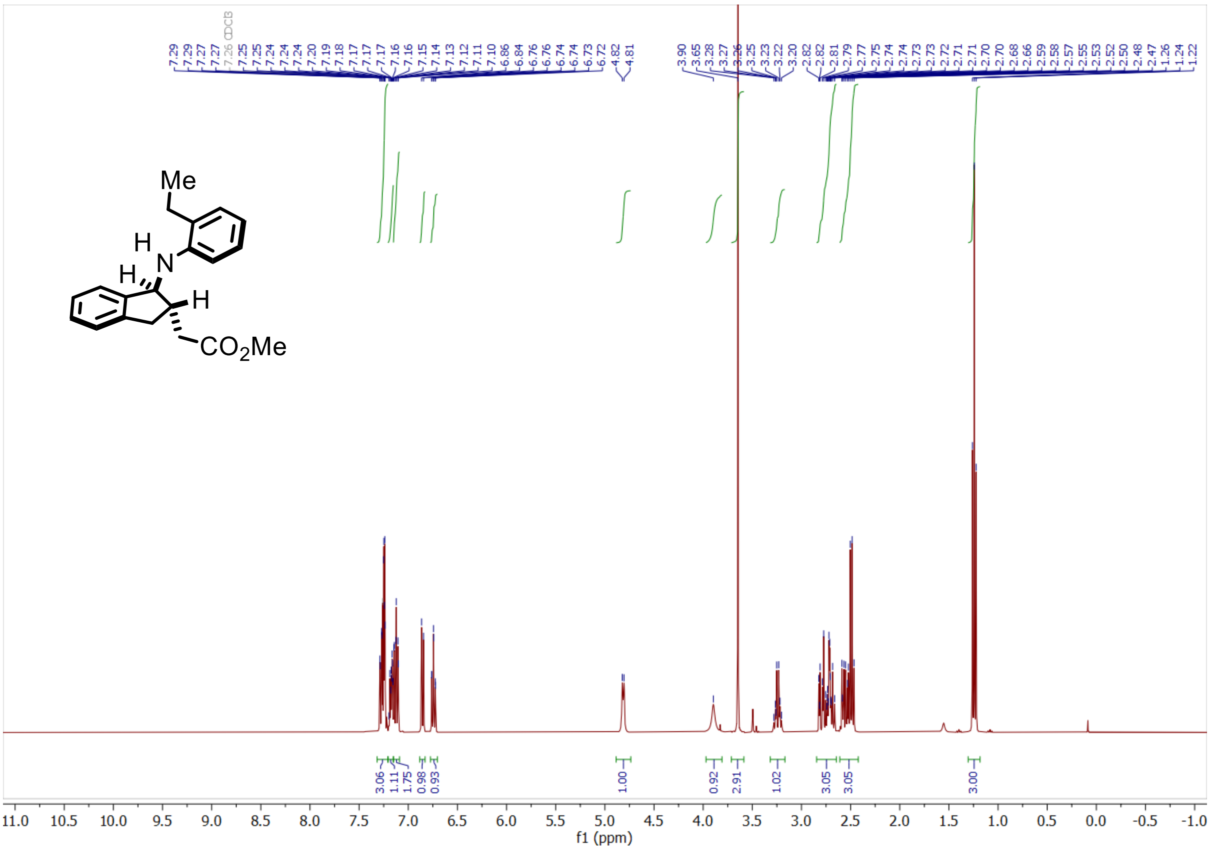
Me
H
N
H
H
CO2Me
f1 (ppm)

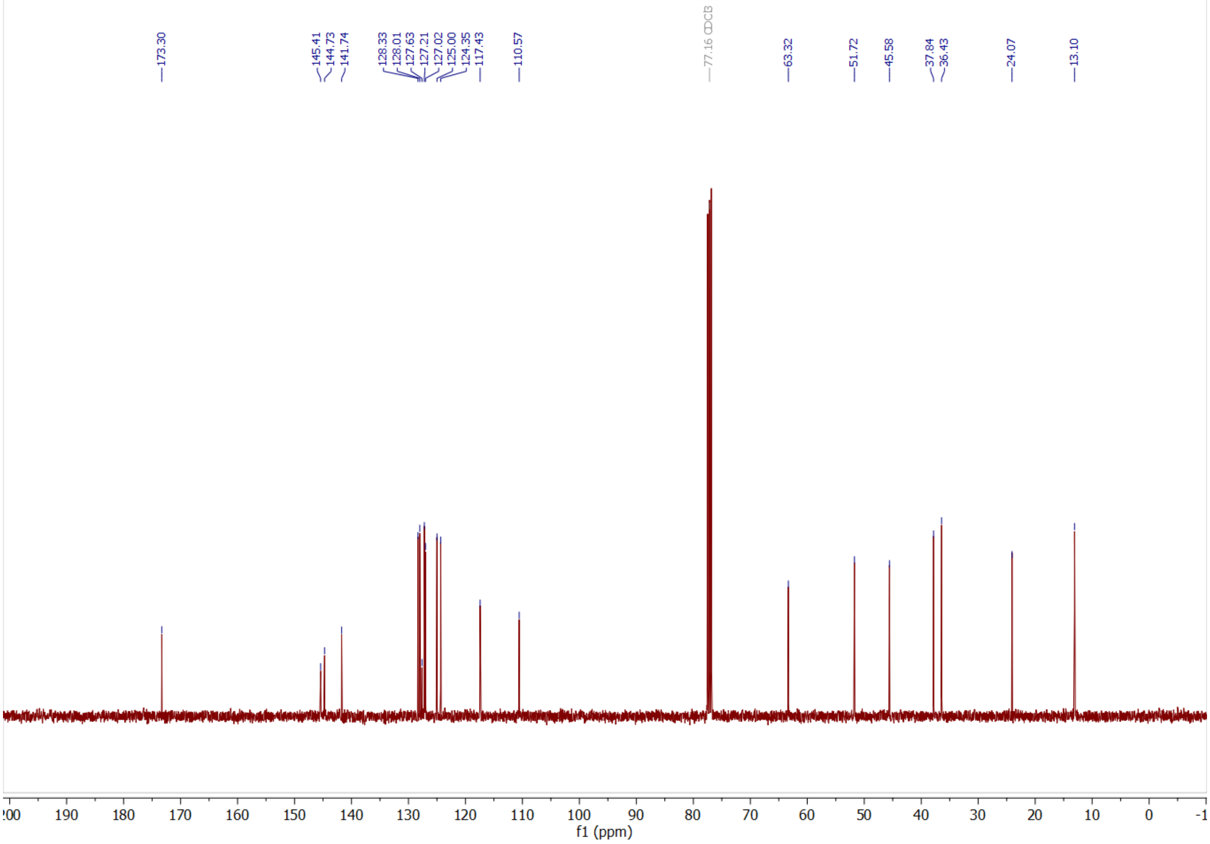
173.30
145.41
144.73
141.74
128.33
128.01
127.63
127.21
127.02
125.00
124.35
117.43
110.57
77.16 CDCl3
63.32
51.72
45.58
37.84
36.43
24.07
13.10
f1 (ppm)

2p

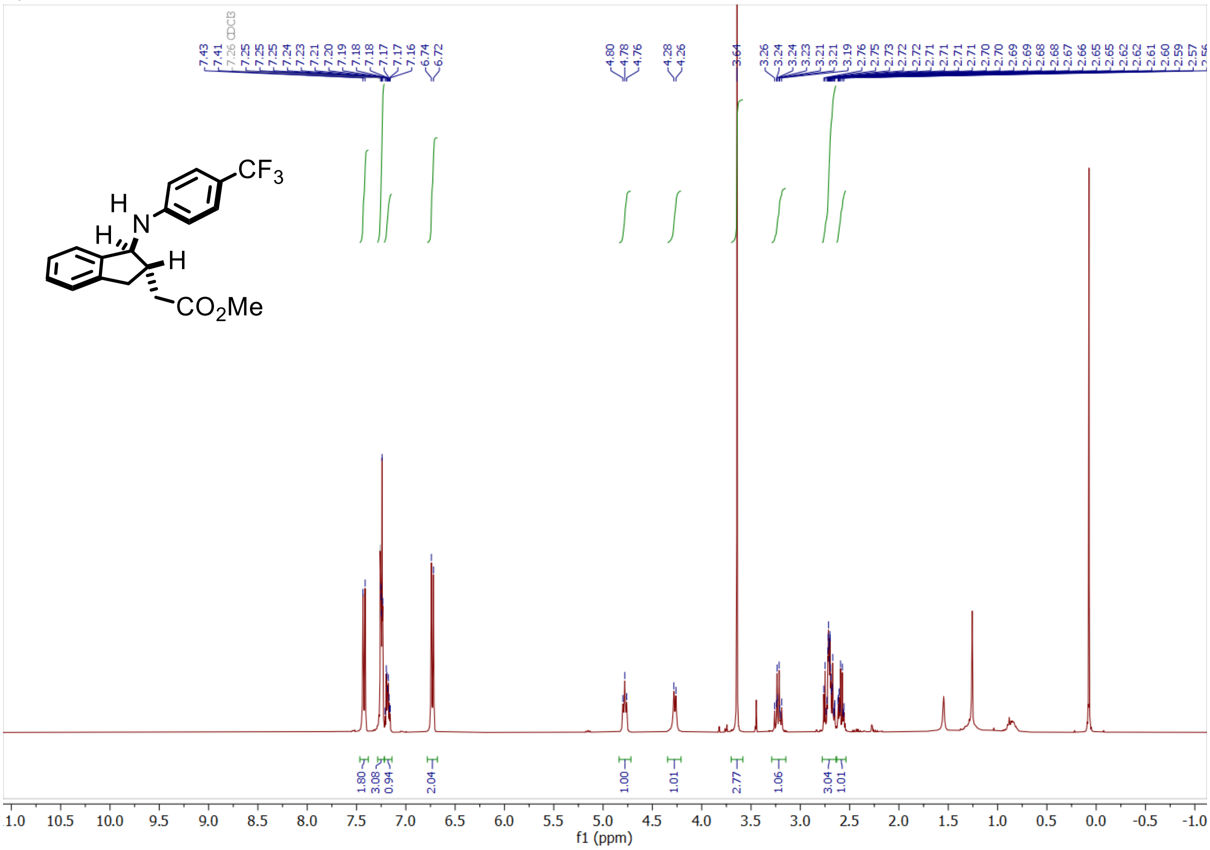

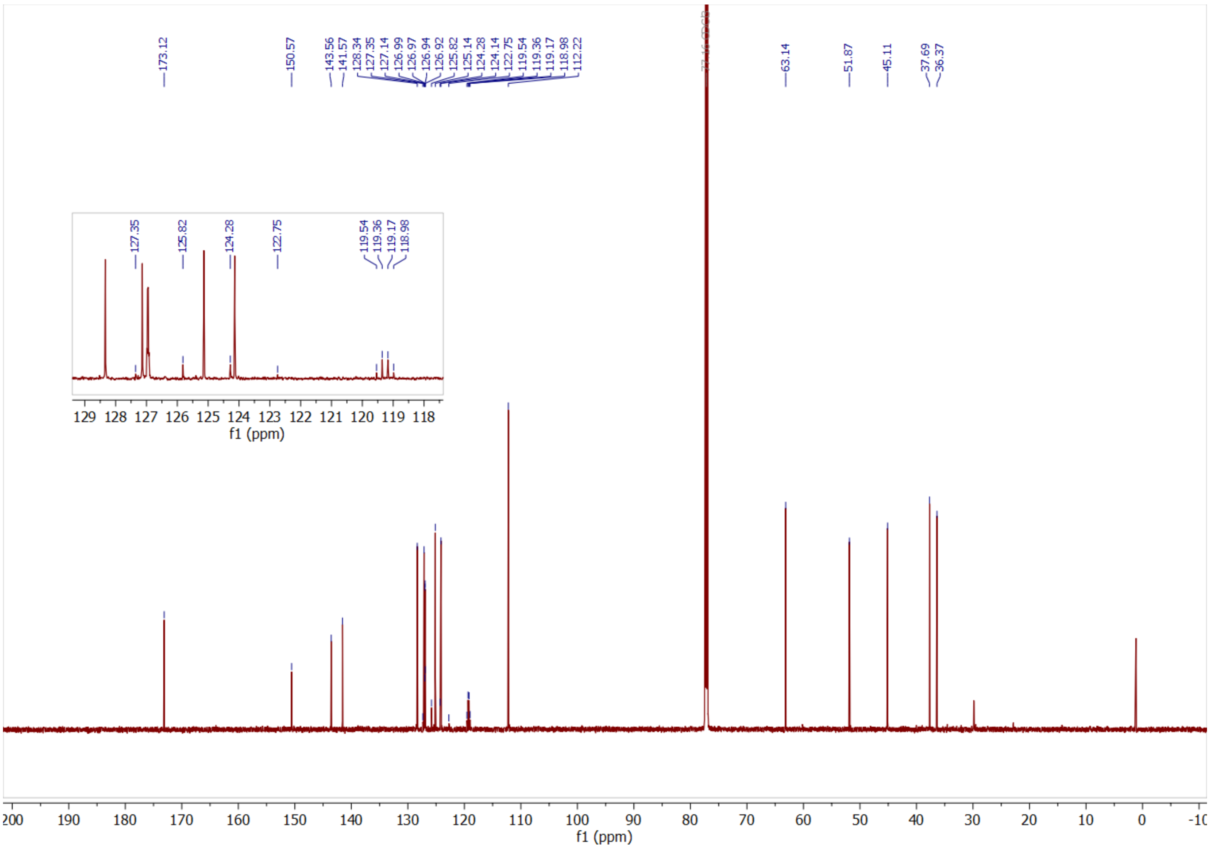

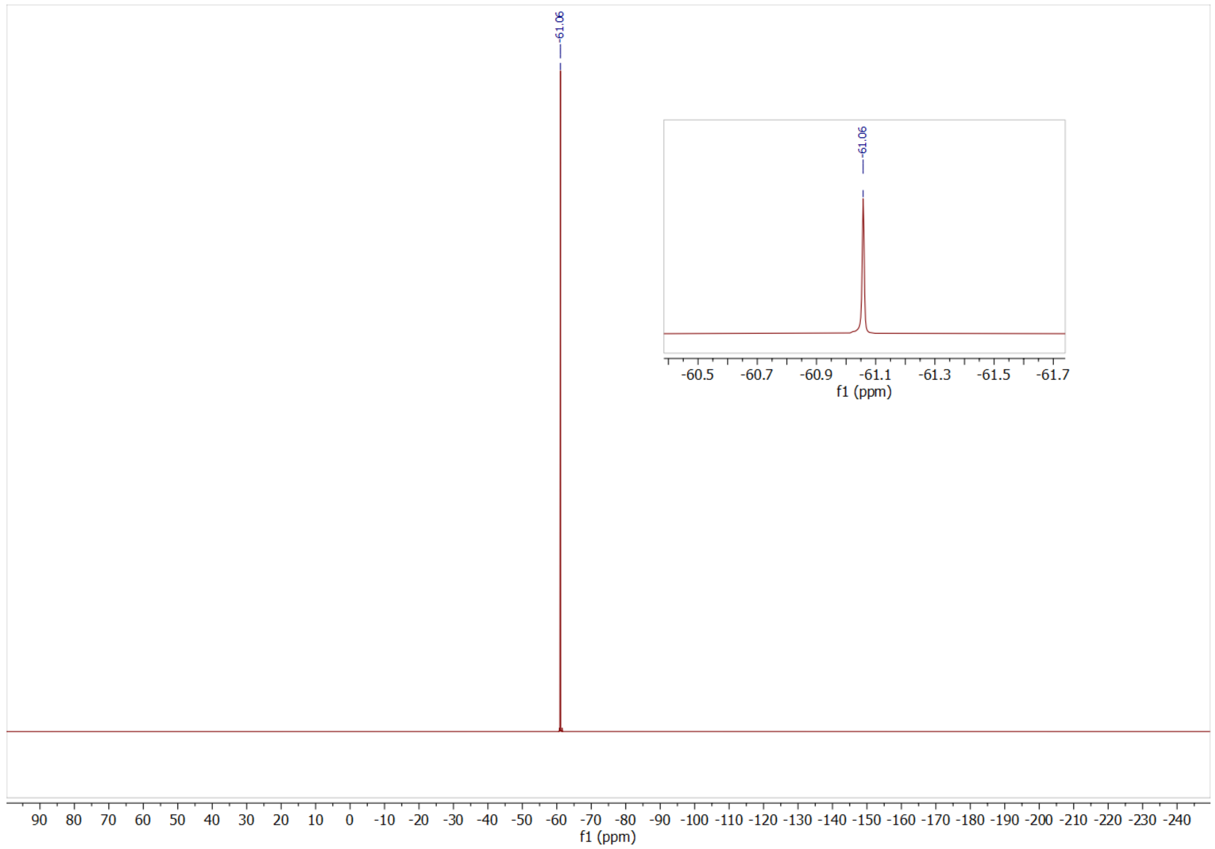

-61.06
-61.06
-60.5
-60.7
-60.9
-61.1
-61.3
-61.5
-61.7
f1 (ppm)
90 80 70 60 50 40 30 20 10 0 -10 -20 -30 -40 -50 -60 -70 -80 -90 -100 -110 -120 -130 -140 -150 -160 -170 -180 -190 -200 -210 -220 -230 -240
f1 (ppm)

2q

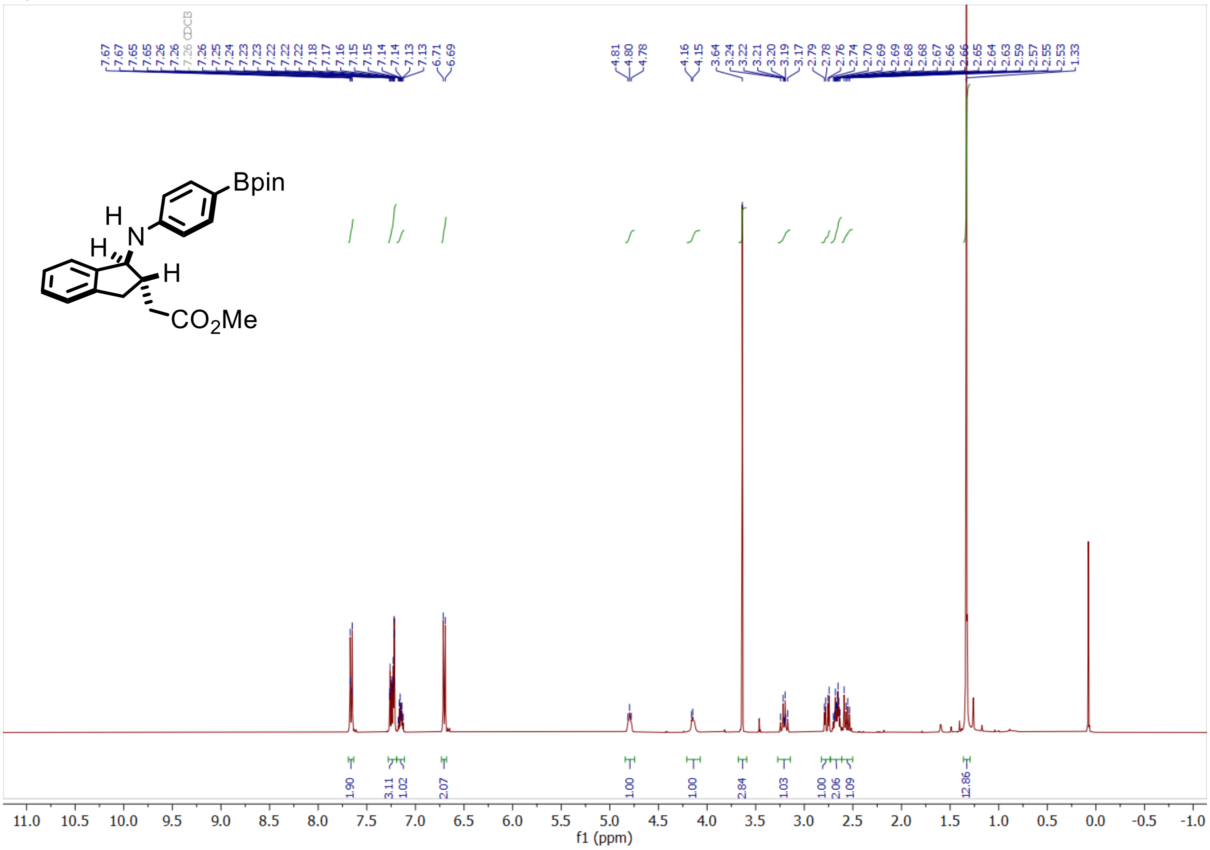
7.67 7.67 7.65 7.65 7.26 7.26 CDCl3 7.26 7.25 7.24 7.23 7.23 7.22 7.22 7.22 7.18 7.17 7.16 7.15 7.15 7.14 7.13 7.13 6.71 6.69
4.81 4.80 4.78
4.16 4.15 3.64 3.24 3.22 3.21 3.20 3.19 3.17 2.79 2.78 2.76 2.74 2.70 2.69 2.69 2.68 2.68 2.67 2.66 2.66 2.65 2.64 2.63 2.59 2.57 2.55 2.53 1.33
Bpin
H
N
H
H
CO2Me
1.90 3.11 1.02 2.07 1.00 1.00 2.84 1.03 1.00 2.06 1.09 12.86
11.0 10.5 10.0 9.5 9.0 8.5 8.0 7.5 7.0 6.5 6.0 5.5 5.0 4.5 4.0 3.5 3.0 2.5 2.0 1.5 1.0 0.5 0.0 -0.5 -1.0
f1 (ppm)

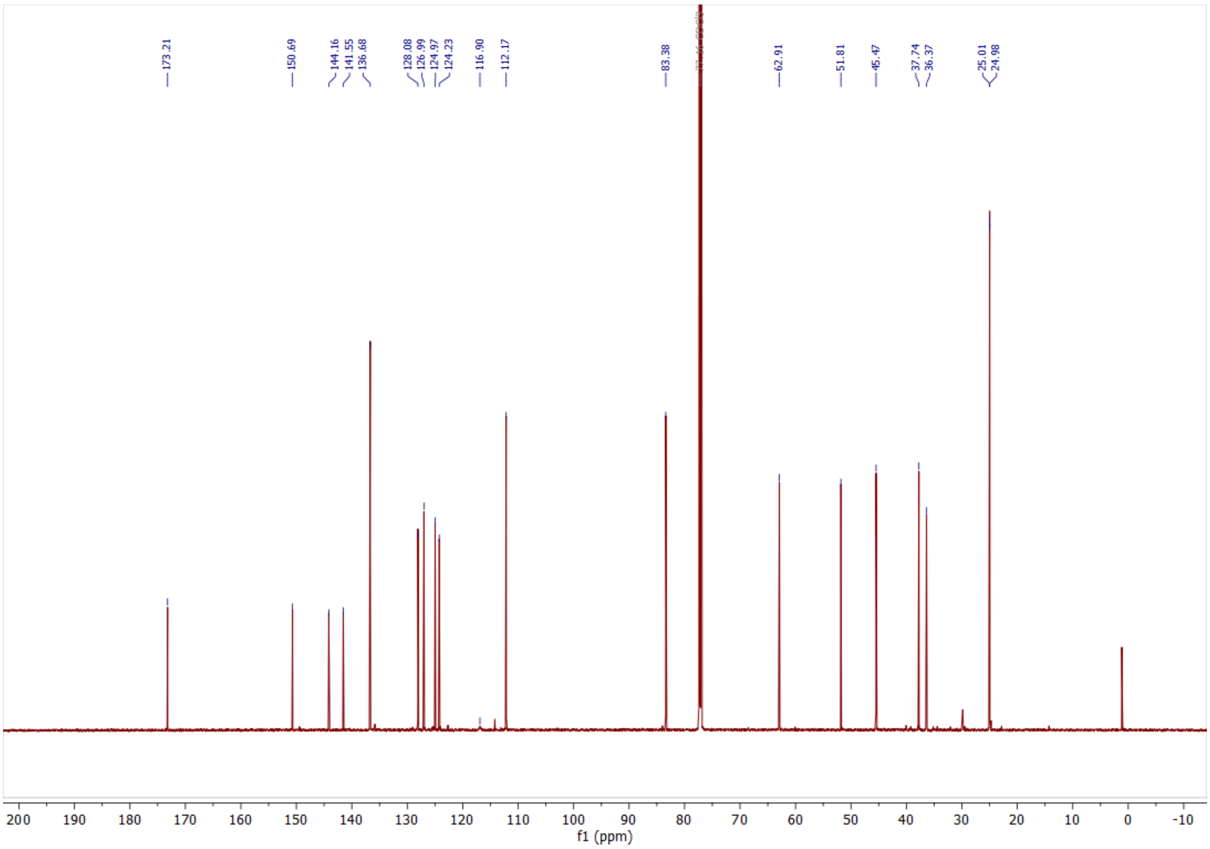
173.21
150.69
144.16 141.55 136.68
128.08 126.99 124.97 124.23
116.90
112.17
83.38
62.91
51.81
45.47
37.74 36.37
25.01 24.98
200 190 180 170 160 150 140 130 120 110 100 90 80 70 60 50 40 30 20 10 0 -10
f1 (ppm)

3a

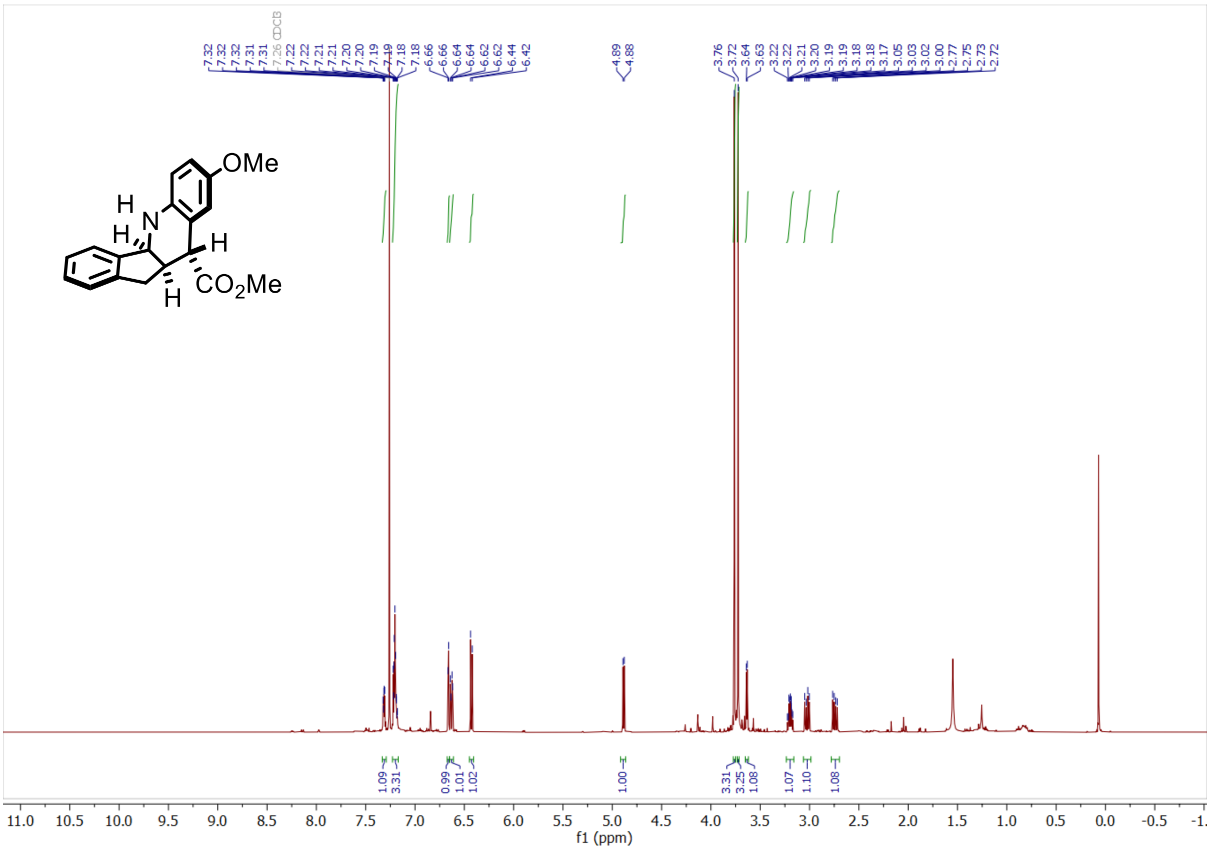

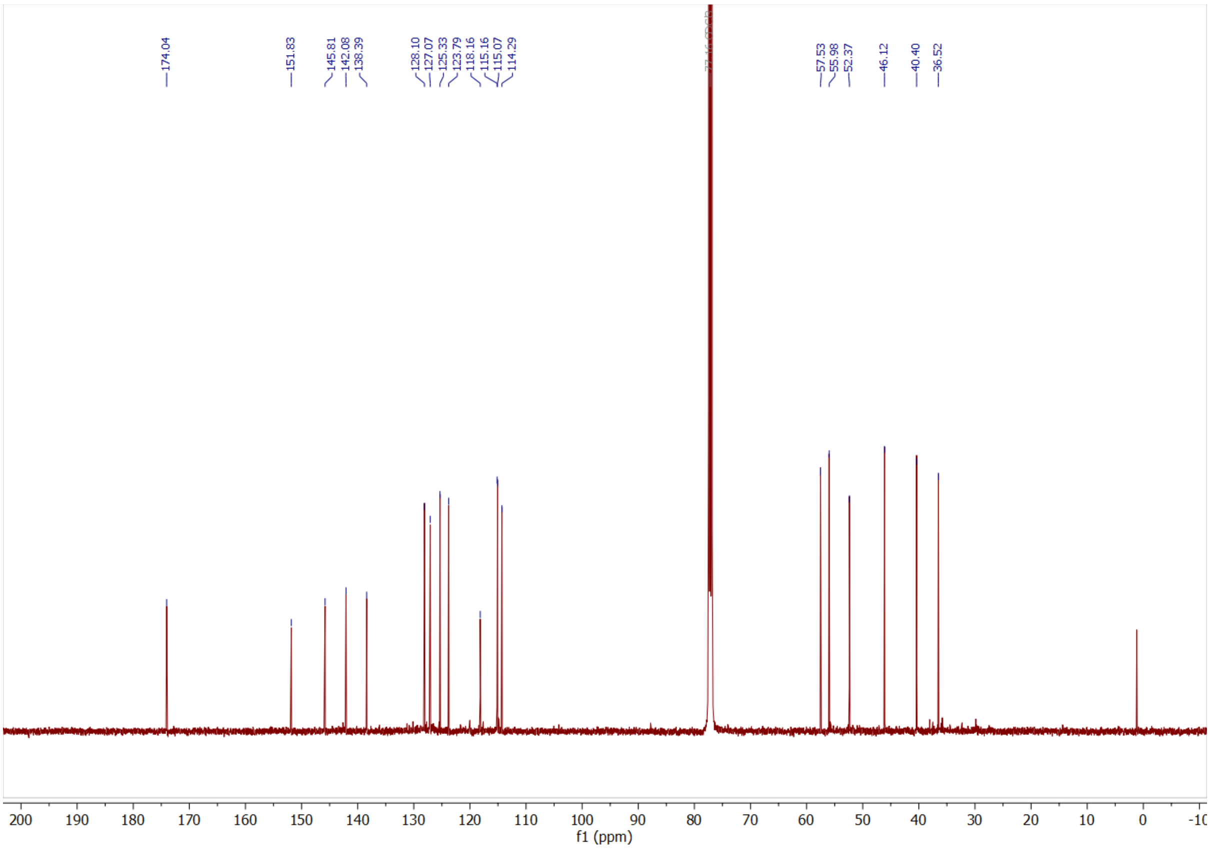

3b

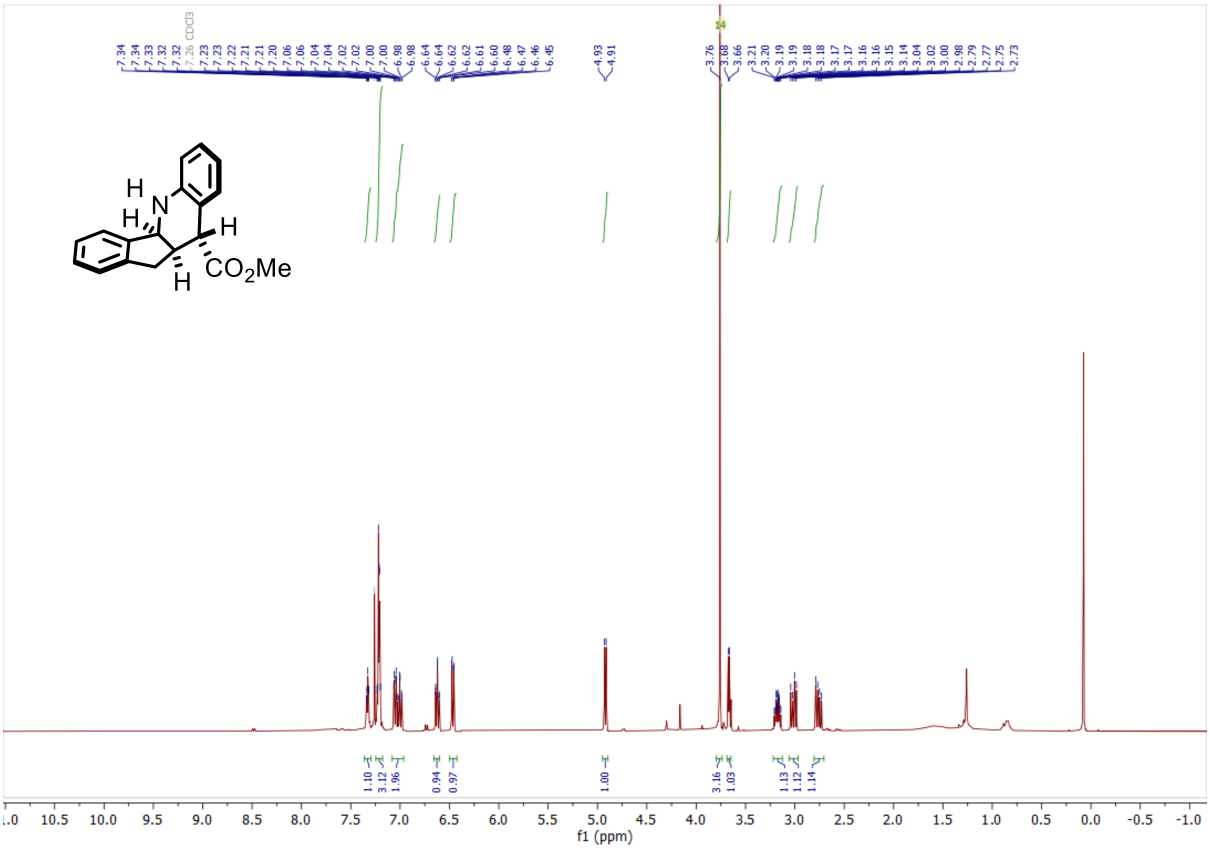

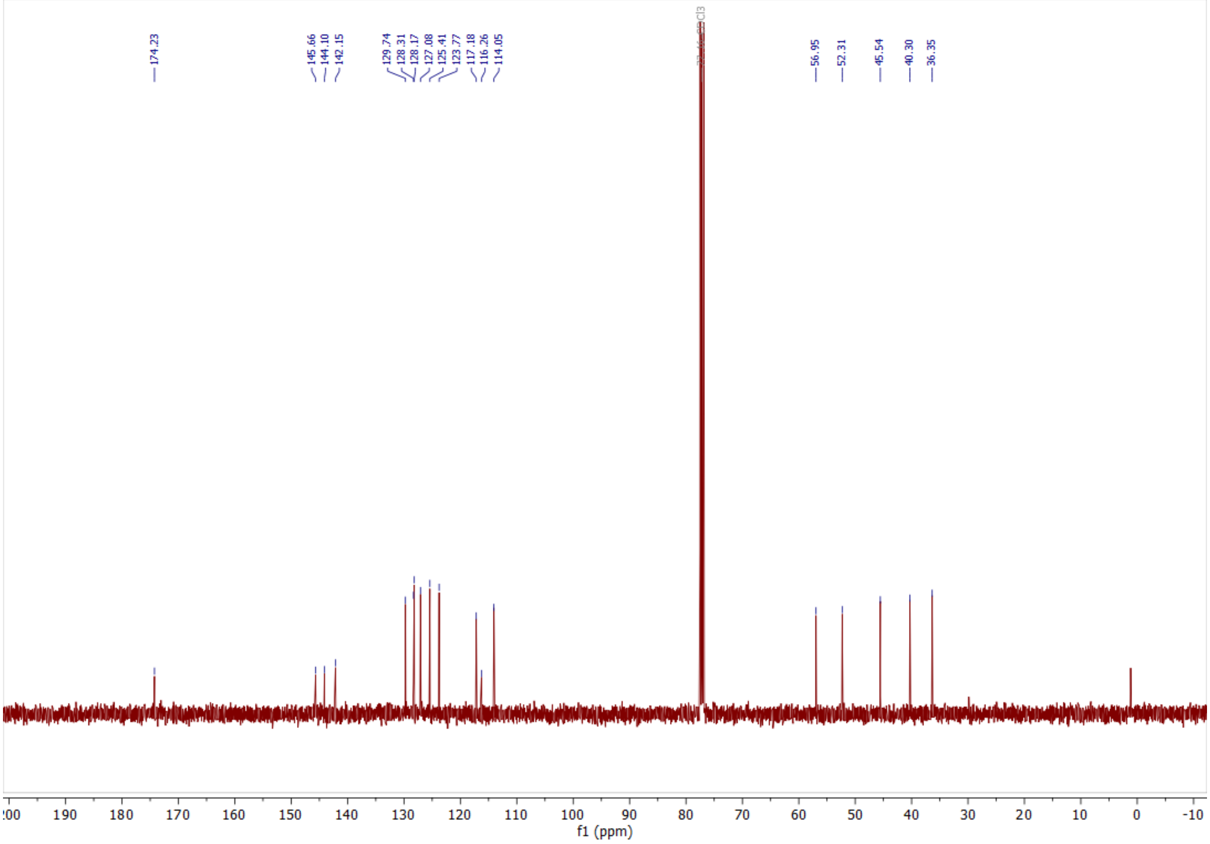

3c

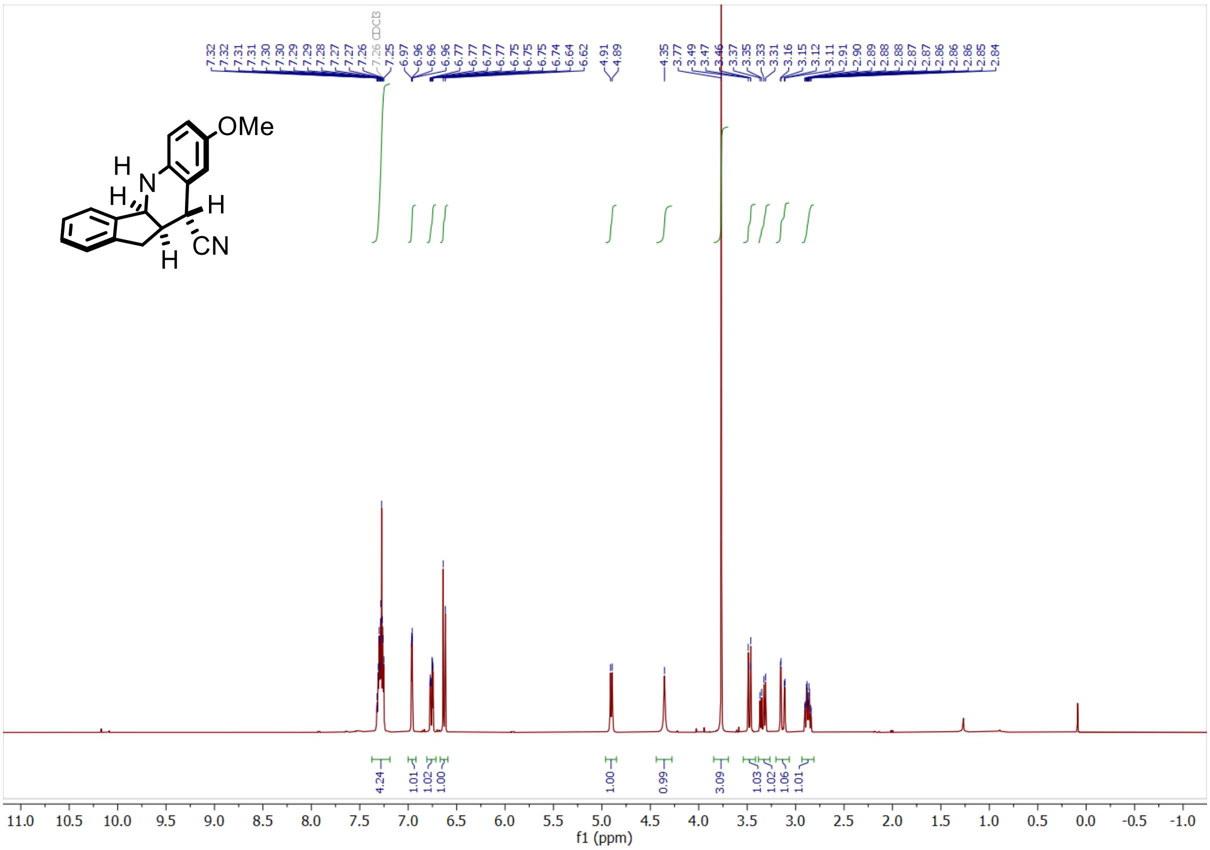
OMe
H
N
H
H
CN
H
7.32 7.32 7.31 7.31 7.30 7.30 7.29 7.29 7.29 7.28 7.27 7.27 7.26 7.26 CDCl3 7.25 6.97 6.96 6.96 6.96 6.77 6.77 6.77 6.75 6.75 6.75 6.74 6.64 6.62
4.91 4.89
4.35 3.77 3.49 3.47 3.46 3.37 3.35 3.33 3.31 3.16 3.15 3.12 3.11 2.91 2.90 2.89 2.88 2.88 2.87 2.87 2.86 2.86 2.86 2.85 2.84
4.24 1.01 1.02 1.00 1.00 0.99 3.09 1.03 1.02 1.06 1.01
f1 (ppm)

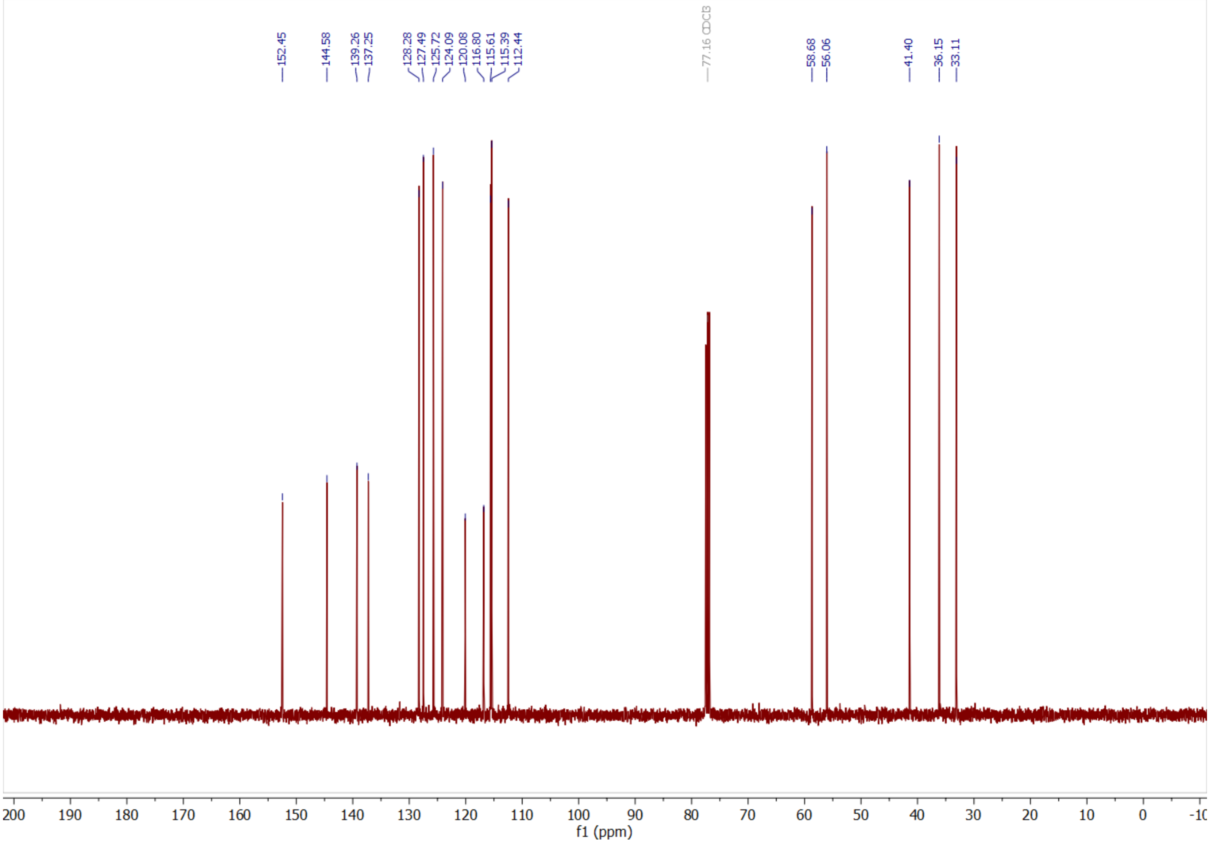
152.45
144.58
139.26 137.25
128.28 127.49 125.72 124.09 120.08 116.80 115.61 115.39 112.44
77.16 CDCl3
58.68 56.06
41.40 36.15 33.11
f1 (ppm)

3d

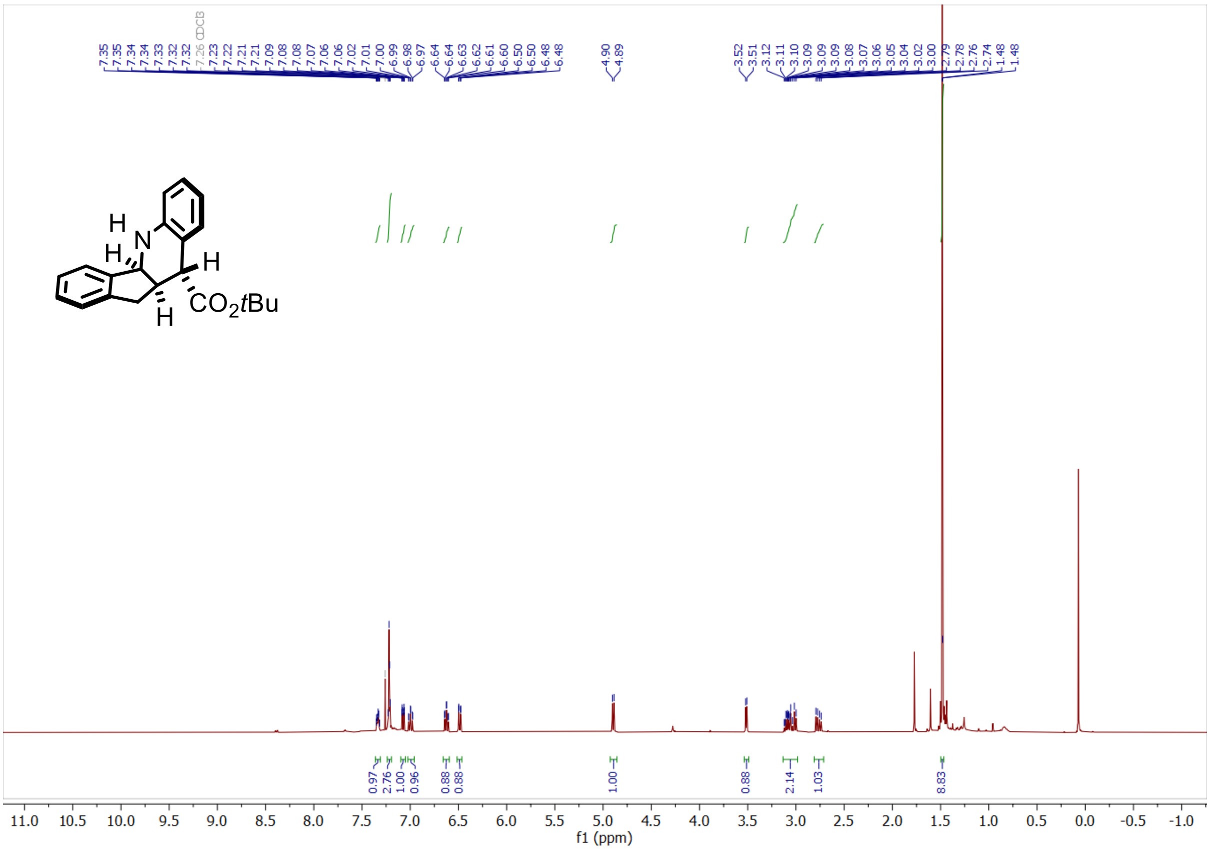

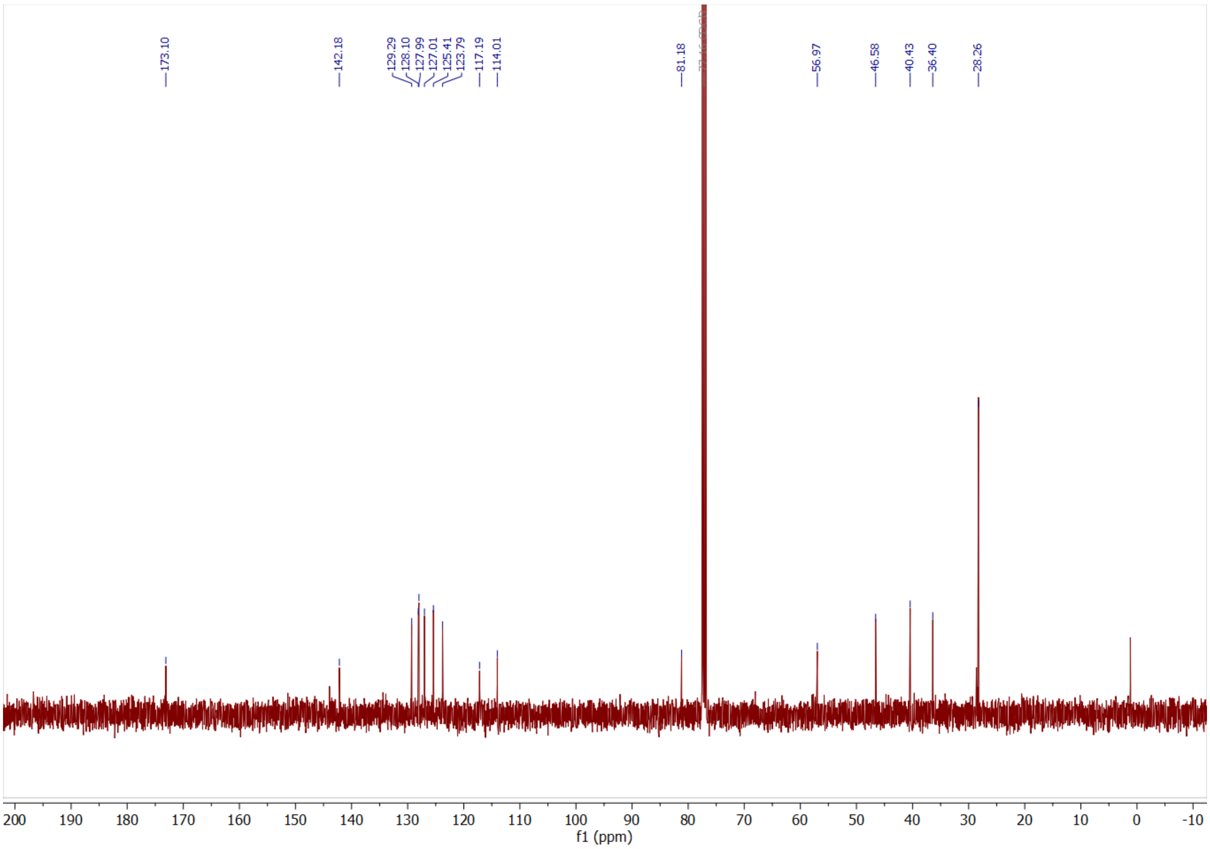

3e

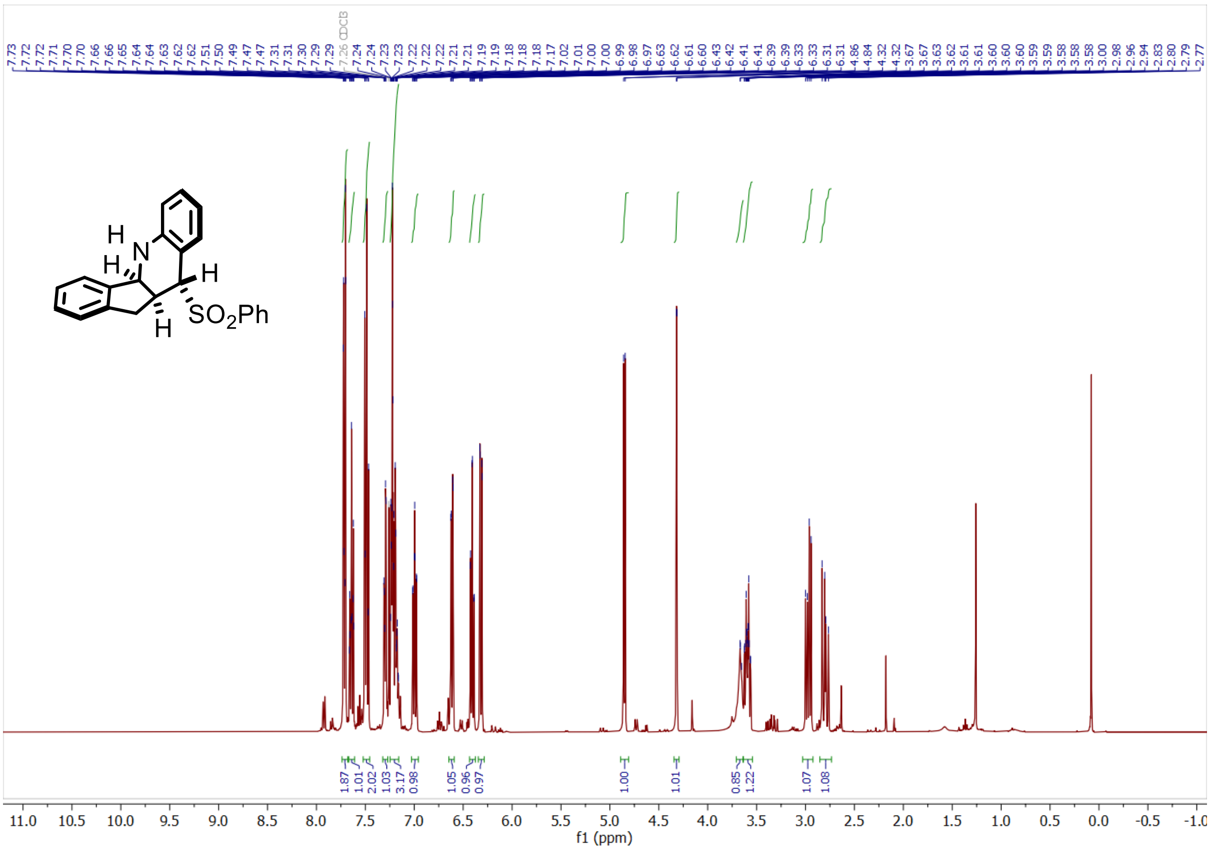

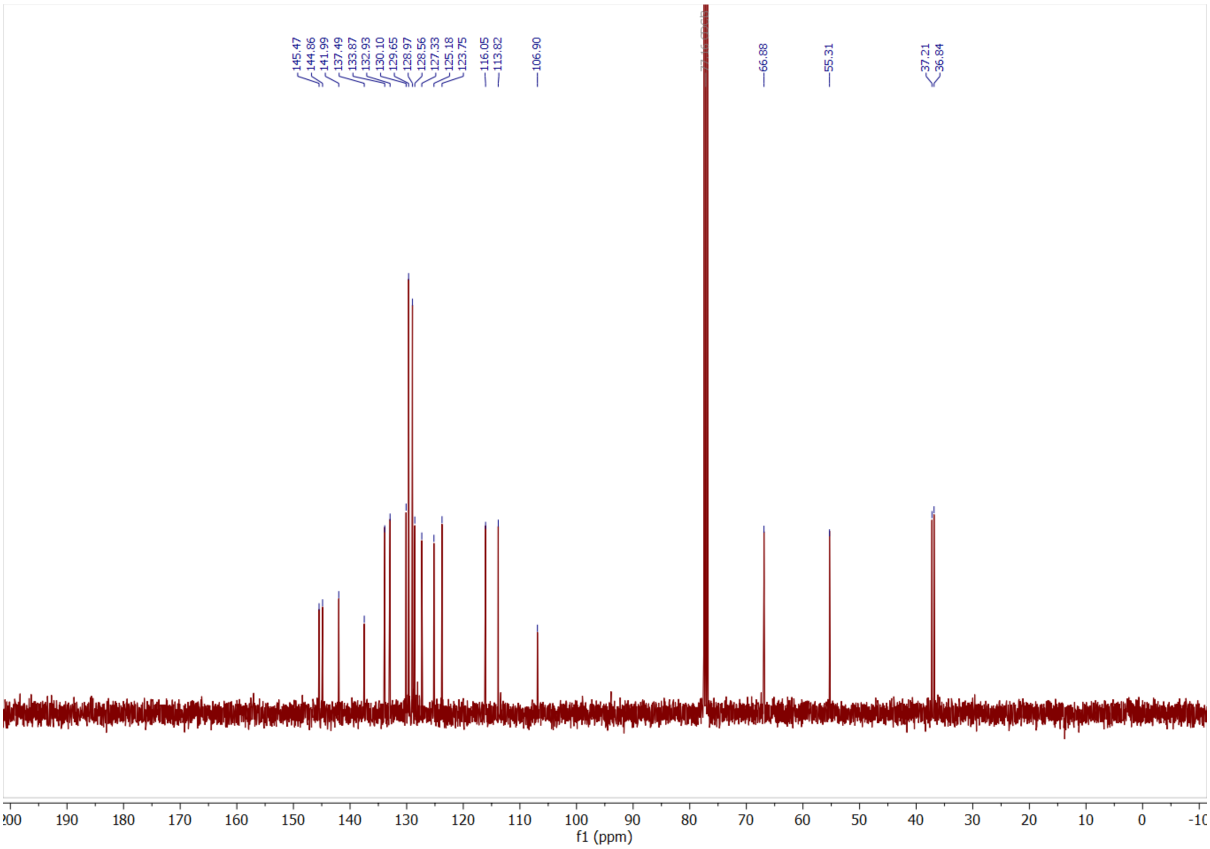

3f

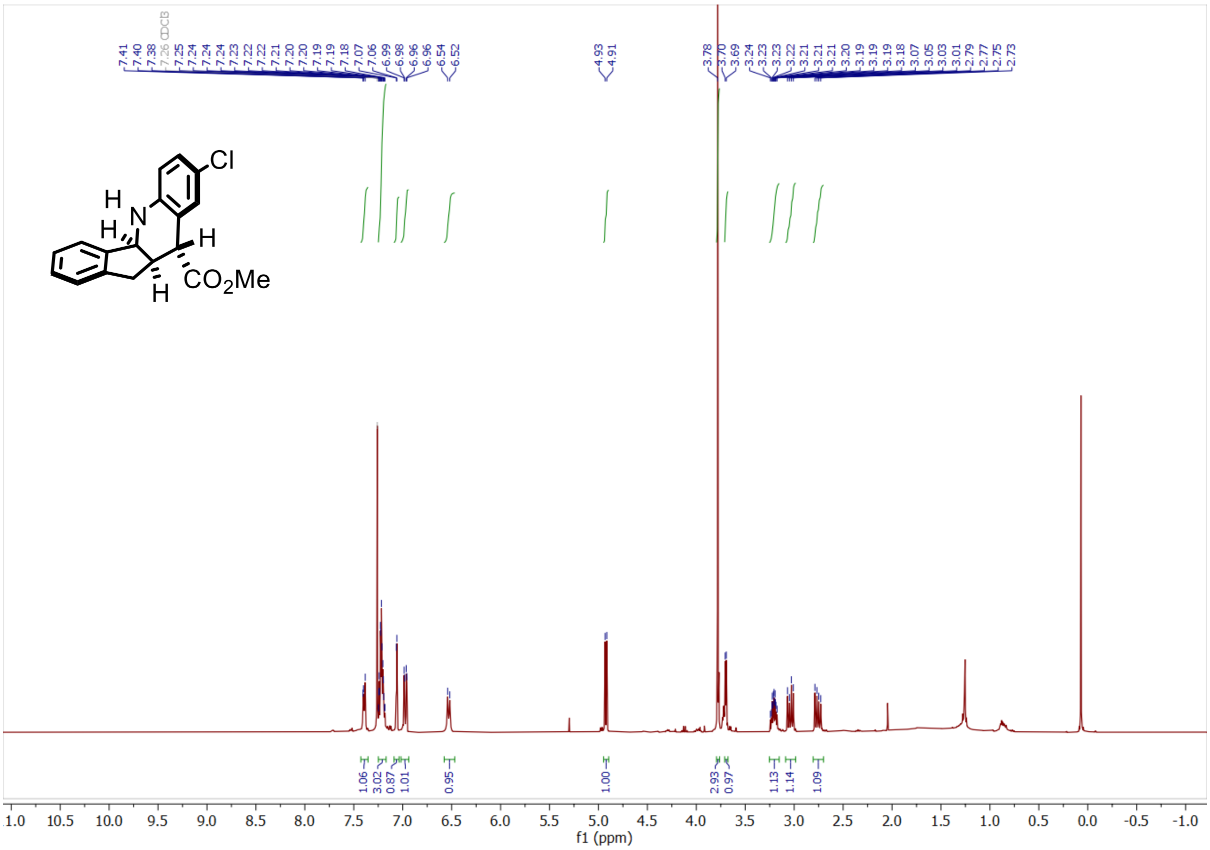

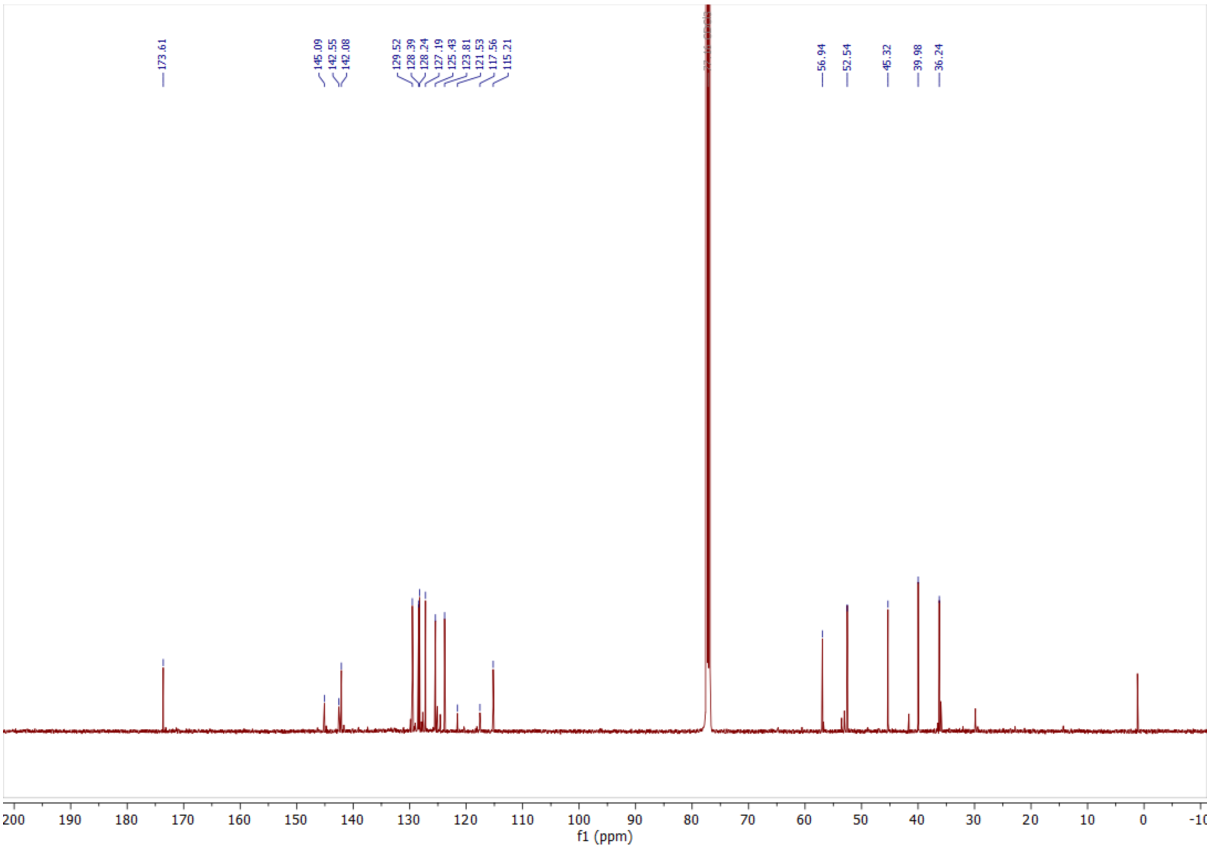

3g

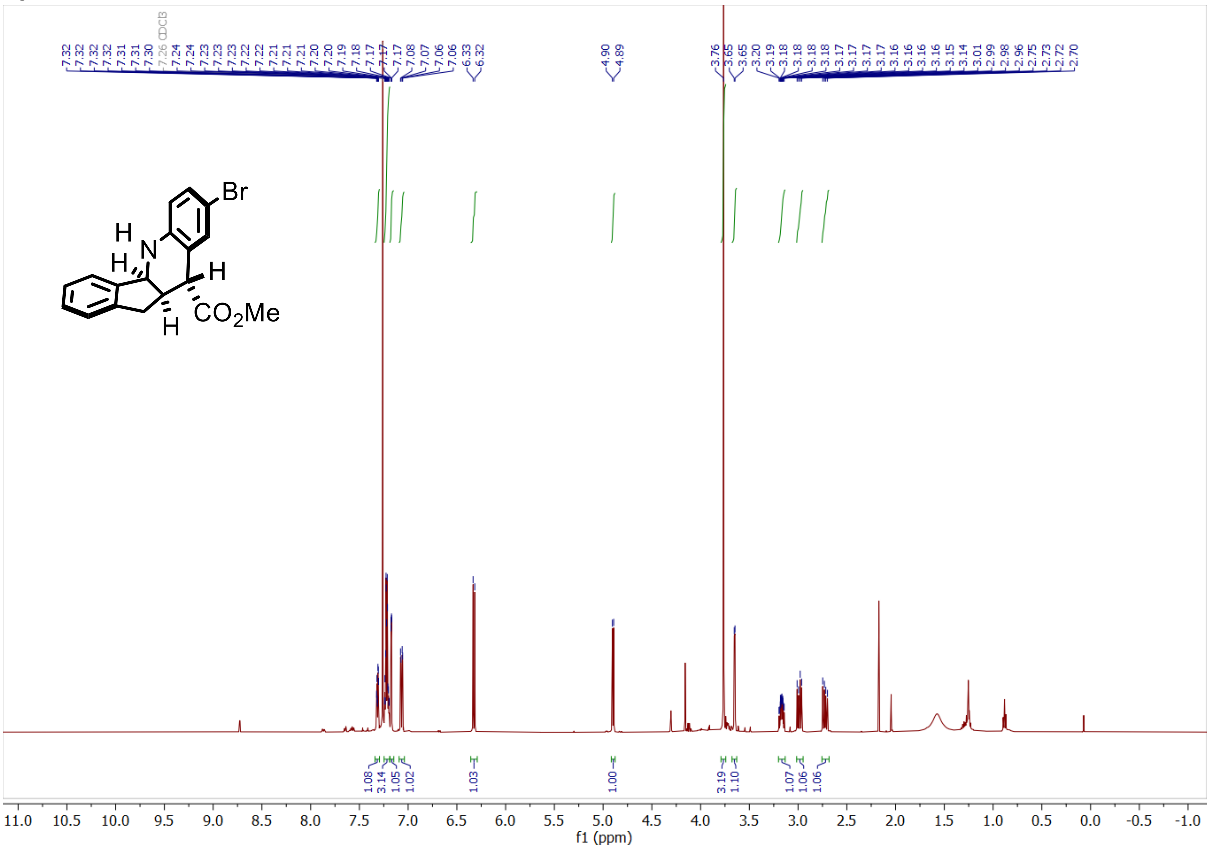

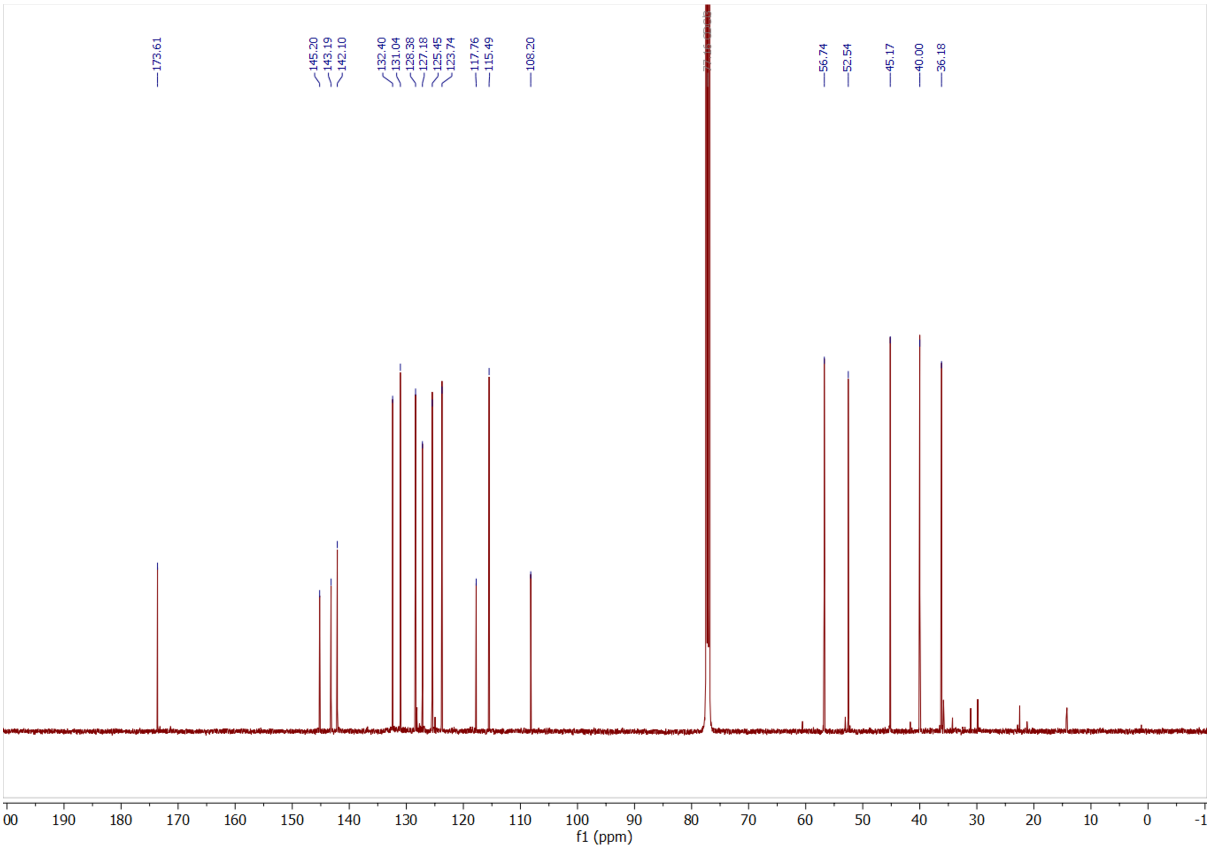

3h-1

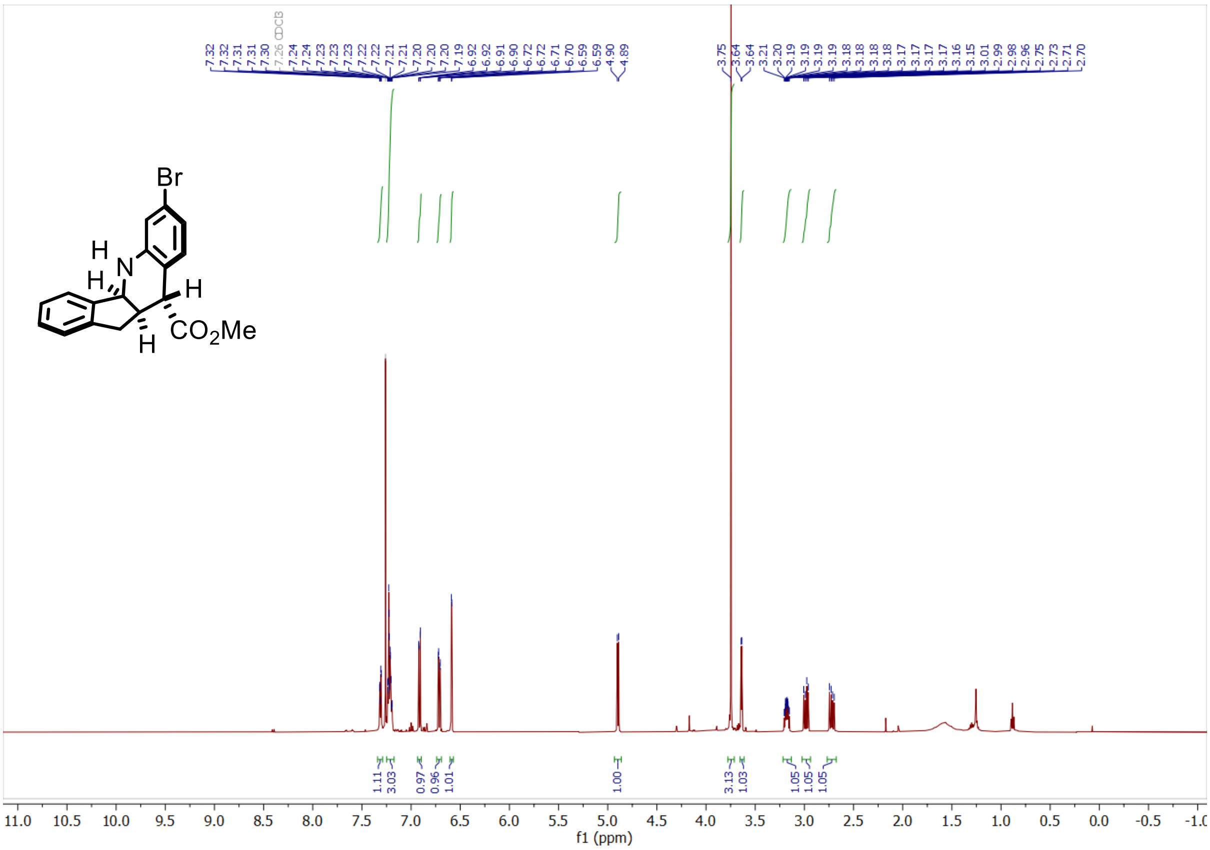

7.32
7.32
7.31
7.31
7.30
7.26 CDCl3
7.24
7.24
7.23
7.23
7.23
7.23
7.22
7.22
7.21
7.21
7.20
7.20
7.20
7.19
6.92
6.92
6.91
6.90
6.72
6.72
6.71
6.70
6.59
4.90
4.89
3.75
3.64
3.64
3.21
3.20
3.19
3.19
3.19
3.19
3.18
3.18
3.18
3.17
3.17
3.17
3.17
3.16
3.15
3.01
2.99
2.98
2.96
2.75
2.73
2.71
2.70
Br
H
N
H
H
H
CO2Me
1.11
3.03
0.97
0.96
1.01
1.00
3.13
1.03
1.05
1.05
1.05
11.0 10.5 10.0 9.5 9.0 8.5 8.0 7.5 7.0 6.5 6.0 5.5 5.0 4.5 4.0 3.5 3.0 2.5 2.0 1.5 1.0 0.5 0.0 -0.5 -1.0
f1 (ppm)

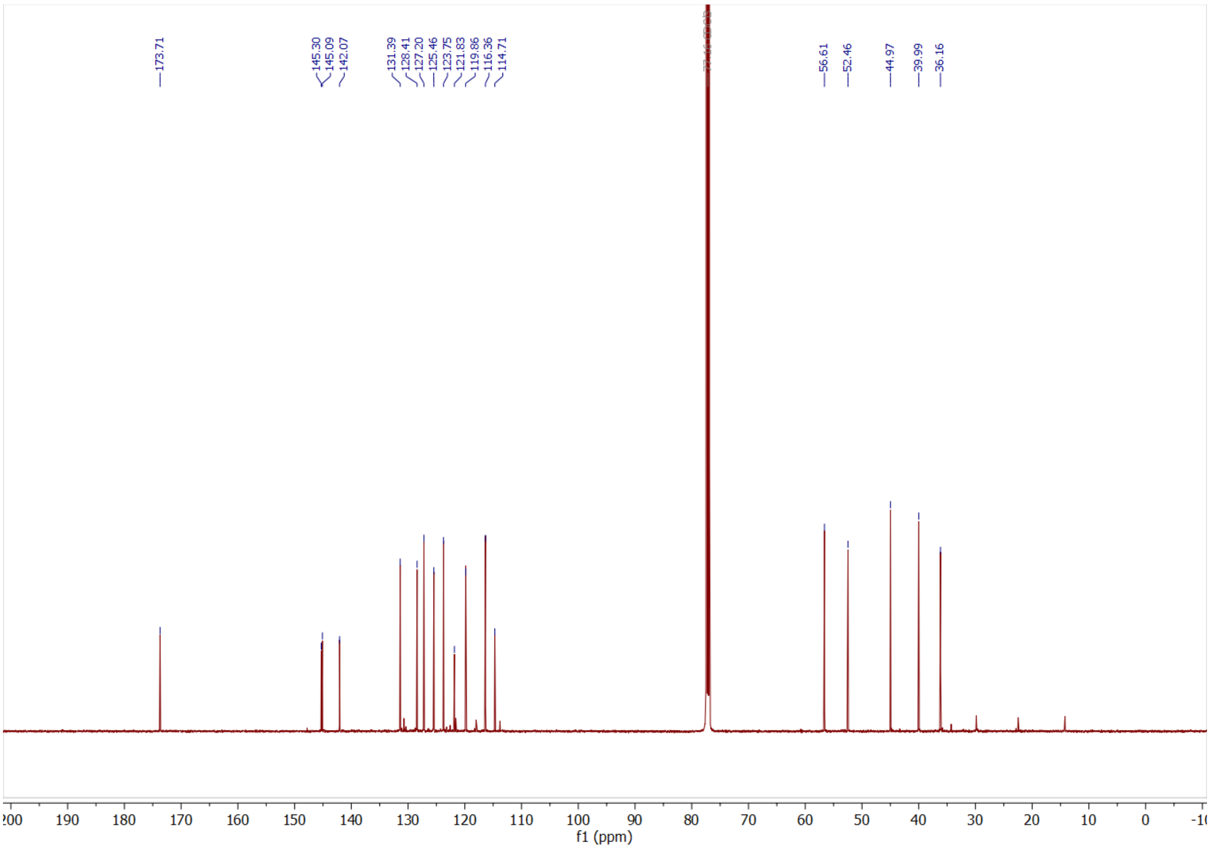

173.71
145.30
145.09
142.07
131.39
128.41
127.20
125.46
123.75
121.83
119.86
116.36
114.71
56.61
52.46
44.97
39.99
36.16
200 190 180 170 160 150 140 130 120 110 100 90 80 70 60 50 40 30 20 10 0 -10
f1 (ppm)

3h-2

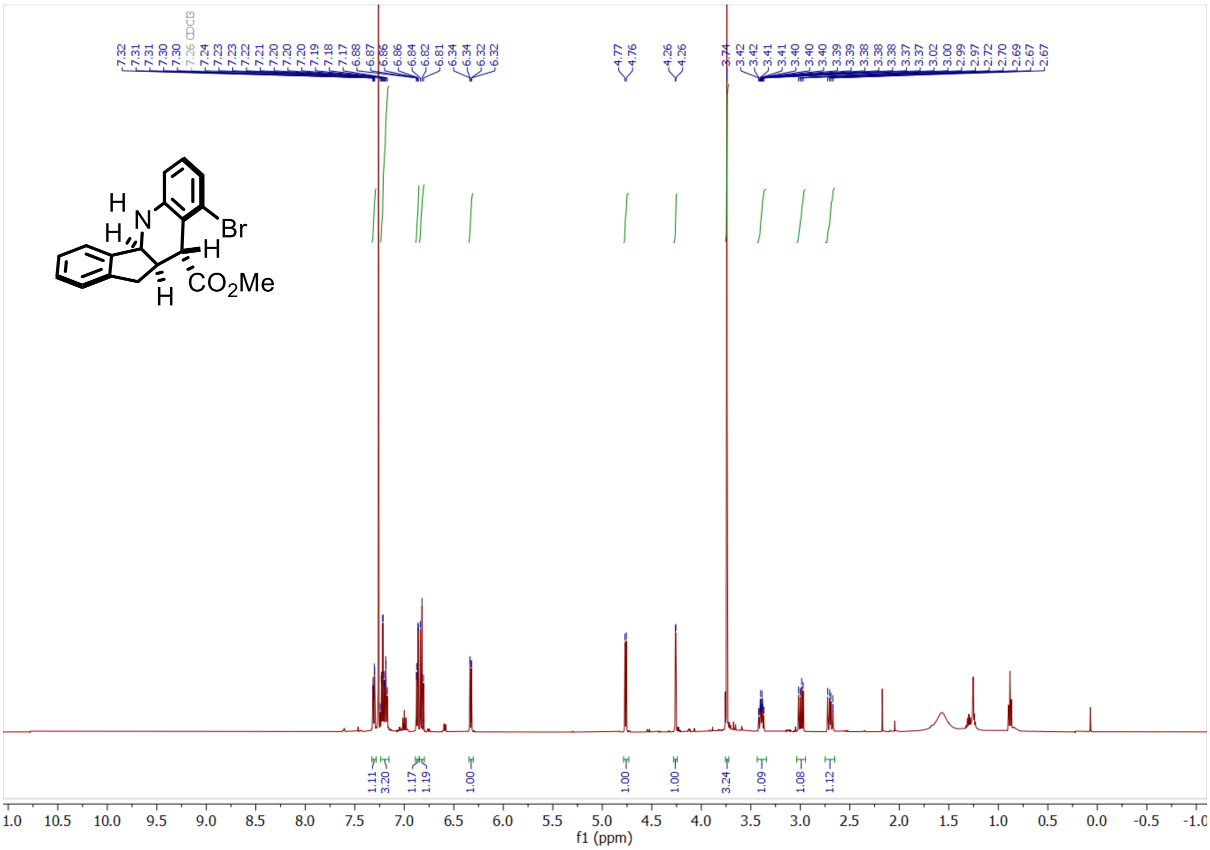

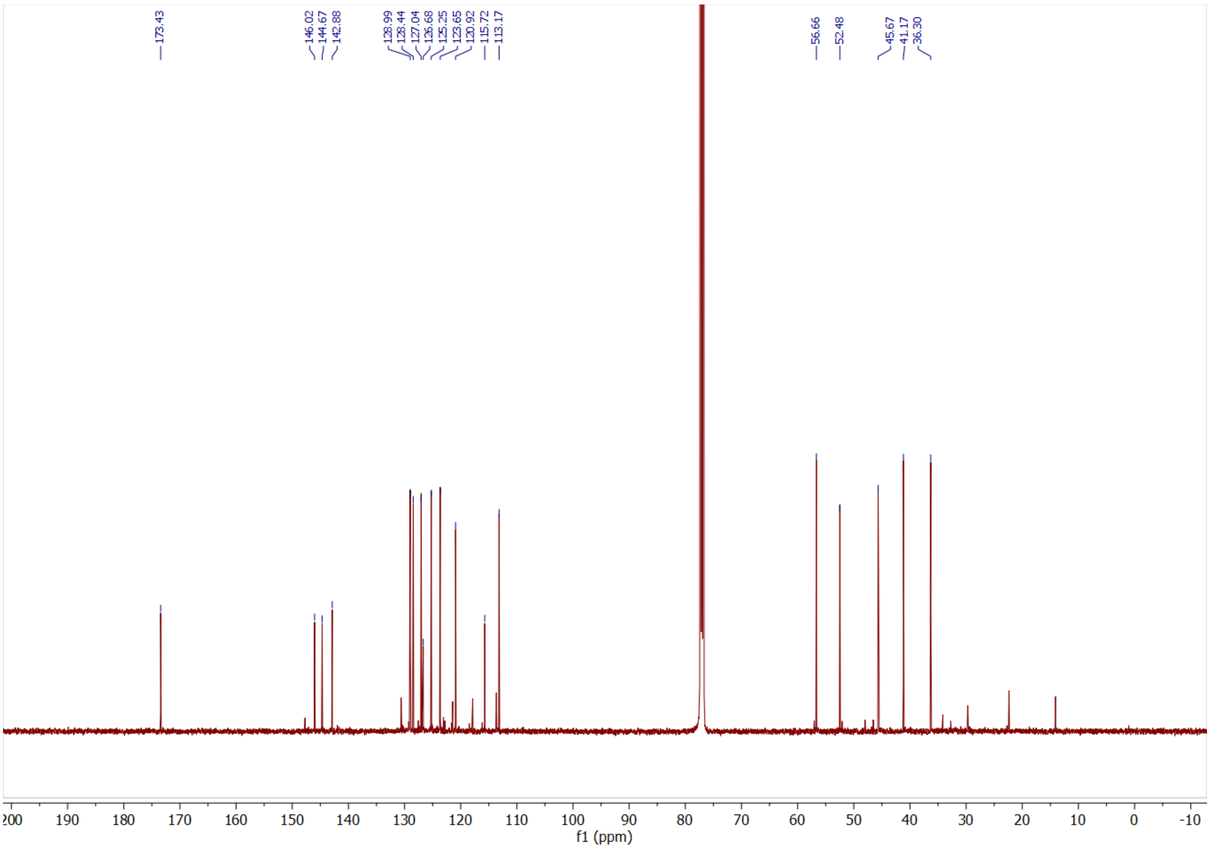

3i

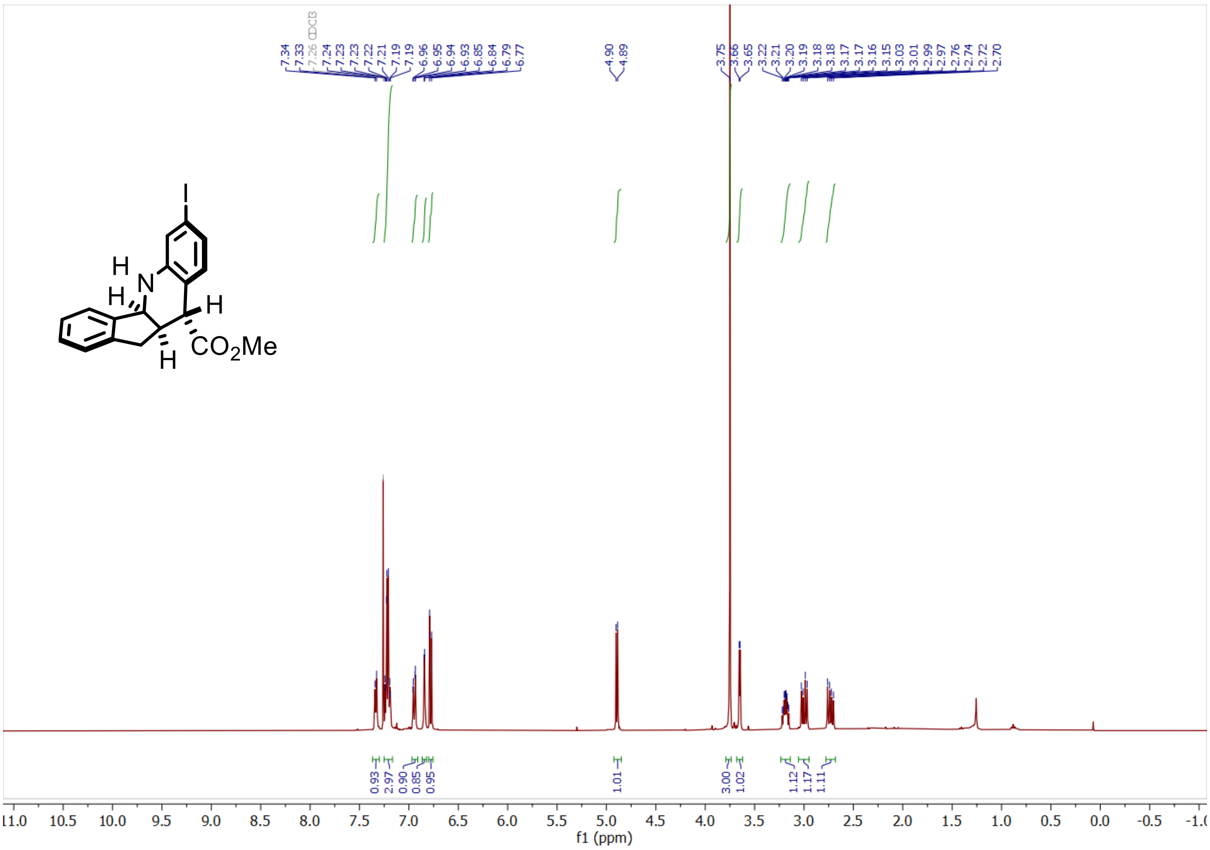
7.34 7.33 7.26 CDCl3 7.24 7.23 7.23 7.22 7.21 7.19 7.19 6.96 6.95 6.94 6.93 6.85 6.84 6.79 6.77
4.90 4.89
3.75 3.66 3.65 3.22 3.21 3.20 3.19 3.18 3.18 3.17 3.16 3.15 3.03 3.01 2.99 2.97 2.76 2.74 2.72 2.70
H
N
H
H
CO2Me
H
I
0.93 2.97 0.90 0.85 0.95 1.01 3.00 1.02 1.12 1.17 1.11
f1 (ppm)

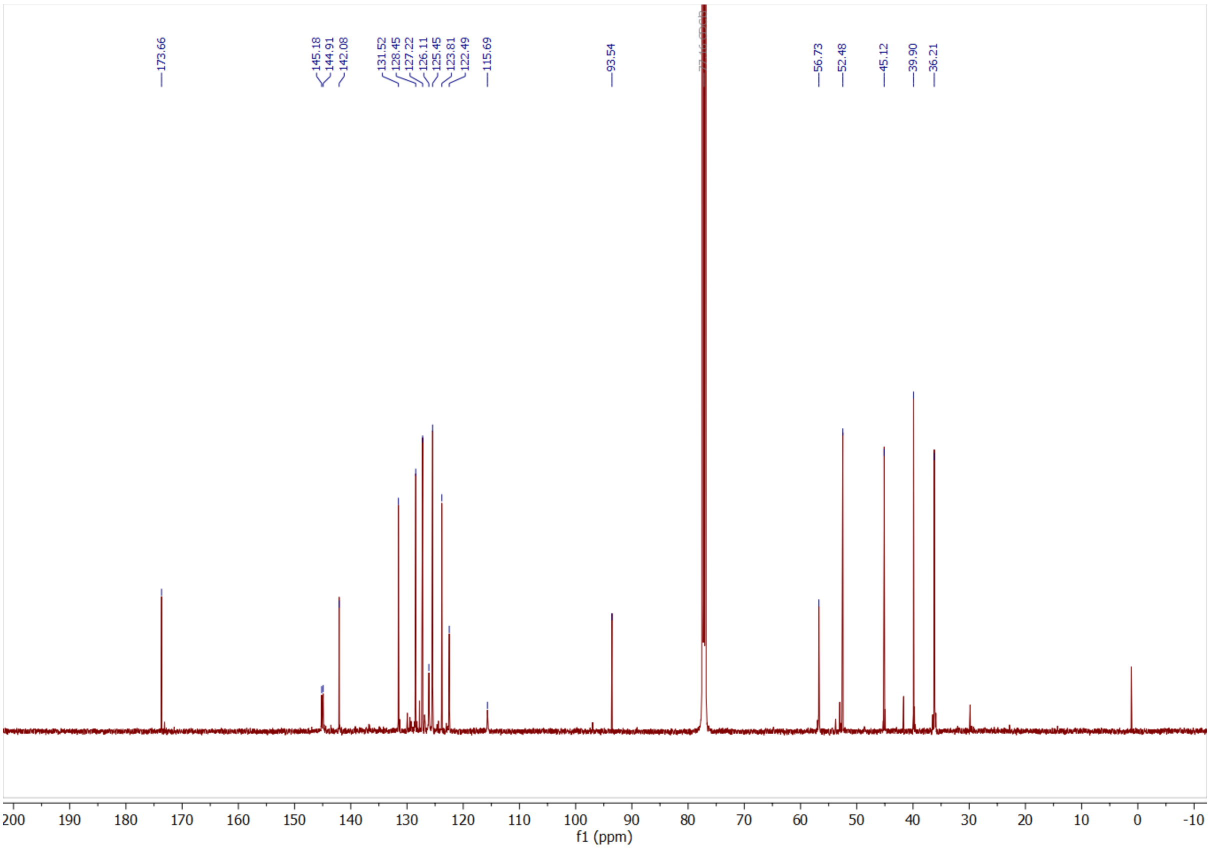
173.66
145.18 144.91 142.08
131.52 128.45 127.22 126.11 125.45 123.81 122.49 115.69
93.54
56.73 52.48 45.12 39.90 36.21
f1 (ppm)

3j

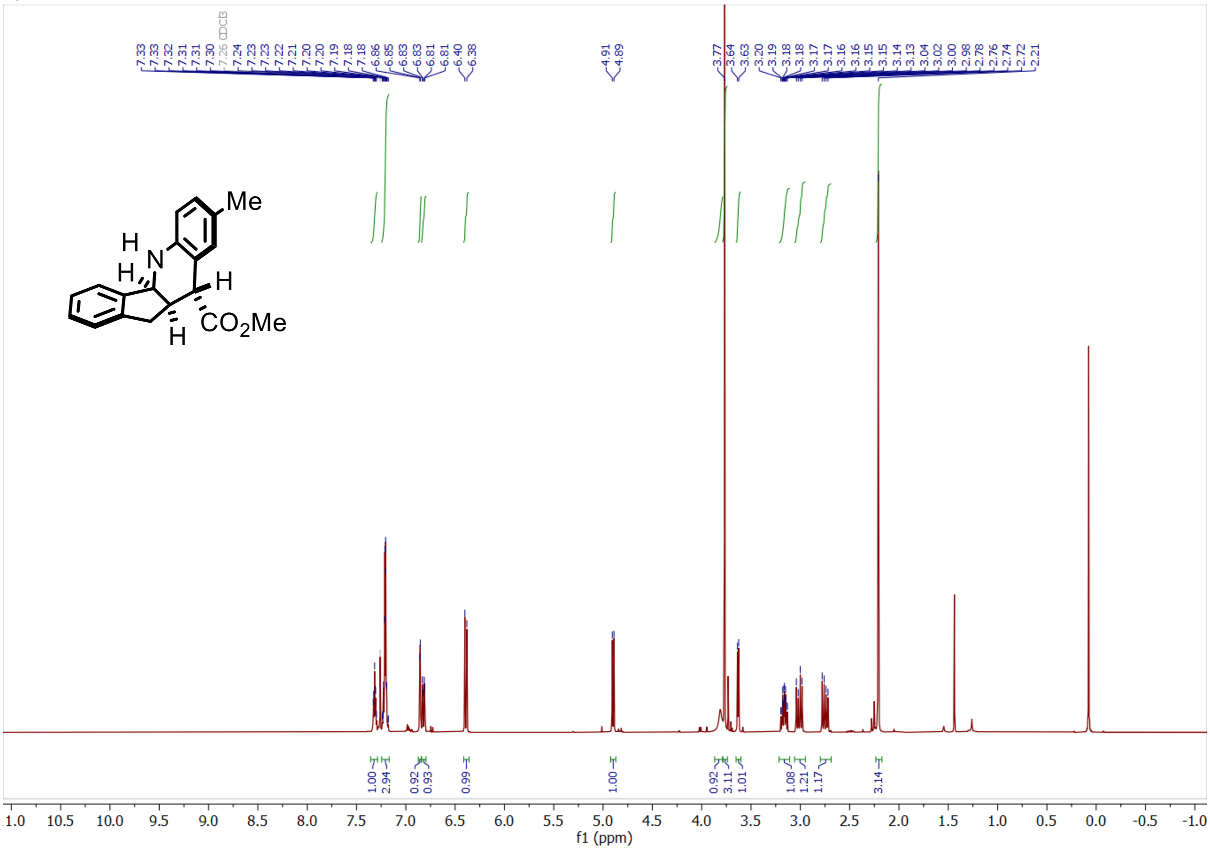
7.33
7.33
7.32
7.31
7.31
7.30
7.26 CDCl3
7.24
7.23
7.23
7.22
7.21
7.20
7.20
7.19
7.18
7.18
6.86
6.85
6.83
6.83
6.81
6.81
6.40
6.38
4.91
4.89
3.77
3.64
3.63
3.20
3.19
3.18
3.18
3.17
3.17
3.16
3.15
3.15
3.14
3.13
3.04
3.02
3.00
2.98
2.78
2.76
2.74
2.72
2.21
Me
H
N
H
H
CO2Me
H
1.00
2.94
0.92
0.93
0.99
1.00
0.92
3.11
1.01
1.08
1.21
1.17
3.14
1.0
10.5
10.0
9.5
9.0
8.5
8.0
7.5
7.0
6.5
6.0
5.5
5.0
4.5
4.0
3.5
3.0
2.5
2.0
1.5
1.0
0.5
0.0
-0.5
-1.0
f1 (ppm)

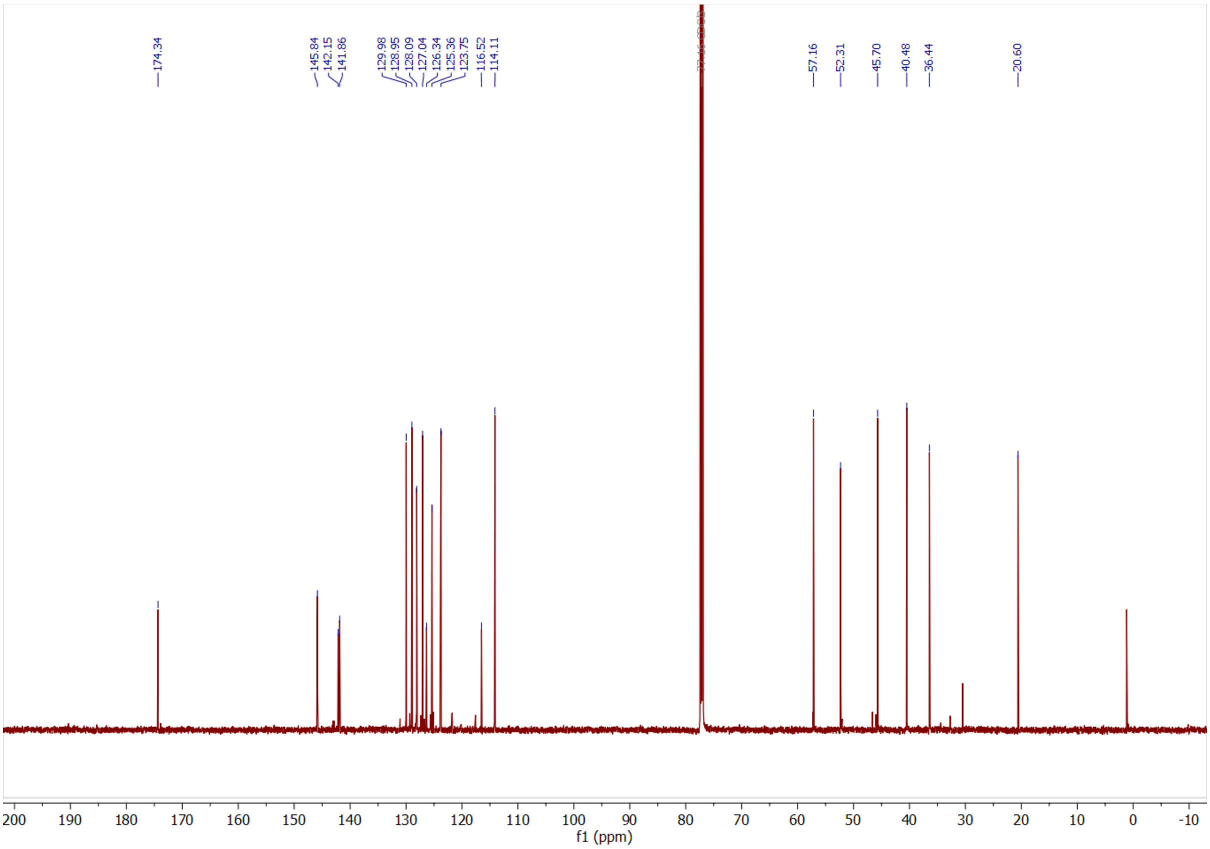
174.34
145.84
142.15
141.86
129.98
128.95
128.09
127.04
126.34
125.36
123.75
116.52
114.11
57.16
52.31
45.70
40.48
36.44
20.60
200
190
180
170
160
150
140
130
120
110
100
90
80
70
60
50
40
30
20
10
0
-10
f1 (ppm)

3k

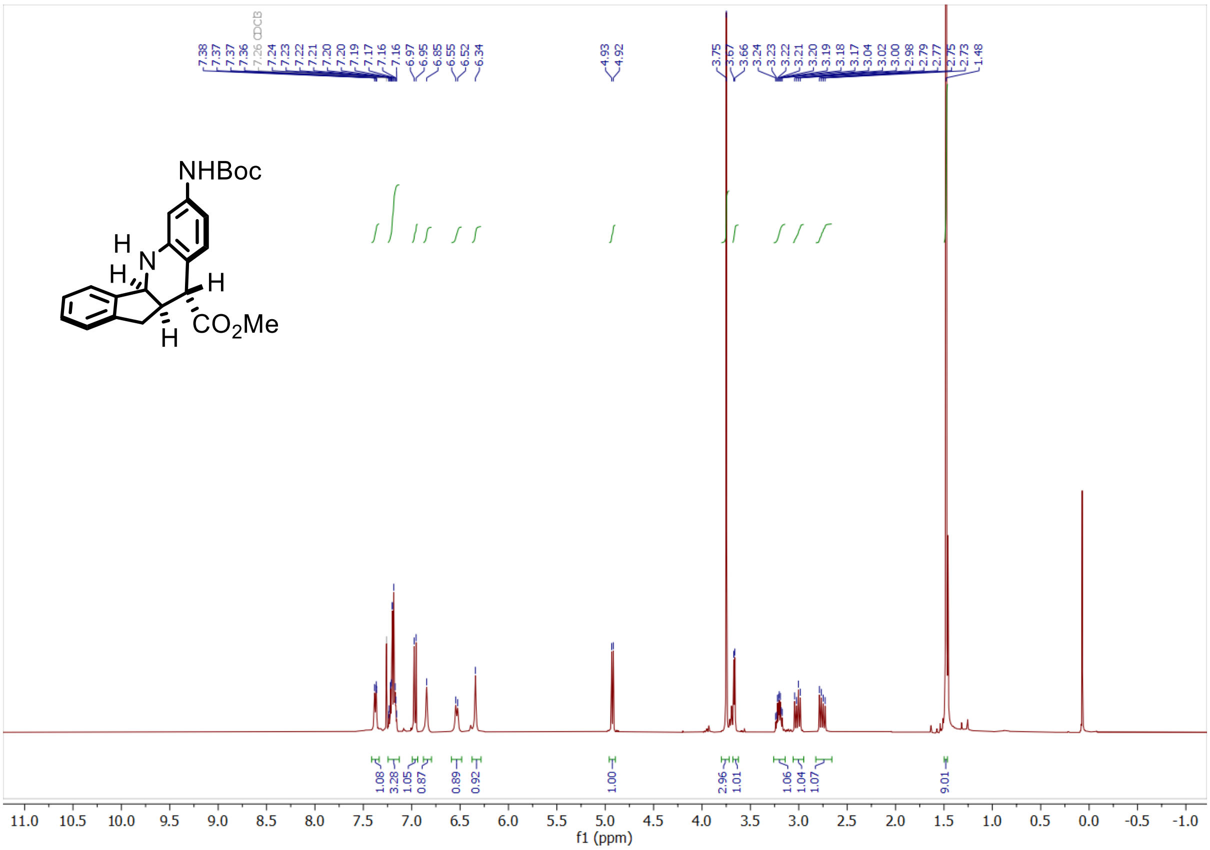
NHBoc
H
N
H
H
CO2Me
H
7.38
7.37
7.37
7.36
7.26 CDCl3
7.24
7.23
7.22
7.21
7.20
7.20
7.19
7.17
7.16
7.16
6.97
6.95
6.85
6.55
6.52
6.34
4.93
4.92
3.75
3.67
3.66
3.24
3.23
3.22
3.21
3.20
3.19
3.18
3.17
3.04
3.02
3.00
2.98
2.79
2.77
2.75
2.73
1.48
1.08
3.28
1.05
0.87
0.89
0.92
1.00
2.96
1.01
1.06
1.04
1.07
9.01
11.0 10.5 10.0 9.5 9.0 8.5 8.0 7.5 7.0 6.5 6.0 5.5 5.0 4.5 4.0 3.5 3.0 2.5 2.0 1.5 1.0 0.5 0.0 -0.5 -1.0
f1 (ppm)

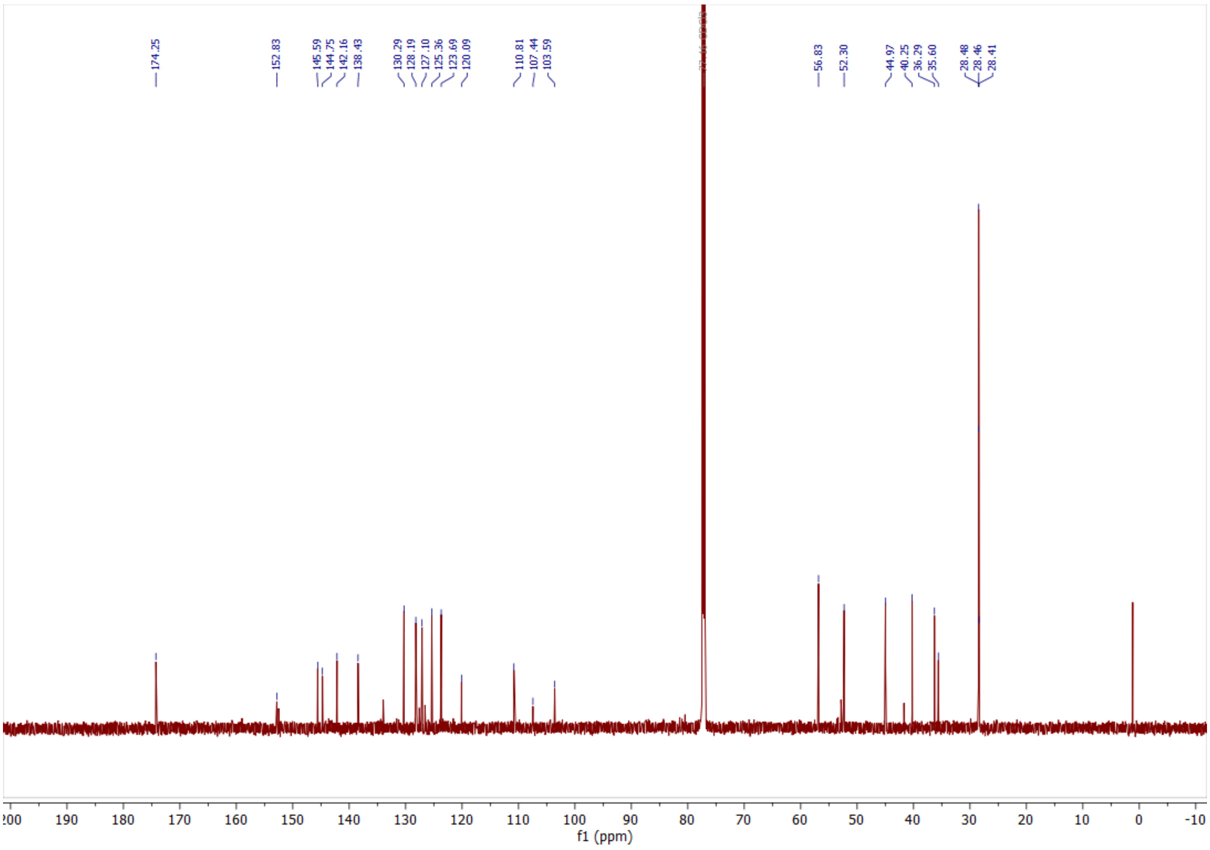
174.25
152.83
145.59
144.75
142.16
138.43
130.29
128.19
127.10
125.36
123.69
120.09
110.81
107.44
103.59
56.83
52.30
44.97
40.25
36.29
35.60
28.48
28.46
28.41
200 190 180 170 160 150 140 130 120 110 100 90 80 70 60 50 40 30 20 10 0 -10
f1 (ppm)

3l

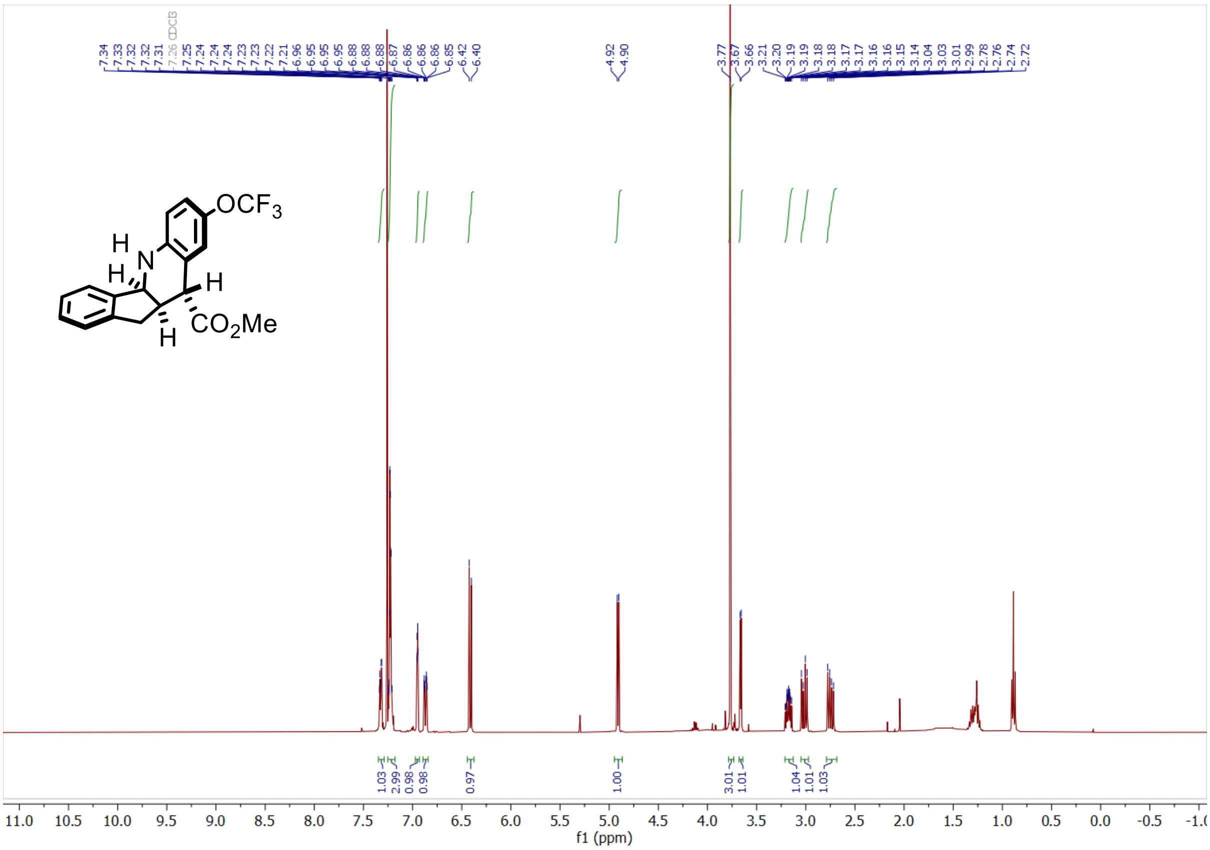

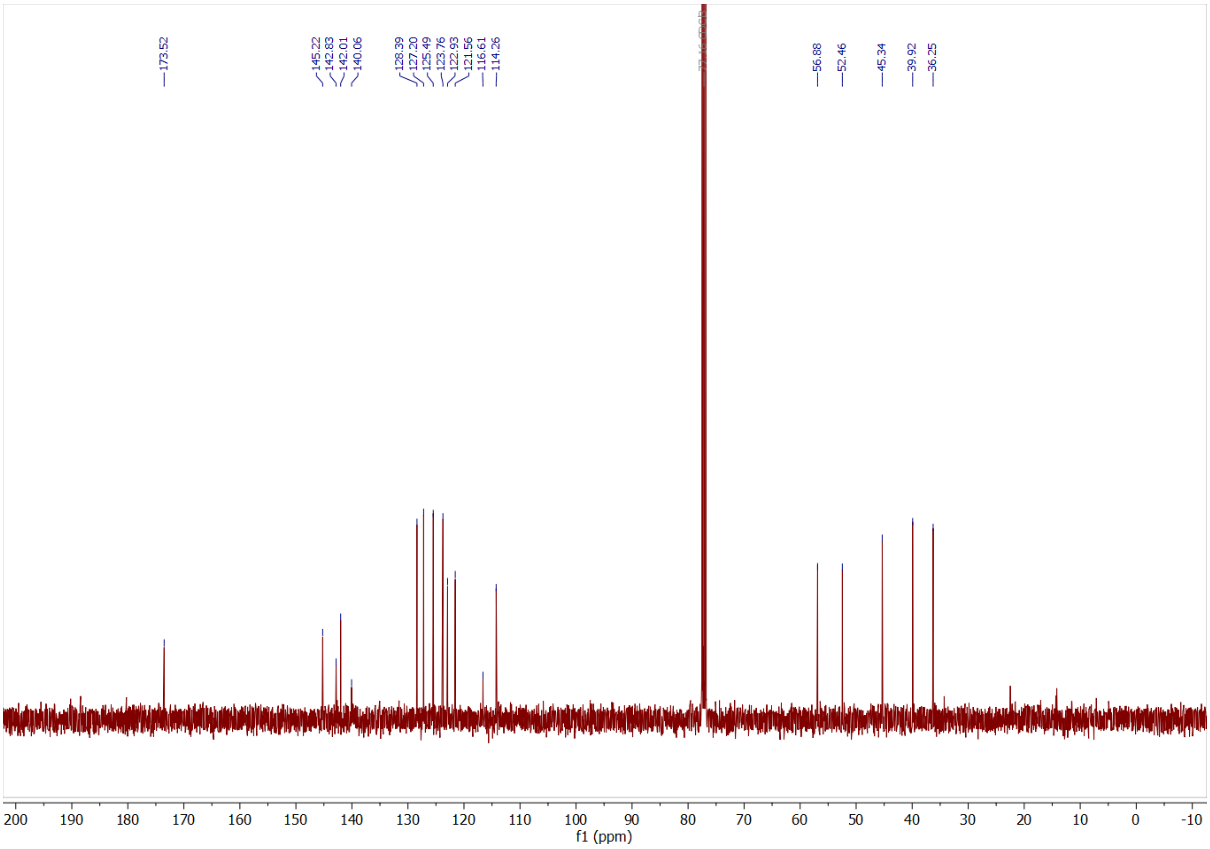

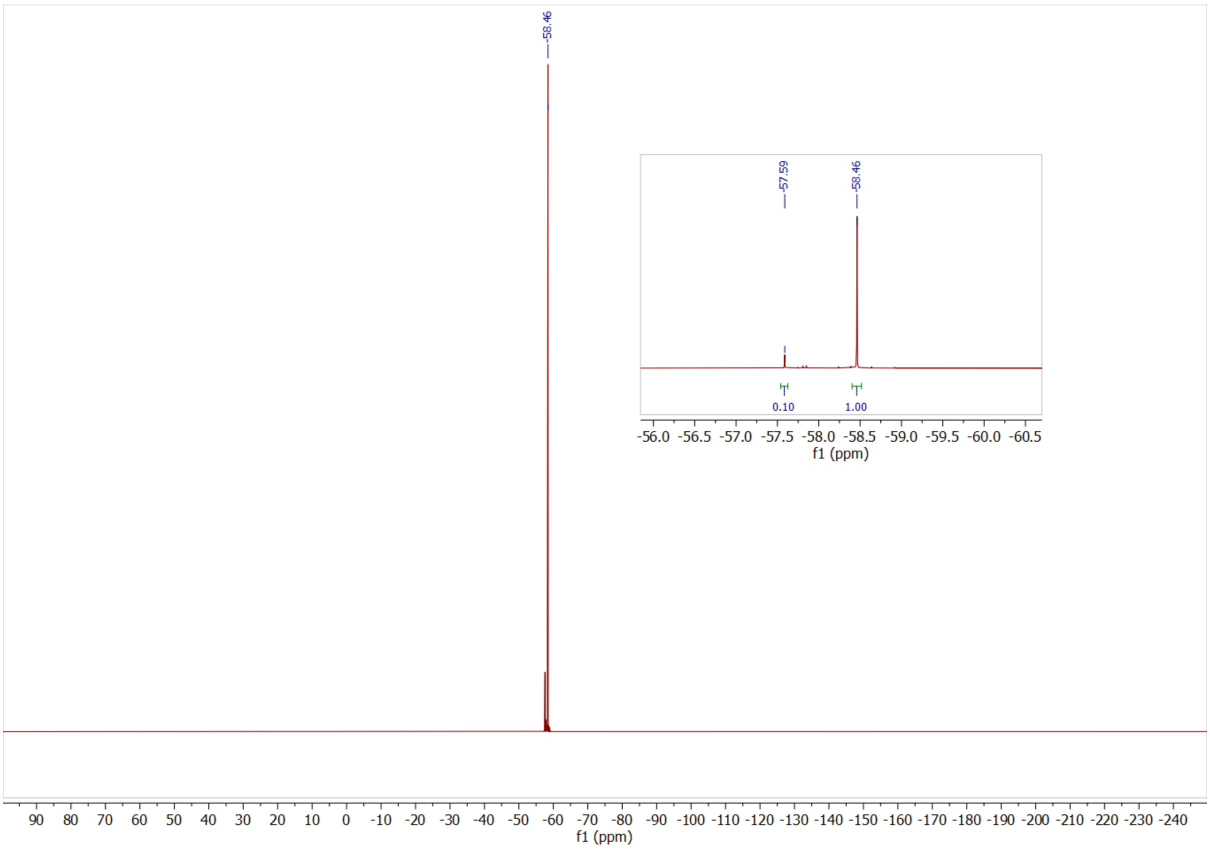
-58.46
-57.59
-58.46
0.10
1.00
-56.0 -56.5 -57.0 -57.5 -58.0 -58.5 -59.0 -59.5 -60.0 -60.5
f1 (ppm)
90 80 70 60 50 40 30 20 10 0 -10 -20 -30 -40 -50 -60 -70 -80 -90 -100 -110 -120 -130 -140 -150 -160 -170 -180 -190 -200 -210 -220 -230 -240
f1 (ppm)

3m

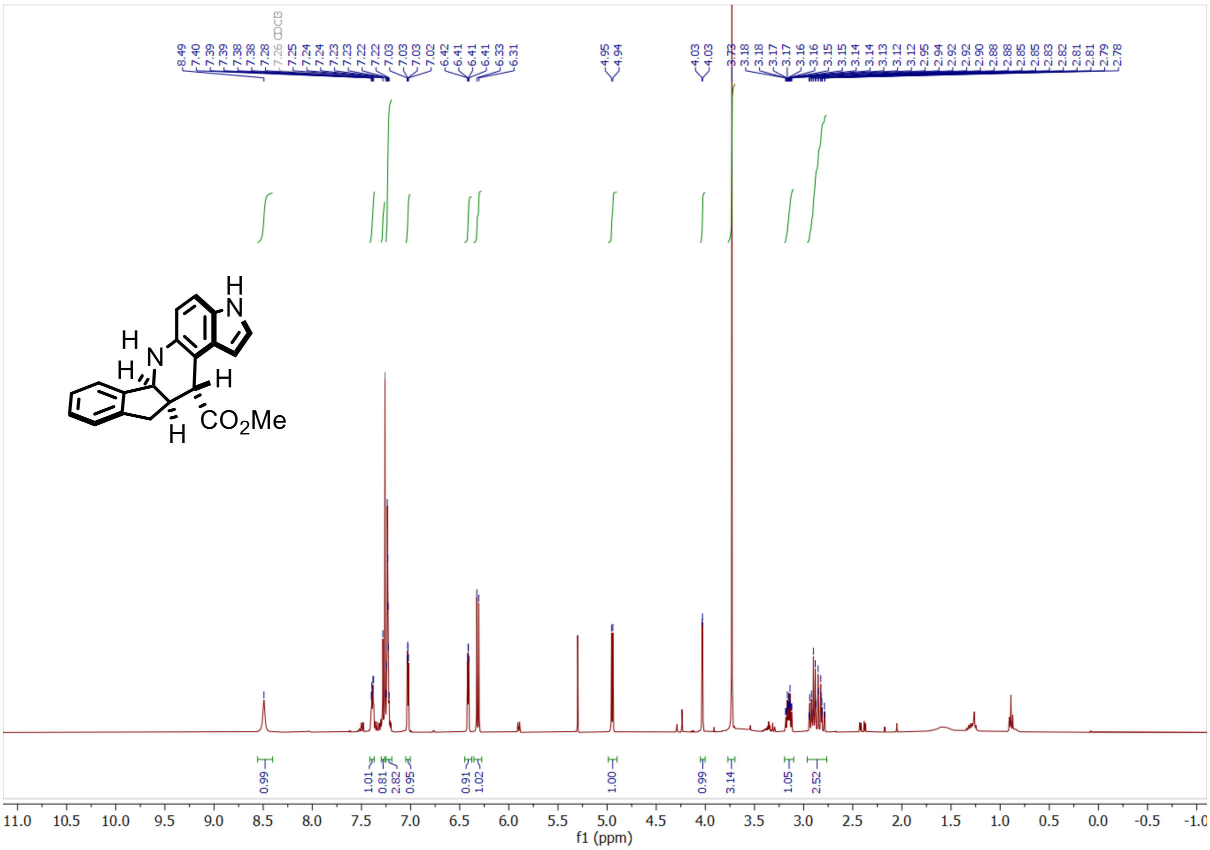
8.49 7.40 7.39 7.39 7.38 7.38 7.28 7.26 CDCl3 7.25 7.24 7.24 7.23 7.23 7.23 7.22 7.22 7.03 7.03 7.03 7.02 6.42 6.41 6.41 6.41 6.33 6.31
4.95 4.94
4.03 4.03
3.73 3.18 3.18 3.17 3.17 3.16 3.16 3.15 3.15 3.14 3.14 3.13 3.12 3.12 2.95 2.94 2.92 2.92 2.90 2.88 2.88 2.85 2.85 2.83 2.82 2.81 2.81 2.79 2.78
H
N
H
N
H
H
H
CO2Me
0.99 1.01 0.81 2.82 0.95 0.91 1.02 1.00 0.99 3.14 1.05 2.52
11.0 10.5 10.0 9.5 9.0 8.5 8.0 7.5 7.0 6.5 6.0 5.5 5.0 4.5 4.0 3.5 3.0 2.5 2.0 1.5 1.0 0.5 0.0 -0.5 -1.0
f1 (ppm)

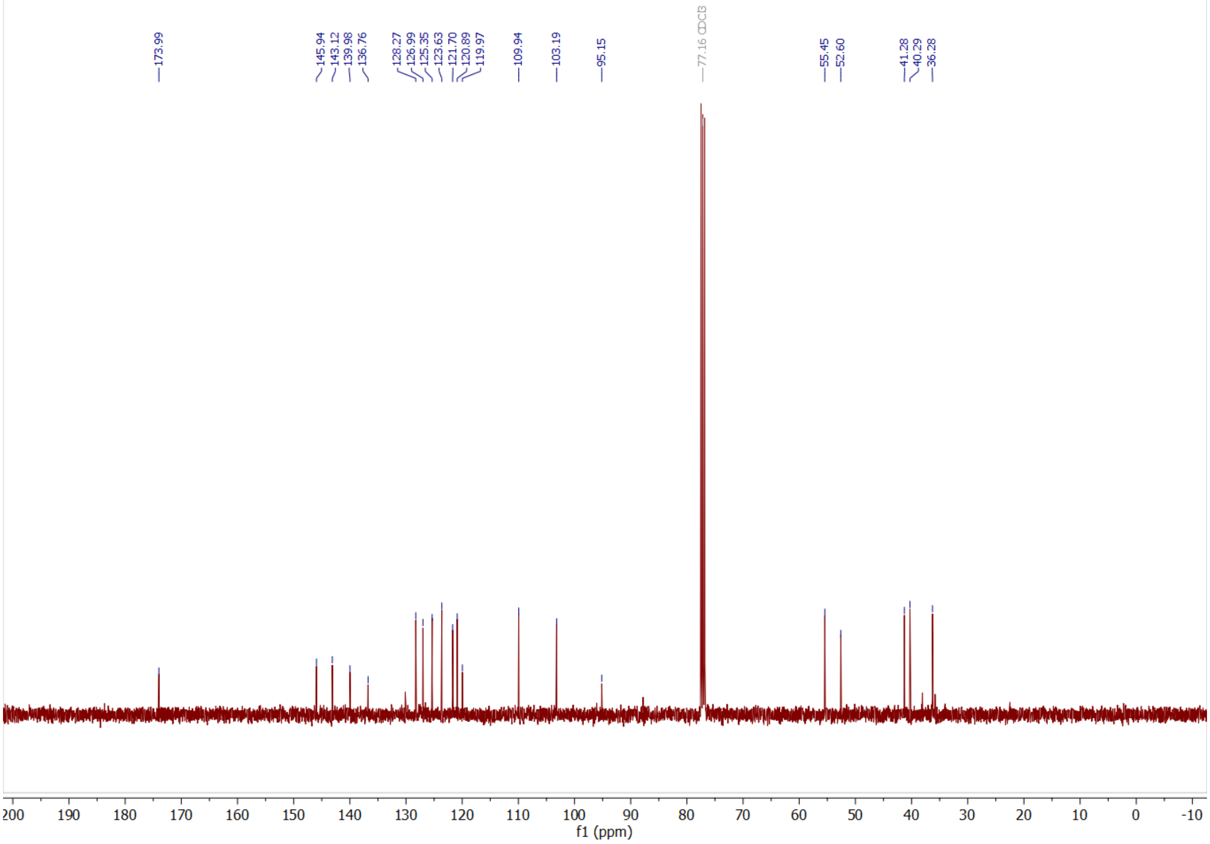
173.99
145.94 143.12 139.98 136.76
128.27 126.99 125.35 123.63 121.70 120.89 119.97
109.94
103.19
95.15
77.16 CDCl3
55.45 52.60
41.28 40.29 36.28
200 190 180 170 160 150 140 130 120 110 100 90 80 70 60 50 40 30 20 10 0 -10
f1 (ppm)

3n

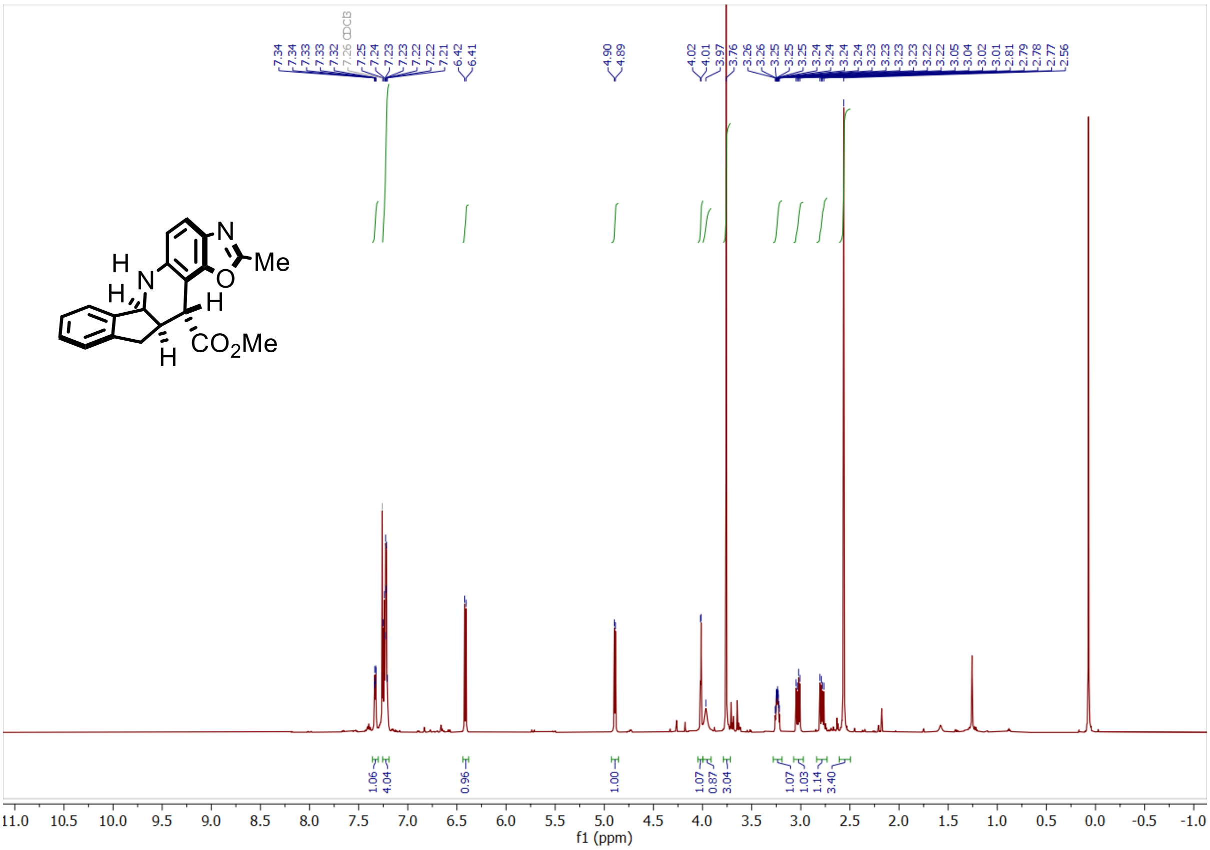

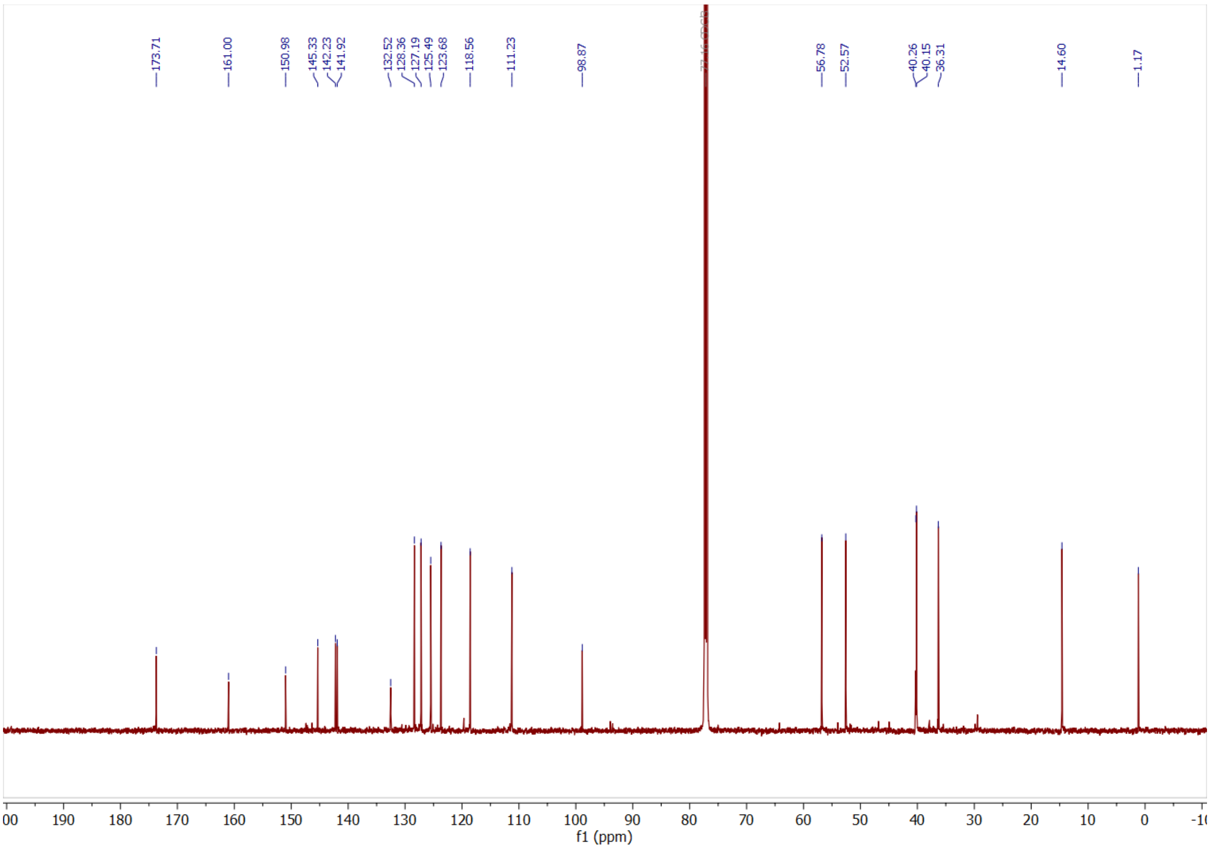

4-aldehyde

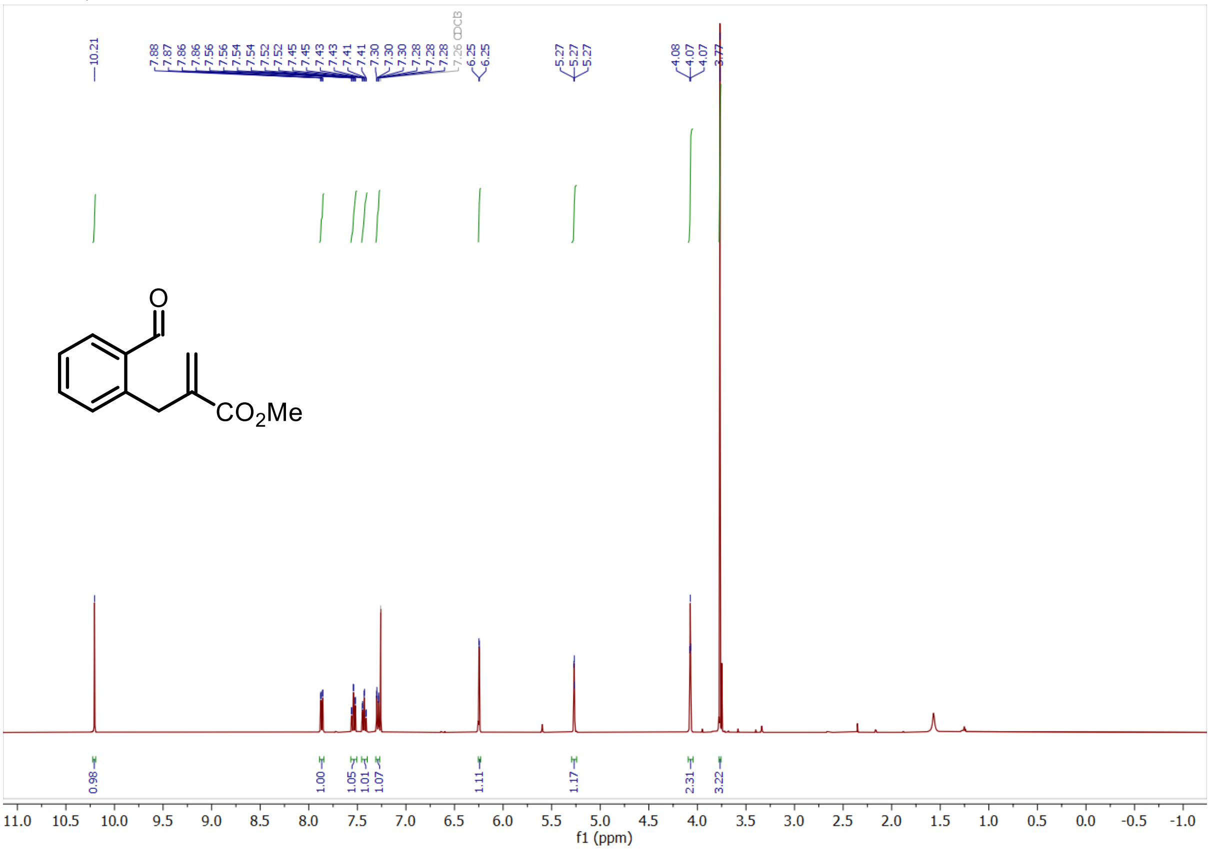
10.21
7.88 7.87 7.86 7.86 7.56 7.56 7.54 7.54 7.52 7.52 7.45 7.45 7.43 7.43 7.41 7.41 7.30 7.30 7.30 7.28 7.28 7.28
7.26 CDCl3
6.25 6.25
5.27 5.27 5.27
4.08 4.07 4.07
3.77
O
CO2Me
0.98
1.00
1.05
1.01
1.07
1.11
1.17
2.31
3.22
11.0 10.5 10.0 9.5 9.0 8.5 8.0 7.5 7.0 6.5 6.0 5.5 5.0 4.5 4.0 3.5 3.0 2.5 2.0 1.5 1.0 0.5 0.0 -0.5 -1.0
f1 (ppm)

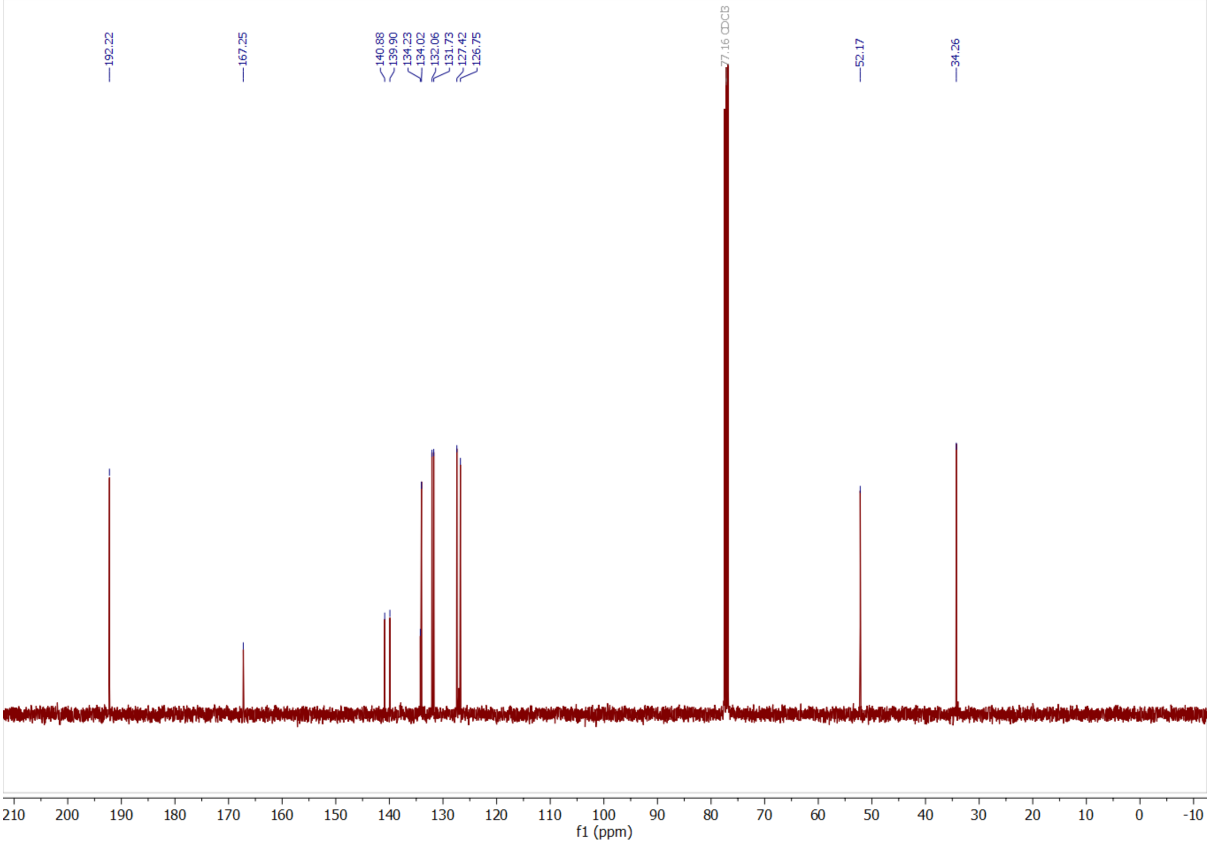
192.22
167.25
140.88 139.90 134.23 134.02 132.06 131.73 127.42 126.75
77.16 CDCl3
52.17
34.26
210 200 190 180 170 160 150 140 130 120 110 100 90 80 70 60 50 40 30 20 10 0 -10
f1 (ppm)

5

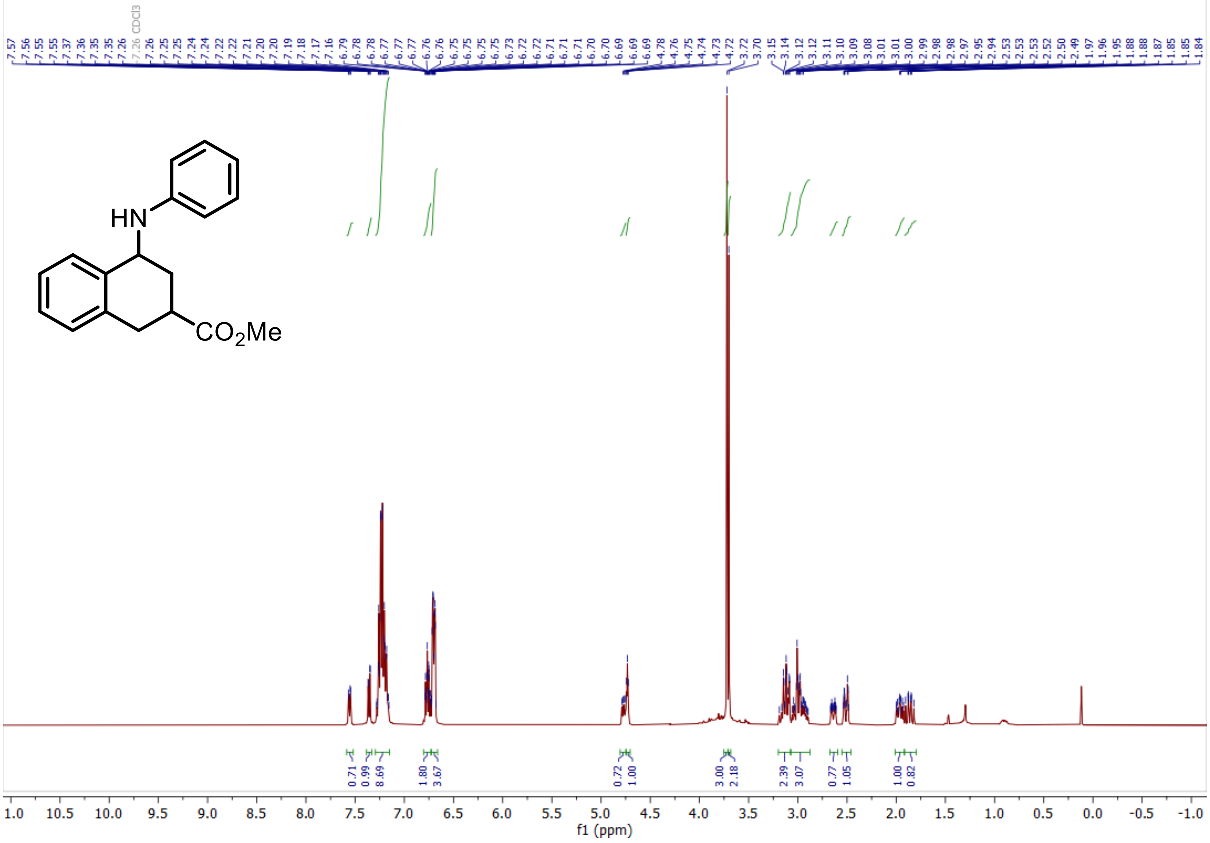

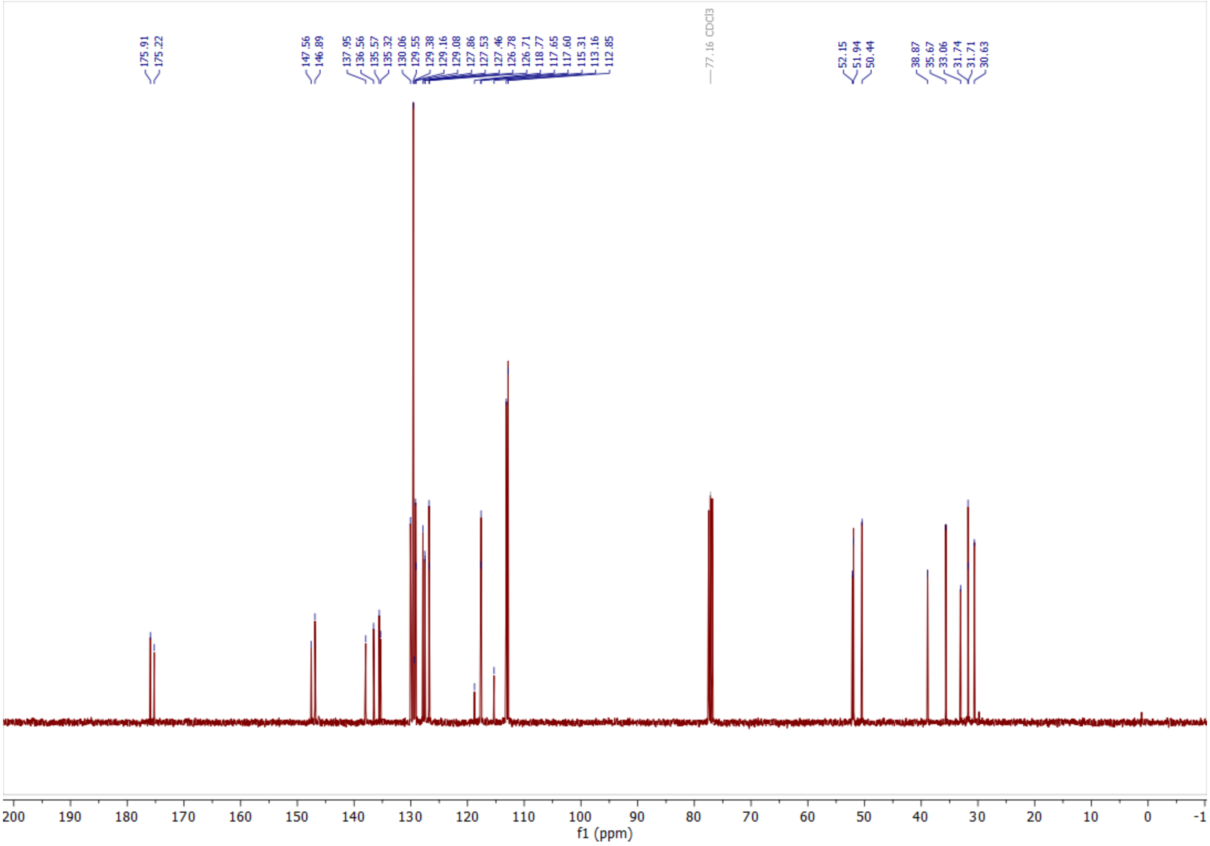

7

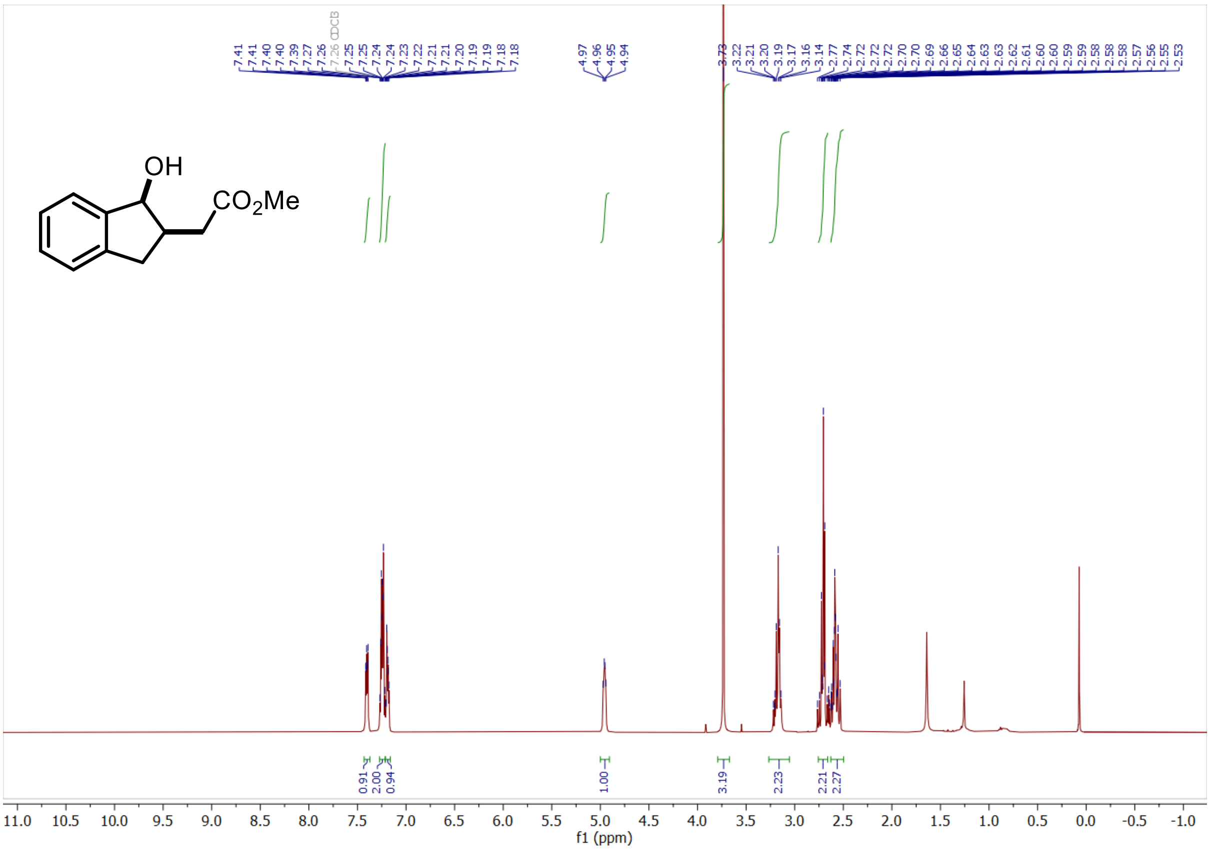
OH
CO2Me
7.41 7.41 7.40 7.39 7.27 7.26 CDCl3 7.25 7.25 7.24 7.24 7.23 7.22 7.21 7.21 7.20 7.19 7.19 7.18 7.18
4.97 4.96 4.95 4.94
3.73 3.22 3.21 3.20 3.19 3.17 3.16 3.14 2.77 2.74 2.72 2.72 2.72 2.70 2.69 2.66 2.65 2.64 2.63 2.62 2.61 2.60 2.59 2.59 2.58 2.58 2.57 2.56 2.55 2.53
0.91 2.00 0.94 1.00 3.19 2.23 2.21 2.27
f1 (ppm)

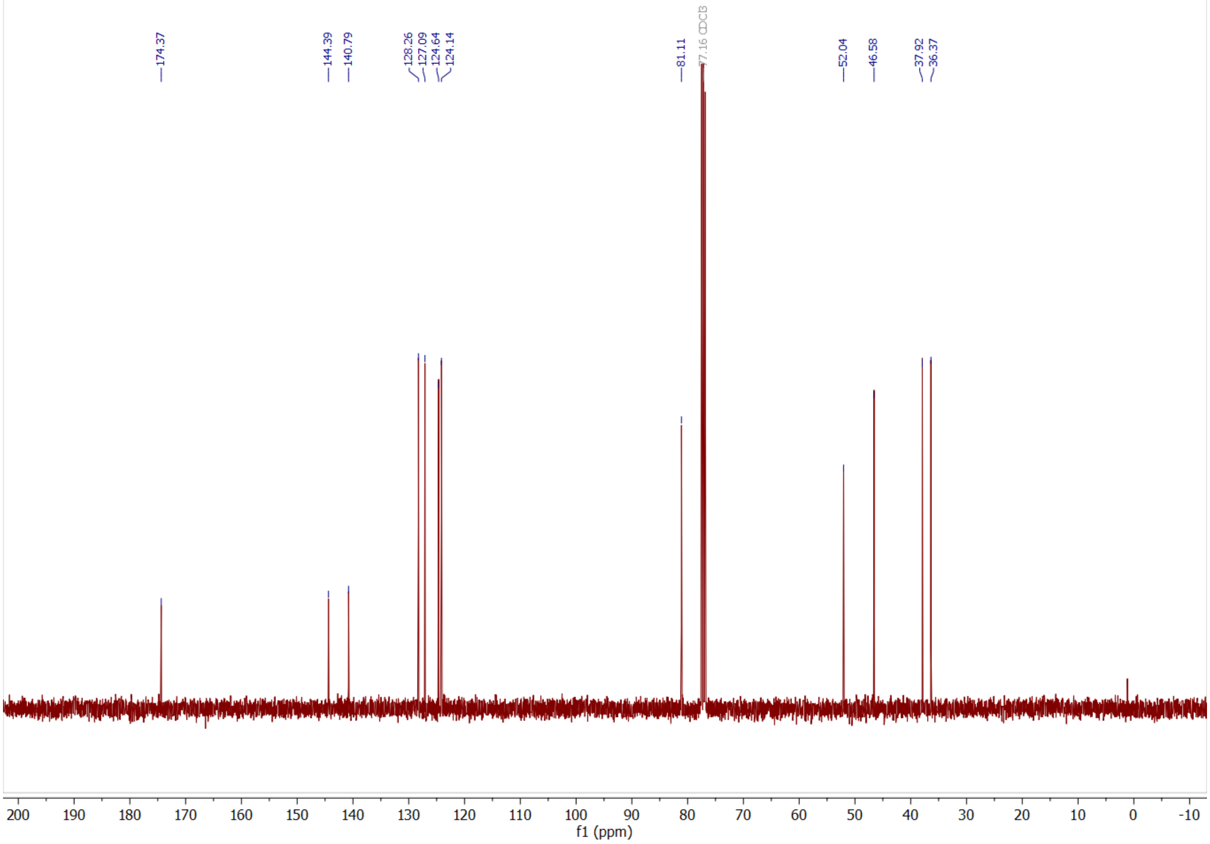
174.37
144.39 140.79
128.26 127.09 124.64 124.14
81.11
77.16 CDCl3
52.04 46.58 37.92 36.37
f1 (ppm)

9a-*trans*

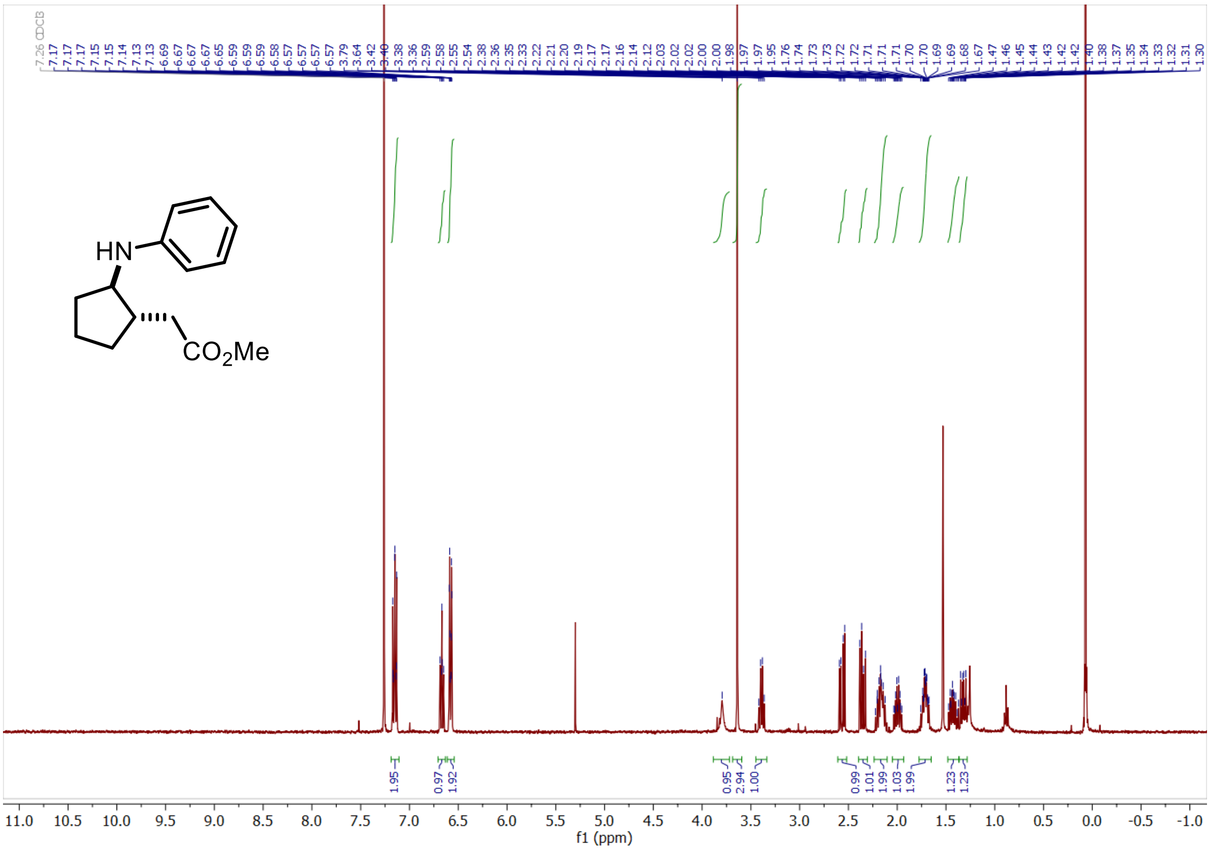

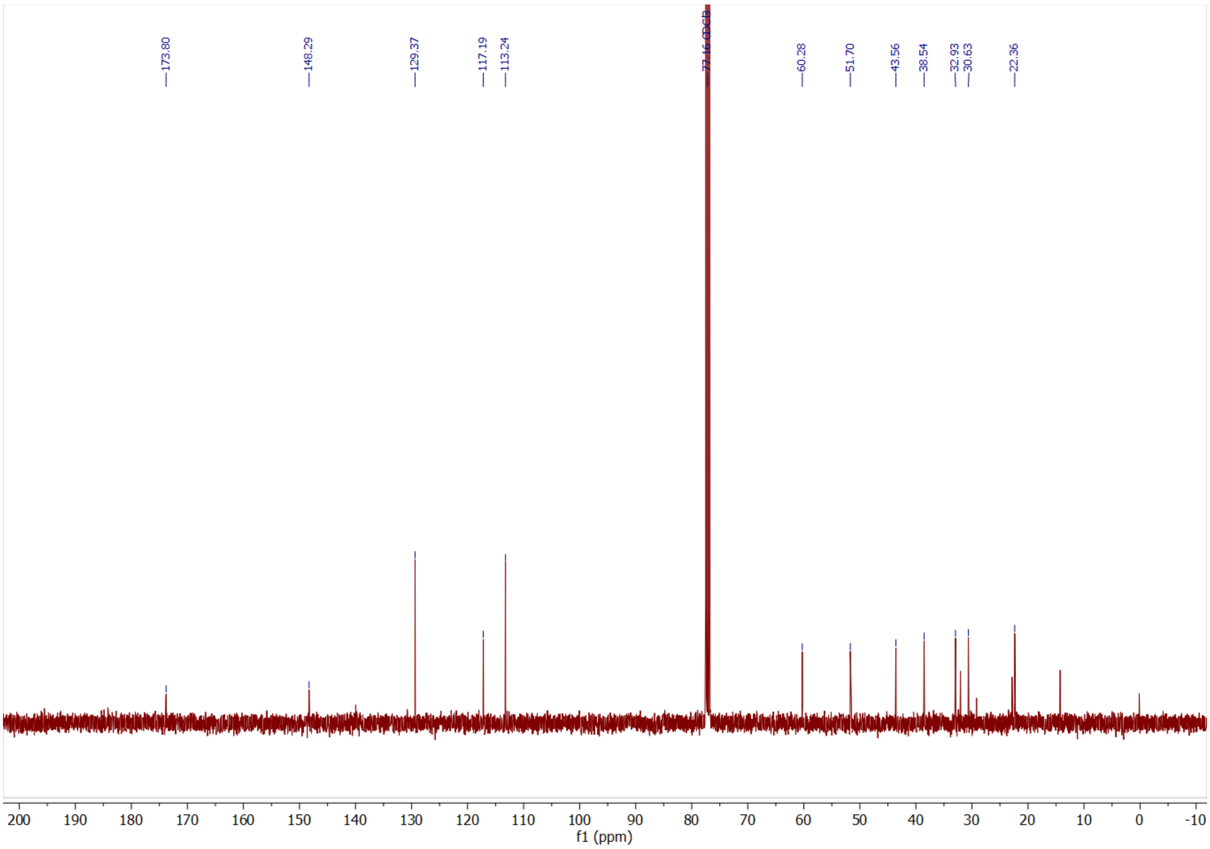

9a-*cis*

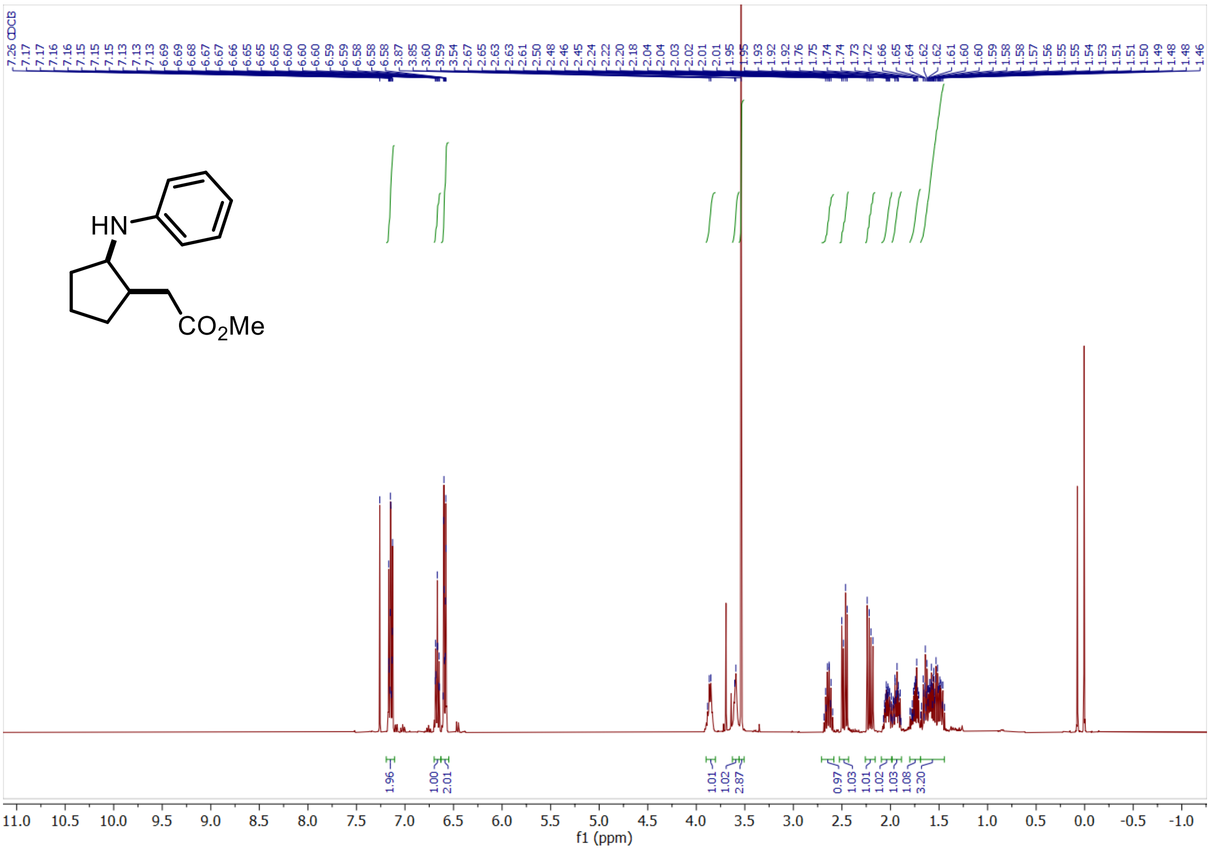

7.26 CDCl3
7.17
7.17
7.16
7.16
7.15
7.15
7.15
7.13
7.13
7.13
6.69
6.69
6.68
6.67
6.67
6.66
6.65
6.65
6.65
6.60
6.60
6.60
6.59
6.59
6.59
6.58
6.58
6.58
3.87
3.85
3.60
3.59
3.54
2.67
2.65
2.63
2.63
2.61
2.50
2.48
2.46
2.45
2.24
2.22
2.20
2.18
2.04
2.04
2.03
2.02
2.01
2.01
1.95
1.95
1.93
1.92
1.92
1.76
1.75
1.74
1.74
1.73
1.72
1.66
1.65
1.64
1.62
1.62
1.61
1.60
1.60
1.59
1.58
1.58
1.57
1.56
1.55
1.55
1.54
1.53
1.51
1.51
1.50
1.49
1.48
1.48
1.46
HN
CO2Me
1.96
1.00
2.01
1.01
1.02
2.87
0.97
1.03
1.01
1.02
1.03
1.08
3.20
11.0 10.5 10.0 9.5 9.0 8.5 8.0 7.5 7.0 6.5 6.0 5.5 5.0 4.5 4.0 3.5 3.0 2.5 2.0 1.5 1.0 0.5 0.0 -0.5 -1.0
f1 (ppm)

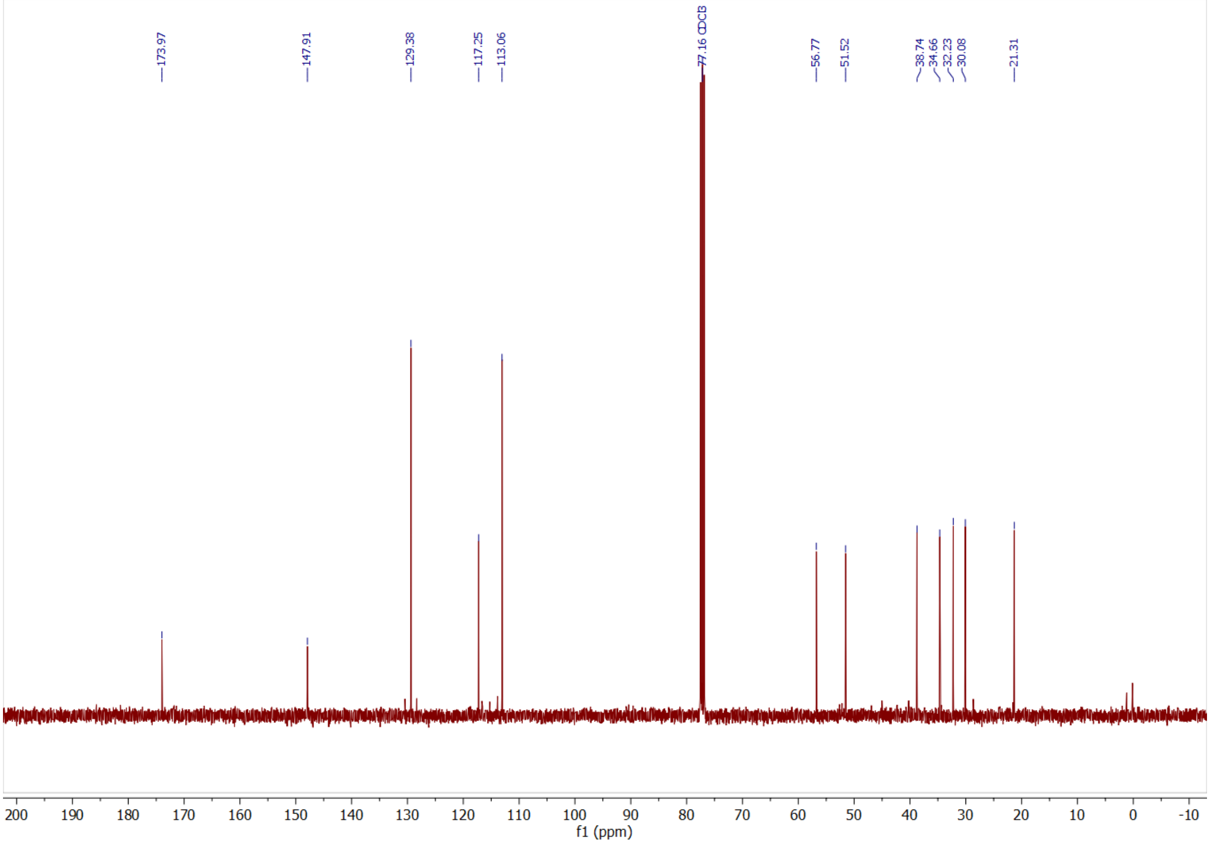

173.97
147.91
129.38
117.25
113.06
77.16 CDCl3
56.77
51.52
38.74
34.66
32.23
30.08
21.31
200 190 180 170 160 150 140 130 120 110 100 90 80 70 60 50 40 30 20 10 0 -10
f1 (ppm)

9b-*trans*

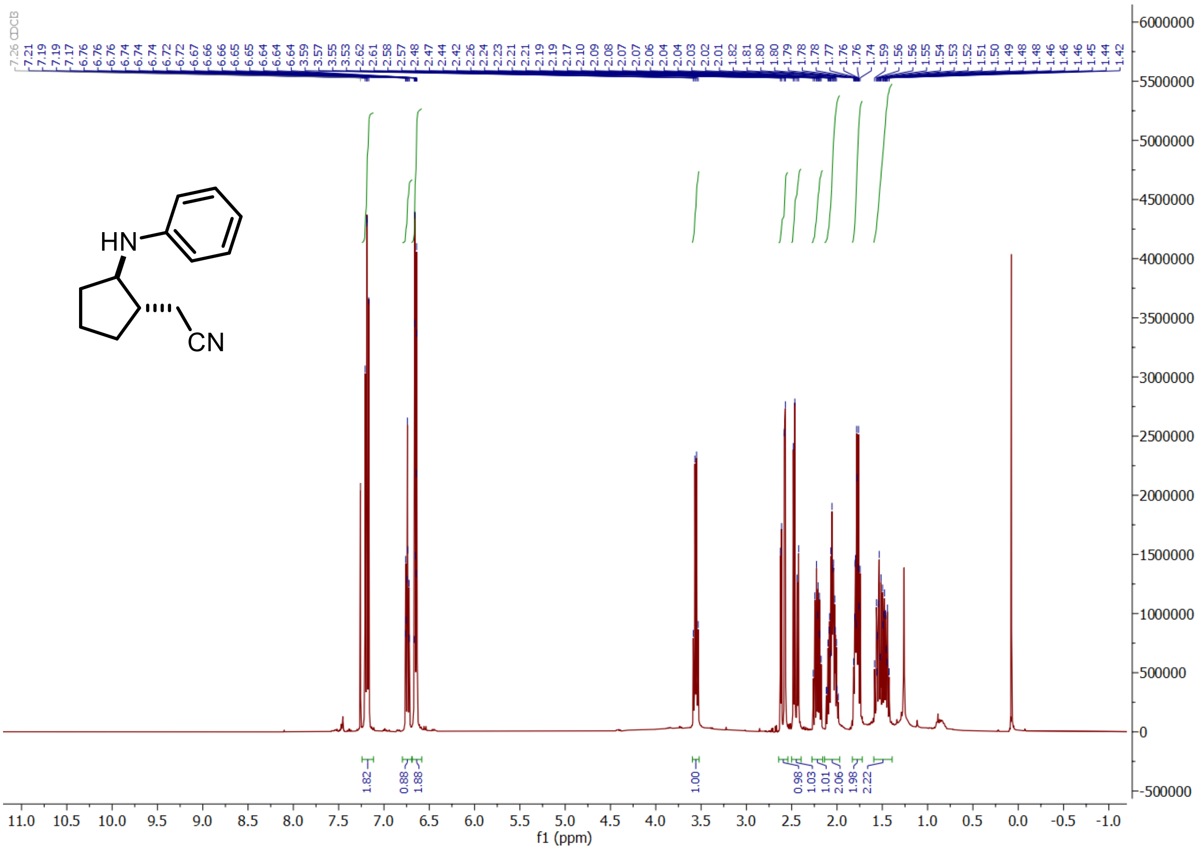

7.26 CDCl3
7.21
7.19
7.19
7.17
6.76
6.76
6.76
6.74
6.74
6.74
6.72
6.72
6.67
6.66
6.66
6.65
6.64
6.64
6.64
3.59
3.57
3.55
3.53
2.62
2.61
2.58
2.57
2.48
2.47
2.44
2.42
2.26
2.24
2.23
2.21
2.21
2.19
2.19
2.17
2.10
2.09
2.08
2.07
2.07
2.06
2.04
2.04
2.03
2.02
2.01
1.82
1.81
1.80
1.80
1.79
1.78
1.78
1.77
1.76
1.76
1.74
1.59
1.56
1.56
1.55
1.54
1.53
1.52
1.51
1.50
1.49
1.48
1.48
1.46
1.46
1.45
1.44
1.42
HN
CN
1.82
0.88
1.88
1.00
0.98
1.03
1.01
2.06
1.98
2.22
f1 (ppm)

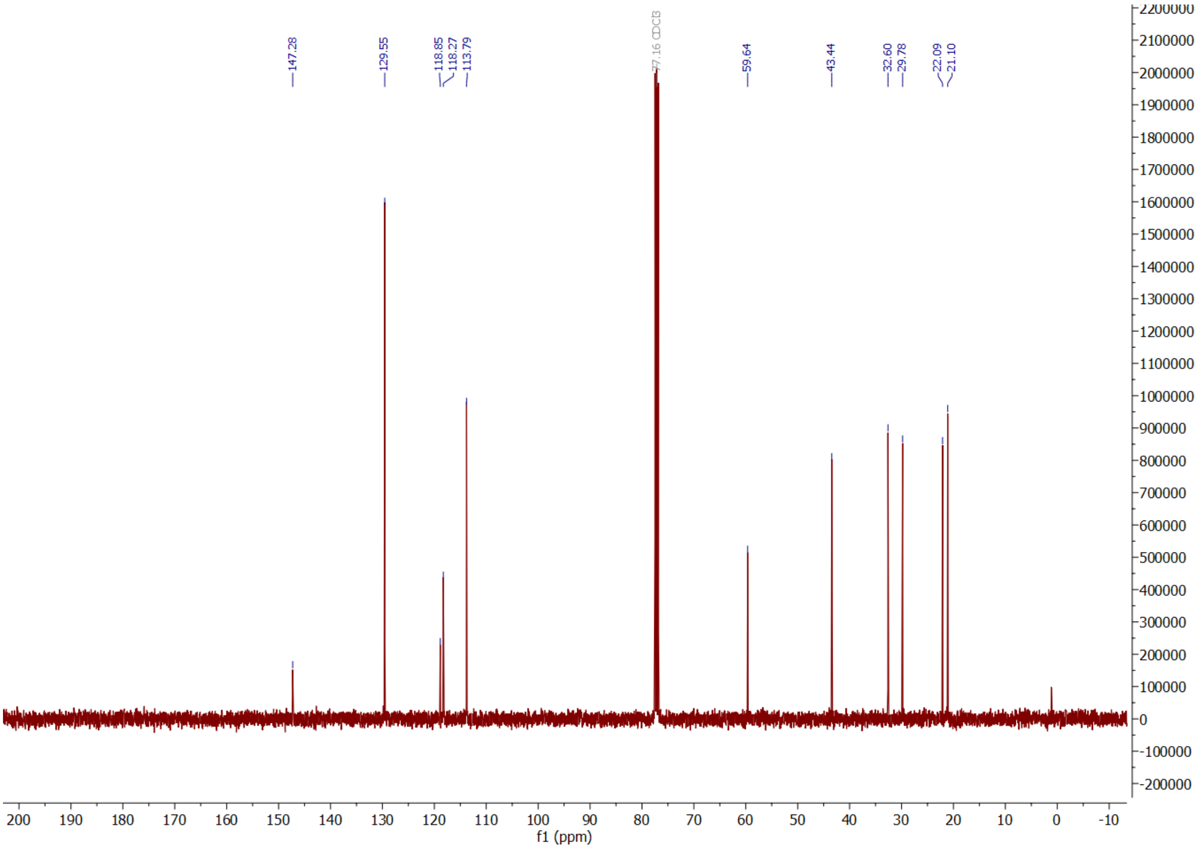

147.28
129.55
118.85
118.27
113.79
77.16 CDCl3
59.64
43.44
32.60
29.78
22.09
21.10
f1 (ppm)

9b-*cis*

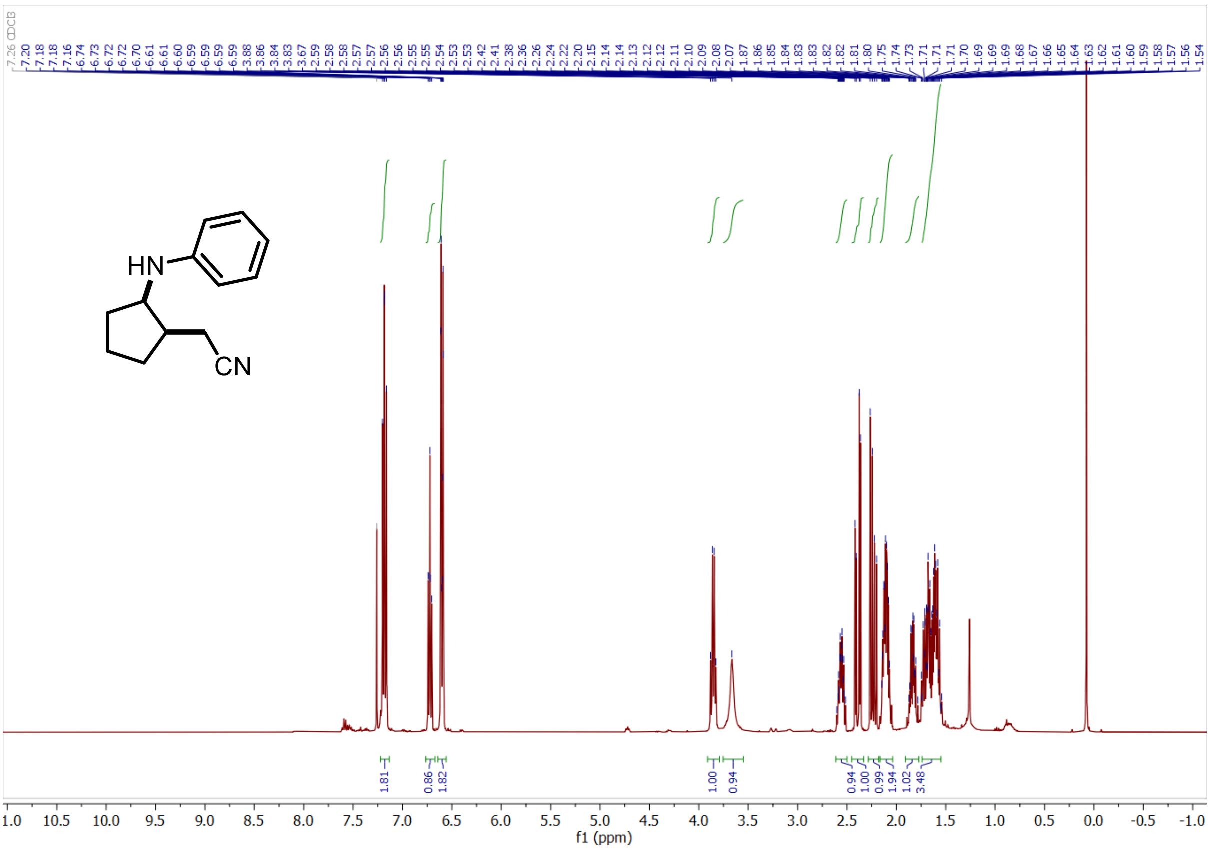
7.26 CDCl3
7.20 7.18 7.18 7.16 6.74 6.73 6.72 6.70 6.61 6.60 6.59 6.59 6.59 6.59 3.88 3.86 3.84 3.83 3.67 2.59 2.58 2.58 2.57 2.57 2.56 2.56 2.55 2.55 2.54 2.53 2.53 2.42 2.41 2.38 2.36 2.26 2.24 2.22 2.20 2.15 2.14 2.13 2.12 2.12 2.11 2.10 2.09 2.08 2.07 1.87 1.86 1.85 1.84 1.83 1.83 1.82 1.82 1.81 1.80 1.75 1.74 1.73 1.71 1.71 1.71 1.70 1.69 1.69 1.69 1.68 1.67 1.66 1.65 1.64 1.63 1.62 1.61 1.60 1.59 1.58 1.57 1.56 1.54
HN
CN
1.81
0.86
1.82
1.00
0.94
0.94
1.00
0.99
1.94
1.02
3.48
f1 (ppm)

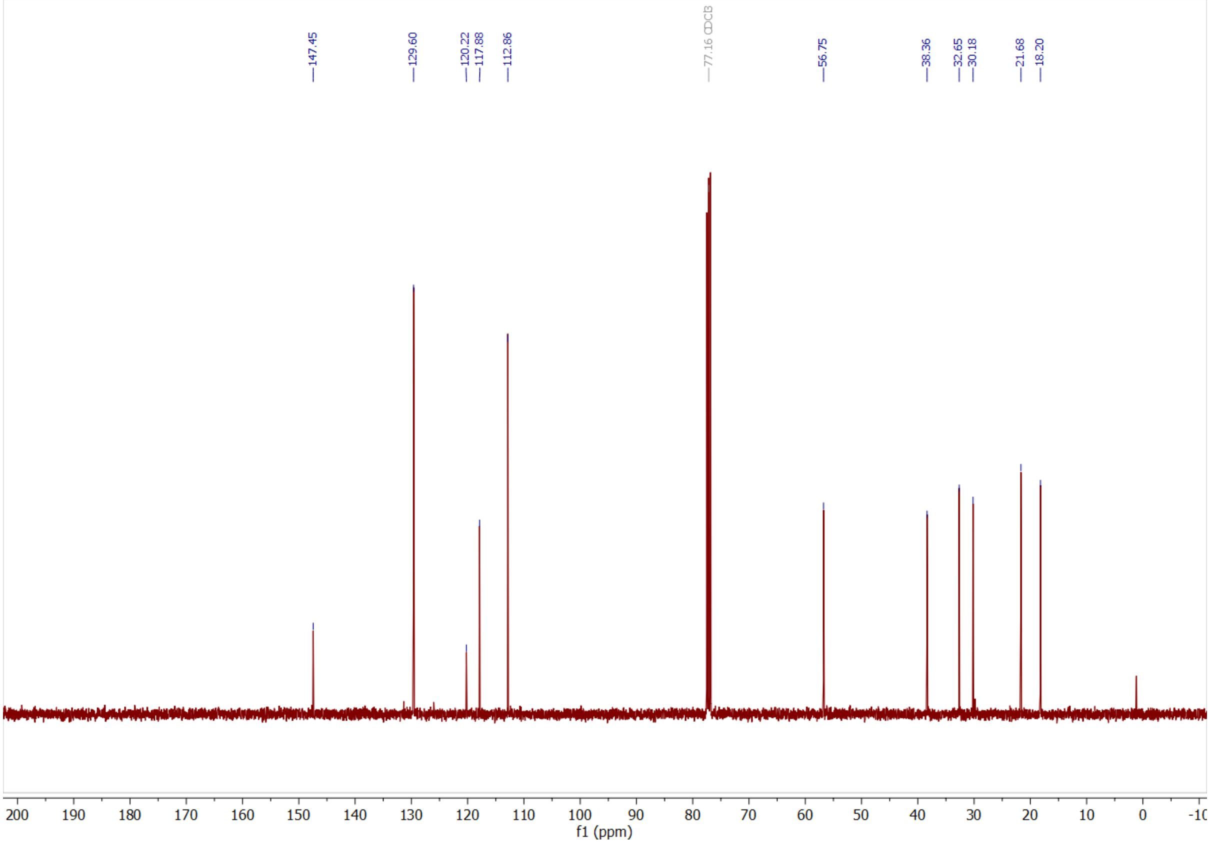
147.45
129.60
120.22
117.88
112.86
77.16 CDCl3
56.75
38.36
32.65
30.18
21.68
18.20
f1 (ppm)

S1

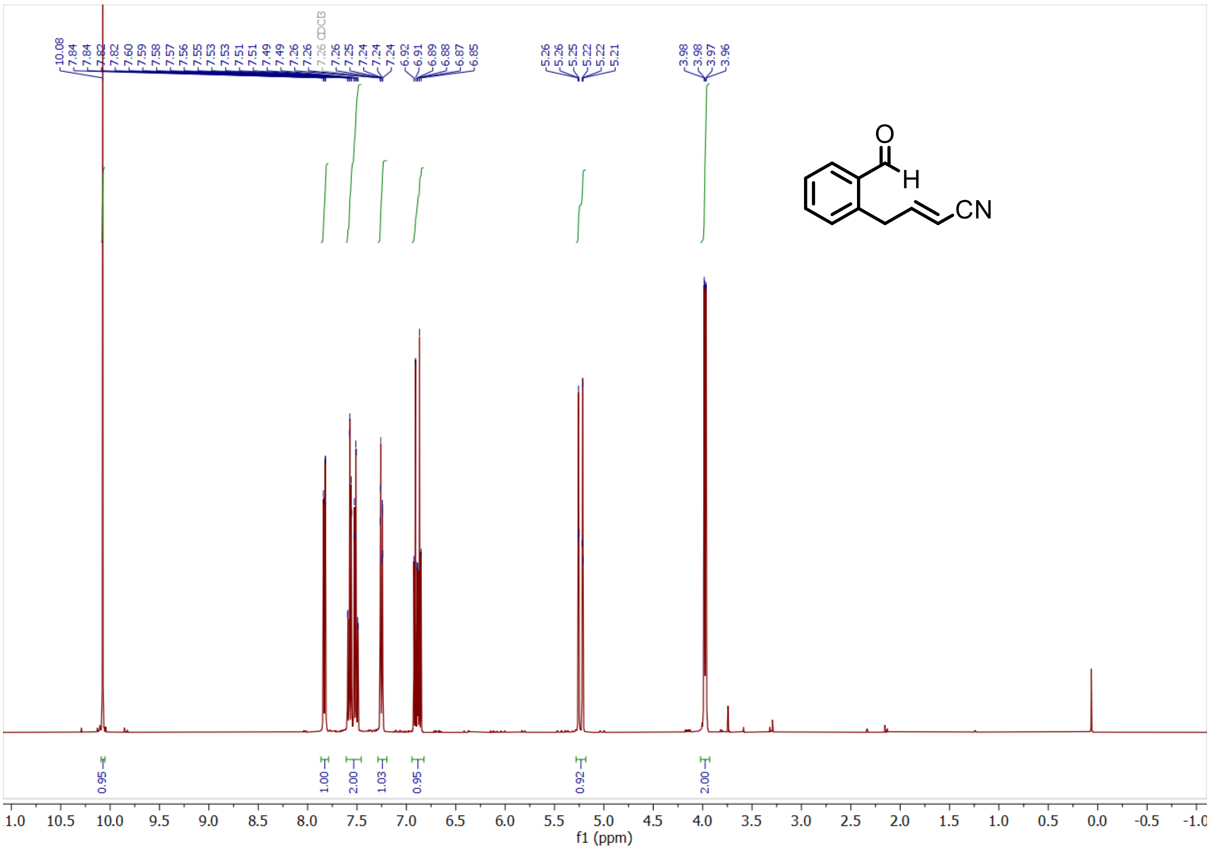
10.08 7.84 7.84 7.82 7.82 7.60 7.59 7.58 7.57 7.56 7.55 7.53 7.53 7.51 7.51 7.49 7.49 7.26 7.26 7.26 CDCl3 7.26 7.25 7.24 7.24 7.24 6.92 6.91 6.89 6.88 6.87 6.85
5.26 5.26 5.25 5.22 5.22 5.21
3.98 3.98 3.97 3.96
O
H
CN
0.95
1.00
2.00
1.03
0.95
0.92
2.00
f1 (ppm)

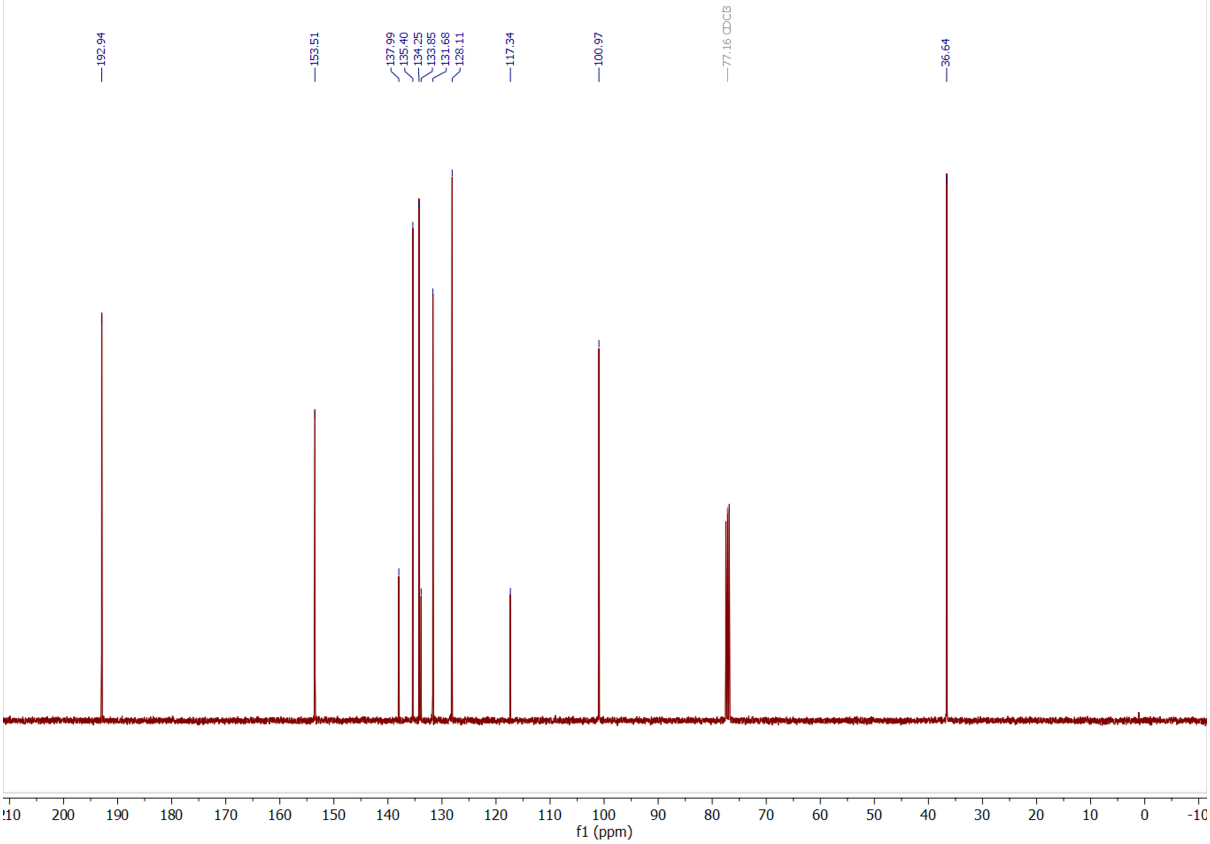
192.94
153.51
137.99 135.40 134.25 133.85 131.68 128.11
117.34
100.97
77.16 CDCl3
36.64
f1 (ppm)

S2

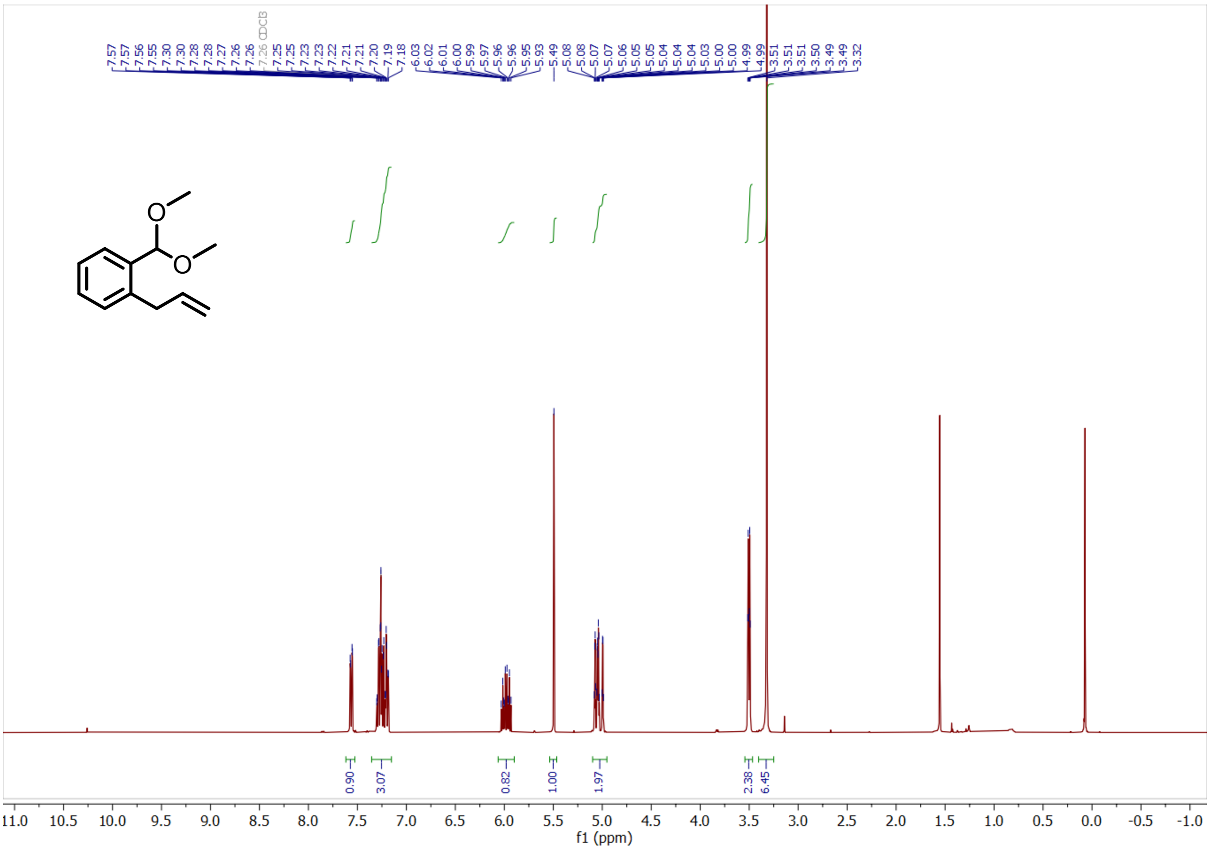
7.57
7.57
7.56
7.55
7.30
7.30
7.28
7.28
7.27
7.26
7.26 CDCl3
7.25
7.25
7.23
7.23
7.22
7.21
7.21
7.20
7.19
7.18
6.03
6.02
6.01
6.00
5.99
5.97
5.96
5.96
5.95
5.93
5.49
5.08
5.08
5.07
5.07
5.06
5.05
5.05
5.04
5.04
5.04
5.03
5.00
5.00
5.00
4.99
4.99
3.51
3.51
3.51
3.50
3.49
3.49
3.32
0.90
3.07
0.82
1.00
1.97
2.38
6.45
f1 (ppm)

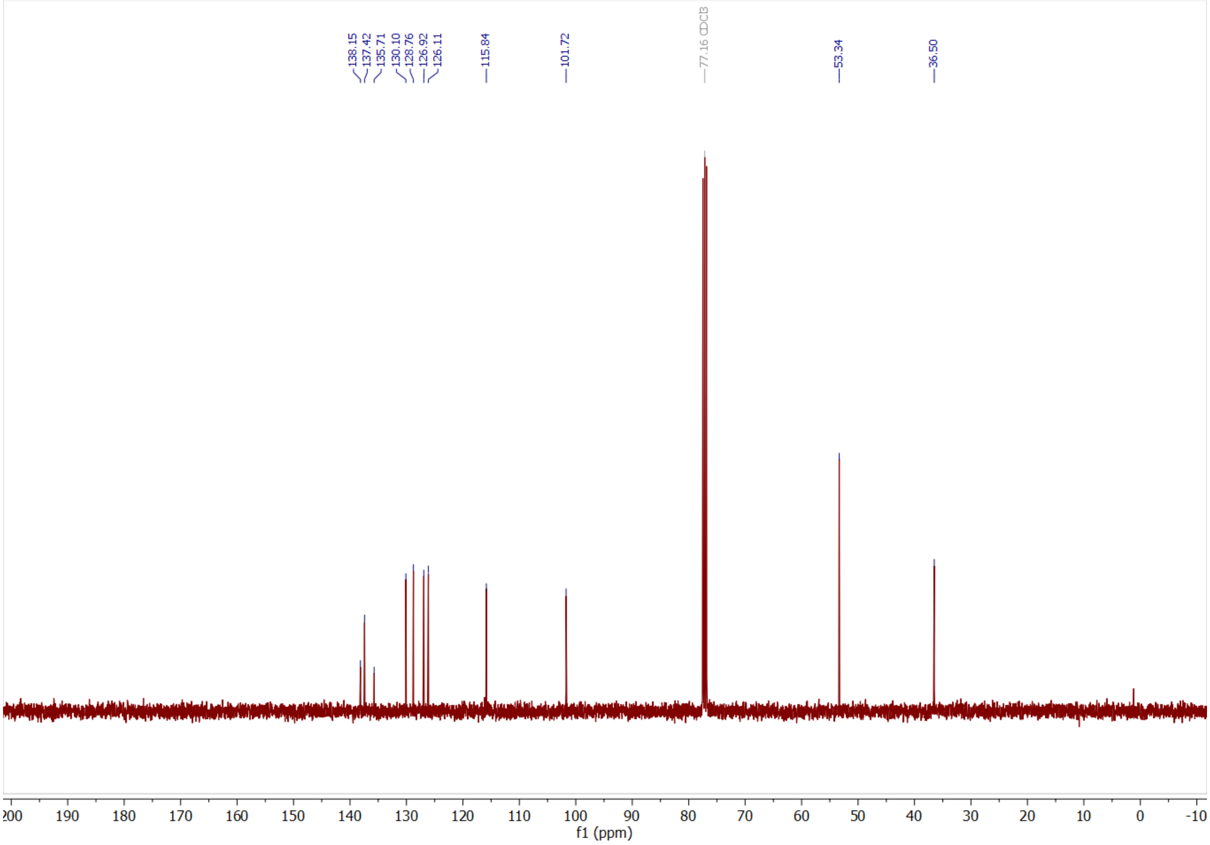
138.15
137.42
135.71
130.10
128.76
126.92
126.11
115.84
101.72
77.16 CDCl3
53.34
36.50
f1 (ppm)

S3

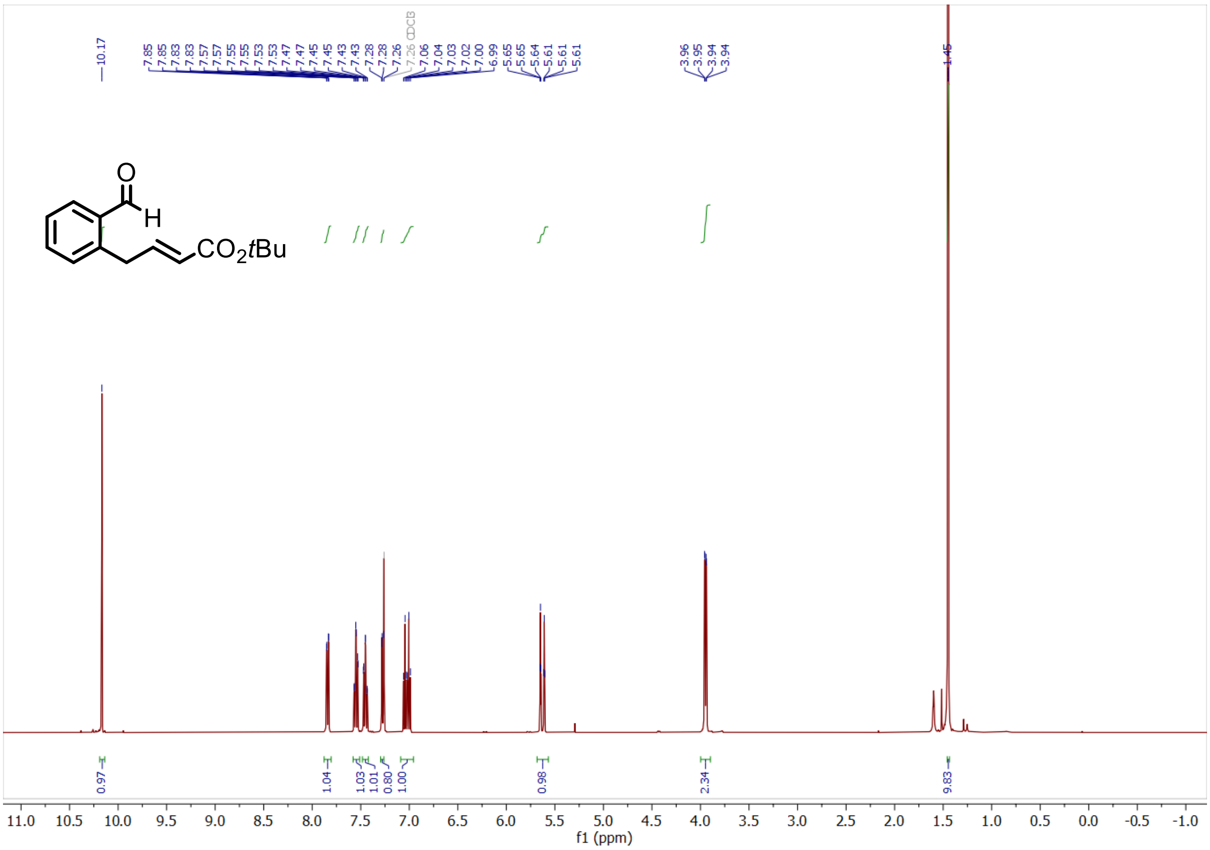
CO2tBu
H
O
10.17
7.85
7.85
7.83
7.83
7.57
7.57
7.55
7.55
7.53
7.53
7.47
7.47
7.45
7.45
7.43
7.43
7.28
7.28
7.26 CDCl3
7.06
7.04
7.03
7.02
7.00
6.99
5.65
5.65
5.64
5.61
5.61
5.61
3.96
3.95
3.94
3.94
1.45
0.97
1.04
1.03
1.01
0.80
1.00
0.98
2.34
9.83
11.0 10.5 10.0 9.5 9.0 8.5 8.0 7.5 7.0 6.5 6.0 5.5 5.0 4.5 4.0 3.5 3.0 2.5 2.0 1.5 1.0 0.5 0.0 -0.5 -1.0
f1 (ppm)

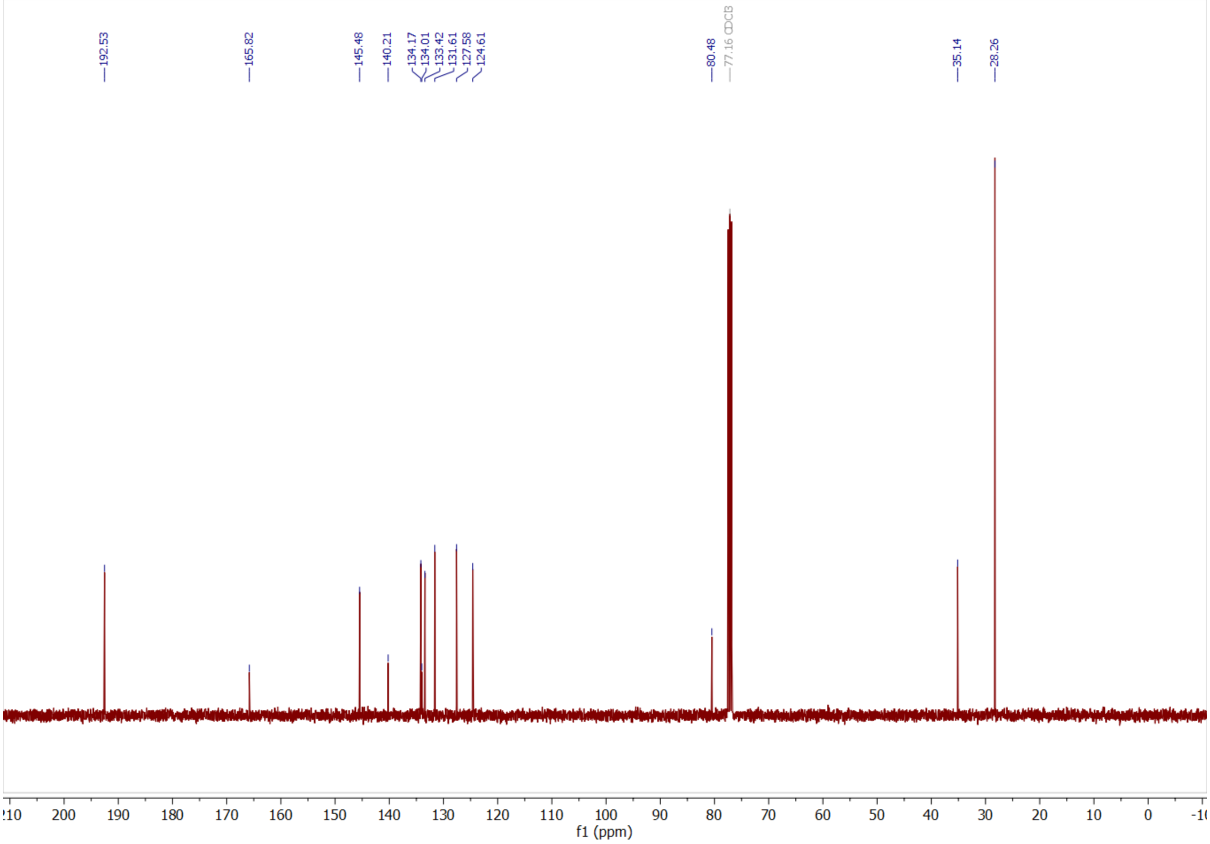
192.53
165.82
145.48
140.21
134.17
134.01
133.42
131.61
127.58
124.61
80.48
77.16 CDCl3
35.14
28.26
210 200 190 180 170 160 150 140 130 120 110 100 90 80 70 60 50 40 30 20 10 0 -10
f1 (ppm)

S4

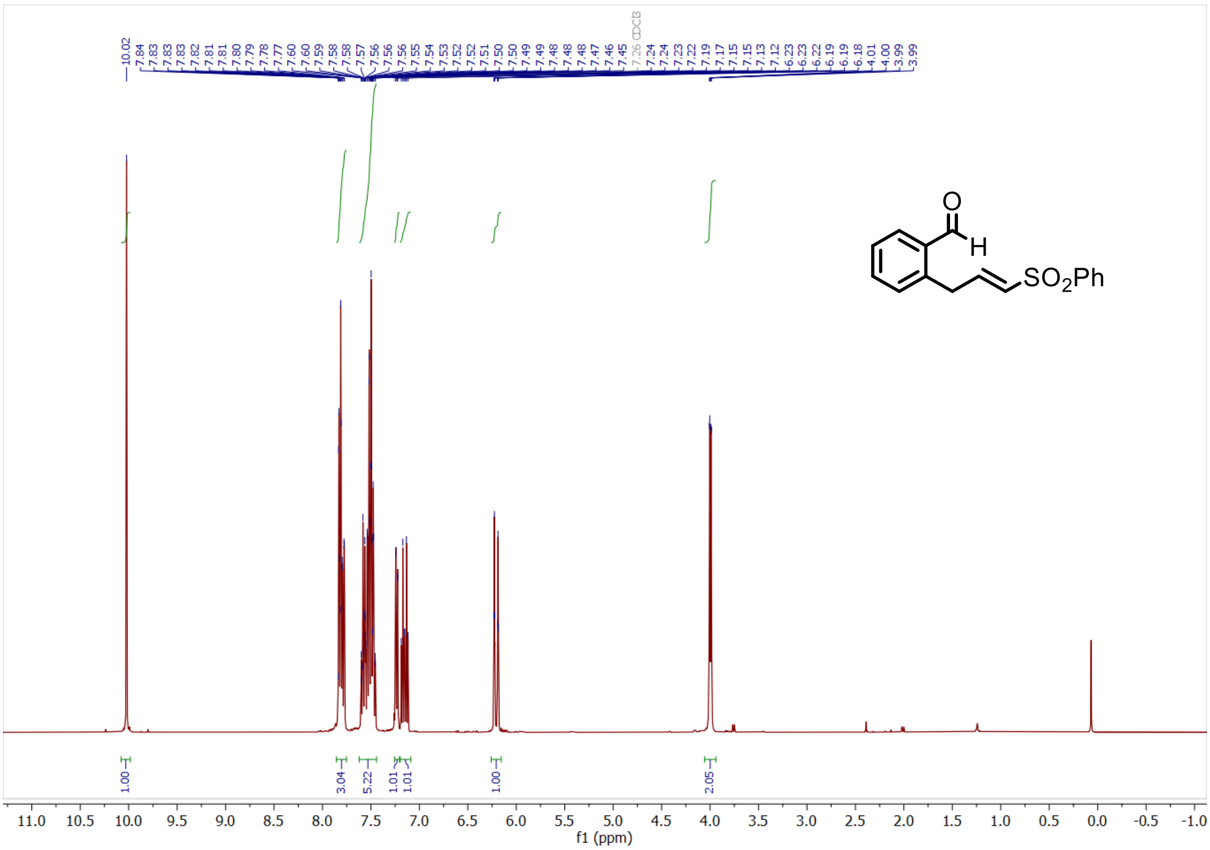

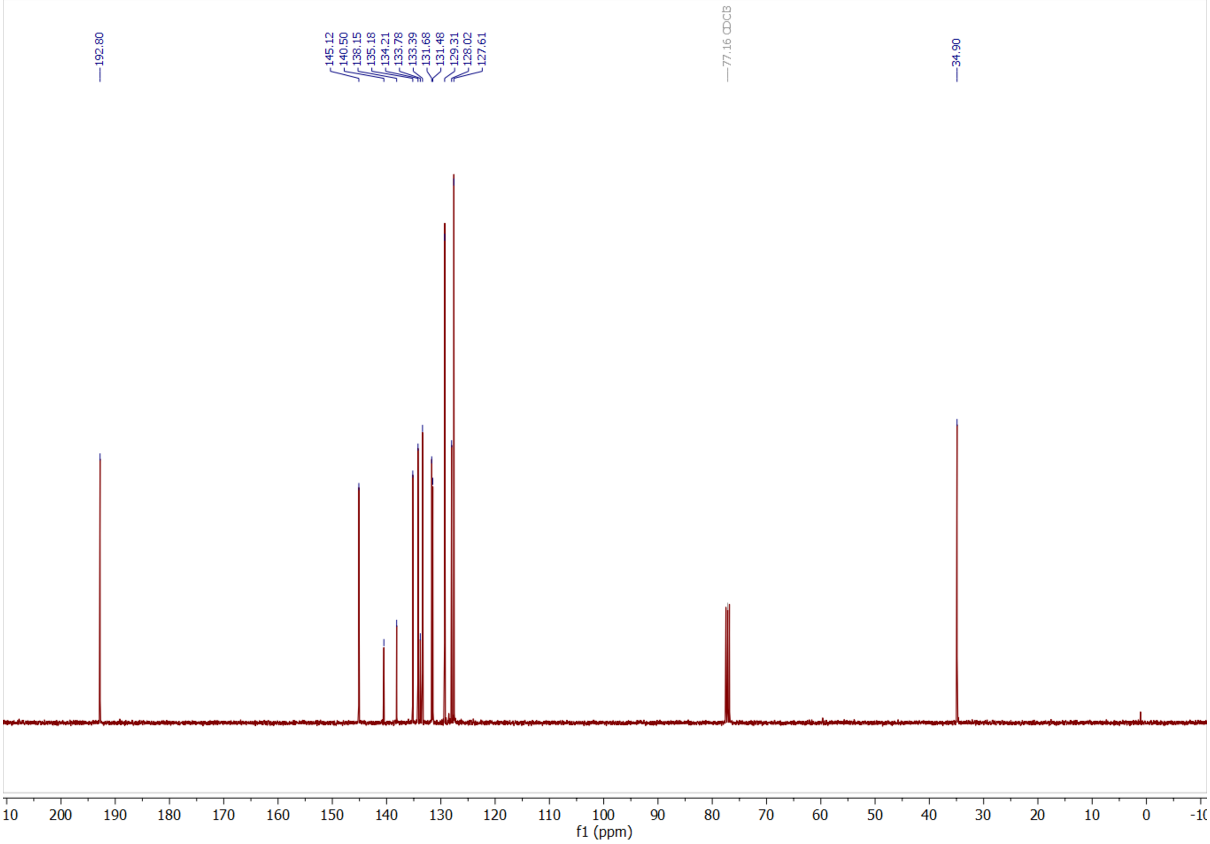

Supplement: Supplementary file 1 — Supporting Information [file ANIE-60-24116-s001.pdf]
